# Caylobolide B: Structure Revision, Total Synthesis, Biological Characterization, and Discovery of New Analogues

**DOI:** 10.1002/anie.202523117

**Published:** 2025-12-10

**Authors:** Malcolm R. P. George, Lobna A. Elsadek, Max Deering, Larissa Costa de Almeida, Jasper L. Tyler, Adam Noble, Valerie J. Paul, Hendrik Luesch, Craig P. Butts, Varinder K. Aggarwal

**Affiliations:** ^1^ School of Chemistry University of Bristol Cantock's Close Bristol BS8 1TS UK; ^2^ Department of Medicinal Chemistry and Center for Natural Products Drug Discovery and Development (CNPD3) University of Florida PO Box 100485 Gainesville FL 32610 USA; ^3^ Smithsonian Marine Station 701 Seaway Drive Fort Pierce FL 34949 USA; ^4^ Program in Cancer and Stem Cell Biology Duke‐NUS Medical School Singapore 169857 Singapore

**Keywords:** Natural products, Chemogenomic profiling, Marine macrolides, Structural elucidation, Total synthesis

## Abstract

The unique potential of marine polyhydroxylated macrolides in chemical biology and drug discovery has long been constrained by their structural complexity and limited material availability, frustrating efforts in stereochemical assignment, synthesis, and mechanism‐of‐action elucidation. Here, we establish an integrated workflow, combining chemogenomic profiling, ultra‐high‐resolution NMR, and modular total synthesis, for the comprehensive functional and structural interrogation of this challenging natural product class. Applying this approach to caylobolides, natural products isolated from scarce samples of *Okeania* sp., we performed structure‐activity relationship studies revealing that acetylation at C29 markedly reduces both cytotoxicity and antifungal activity, pinpointing a key pharmacophore. Mechanistic profiling suggests that these macrolides disrupt membrane integrity, similar to amantelide A. Using natural compound samples, we simultaneously revised the structure of caylobolide B through ^1^H, 1D‐selective TOCSY and HSQC NMR, and developed a modular fragment‐based synthesis of these compounds. By providing a unified methodology for genetic sensitivity profiling, precise structure and stereochemistry determination, and modular total synthesis, this work unlocks new opportunities for the discovery and rational design of potent marine‐derived therapeutics.

## Graphical abstract

Marine polyhydroxylated macrolides’ drug discovery potential is limited by structural complexity and scarce material supply, hindering structure assignment, synthesis, and biological studies. Here, we present an integrated workflow that combines chemogenomic profiling, ultra‐high‐resolution NMR‐guided structural revision and stereochemical assignment, and modular total synthesis to enable comprehensive interrogation of the caylobolide B family.

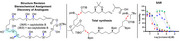

Marine cyanobacteria are a rich source of bioactive natural products, many of which feature unique chemical structures and exhibit potent biological activities.^[^ ^1^ ^]^ Among these, polyhydroxylated macrolides such as bastimolide A^[^ ^2^ ^]^ and B,^[^ ^3^ ^]^ amantelide A and B,^[^ ^4^ ^]^ caylobolide A^[^ ^5^ ^]^ and B,^[^ ^6^, ^7^ ^]^ and palstimolide^[^ ^8^ ^]^ stand out for their remarkable structural diversity and pharmacological promise, garnering significant attention towards their structural elucidation, biological evaluation, and synthetic production (Figure 1a).^[^ ^2^, ^3^, ^4^, ^9^, ^10^ ^]^ The ability to accurately assign stereochemical configurations, and access these molecules by synthesis, is critical for both understanding their biological function and enabling drug discovery efforts. However, the structural elucidation of these macrolides has been hampered by their highly repetitive polyol motifs, which complicate conventional spectroscopic analysis and often lead to misassignment of key skeletal connectivity.^[^ ^6^ ^]^ The extreme difficulty of stereochemical elucidation, in tandem with limited sample availability, can lead to a choice between biological evaluation and structural assignment of these molecules, especially as conventional elucidation techniques degrade and/or irreversibly consume precious sample.^[^ ^9^, ^11^, ^12^, ^13^, ^14^ ^]^

**Figure 1:**
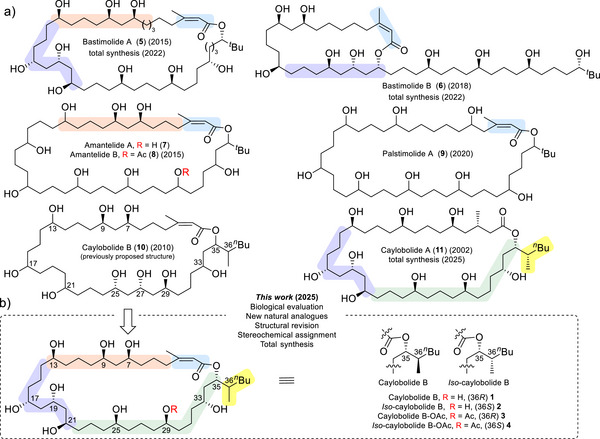
Structurally related marine macrolides possessing a 1,3‐ and 1,5‐polyol backbone: a) bastimolide A (**5**) and B (**6**), amantelide A (**7**) and B (**8**), palstimolide A (**9**), caylobolide A (**11**), and caylobolide B (previously reported structure, **10**).^[^ ^2^, ^3^, ^4^, ^5^, ^6^, ^7^, ^8^ ^]^ b) This work: solving the stereochemical assisgnment of caylobolide B (**1**), *iso*‐caylobolide B (**2**), caylobolide B‐OAc (**3**), and *iso*‐caylobolide B‐OAc (**4**). Regions of similarity between caylobolide B and other marine macrolides are highlighted.

Caylobolide B, initially isolated from marine cyanobacteria in Florida, exemplifies these challenges. Although reported over a decade ago, its complete stereochemical configuration has remained uncertain, impeding both biological studies and synthetic access.^[^ ^6^ ^]^ In a recent publication, we demonstrated that a combination of ultra‐high‐resolution NMR spectroscopy, Mosher's ester analysis, and mixed isomer syntheses could be used to elucidate the structure and stereochemistry of the related marine macrolide caylobolide A.^[^ ^15^ ^]^ However, the necessity to form the Mosher ester adduct meant that structural elucidation required the consumption of the available natural product material which could therefore no longer be used in bioactivity studies. Furthermore, the unresolved stereochemical ambiguity, which could not be clarified through spectroscopic analysis, necessitated the preparation of multiple natural product diastereomers, representing a substantial experimental undertaking.

Herein, we establish an integrated workflow that employs our recently established NMR‐based method for 1,5‐polyol stereochemical assignment,^[^ ^16^ ^]^ combined with Kishi's universal NMR database^[^ ^17^ ^]^ and ultra‐high‐resolution NMR spectroscopy, to solve the structure and stereochemistry of caylobolide B while preserving the isolated material for biological evaluation (Figure 1b). Consequently, we simultaneously establish the bioactivity profile of caylobolide B, defining a conserved pharmacophore and revealing new insights into structure‐activity relationships. Chemogenomic profiling with *iso*‐caylobolide B (its C36 epimer) further illuminated the mechanism‐of‐action of this compound class. In addition, we report the first total synthesis of caylobolide B and *iso*‐caylobolide B.

Cytotoxicity‐guided fractionation of *Okeania* sp. samples collected from Tumon Bay, Guam, coupled with HR‐LCMS dereplication indicated the presence of caylobolide B^[^ ^6^ ^]^ and an acetylated derivative. ^1^H NMR then revealed that each compound co‐existed with a stereoisomer, as evidenced by methyl proton chemical shift changes and the resulting multiplet collapse. Consequently, we purified 4 discrete compounds from this sample: caylobolide B (**1**), *iso*‐caylobolide B (**2**), caylobolide B‐OAc (**3**), and *iso*‐caylobolide B‐OAc (**4**)

To determine which structural features govern function, we next evaluated cytotoxic and antifungal activities and probed the mechanism‐of‐action. Prior work reported caylobolide B to be cytotoxic toward HT29 colorectal adenocarcinoma and HeLa cervical carcinoma cells (IC_50_ = 4.5 and 12.2 µM, respectively).^[^ ^1^ ^]^ We expanded the evaluation to HCT116 colorectal cancer cells and *Saccharomyces cerevisiae*, benchmarking against amantelides A and B. In HCT116 cells, *iso*‐caylobolide B is ∼2‐fold more potent than caylobolide B (IC_50_ = 0.2 µM versus 0.5 µM), whereas C29 monoacetates showed 24–65‐fold decrease in potency (Figure 2a). Similarly, modification at C33 of amantelide A (IC_50_ = 0.3 µM) gave a 17‐fold reduction in potency.^[^ ^4^ ^]^ In *S. cerevisiae*, *iso*‐caylobolide B displays a minimum inhibitory concentration (MIC) of 12.5 µM versus 25 µM for caylobolide B, and acetylated analogues are inactive (Figure 2b). These trends define a conserved pharmacophore, a hydroxyl group six carbons from the lactone oxygen (C33 in amantelide A; C29 in the caylobolides), with potency further tuned by C36 stereochemistry.

**Figure 2:**
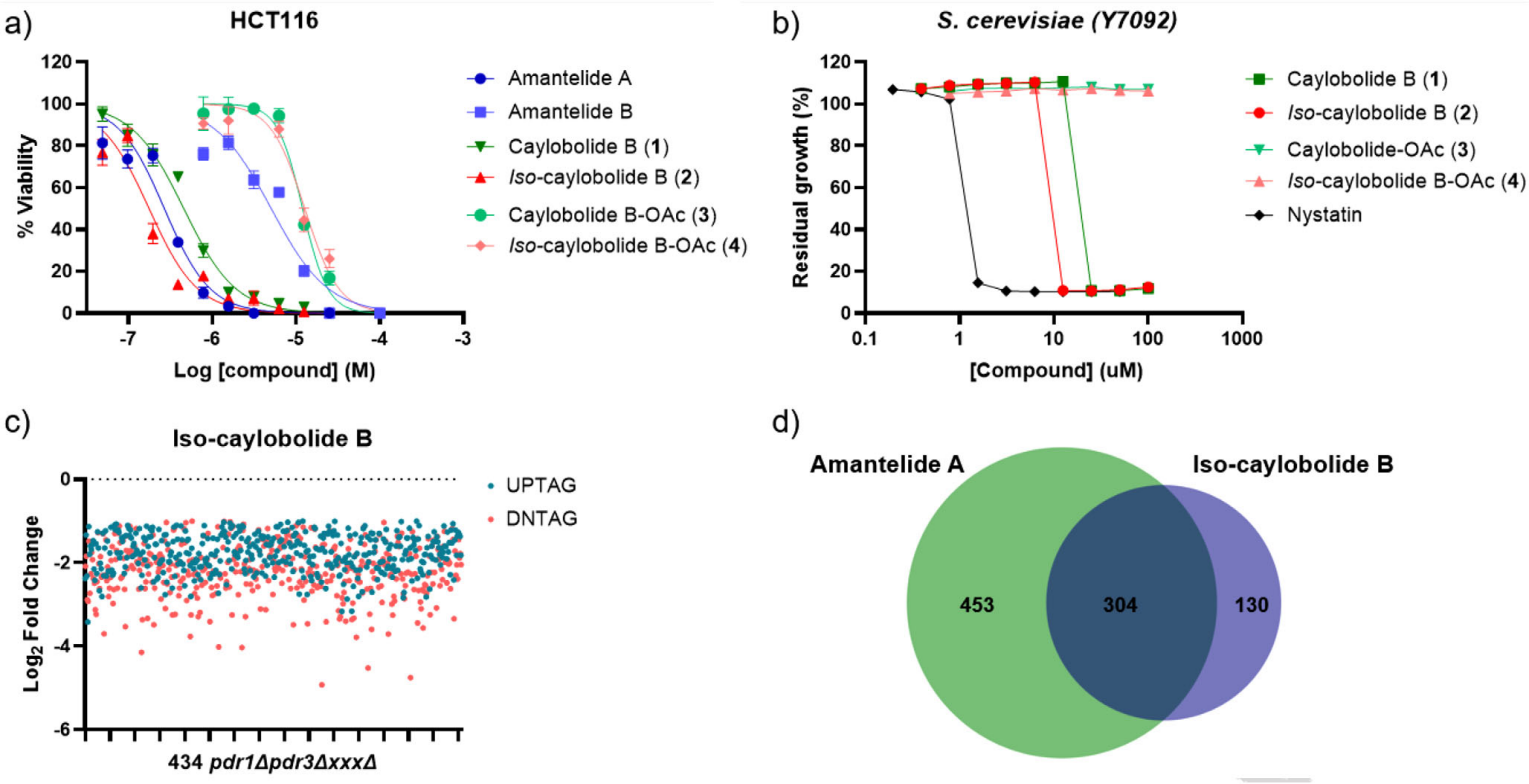
Bioactivity and chemogenomic profiling. a) Effects of the caylobolide B series and amantelides on HCT116 cell viability. Data are mean ±SD of three technical replicates (*n* = 3). IC_50_ values were obtained by four‐parameter nonlinear regression. b) Dose‐response curves of caylobolides against *S. cerevisiae* in YPD media. Error bars indicate ±1 SD from three technical replicates (*n* = 3). c) Scatter plot of hits sensitive to *iso‐*caylobolide B (fold change > 2; false discovery rate (FDR) ˂ 0.05). Chemogenomic profiling was performed using duplicate samples per condition (n = 2). d) Area‐proportional Venn diagram of yeast deletion strains sensitive to *iso*‐caylobolide B (this study) and amantelide A (fold change > 2; FDR ˂ 0.05).

Given that amantelide A targets sterol‐containing membranes and forms pores without strict ergosterol dependence,^[^ ^10^ ^]^ we investigated whether the caylobolides act similarly. We profiled *iso*‐caylobolide B at IC_20_ in *S. cerevisiae* using a barcoded library of ∼4300 homozygous deletion strains (each uniquely identified by UPtag/DNtag barcodes), following our established workflow.^[^ ^10^ ^]^ Of 3531 strains detected, 434 were significantly sensitized (>2‐fold change, FDR<0.05; Figure 2c). Because strains are completely deleted of nonessential genes, sensitization reflects pathways that buffer the compound‐target effects and are important for growth rather than direct molecular targets.^[^ ^18^ ^]^ Gene Ontology enrichment implicated the plasma membrane and cell periphery (Table S8). At a more stringent threshold (log_2_ fold change ≤ −3; DNtag hits, Table S9), deletions affecting actin‐cytoskeleton regulation were enriched, similar to what we observed in amantelide A and consistent with the actin cortex supporting membrane integrity.^[^ ^10^ ^]^ Comparative analysis demonstrated 70% of *iso*‐caylobolide B sensitized hits overlap with those of amantelide A (Figure 2d), and their enrichment again resulted in genes associated with cell periphery and plasma membrane (Table S10). The larger amantelide A set likely reflects its higher screening concentration (IC_25_ versus IC_20_). Together with the functional loss of the C29 monoacetates, these findings are consistent with a membrane‐targeting mechanism similar to amantelide A, that critically depends on the hydroxyl group six carbons from the lactone oxygen.^[^ ^10^ ^]^ The C29 hydroxyl acetylation likely hampers binding to sterol‐containing membranes, as shown with the amantelide A monoacetylated analogue.^[^ ^10^ ^]^ The shared polyhydroxylated macrolide architecture of bastimolide A/B and palstimolide A with the amantelides and caylobolides (Figure 1), which are reported to have potent antimalarial activity, suggests a related membrane‐targeting mode of action.^[^ ^2^, ^8^ ^]^ Differences in molecular lipid‐recognition could confer organism‐specific selectivity, though this remains to be experimentally validated.

Guided by these findings, we next resolved the full stereochemical configuration to connect structure with function. Structural characterization of polyhydroxylated macrolides is made challenging by the presence of repeated, identical spin systems, resulting in pronounced spectral degeneracy.^[^ ^15^ ^]^ To overcome this, ultra‐high‐resolution HSQC‐TOCSY (< 1 Hz digital resolution in F1), band‐selective HSQC, and HMBC spectra were acquired to enhance resolution in the F1 dimension.^[^ ^15^ ^]^ This enabled better separation of overlapped ^13^C signals in 2D spectra. In pyridine‐*d_5_*, ^3^ *J*_CH_ correlations from stereogenic C*H* atoms to central methylene (CH_2_) signals were used to assign the connectivity of all 1,n‐diol relationships, revealing that the originally proposed structure required revision, relocating the 1,3,5‐triol from C25–C29 to C17–C21 (Figure 3a). This revision mirrors our recent findings for the related macrolide, caylobolide A.^[^ ^15^ ^]^

**Figure 3:**
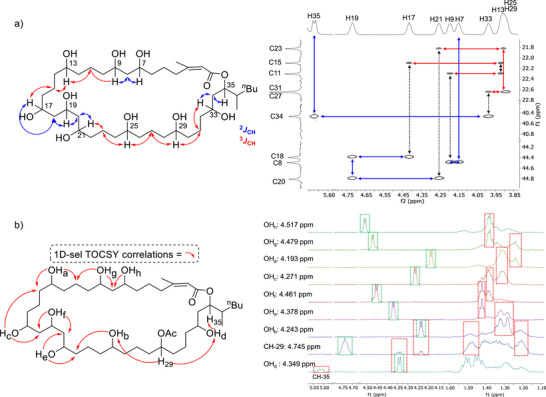
a) HSQC‐TOCSY correlations used for the skeletal reassignment of caylobolide B. Blue arrows are ^2^ *J*_CH_ (1,3‐diol) related and red are ^3^ *J*_CH_ related (1,5‐diol) signals in pyridine‐d_5_. b) Key 1D TOCSY correlations used to determine the skeletal structure of the 1,n‐polyol region of caylobolide B‐OAc. Dashed green boxes indicate the proton irradiated, and red boxes indicate the shared correlations used to determine 1,n‐polyol connectivity in DMSO‐d_6_.

In DMSO*‐d_6_* the C*H* signals of interest were partially obstructed by water in the sample which, due to the hygroscopic nature of the solvent, was not possible to remove. To identify 1,5‐polyol connectivity of caylobolide B‐OAc in DMSO‐*d_6_* a series of 1D‐selective TOCSY (total correlation spectroscopy) correlations were acquired, irradiating each of the polyol O*H* (a‐f) and C*H*‐29 signals to deconvolute highly overlapped methylene (CH_2_) signals. Multiplet pattern matching between the separate 1D‐selective TOCSY spectra enabled the identification of the 1,n‐polyol order (Figure 3b). Full chemical shift assignments for caylobolide B and *iso*‐caylobolide B confirmed that the compounds express a shared carbon skeleton, indicating that *iso*‐caylobolide B is a naturally occurring diastereomer of caylobolide B.

Following this, our simple rule for the relative configurational analysis of 1,5‐diols was applied to caylobolide B, where *syn* 1,5‐diols possess large Δ*δ*_HaHb_ values (≥0.17 ppm), whereas *anti* 1,5‐diols possess small Δ*δ*_HaHb_ values (≤0.13 ppm) (Figure 4a).^[^ ^16^ ^]^ Kishi's universal NMR database was utilized towards the relative configurational analysis of the 1,3‐diol and triols and indicated a *syn* relationship for C7‐C9 and an *anti*‐*syn* relationship for C17–21.^[^ ^17^ ^]^ Analysis of the polyol region was consistent for both caylobolide B isomers, and was also consistent with that of the C9–C33 fragment of caylobolide A.^[^ ^15^ ^]^ As a result, absolute stereochemistry was assigned by analogy to caylobolide A, as enantiomeric natural products of this complexity are exceedingly rare.^[^ ^19^ ^]^ ROESY (rotating frame overhauser effect spectroscopy) correlation between H42 and H2 supported the previously proposed (*Z*)‐configuration of the α,β‐unsaturated ester moiety.^[^ ^7^ ^]^ To pinpoint the site of stereochemical variation, we compared the chemical shifts of the caylobolide B isomers (Figure 4b). The largest differences were observed near C35 and C36, indicating that the stereochemistry at one of these positions was inverted. Through comparison of the chemical shift differences (Δ*δ* ^13^C) of *iso*‐caylobolide B and caylobolide B, to caylobolide A and a previously synthesized C36 epimer, a strong correlation between the Δ*δ* ^13^C values was observed (Figure 4c). We therefore concluded that the stereochemical difference between the caylobolide B isomers would be most likely located at C36.

**Figure 4:**
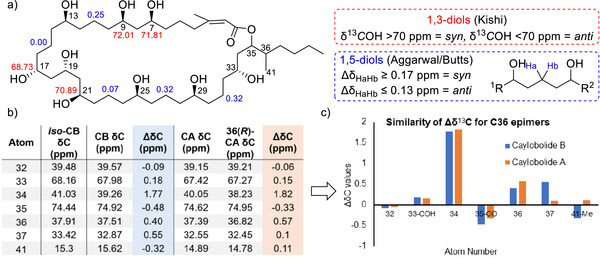
a) Overview for the relative stereochemical analysis of 1,3‐ and 1,5‐diol moieties of caylobolide B using Kishi's universal NMR database, our Δ*δ*_HaHb_ analysis, and through structural analogy to caylobolide A. b) ^13^C chemical shift differences between: *iso*‐caylobolide B and caylobolide B (blue), caylobolide A and 36(R)‐caylobolide A (orange). Only significant differences are displayed. For full comparison see Table S4. c) Bar chart demonstrating the similarities between C36 epimers of caylobolide B (blue) and caylobolide A (orange).

With absolute configurations assigned and a likely site of epimeric variation at C36, we designed a modular, late‐stage stereodivergent synthetic route to access both C‐36 diastereomers. Retrosynthetic disconnection of the macrocycle was proposed through a one‐pot hydroboration‐Suzuki cross coupling, a strategy previously utilized towards bastimolide B (Scheme 1).^[^ ^9^ ^]^ Ester formation could then be used to install the (*Z*)‐iodoacrylate moiety. Further retrosynthetic disconnection of key C─C bonds utilizing boronic ester homologation reactions ultimately provides a five‐fragment strategy, where four of the five building blocks (fragments 2–5) were used in the synthesis of bastimolide B^[^ ^9^ ^]^ and caylobolide A,^[^ ^15^ ^]^ exemplifying the modularity of such polyketide syntheses. Key to the ability to access both natural product diastereomers simultaneously was the design of a route that would allow the late‐stage incorporation of C36, using a building block that could be easily accessed as either enantiomer (fragment 4).

**Scheme 1:**
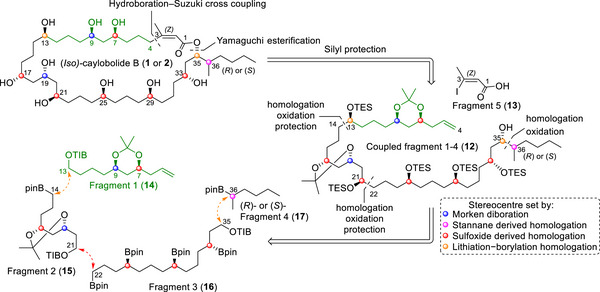
Retrosynthetic analysis of (*iso*‐)caylobolide B (**1** and **2**) to fragments 1–5 where fragments 1–4 were unified by metalation‐borylation strategies. Fragment 1 (**14**, highlighted) was unique to caylobolide B, and structures in black are in common with caylobolide A (fragment 2 (**15**), fragment 3 (**16**), fragment 4 (**17**)), and bastimolide B (fragment 2 (**15**) fragment 5 (**13**)).

The synthesis of fragment 1 (**14**) was achieved in a five‐step sequence (Scheme 2). Enantioselective platinum‐catalyzed diboration of the terminal alkene of **18** was performed to obtain bis‐boronic ester **19** in high e.r. and excellent yield.^[^ ^20^ ^]^ Regioselective homologation of the primary boronic ester moiety of **19** with the magnesiated carbenoid of sulfoxide **20** (generated through sulfoxide‐magnesium exchange), followed by oxidation of the resulting 1,3‐bisboronic ester afforded 1,3‐diol **21** in a good yield over two steps. Acetonide protection of **21** afforded enantio‐ and diastereomerically pure **14**, upon removal of the minor diastereomer by preparative HPLC. Fragments 2–5 were prepared according to the previous syntheses of caylobolide A and bastimolide B.^[^ ^9^, ^15^ ^]^

**Scheme 2:**
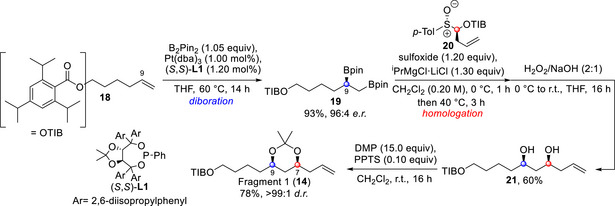
Synthetic route to access fragment 1 (**14**) of caylobolide B. DMP, 2,2‐dimethoxypropane; dba, dibenzylideneacetone; PPTS, and pyridinium *p*‐toluenesulfonate.

Fragment coupling towards caylobolide B began with lithiation of fragment 1 (**14**) at C13 by (+)‐sparteine‐ligated s‐BuLi, with subsequent trapping by the boronic ester group of fragment 2 (**15**) (Scheme 3). The resulting secondary boronic ester was oxidized by basic peroxide and TES protected to avoid unwanted side reactions in further homologations. C21 of the TES protected product was lithiated with (+)‐sparteine‐ligated s‐BuLi, transmetalated with MgBr_2_•Et_2_O, and trapped with (+)‐Andersen's sulfinate to afford sulfoxide **22** in 64% yield over four steps in excellent d.r. with respect to C13. The minor diastereomer generated at C21 was removed through chromatographic separation. Chemoselective homologation of the primary boronic ester of fragment 3 (**16**) was performed with the magnesiated carbenoid of **22**. Oxidation of the resulting tetraboronic ester, and subsequent TES protection of the tetraol, yielded **23** with full stereocontrol in a moderate yield over three steps.

**Scheme 3:**
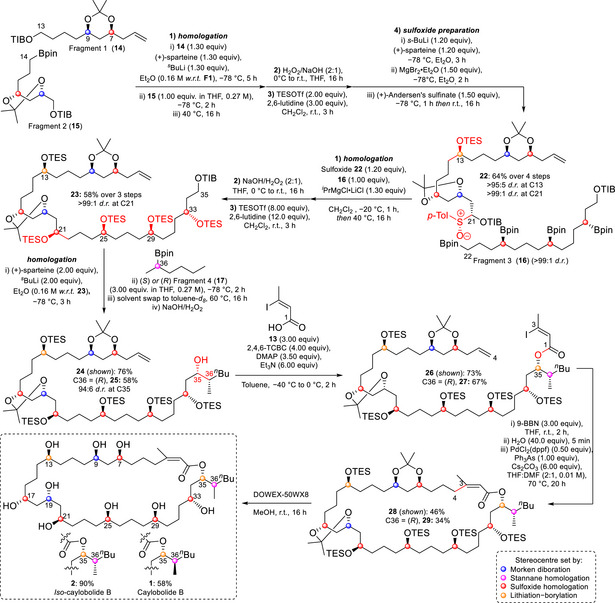
Total synthesis of caylobolide B and *iso*‐caylobolide B accessed simultaneously through late stage stereodivergence.

Fragment **23** was lithiated with (+)‐sparteine‐ligated *s*‐BuLi to set the C35 stereochemistry, then coupled in separate reactions with (*S*)‐configured and (*R*)‐configured fragment 4 (**17**). The resulting secondary boronic esters were oxidized to afford the two epimeric alcohol products **24** and **25** in moderate to good yield, varying in configuration at C36. The alcohols were engaged in a modified Yamaguchi esterification with (*Z*)‐iodoacrylate **13** to afford **26** and **27**.^[^ ^9^, ^21^ ^]^ The terminal alkene of each epimer was hydroborated with 9‐BBN before the crude product was directly engaged in ring‐closing Suzuki cross‐coupling with the (*Z*)‐iodoacrylate moiety to form **28** and **29** in moderate yields. Final global deprotection was performed with DOWEX‐50W‐X8 sulfonic acid bound resin to afford epimers **1** and **2**. Comparison of the two diastereomeric products revealed that the (36*S*) isomer (**2**), which was stereochemically analogous to caylobolide A, matched all NMR spectroscopic data for the newly isolated *iso*‐caylobolide B. Gratifyingly, the (36*R*) isomer (**1**) matched the originally isolated caylobolide B, thereby establishing the absolute configuration of all caylobolides and completing their first total syntheses.

In summary, we present a comprehensive biological characterization of caylobolide B, its C36 epimer *iso*‐caylobolide B, and their C29 acetylated analogues. Our results show that subtle changes in stereochemistry and structure can dramatically alter both cytotoxic and antifungal activities, with the C29 hydroxyl group emerging as critical for bioactivity. Unbiased chemogenomic profiling suggests that *iso*‐caylobolide B disrupts membrane integrity with an overlapping mechanism to amantelide A. By applying ultra‐high‐resolution NMR spectroscopy to the bioactive isolates, we achieved structural revision and biological profiling simultaneously, ensuring that all activity data correspond to fully validated structures. A modular stereodivergent synthesis was then completed to validate the structures, confirm absolute configuration and demonstrate the potential to access non‐natural analogues. Together, these results resolve a longstanding structural ambiguity and establish a robust modular route for synthesis and reveal new mechanistic insights into the bioactivity of marine macrolides. This integrated workflow opens new avenues for rational design and discovery of macrolide‐based therapeutics with improved potency and selectivity.

## Supporting Information

The authors have cited additional references within the Supporting Information.^[^ ^22^, ^23^, ^24^, ^25^, ^26^, ^27^, ^28^, ^29^, ^30^, ^31^, ^32^ ^]^

## Conflict of Interests

The authors declare no conflict of interest.

## Supporting information



Supporting Information

## Data Availability

The data that support the findings of this study are available in the Supporting Information of this article.
