# Supplementary material for: Caylobolide B: Structure Revision, Total Synthesis, Biological Characterization, and Discovery of New Analogues
Source: Angew Chem Int Ed Engl. 2025 Dec 10;65(4):e23117. doi: 10.1002/anie.202523117 (PMC12828451; doi:10.1002/anie.202523117)

Supporting Information

Caylobolide B: Structure Revision, Total Synthesis, Biological Characterization and Discovery of New Analogues

Malcolm R. P. George +[a], Lobna A. Elsadek +[b], Max Deering [a], Larissa Costa de Almeida [b], Jasper L. Tyler [a], Adam Noble [a], Valerie J. Paul [c], Hendrik Luesch *[b,d], Craig P. Butts *[a], Varinder K. Aggarwal *[a]

[a]School of Chemistry, University of Bristol, Cantock's Close, Bristol BS8 1TS, UK.

*Email: v.aggarwal@bristol.ac.uk

*Email: craig.butts@bristol.ac.uk

[b]Medicinal Chemistry, University of Florida, 1345 Center Dr., Gainesville, Room P3-12, FL 32603, USA

*Email: luesch@cop.ufl.edu

[c]Smithsonian Marine Station 701 Seaway Drive, Fort Pierce, FL 34949, USA

[d]Program in Cancer and Stem Cell Biology, Duke-NUS Medical School, Singapore, 169857

+ These authors contributed equally to this work.

Table of Contents

1 GENERAL INFORMATION 4

2 MATERIALS AND REAGENTS 5

3 ISOLATION AND STRUCTURAL ANALYSIS OF CAYLOBOLIDE B ANALOGUES 6

3.1 Isolation of Caylobolide B Analogues 6

3.2 Caylobolide B and *Iso*-caylobolide B NMR Analysis (DMSO-d_6) 8

3.3 *Iso*-caylobolide B NMR Analysis 8

3.3.1 ^{1}H NMR (700 MHz, pyridine-d_5) 9

3.3.2 ^{13}C NMR (151 MHz, pyridine-d_5) 10

3.3.3 HMBC NMR (pyridine-d_5, 700 MHz) 11

3.3.4 Multiplicity Edited Pure Shift HSQC NMR 12

3.3.5 HSQC-TOCSY NMR (pyridine-d_5, 700 MHz) 16

3.3.6 ^{1}H NMR (DMSO-d6, 600 MHz) 20

3.3.7 HSQC NMR (DMSO-d6, 600 MHz) 21

3.3.8 COSY NMR (DMSO-d6, 600 MHz) 22

3.3.9 HMBC NMR (DMSO-d6, 600 MHz) 23

3.3.10 HSQC-TOCSY NMR (DMSO-d6, 600 MHz) 24

3.4 Caylobolide B NMR Analysis 26

3.4.1 ^{1}H NMR (700 MHz, pyridine-d_5) 26

3.4.2 ^{13}C NMR (151 MHz, pyridine-d_5) 27

3.4.3 HMBC NMR (pyridine-d_5, 700 MHz) 28

3.4.4 Multiplicity Edited Pure Shift HSQC NMR 29

3.4.5 HSQC-TOCSY NMR (pyridine-d_5, 700 MHz) 33

3.4.6 ^{1}H NMR (DMSO-d6, 600 MHz) 39

3.4.7 HSQC NMR (DMSO-d6, 600 MHz) 40

3.4.8 COSY NMR (DMSO-d6, 600 MHz) 41

3.4.9 HMBC NMR (DMSO-d6, 600 MHz) 42

3.5 *Iso*-caylobolide B-OAc NMR Analysis 44

3.5.1 ¹H NMR (DMSO-d6, 600 MHz) 44
3.5.2 HSQC NMR (DMSO-d6, 600 MHz) 45
3.5.3 COSY NMR (DMSO-d6, 600 MHz) 49
3.5.4 HMBC NMR (DMSO-d6, 600 MHz) 50
3.5.5 HSQC-TOCSY NMR (DMSO-d6, 600 MHz) 53
3.6 Caylobolide B-OAc NMR Analysis 55
3.6.1 ¹H NMR (DMSO-d6, 600 MHz) 55
3.6.2 HSQC NMR (DMSO-d6, 600 MHz) 56
3.6.3 COSY NMR (DMSO-d6, 600 MHz) 60
3.6.4 HMBC NMR (DMSO-d6, 600 MHz) 61
3.6.5 HSQC-TOCSY NMR (DMSO-d6, 600 MHz) 63
3.4 Bioactivity and SAR of Caylobolide B Analogues 65
3.4.1 MTT Cell Viability Assay 65
3.4.2 S. Cerevisiae Growth Inhibition Assay 65
3.4.3 Chemogenomic Homologous Deletion Profiling (HOP) Assay 65
3.4.4 Bioinformatic analysis of HOP assay 66
3.4.5 Gene Ontology Term Analysis 67
4 SYNTHETIC PROCEDURES 69
General Procedure 1 (GP1) – α-Sulfinylbenzoate Preparation 69
General Procedure 2 (GP2) – Sulfoxide Homologation 70
General Procedure 3 (GP3) – Hydroboration 70
General Procedure 4 (GP4) – Boronic Ester Oxidation 71
General Procedure 5 (GP5) – TES Protection of Alcohols 71
General Procedure 6 (GP6) – Yamaguchi Esterification 71
General Procedure 7 (GP7) – One-pot Hydroboration-Suzuki Cross Coupling 72
5 PREPARATION AND CHARACTERISATION DATA 72
Fragment 1 (14) 75
Fragment 2 (15) 80
Fragment 3 (16) 85
Fragment 4 (17) 96
Caylobolide B fragment coupling 101
Iso-caylobolide B (2) final steps 110
Caylobolide B (1) final steps 118
5 REFERENCES 126

6 SPECTRAL APPENDIX..127

1 GENERAL INFORMATION

Solvents, Reagents, Glassware and Reaction Setup: unless otherwise stated, all reactions were conducted under an inert atmosphere of nitrogen in flame dried glassware using standard Schlenk techniques. Air- and moisture-sensitive liquids and solutions were transferred via syringe into the reaction vessels through a rubber septum. Unless otherwise specified, all reagents were purchased at highest commercial quality and used as received. Non-anhydrous solvents were purchased (unless specified) at the highest commercial quality and used as received. CH_2Cl_2, Et_2O and THF were dried on an Anhydrous Engineering alumina column drying system. Temperatures described below −10 °C were achieved using Thermo Scientific EK-90 or Huber TC100E cryostats or appropriate solvent/dry ice baths.

Chromatography: flash column chromatography was carried out using Sigma-Aldrich silica gel (60 Å, 230-400 mesh, 40-63 µm) or a Biotage Isolera One automated flash purification system, as indicated. Reactions were followed by thin-layer chromatography (TLC) where practical, using aluminium-backed Merck Kieselgel 60 F254 fluorescent treated silica gel plates, which were visualised under UV light or by staining with aqueous basic $KMnO_4$, acidic *p*-anisaldehyde solution in ethanol, or phosphomolybdic acid solution in ethanol.

NMR: high-field NMR spectra were collected on either a 9.4 T Jeol ECZ spectrometer fitted with a 5 mm 40ROHFXS probe (400 MHz), 9.4 T Jeol ECS spectrometer fitted with a 5 mm 40RO5AT probe (400 MHz), 9.4 T Bruker Nano spectrometer fitted with a 5 mm BBFO probe (400 MHz), 11.75 T Bruker Avance III HD spectrometer fitted with a 5 mm DCH 500S1 cryoprobe (500 MHz), 14.1 T Bruker Neo spectrometer fitted with a 5 mm TXO 600S3 cryoprobe (600 MHz), 16.44 T Bruker AVANCE III HD spectrometer fitted with a 1.7-mm TCI triple resonance micro cryoprobe (700 MHz), 14.1 T Bruker Avance Neo spectrometer fitted with a 5 mm BBO 600S3 cryoprobe (600 MHz) or a 14.1 T Agilent VNMRS-600 MHz, spectrometer fitted with a 5-mm cold probe. All NMR experiments were collected at 1 atm and 298 K according to the respective spectrometer console. Chemical shifts (δ) are quoted in parts per million (ppm) and referenced to the appropriate NMR solvent peak(s) and are assigned in accordance with numbered diagrams; with resonances

described as s (singlet), d (doublet), t (triplet), q (quartet), p (pentet), combinations thereof (i.e. td indicates a triplet of doublets) or m (multiplet) and br. s (broad singlet). Numbering of C-atoms for NMR assignment follows the numbering given to Caylobolide B in the original paper.[1]

HRMS (high resolution mass spectra) were recorded on a Bruker Daltonics MicrOTOF II by Electrospray Ionisation (ESI); a Thermo Scientific QExactive by Electron Ionisation (EI); a Thermo Scientific Orbitrap Elite by ESI or Atmospheric Pressure Chemical Ionisation (APCI); a Bruker UltrafleXtreme by Matrix-assisted Laser Desorption/Ionisation (MALDI); or a Agilent LC-TOF mass spectrometer equipped with APCI/ESI multimode ion source-detector in positive mode. Only molecular ions ($[M+H]^+$, $[M+NH_4]^+$ or $[M+Na]^+$) are reported.

HPLC: enantiomeric ratios were determined by HPLC analysis on chiral stationary phase performed using Daicel Chiralpak IA/IB columns (4.6 mm×250 mm×5 μm)/OD column (4.6 mm×250 mm×10 μm)/AD-H (4.6 mm×250 mm×5 μm) column on an Agilent system and monitored using a diode array detector (DAD).

IR spectra were recorded on neat compounds using a Perkin Elmer (Spectrum One) FT-IR spectrometer (ATR sampling accessory). Selected absorbances (ν_{max}, expressed in cm^{-1}) are reported.

Melting points (m.p.) were recorded in degrees Celsius (°C) using a Stuart SMP30 melting point apparatus.

Optical rotations ($[\alpha]_D^{25}$) were measured on a Bellingham & Stanley Ltd. ADP 220 polarimeter or a Perkin-Elmer 341 polarimeter.

Naming of compounds: Compound names are generated by ChemDraw Professional 20.0 software (PerkinElmer), following the IUPAC nomenclature.

2 MATERIALS AND REAGENTS

$Pt(dba)_3$ [CAS 11072-92-7] was purchased from Strem Chemicals, Inc. and used as received. Morken's diboration ligand (*S*,*S*)-3,5-di-*iso*-propylphenylTADDOLPPh [(*S*,*S*)-**L1**], was prepared in-house according to the published procedure.[2] (+)-Andersen's sulfinate [CAS 91796-57-5] was purchased from Sigma Aldrich and recrystallised from Et_2O before use. (−)-Andersen's sulfinate [CAS 1517-82-4] was purchased from Henan Tianfu Chemical Co., Ltd

and recrystallised from Et_2O before use. Pt(dppf)Cl_2 [CAS 72287-26-4] was purchased from Fluorochem and used as received. Triphenylarsine [CAS 603-32-7] was purchased from Sigma Aldrich and used as received. Trimethyltin chloride [CAS 1066-45-1] was purchased from Sigma Aldrich as a 1.0 M solution in THF and used as received. 2,6-lutidine [CAS 108-48-5] was purchased from Sigma Aldrich and used as received. Bis(1,5-cyclooctadiene)diiridium(I) dichloride [CAS 12112-67-3] was purchased from Sigma Aldrich and used as received.

n-Butyllithium [CAS 109-72-8] was purchased from Acros as a 1.6 M solution in *n*-hexane. *s*-Butyllithium [CAS 598-30-1] was purchased from Acros Organics as a 1.3 M solution in cyclohexane:*n*-hexane 98:2. The molarity of organolithium solutions was determined by titration with *N*-benzylbenzamide.[3] *i*-PrMgCl·LiCl was purchased from Sigma Aldrich as a 1.2 M solution in THF and the molarity was verified by titration with iodine.[4]

TMEDA, Et_3N, TESOTf and TMSCl were distilled over CaH_2 before use. Pinacolborane and 2,4,6-trichlorobenzoyl chloride were distilled before use. (–)-Sparteine was isolated from the commercially available sulfate pentahydrate salt following a procedure by Beak.[5] (+)-Sparteine was purchased as the free base and distilled over CaH_2. The sparteine free base readily absorbs atmospheric carbon dioxide (CO_2) and so should be stored under argon/nitrogen at −20 °C in a sealed Schlenk tube. Sparteine can be recovered reliably during work-up with aqueous HCl as reported in the literature.[6]

All other reagents were purchased from various commercial sources and used as received.

3 ISOLATION AND STRUCTURAL ANALYSIS OF CAYLOBOLIDE B ANALOGUES

3.1 Isolation of Caylobolide B Analogues

Brown wavy samples of *Okeania* sp. (VPG 16-72) were collected from Tumon Bay, Guam on 12/8/2016 by Valerie Paul and coworkers.

The freeze-dried sample was subjected to non-polar extraction with 1:1 EtOAc–MeOH and polar extraction with 1:1 EtOH–H_2O. The non-polar extract was subsequently partitioned between hexane and MeOH:H_2O (9:1). The MeOH:H_2O fraction was further partitioned between EtOAc and H_2O. The EtOAc was fractionated using silica column chromatography applying a gradient of increasing polarity (DCM, 90% DCM/iPrOH, 70%

DCM/iPrOH, 50% DCM/i-PrOH, 20% DCM/i-PrOH, iPrOH, 90% DCM/MeOH, 70% DCM/MeOH, MeOH). The fraction eluting with 50% DCM/iPrOH was purified using reversed-phase HPLC [SynergiHydro, 250 × 10.0 mm; flow rate, 4.0 mL/min; PDA detection 200–800 nm] using a linear MeCN–H_2O gradient (30–100% MeCN over 21 min) to afford fraction 2 (t_R 19.7 min), containing mixture of **3** and **4**. Fraction 2 was purified using was purified using reversed-phase HPLC [Luna C18, 250 × 10.0 mm; flow rate, 4.0 mL/min; PDA detection 200–800 nm] using 75% MeOH/H_2O to afford **3** (0.5 mg, t_R 19.2 min) and **4** (4.6 mg, t_R 18.4 min). The acetylated analogs were present in the EtOAc partitioned fraction; therefore it was fractionated using silica column chromatography applying a gradient of increasing polarity (DCM, 90% DCM/iPrOH, 70% DCM/iPrOH, 50% DCM/i-PrOH, 20% DCM/i-PrOH , iPrOH, 90% DCM/MeOH, 70% DCM/MeOH, MeOH). The fraction eluting with 50% DCM/iPrOH was purified using reversed-phase [SynergiHydro, 250 × 10.0 mm; flow rate, 4.0 mL/min; PDA detection 200–800 nm] using a linear MeCN–H_2O gradient (40–90% MeCN over 31 min) to afford fraction 9 (t_R 17.7 min), containing a mixture of **1** and **2**. Fraction 9 was purified using reversed-phase HPLC [Luna C18, 250 × 10.0 mm; flow rate, 4.0 mL/min; PDA detection 200–800 nm] using 75% MeOH/H_2O to afford **1** (2 mg, t_R 32 min) and **2** (6 mg, t_R 29.5 min).

Caylobolide B (**1**): white amorphous solid; $[\alpha]^{25}_D$ −21 (*c* 0.024, MeOH); NMR data, ^1H NMR, COSY, HSQC, HMBC, in DMSO-d_6 and pyridine-d_5 *see Table S3 and S4*; HRESIMS *m/z:* 761.5748 $[M + H]^+$ (calcd for $C_{42}H_{81}O_{11}$, 761.5778)

Iso-caylobolide B (**2**): white amorphous solid; $[\alpha]^{26}_D$ −21 (*c* 0.36, MeOH); NMR data, ^1H NMR, COSY, HSQC, HMBC, in DMSO-d_6, and pyridine-d_5 *see Table S1 and S2*; HRESIMS *m/z:* 761.5751 $[M + H]^+$ (calcd for $C_{42}H_{81}O_{11}$, 761.5778)

Caylobolide B-OAc (**3**): colourless amorphous solid; $[\alpha]^{25}_{D}$ −14 (*c* 0.22, MeOH); NMR data, ^{1}H NMR, COSY, HSQC, HMBC, in DMSO-d_6 and pyridine-d_5 *see Table S6*; HRESIMS *m/z:* 803.5868 $[M + H]^+$ (calcd for $C_{44}H_{83}O_{12}$, 803.5884)

Iso-caylobolide B-OAc (**4**): colourless amorphous solid; $[\alpha]^{25}_{D}$ −19 (*c* 0.28, MeOH); NMR data, ^{1}H NMR, COSY, HSQC, HMBC, in DMSO-d_6 and pyridine-d_5 *see Table S5*; HRESIMS *m/z:* 803.5856 $[M + H]^+$ (calcd for $C_{44}H_{83}O_{12}$, 803.5884)

3.2 Caylobolide B and *Iso*-caylobolide B NMR Analysis (DMSO-d_6)

3.3 *Iso*-caylobolide B NMR Analysis

For the full structural assignment of caylobolide B and *iso*-caylobolide B, 1.0 mg of each isolated natural product was dissolved in 40 μL of pyridine-d_5 and added to a 1.7 mm NMR tube. The sample was subjected to an array of experiments to uniquely assign all ^{13}C and ^{1}H chemical shifts. A key experiment for the full structural assignment of such highly convoluted spectra is the HSQC-TOCSY (Heteronuclear Single Quantum Coherence-TOtal Correlation SpectroscopY) experiment. This was previously applied by our group to the full skeletal reassignment of synthetic bastimolide A and caylobolide A.[7],[8] The HSQC-TOCSY experiment is a 2D hybrid inverse gated experiment consisting of a HSQC pulse train, followed by a TOCSY mixing period. This provides a 2D spectrum which shows $^{n}J_{CH}$ correlations for a given spin system, where n is highly dependent upon the duration of the TOCSY mixing period (D9 in the pulse program parameters). For the full chemical shift assignment of caylobolide B, two HSQC-TOCSY spectra were acquired at mixing times of 30 ms and 60 ms. The spectra recorded with a 30 ms mixing time showed $^{1\text{-}3}J_{CH}$ correlations, which were used to assign the chemical shifts of the stereogenic carbon atoms, and their respective protons. The spectra recorded with a 60 ms mixing time showed $^{1\text{-}4}J_{CH}$ correlations, which were used to assign the chemical shifts of carbon atoms adjacent to stereocenters.

3.3.1 ^{1}H NMR (700 MHz, pyridine-d_5)

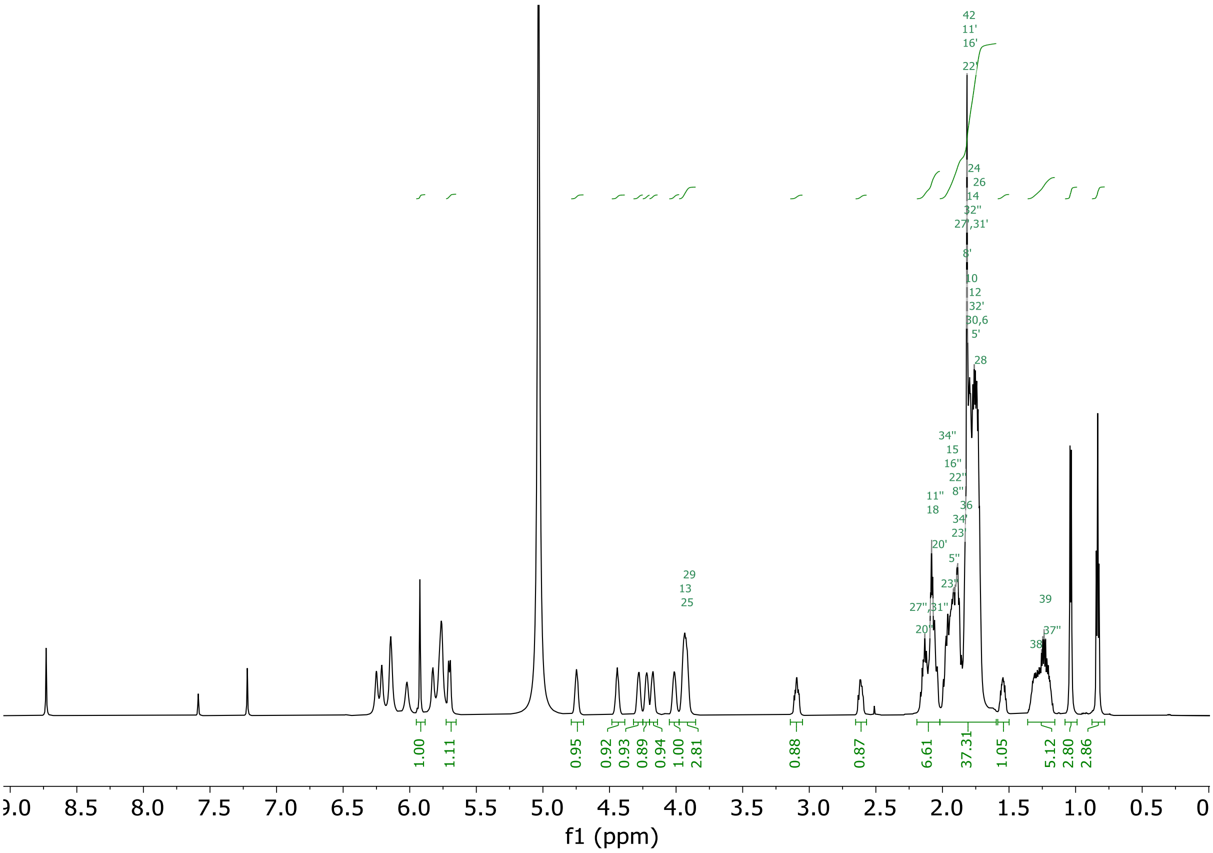

Figure S1: ^{1}H NMR spectrum (700 MHz, pyridine-d_5) of *iso*-caylobolide B (**2**).

3.3.2 ^{13}C NMR (151 MHz, pyridine-d_5)

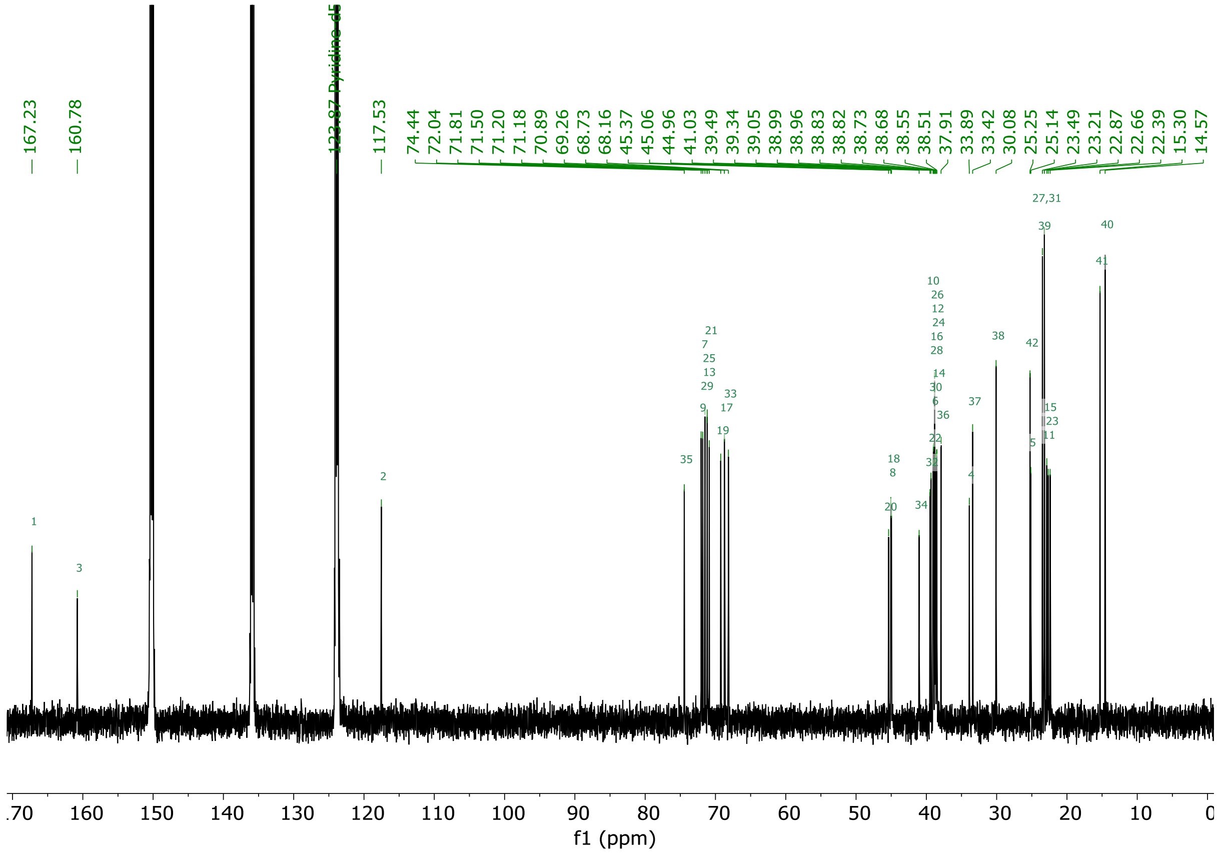

Figure S2: ^{13}C NMR spectrum (151 MHz, pyridine-d_5) of *iso*-caylobolide B (**2**).

3.3.3 HMBC NMR (pyridine-d_5, 700 MHz)

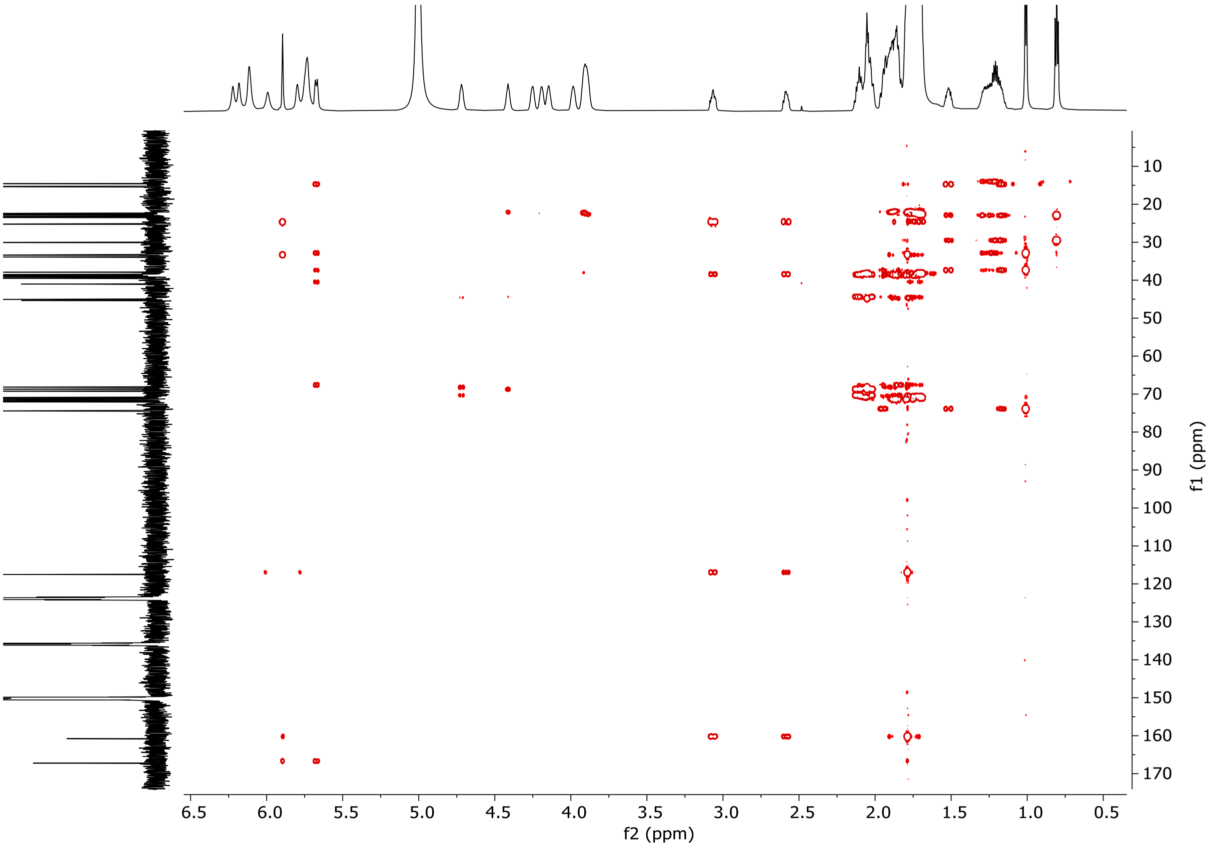

Figure S3: HMBC spectrum (700 MHz, pyridine-d_5) of *iso*-caylobolide B (**2**).

3.3.4 Multiplicity Edited Pure Shift HSQC NMR

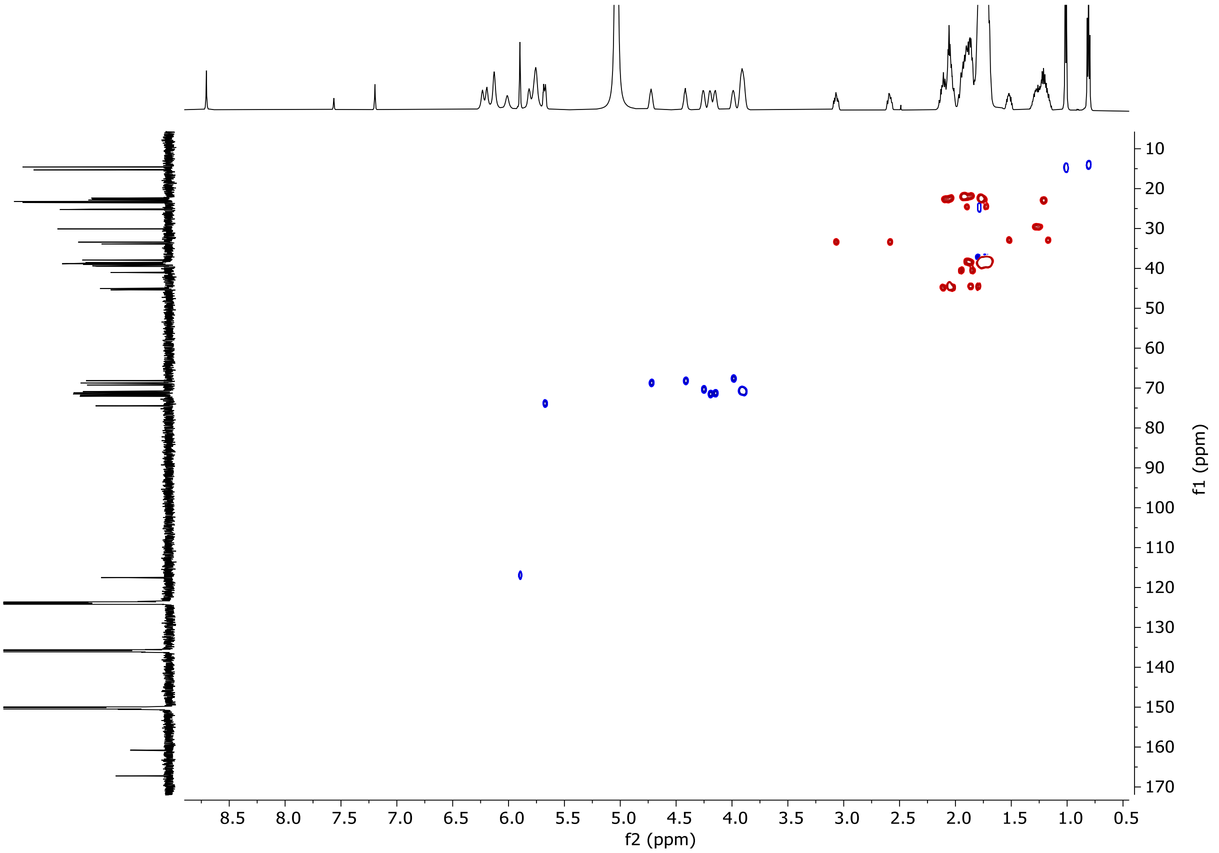

Figure S4: Full HSQC spectrum (700 MHz, pyridine-d_5) of *iso*-caylobolide B (**2**).

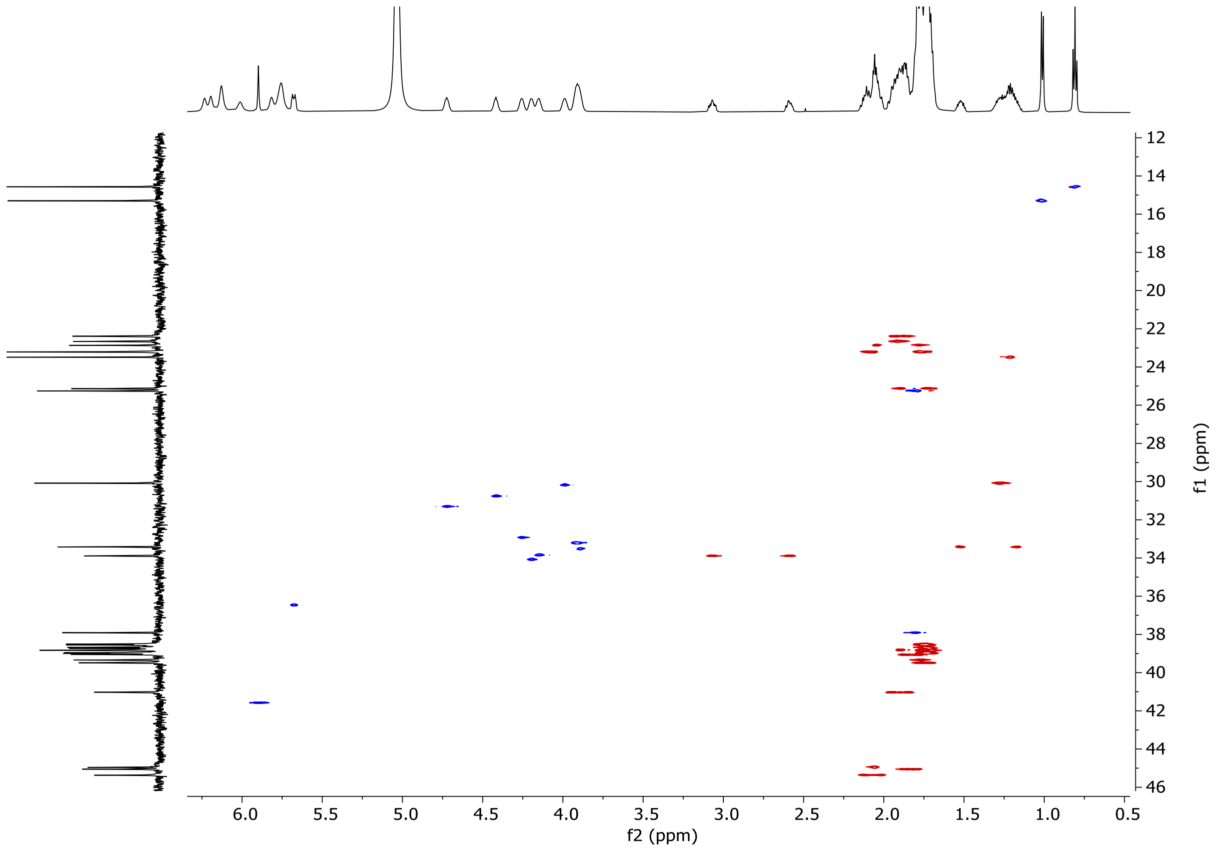

Figure S5: Ultra-high-resolution pure shift HSQC of *iso*-caylobolide B (**2**) in pyridine-d_5 (Phase-sensitive ge-2D multiplicity-edited HSQC using echo-antiecho and inversion and matched sweep adiabatic pulses with broadband homodecoupling: hsqcedetgpsp.3_bbhd). Spectrometer frequency (F2: 700.10 MHz, F1: 176.05 MHz), acquired size (t2: 597, t1: 1024), spectral size (t2: 1024, t1: 8192), spectral width (F2: 7352.9 Hz, F1: 5584.5 Hz), 2 scans. Spectrum centred at the aliphatic region where CHO correlations are folded back into the lower ppm region (correlations between 3-6 ppm in ^{1}H).

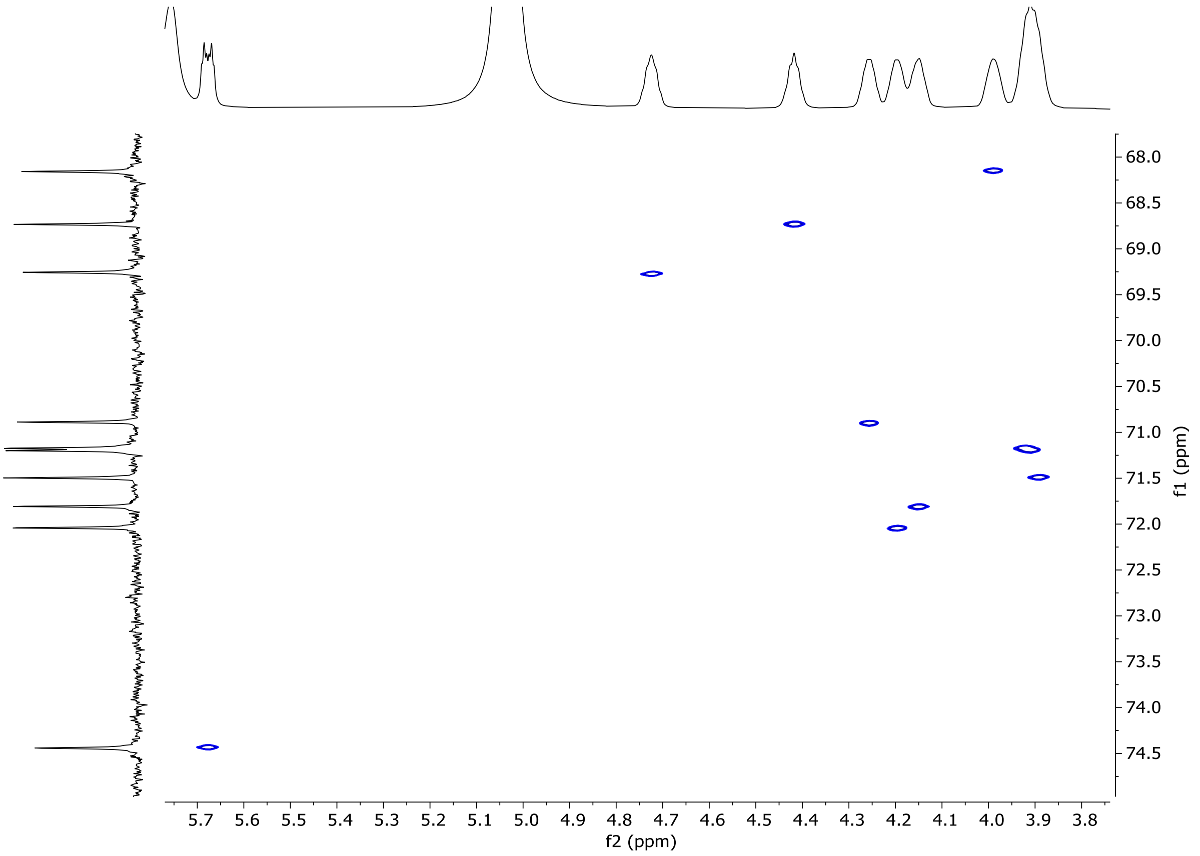

Figure S6: Zoomed in region (^{13}C: 67-75 ppm, ^{1}H: 3.8-5.8 ppm) of ultra-high-resolution pure shift HSQC of *iso*-caylobolide B (**2**) in pyridine-d_5. Spectra recentred to align with CHO correlations.

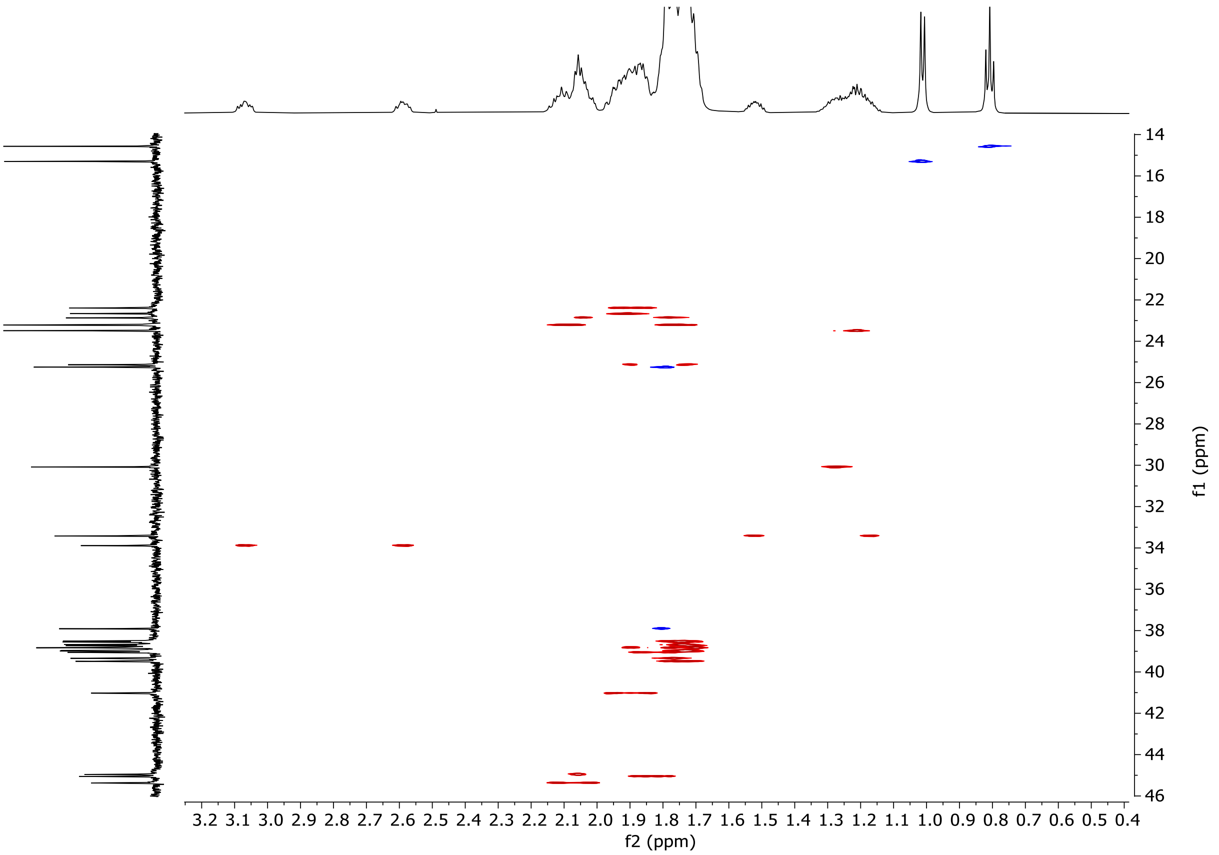

Figure S7: Zoomed in region (^{13}C: 14-46 ppm, ^{1}H: 0.4-3.2 ppm) of ultra-high-resolution pure shift HSQC of *iso*-caylobolide B (**2**) in pyridine-d_5. Spectra recentred to align with alkyl region.

3.3.5 HSQC-TOCSY NMR (pyridine-d_5, 700 MHz)

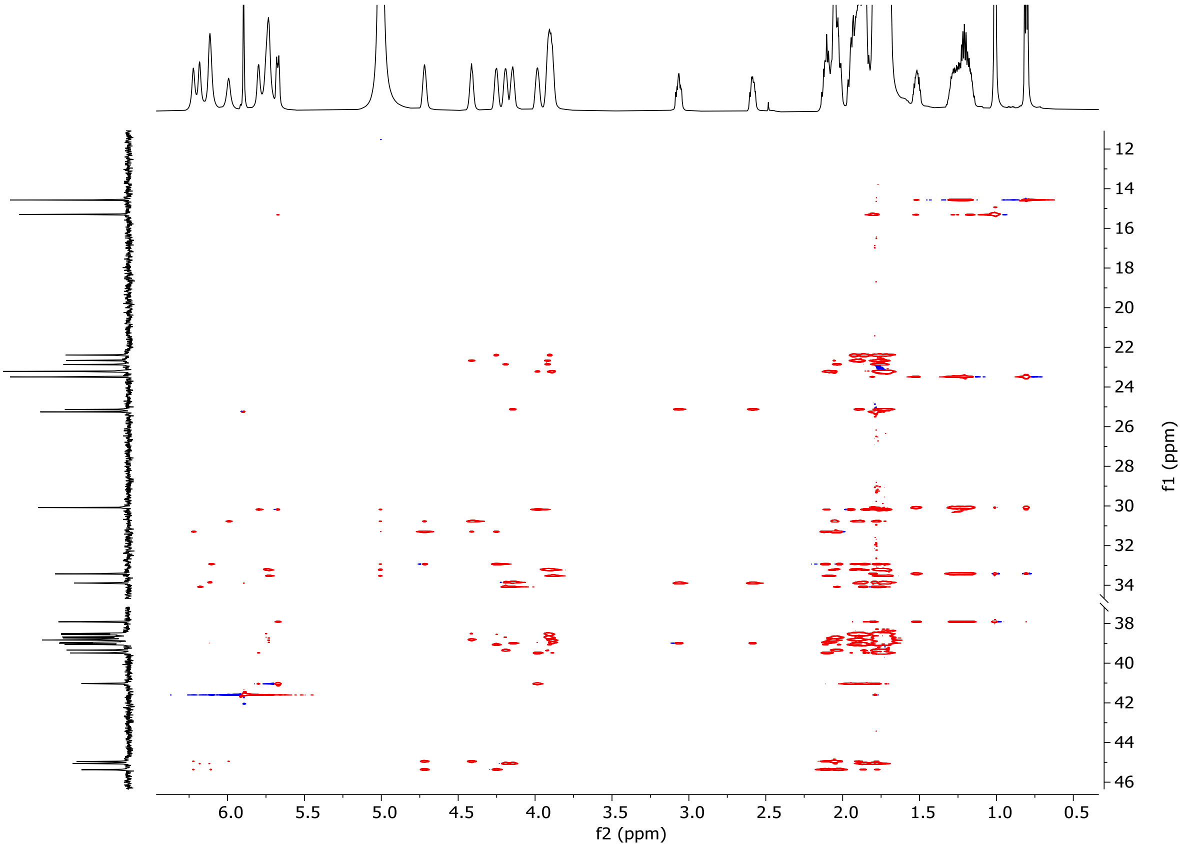

Figure S8: Ultra-high-resolution HSQC-TOCSY of *iso*-caylobolide B (**2**) in pyridine-d_5 (Phase-sensitive ge-2D HSQC-TOCSY with DIPSI-2 using PEP and adiabatic inversion and refocusing pulses: hsqcdietgpsisp.2) with a 30 ms mixing time. Spectrometer frequency (F2: 700.10 MHz, F1: 176.05 MHz) acquired size (t2: 1024, t1: 723), spectral size (t2: 1024, t1: 2048), spectral width (F2: 7352.9 Hz, F1: 6684.6Hz), 4 scans. Spectrum centred at the aliphatic region.

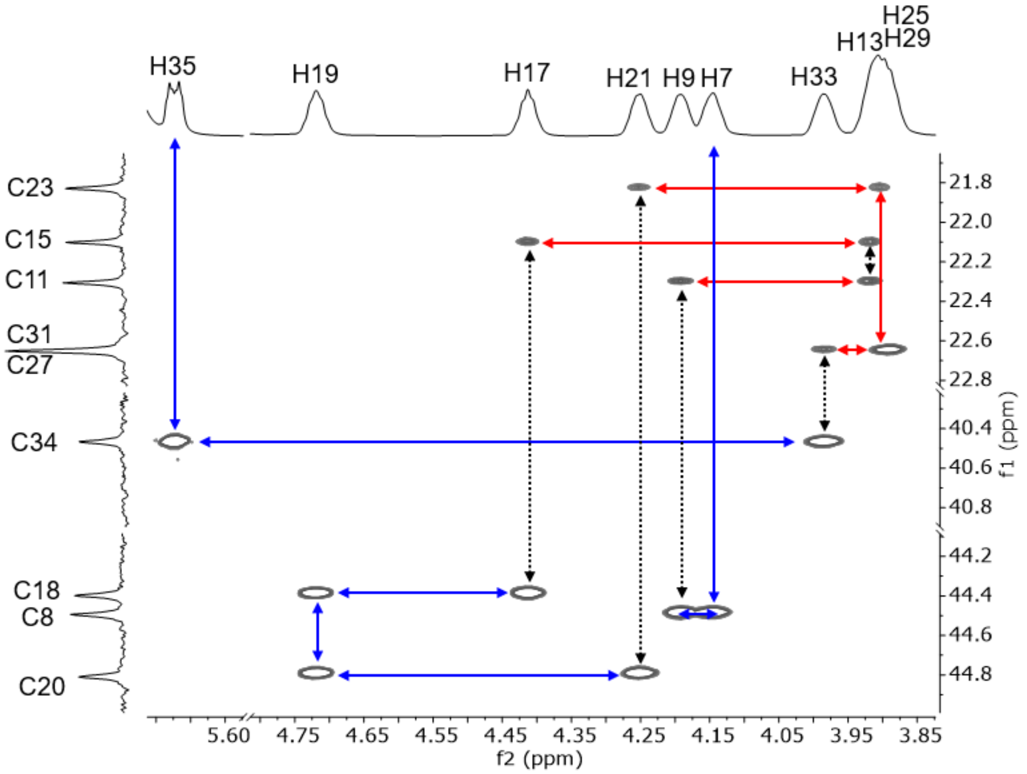

Figure S9: Zoomed in region of ultra-high-resolution HSQC-TOCSY spectrum (700 MHz, pyridine-d_5) with 30 ms mixing time, showing $^{2\text{-}3}J_{CH}$ correlations from HCOR signals to methylene carbon atoms. Blue indicates correlations connecting 1,3-related systems, red indicates correlations connecting 1,5-related systems displaying the key correlations utilised to determine the connectivity of *iso*-caylobolide B (**2**).

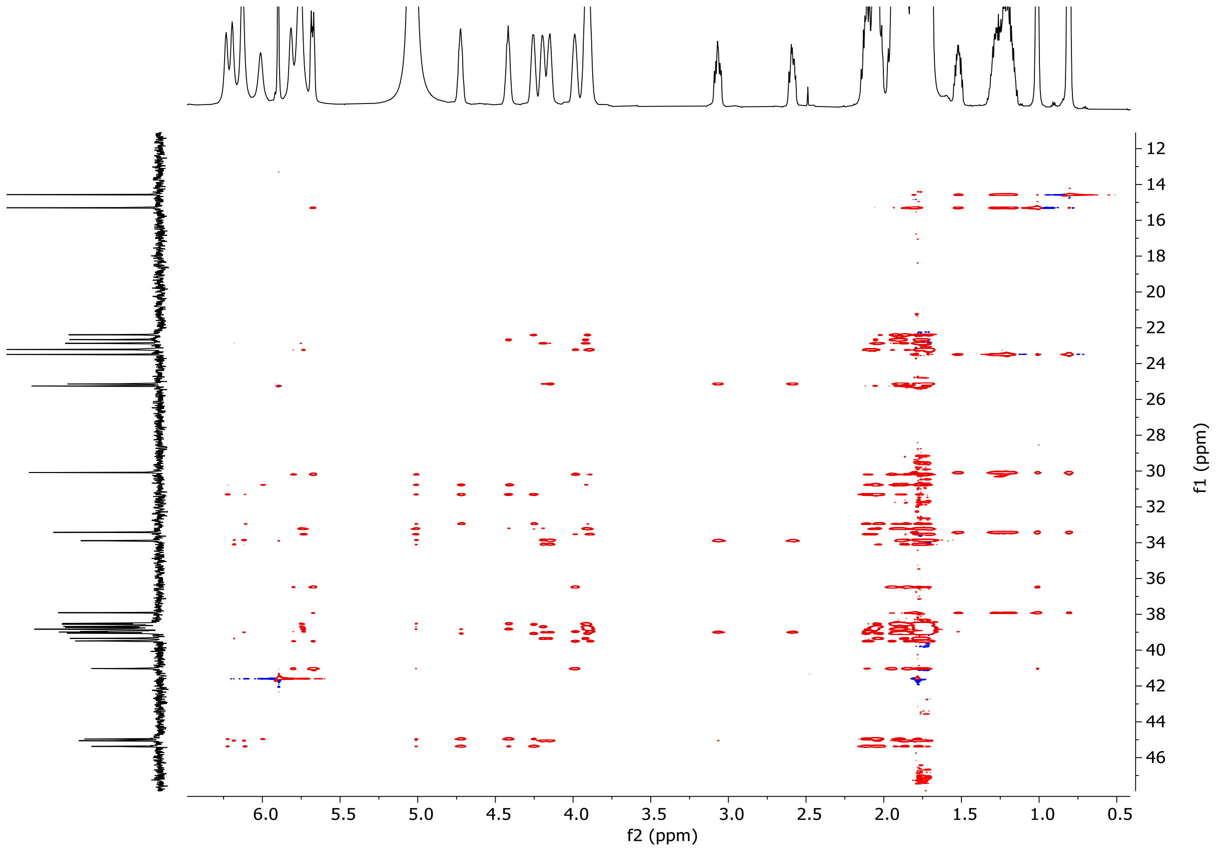

Figure S10: Ultra-high-resolution HSQC-TOCSY of *iso*-caylobolide B (**2**) in pyridine-d_5 (Phase-sensitive ge-2D HSQC-TOCSY with DIPSI-2 using PEP and adiabatic inversion and refocusing pulses: hsqcdietgpsisp.2) with a 60 ms mixing time. Spectrometer frequency (F2: 700.10 MHz, F1: 176.05 MHz) acquired size (t2: 1024, t1: 723), spectral size (t2: 1024, t1: 2048), spectral width (F2: 7352.9 Hz, F1: 6684.6Hz), 16 scans. Spectrum centred at the aliphatic region.

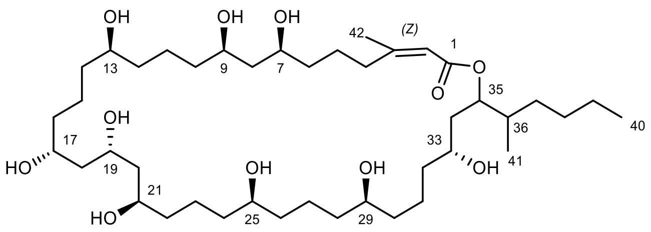

Table S1: ^{1}H and ^{13}C NMR analysis of *iso*-caylobolide B (**2**) in pyridine-d_5 at 700 MHz and 151 MHz respectively.

Position, type	δC (176 MHz, pyr-d_5)	δH (700 MHz, pyr-d_5)
**1 C**	167.13	-
**2 CH**	117.69	5.92
**3 C**	160.57	-
**4 CH_2**	33.91	2.59; 3.11
**5 CH_2**	25.13	1.76; 1.92
**6 CH_2**	38.99	1.77
**7 CHOH**	71.81	4.18
**8 CH_2**	45.03	1.83; 1.90
**9 CHOH**	72.01	4.23
**10 CH_2**	39.32	1.79
**11 CH_2**	22.84	1.81; 2.07
**12 CH_2**	38.66	1.77
**13 CHOH**	71.17	3.95
**14 CH_2**	38.51	1.78
**15 CH_2**	22.66	1.94
**16 CH_2**	38.82	1.80; 1.92
**17 CHOH**	68.73	4.45
**18 CH_2**	44.98	2.08
**19 CHOH**	69.25	4.75
**20 CH_2**	45.38	2.05; 2.14
**21 CHOH**	70.89	4.28
**22 CH_2**	39.06	1.79; 1.90
**23 CH_2**	22.38	1.89; 1.96
**24 CH_2**	38.57	1.77
**25 CHOH**	71.2	3.93
**26 CH_2**	38.71	1.74
**27 CH_2**	23.23	1.80; 2.12
**28 CH_2**	38.8	1.73
**29 CHOH**	71.5	3.92
**30 CH_2**	38.95	1.77
**31 CH_2**	23.23	1.80; 2.12
**32 CH_2**	39.57	1.74; 1.81
**33 CHOH**	67.98	4.02
**34 CH_2**	39.26	1.85; 1.93
**35 CHOR**	74.92	5.69
**36 CH**	37.51	1.94
**37 CH_2**	32.87	1.22; 1.51
**38 CH_2**	30.05	1.24; 1.37
**39 CH_2**	23.49	1.24
**40 CH_3**	14.55	0.83
**41 CH_3**	15.62	1
**42 CH_3**	25.25	1.82

3.3.6 ^{1}H NMR (DMSO-d6, 600 MHz)

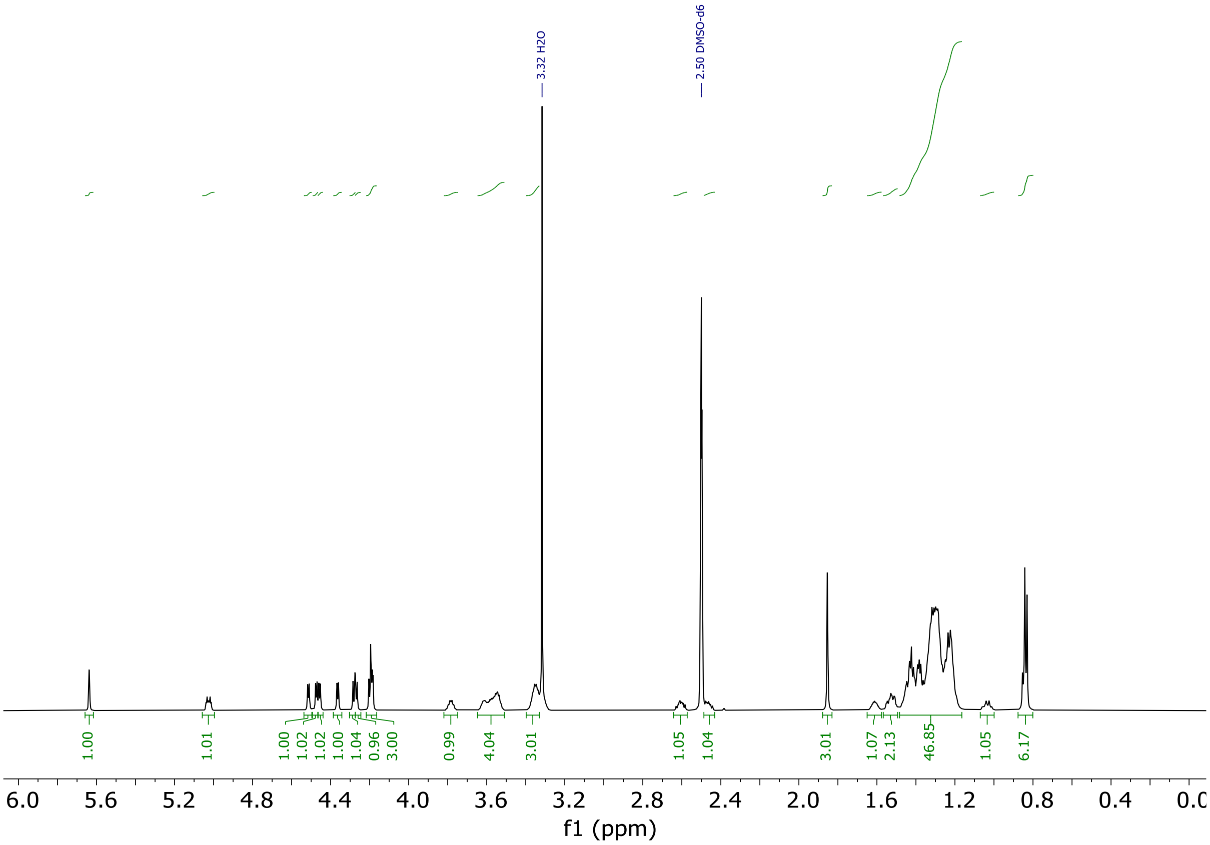

Figure S11: ^{1}H NMR spectrum (600 MHz, DMSO-d_6) of *iso*-caylobolide B (**2**).

3.3.7 HSQC NMR (DMSO-d6, 600 MHz)

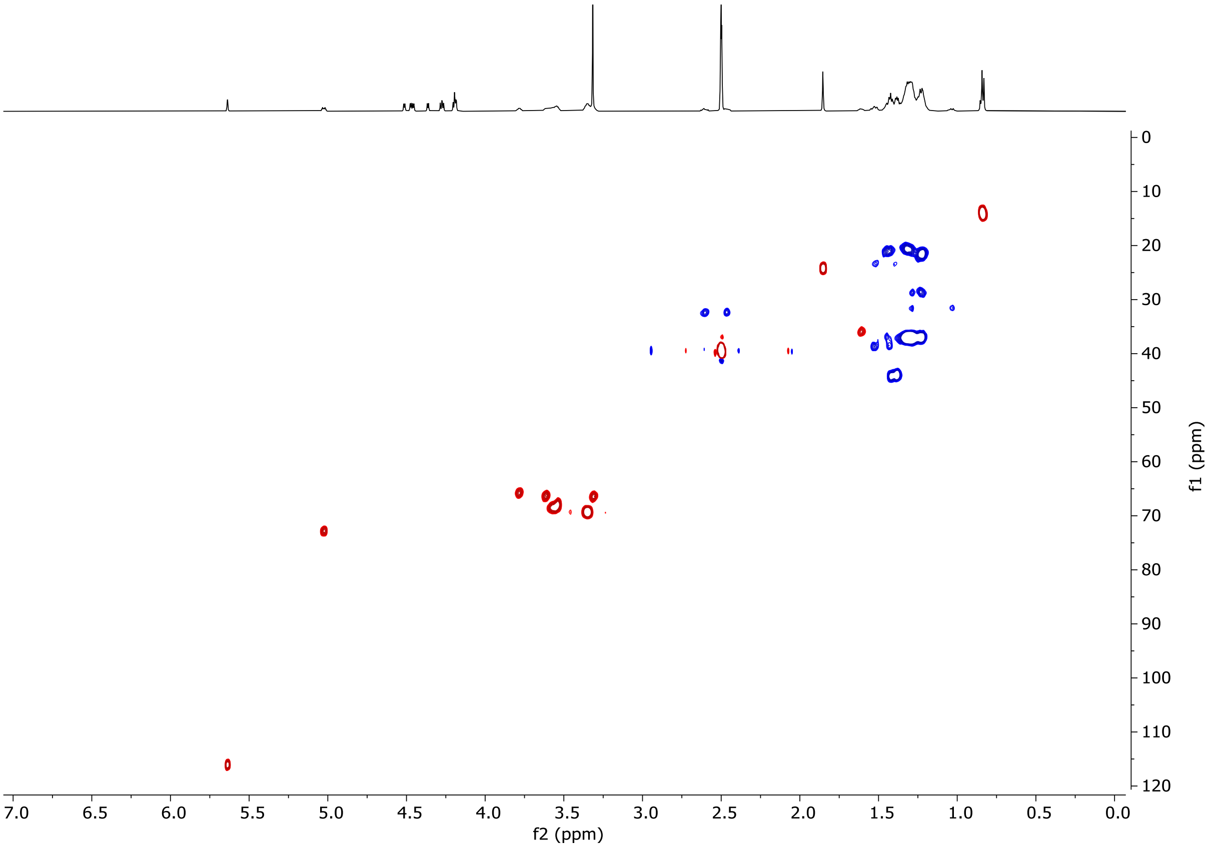

Figure S12: HSQC spectrum (600 MHz, DMSO-d_6) of *iso*-caylobolide B (**2**).

3.3.8 COSY NMR (DMSO-d6, 600 MHz)

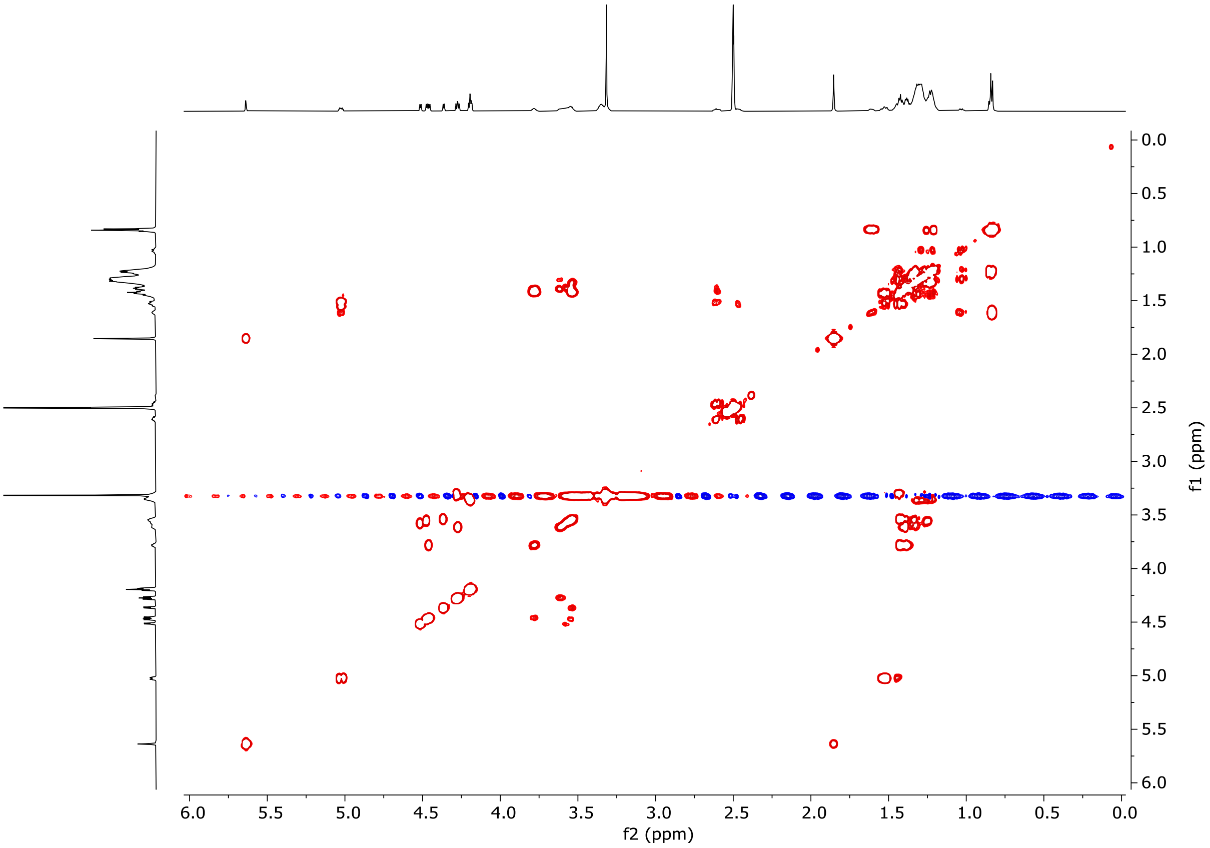

Figure S13: COSY spectrum (600 MHz, DMSO-d_6) of *iso*-caylobolide B (**2**).

3.3.9 HMBC NMR (DMSO-d6, 600 MHz)

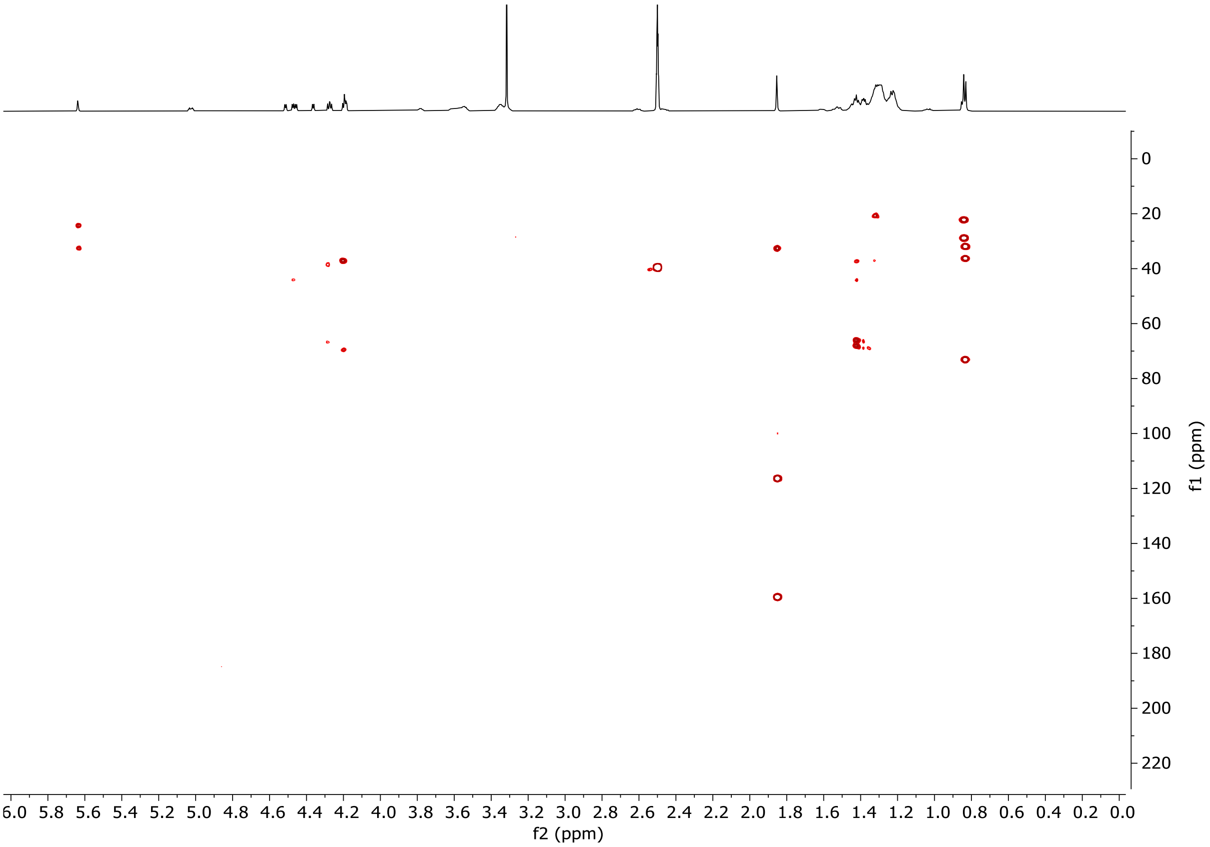

Figure S14: HMBC NMR spectrum (600 MHz, DMSO-d_6) of *iso*-caylobolide B (**2**).

3.3.10 HSQC-TOCSY NMR (DMSO-d6, 600 MHz)

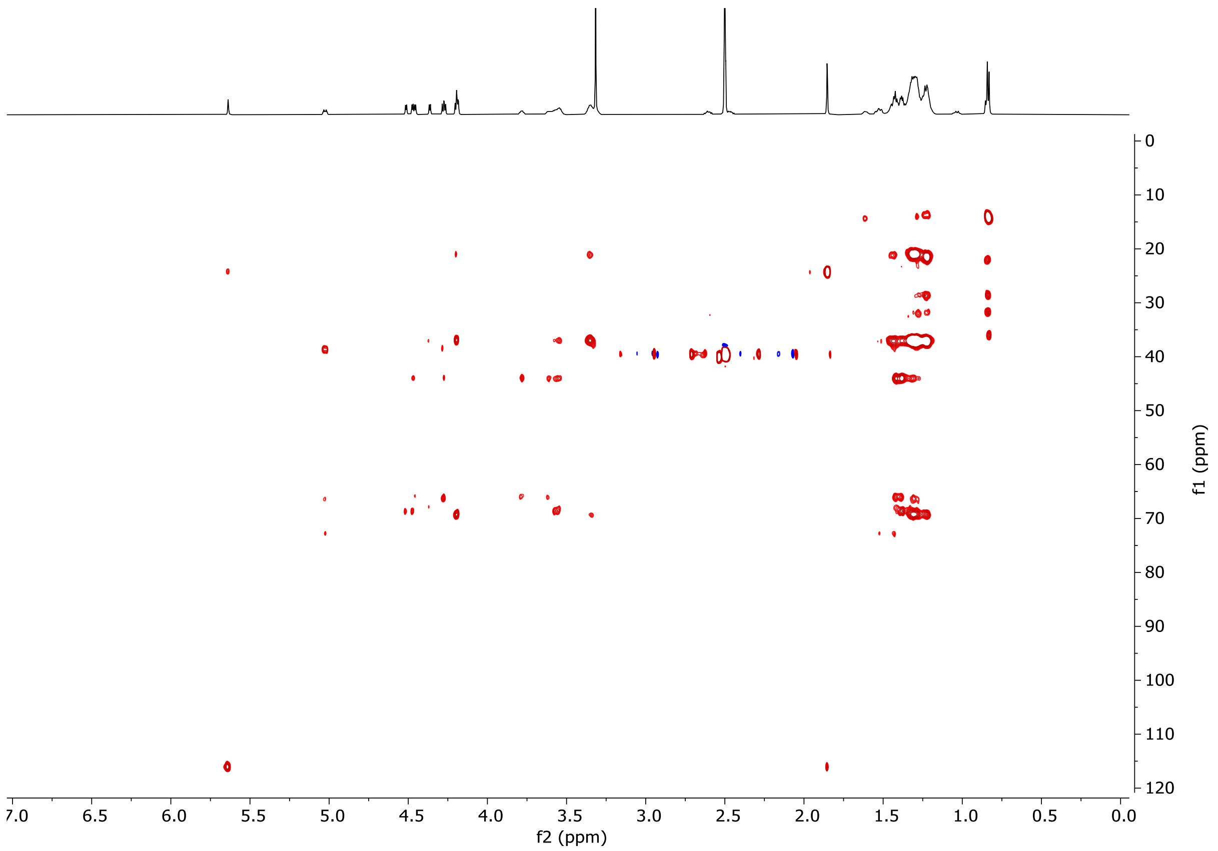

Figure S15: HSQC-TOCSY spectrum (600 MHz, DMSO-d_6) of *iso*-caylobolide B (**2**).

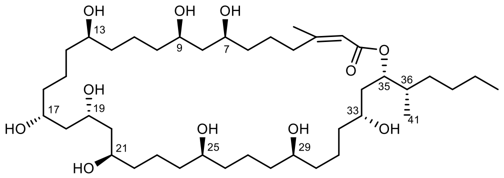

Table S2: ^{1}H and ^{13}C NMR analysis of *iso*-caylobolide B in DMSO-d_6.

Position	δ_C, type	δ_H (*J* in Hz)	H-H COSY	HMBC	HSQC-TOCSY
**1**	unassigned, C				
**2**	116.1, CH	5.64, d (0.9)	42	4, 42	
**3**	159.5, C				
**4**	32.5, CH_2	a: 2.61	5		
		b: 2.47	5		
**5**	23.5, CH_2	a: 1.53	4a, 4b		
		b: 1.41	4a, 4b	6, 7	
**6**	37.00, CH_2	a:1.36	7		
		b:1.28	7		
**7**	68.6, CH	3.58	OH_h, 6, 8		
**8**	44.1, CH_2	1.38	7, 9		
**9**	68.8, CH	3.56	OH_g, 8, 10		
**10**	37.5, CH_2	a: 1.26	9		
		b:1.24	9		
**11**	21.1, CH_2	a: 1.45	12		
		b: 1.24	12		
**12**	36.90, CH_2	1.30	12a, 12b,13		
**13**	69.3 CH	3.36	OH_a, 12, 14		
**14**	37.0, CH_2	1.24	13	13	
**15**	20.5-20.9, CH_2	1.20-1.39			
**16**	37.4, CH_2	a:1.32			17, 18
		b:1.28			17, 18
**17**	66.4, CH	3.62	OH_c, 18		
**18**	43.9, CH_2	1.39	17, 19		
**19**	65.9, CH	3.79, dq (11.6, 6)	OH_f, 18, 20a, 20b		
**20**	44.3, CH_2	a: 1.42	19, 21	18, 22	
		b: 1.39	19, 21		
**21**	67.9, CH	3.54	OH_e, 20a, 20b, 22		
**22**	37.1, CH_2	1.32	21		
**23**	20.5-20.9, CH_2	1.20-1.39			
**24**	36.95, CH_2	1.28	25		
**25**	69.2 CH	3.35	OH_b, 24, 26b		
**26**	36.5, CH_2	a:1.33	25		
		b:1.23			
**27**	21.0, CH_2	a:1.33			
		b:1.24			
**28**	36-38.3, CH_2	1.20-1.34	29		
			29		
**29**	69.6, CH	3.35,	OHi, 28, 30		
**30**	36-38.3, CH_2	1.20-1.34	29		
**31**	20.8, CH_2	a:1.46	32a		
		b:1.33	32a, 32b		
**32**	37.3, CH_2	a:1.30	31a, 33		
		b:1.28	31a, 31b, 33		
**33**	66.4, CH	3.31	OH_d, 34a, 34b, 32		
**34**	38.7, CH_2	a: 1.54	33, 35		
		b: 1.44	33, 35		
**35**	72.8, CH	5.03, dt (9.3, 3.4)	34a,34b, 36		
**36**	36.0, CH	1.61, m	35, 37b, 41		
**37**	31.8, CH_2	a: 1.30	38		
		b:1.03	36, 38		
**38**	28.7, CH_2	a: 1.28	37a, 37b		37, 39
		b: 1.22	37a, 37b		37, 39
**39**	22.1, CH_2	a: 1.25	40		
		b:1.22	40		

**40**	13.7, CH$_3$	0.84	39a, 39b	38 39	
**41**	14.4, CH$_3$	0.83	36	35, 36, 37	
**42**	24.3, CH$_3$	1.85, d (1.2)	2	2, 3, 4	
**OH$_a$**	4.18		13	12, 13, 14	12, 14
**OH$_b$**	4.19		25	24, 25, 26	24
**OH$_c$**	4.27		17	16, 17, 18	18
**OH$_d$**	4.28		33	32, 33, 34	32, 33, 34
**OH$_e$**	4.36		21	21, 22	20, 21, 22
**OH$_f$**	4.46		19	18, 20	18, 19, 20
**OH$_g$**	4.47		9	8, 10	9, 8, 10
**OH$_h$**	4.51		7	6, 8	6, 7, 8
**OHi**	4.20		29	28, 29, 30	28, 29, 30

3.4 Caylobolide B NMR Analysis

3.4.1 ¹H NMR (700 MHz, pyridine-d_5)

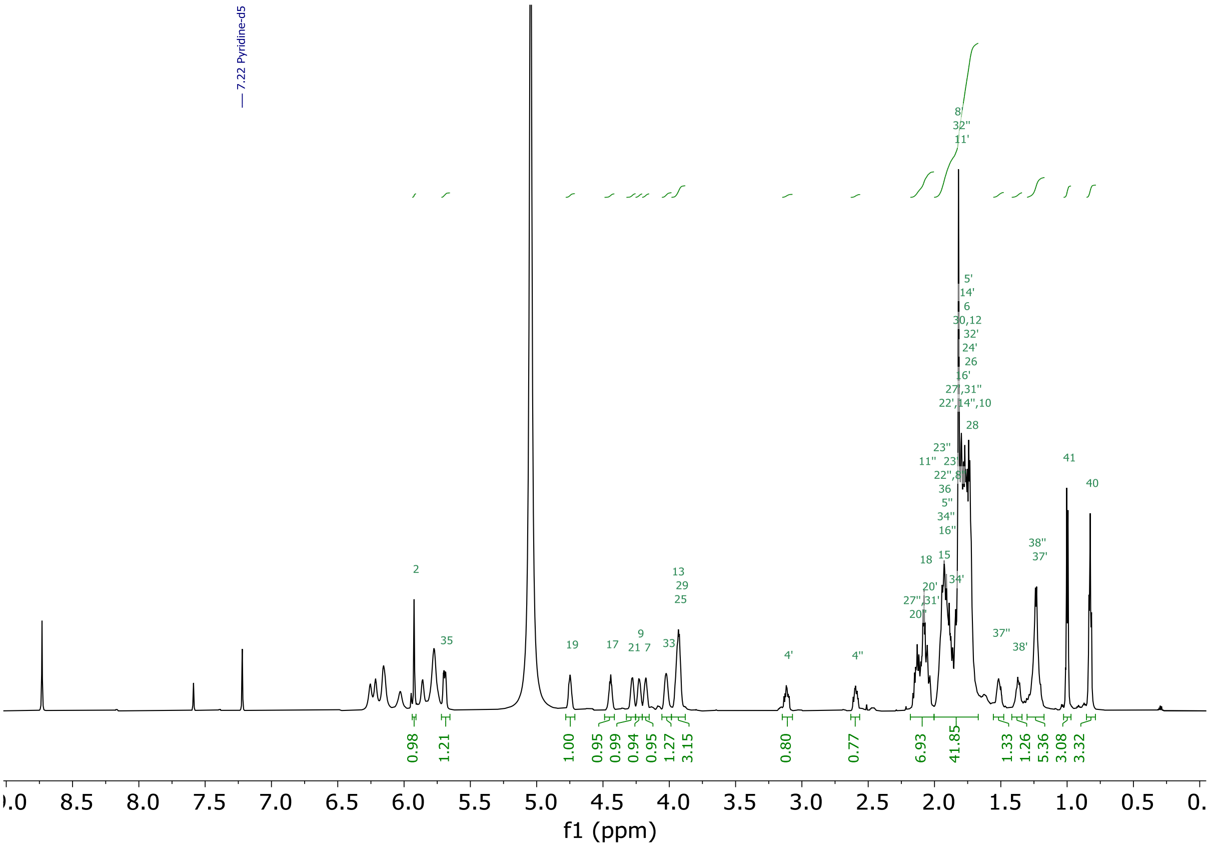

Figure S16: ¹H NMR spectrum (700 MHz, pyridine-d_5) of caylobolide B (**1**).

3.4.2 ^{13}C NMR (151 MHz, pyridine-d_5)

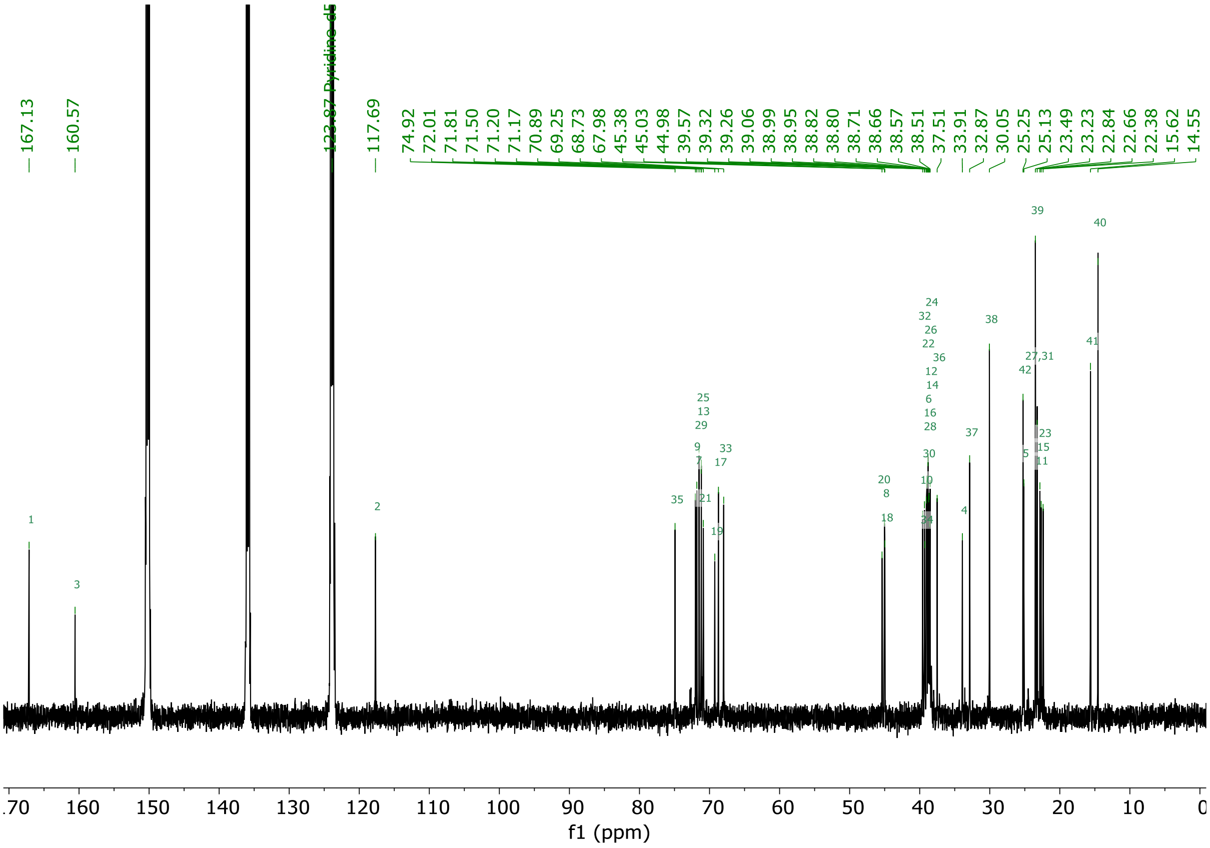

Figure S17: ^{13}C NMR spectrum (151 MHz, pyridine-d_5) of caylobolide B (**1**).

3.4.3 HMBC NMR (pyridine-d_5, 700 MHz)

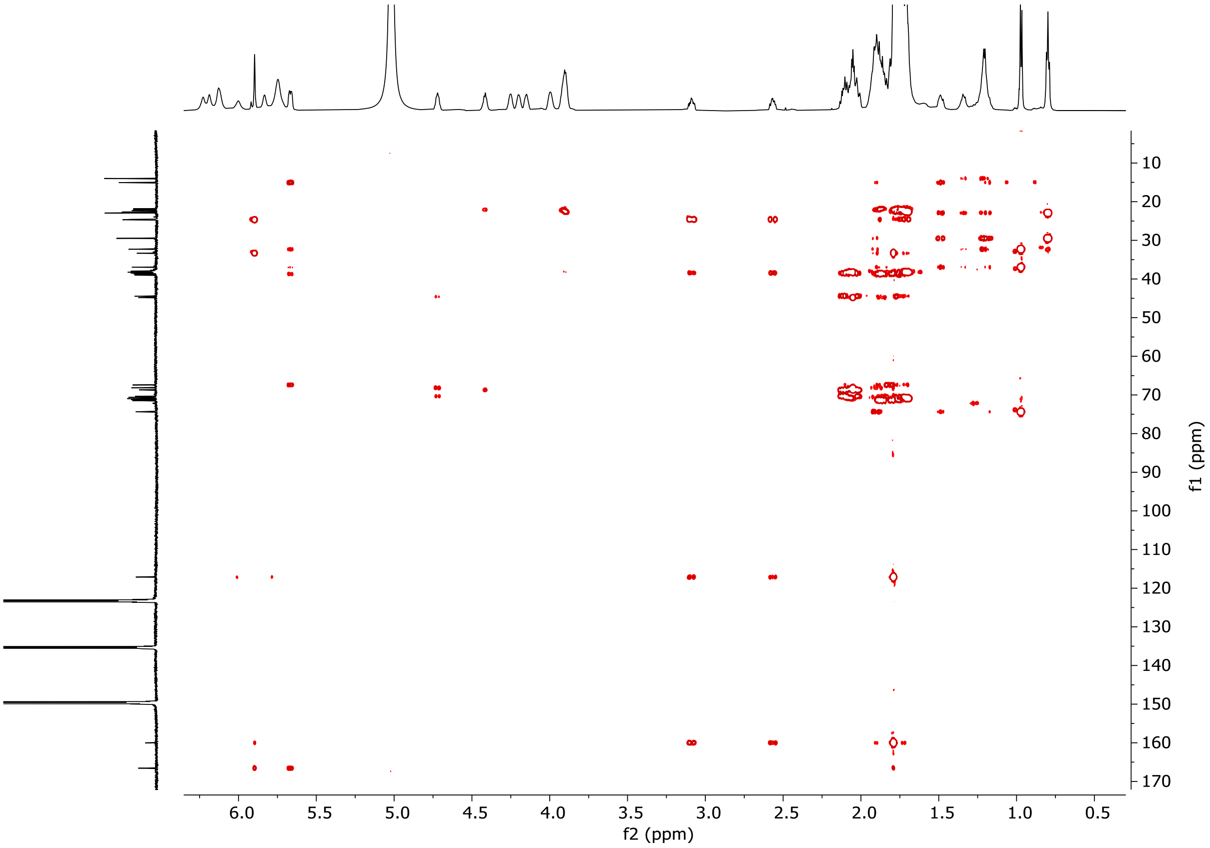

Figure S18: HMBC spectrum (700 MHz, pyridine-d_5) of caylobolide B (**1**).

3.4.4 Multiplicity Edited Pure Shift HSQC NMR

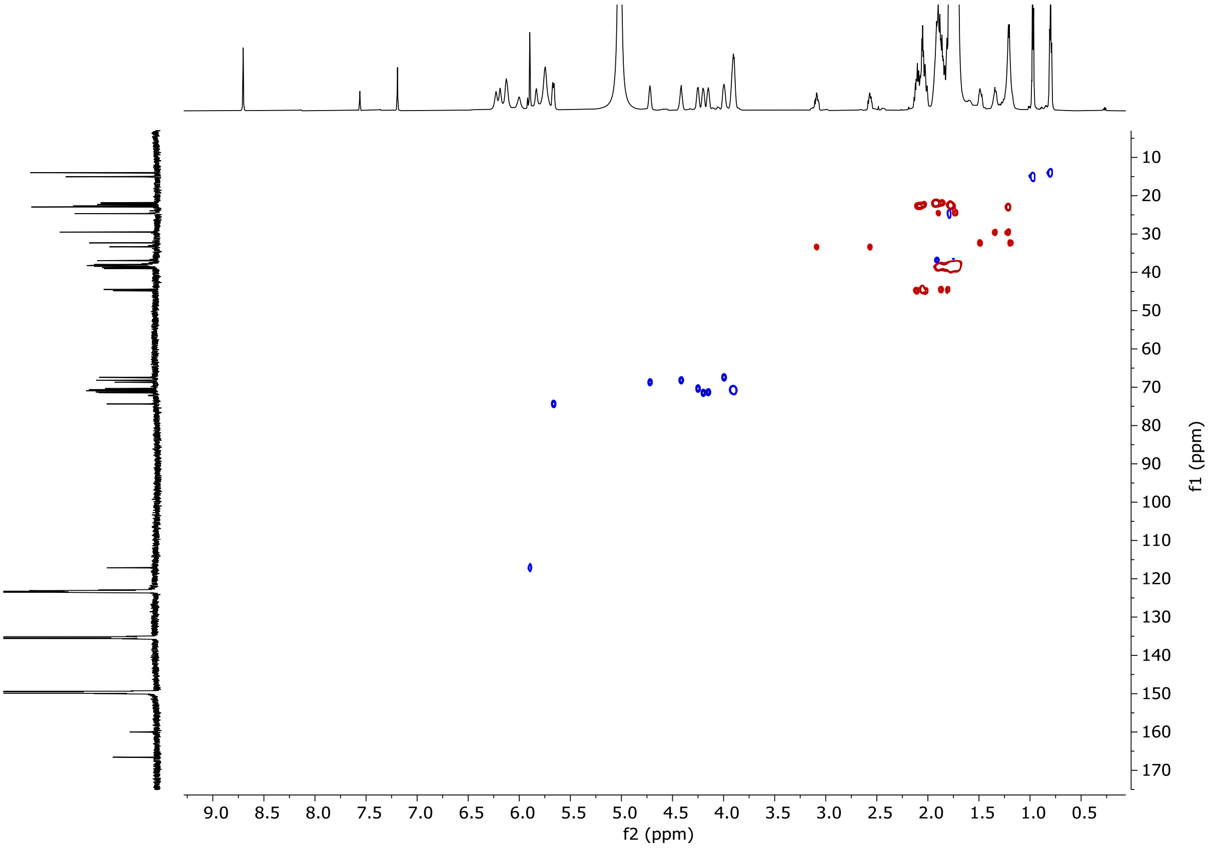

Figure S19: Full HSQC spectrum (700 MHz, pyridine-d_5) of caylobolide B (**1**).

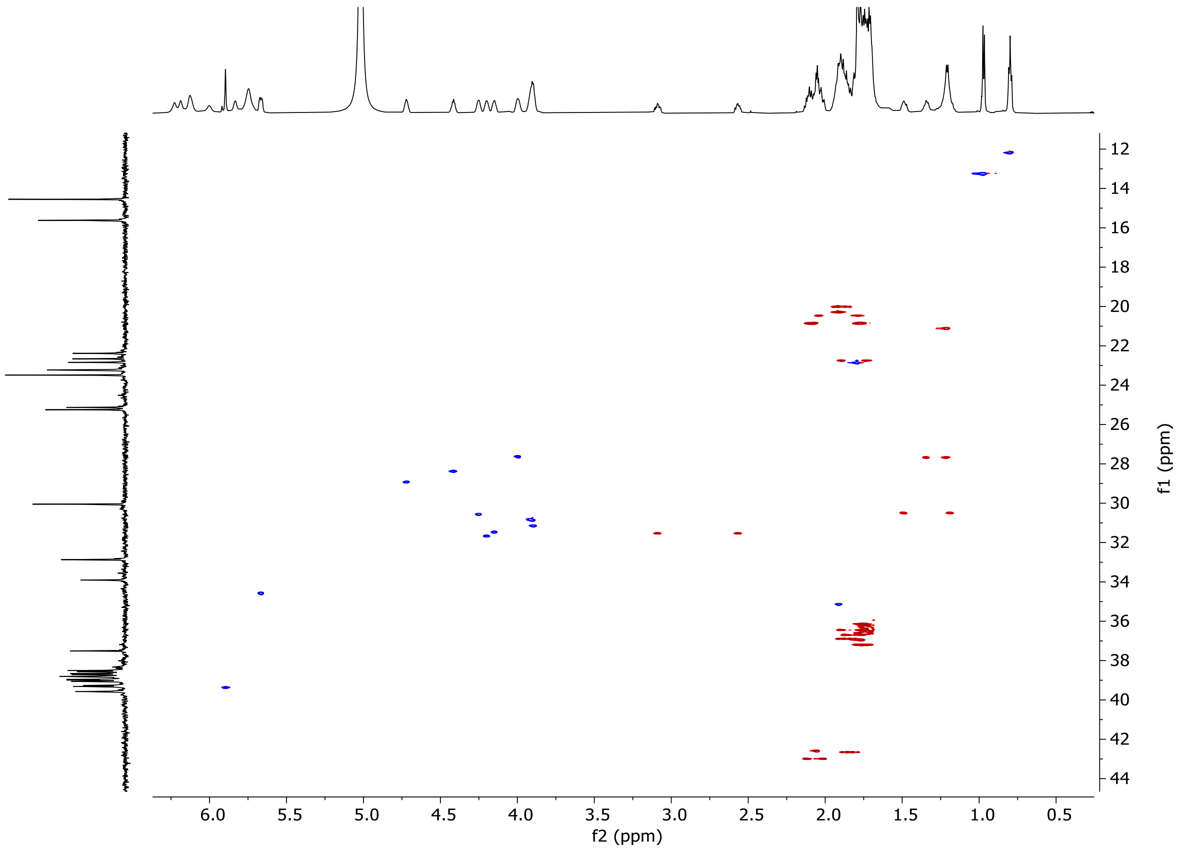

Figure S20: Ultra-high-resolution pure shift HSQC of caylobolide B (**1**) in pyridine-d_5 (Phase-sensitive ge-2D multiplicity-edited HSQC using echo-antiecho and inversion and matched sweep adiabatic pulses with broadband homodecoupling: hsqcedetgpsp.3_bbhd). Spectrometer frequency (F2: 700.10 MHz, F1: 176.05 MHz), acquired size (t2: 597, t1: 1024), spectral size (t2: 1024, t1: 8192), spectral width (F2: 7352.9 Hz, F1: 6684.5 Hz), 2 scans. Spectrum centred at the aliphatic region where CHO correlations are folded back into the lower ppm region (correlations between 3-6 ppm in ^{1}H).

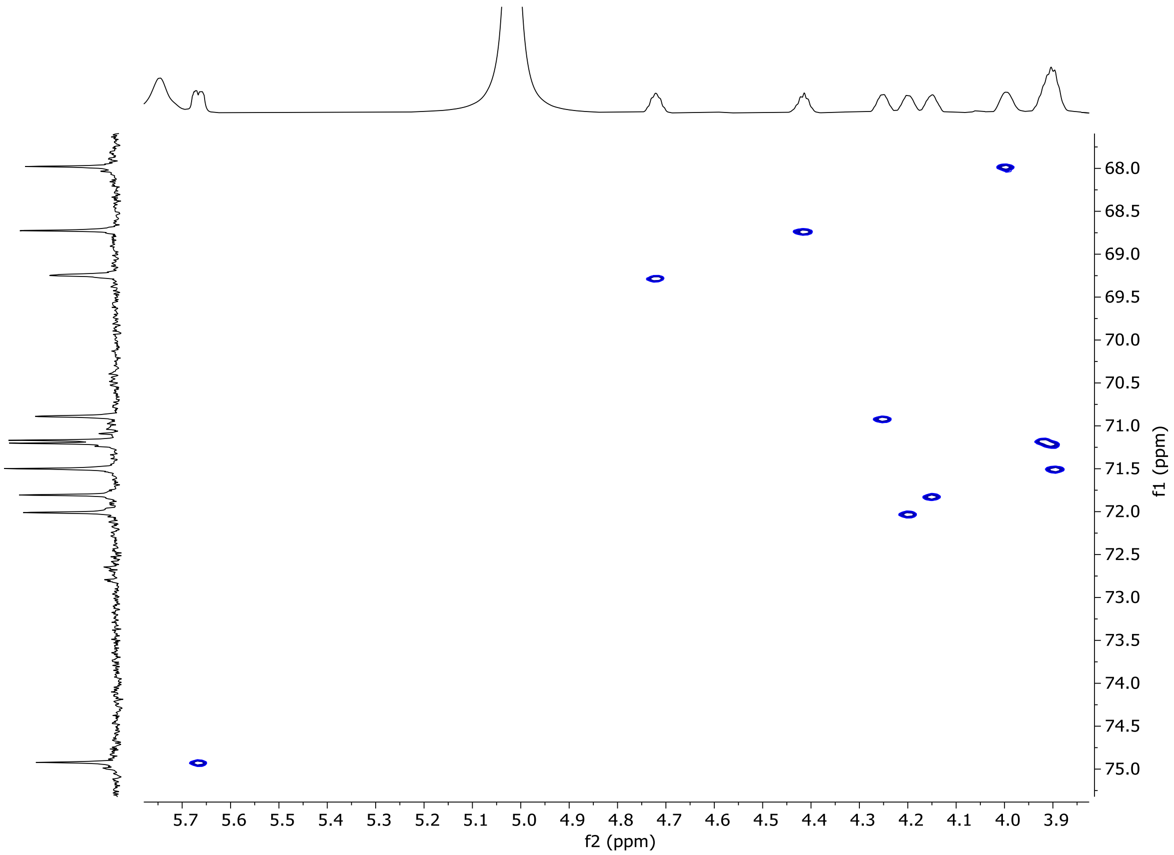

Figure S21: Zoomed in region (^{13}C: 67-75 ppm, ^{1}H: 3.8-5.8 ppm) of ultra-high-resolution pure shift HSQC of caylobolide B (**1**) in pyridine-d_5. Spectra recentred to align with CHO correlations.

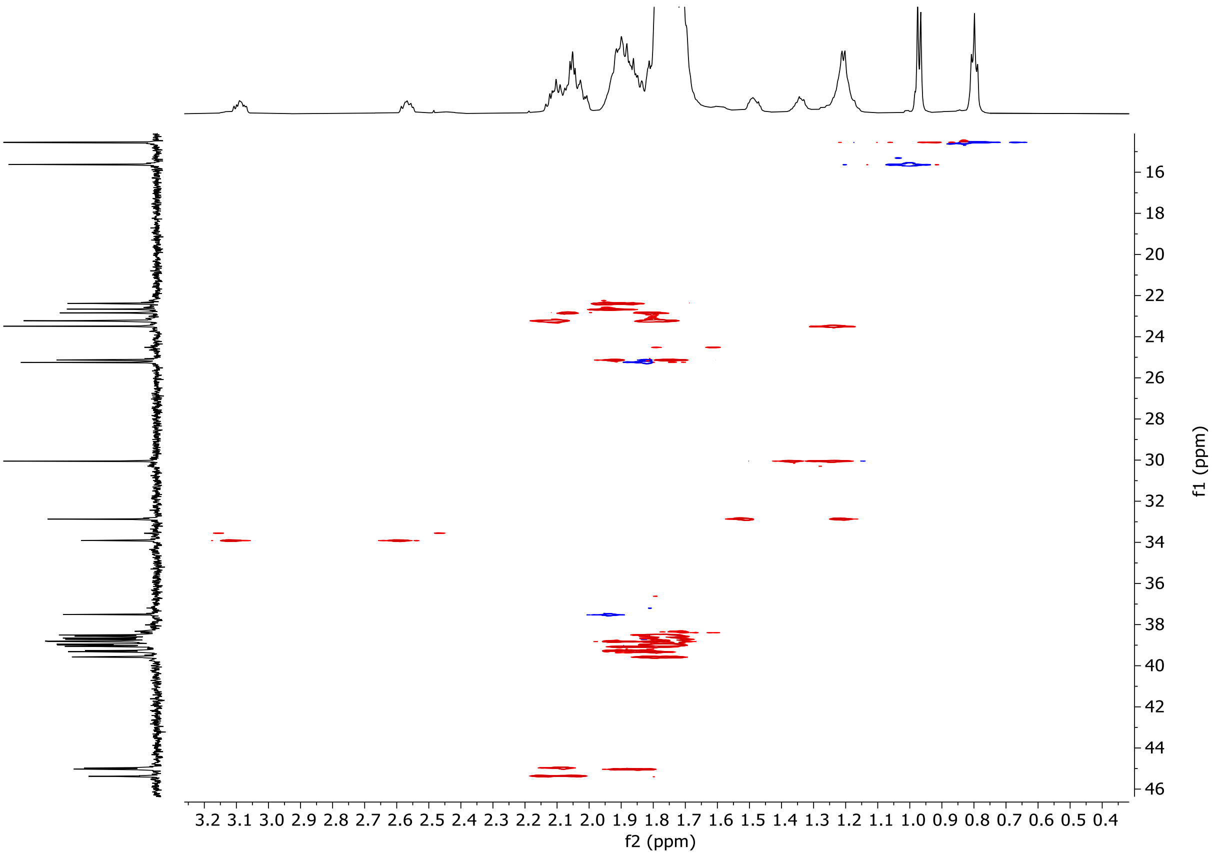

Figure S22: Zoomed in region (^{13}C: 14-46 ppm, ^{1}H: 0.4-3.2 ppm) of ultra-high-resolution pure shift HSQC of caylobolide B (**1**) in pyridine-d_5. Spectra recentred to align with alkyl region.

3.4.5 HSQC-TOCSY NMR (pyridine-d_5, 700 MHz)

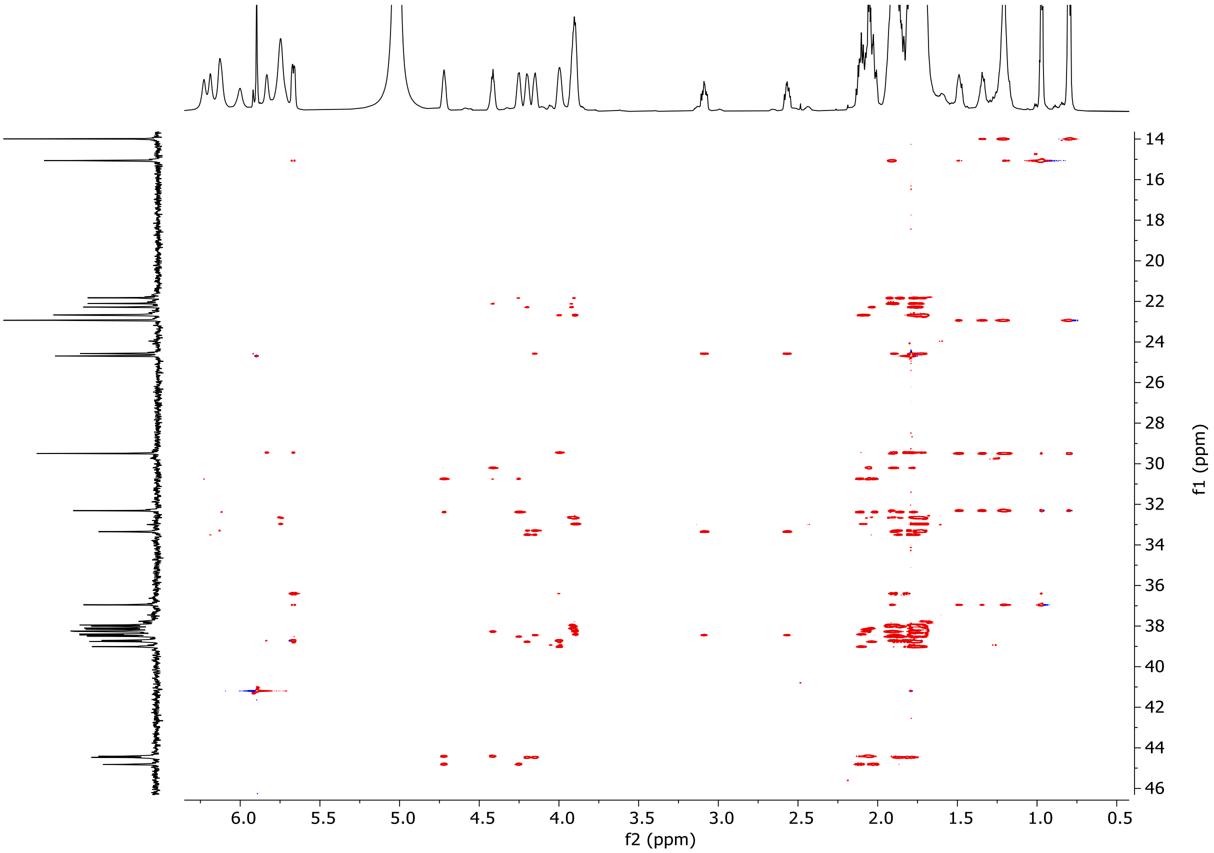

Figure S23: Ultra-high-resolution HSQC-TOCSY of caylobolide B (**1**) in pyridine-d_5 (Phase-sensitive ge-2D HSQC-TOCSY with DIPSI-2 using PEP and adiabatic inversion and refocusing pulses: hsqcdietgpsisp.2) with a 30 ms mixing time. Spectrometer frequency (F2: 700.10 MHz, F1: 176.05 MHz) acquired size (t2: 1024, t1: 723), spectral size (t2: 1024, t1: 2048), spectral width (F2: 7352.9 Hz, F1: 6684.6Hz), 4 scans. Spectrum centred at the aliphatic region.

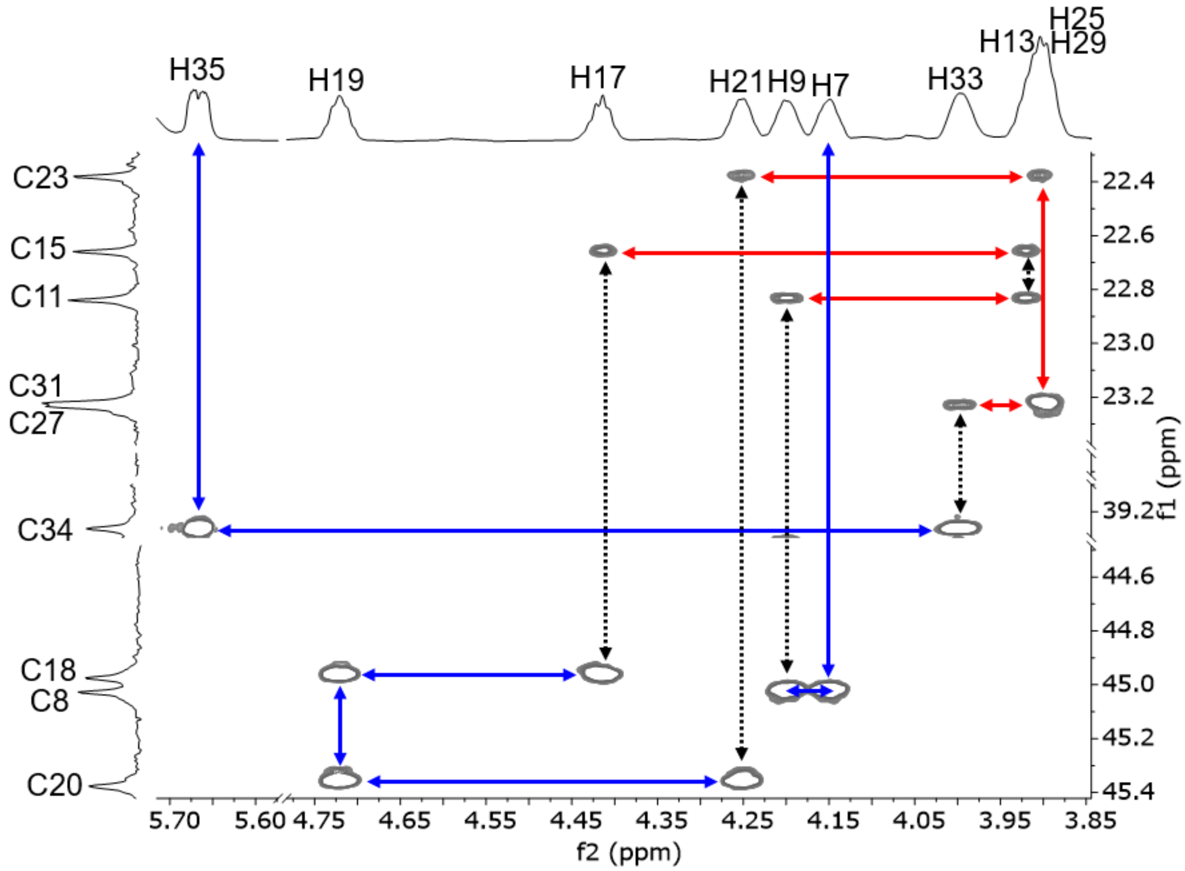

Figure S24: Zoomed in region of ultra-high-resolution HSQC-TOCSY spectrum (700 MHz, pyridine-d_5) with 30 ms mixing time, showing $^{2\text{-}3}J_{CH}$ correlations from HCOR to methylene carbon atoms. Blue indicates correlations connecting 1,3-related systems, red indicates correlations connecting 1,5-related systems displaying the key correlations utilised to determine connectivity of caylobolide B (**1**).

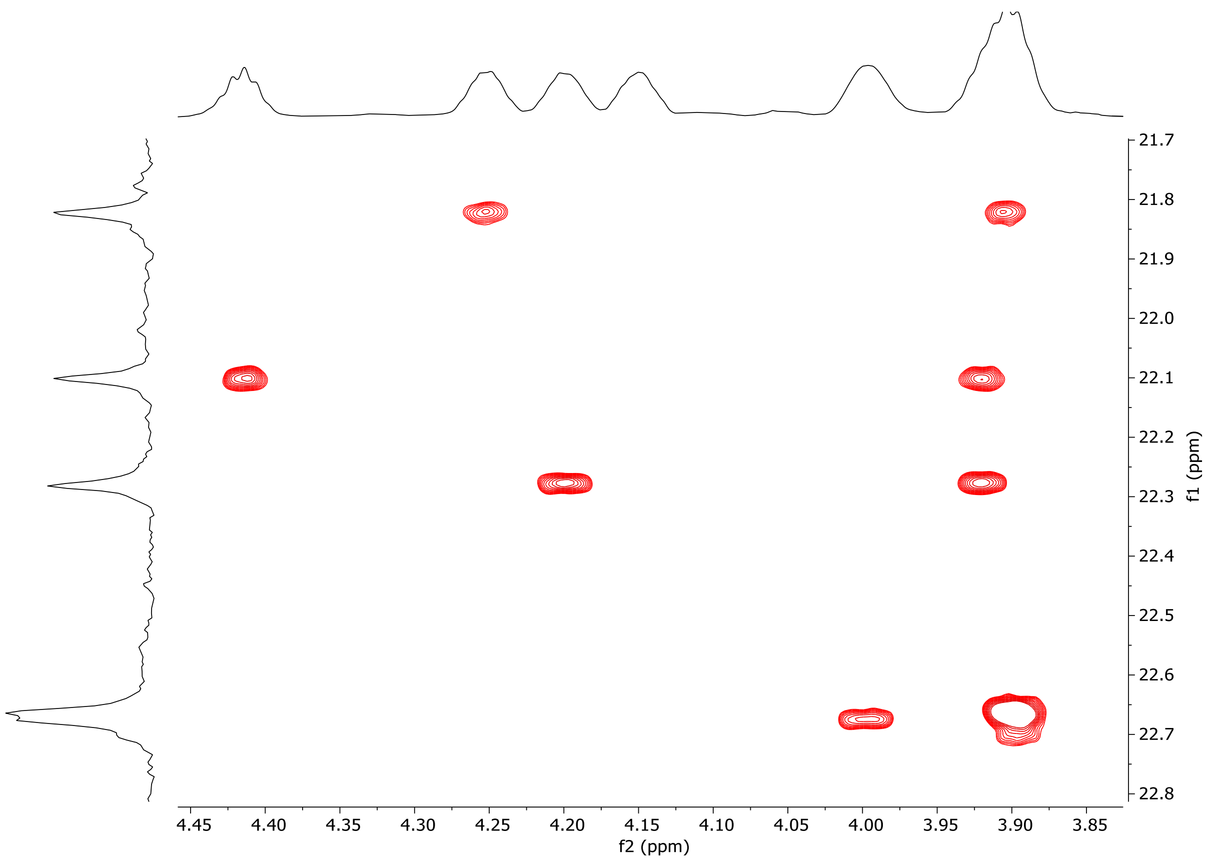

Figure S25: Zoomed in region (^{13}C: 22-24 ppm, ^{1}H: 3.7-4.5 ppm) of ultra-high-resolution pure shift HSQC-TOCSY (30 ms mixing time) of caylobolide B (**1**) in pyridine-d_5 showing $^{3}J_{CH}$ correlations from *H*COH to β-methylene carbon atoms.

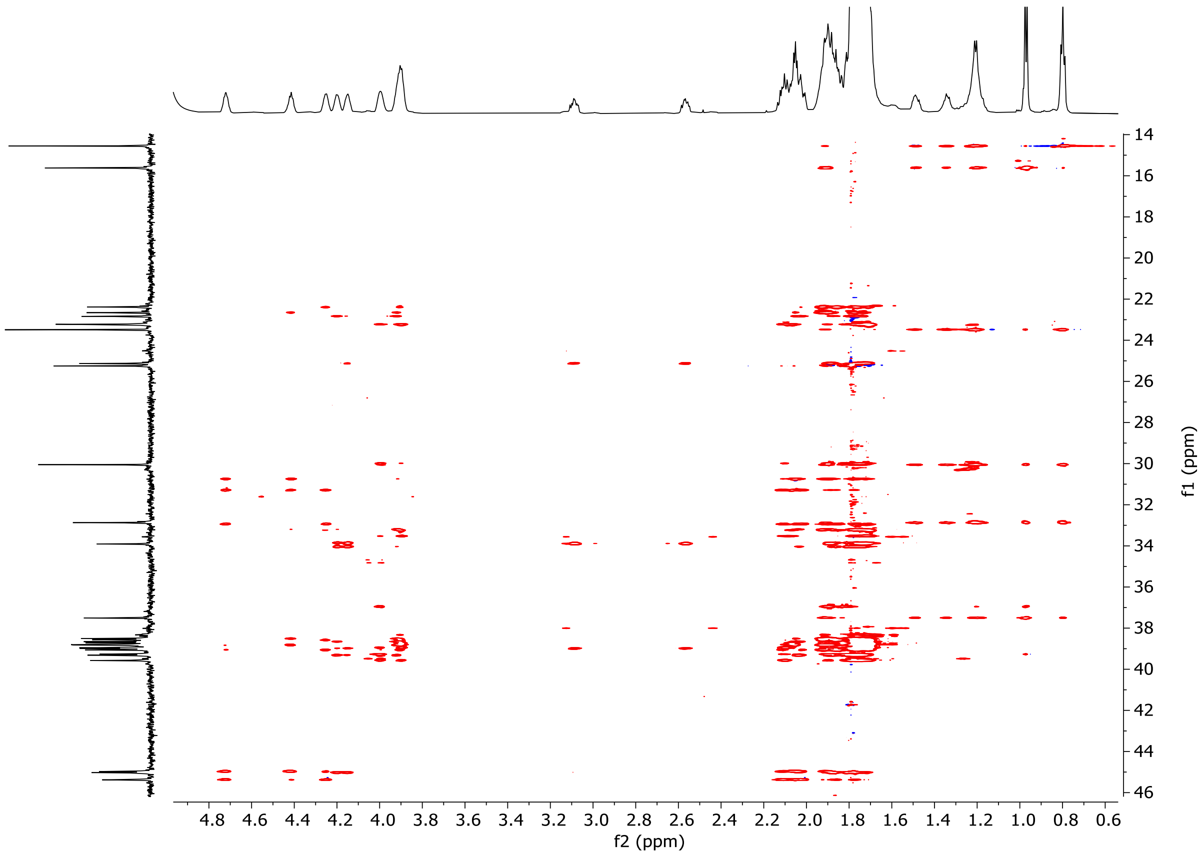

Figure S26: Ultra-high-resolution HSQC-TOCSY of caylobolide B (**1**) in pyridine-d_5 (Phase-sensitive ge-2D HSQC-TOCSY with DIPSI-2 using PEP and adiabatic inversion and refocusing pulses: hsqcdietgpsisp.2) with a 60 ms mixing time. Spectrometer frequency (F2: 700.10 MHz, F1: 176.05 MHz) acquired size (t2: 1024, t1: 723), spectral size (t2: 1024, t1: 2048), spectral width (F2: 7352.9 Hz, F1: 6684.6Hz), 16 scans. Spectrum centred at the aliphatic region.

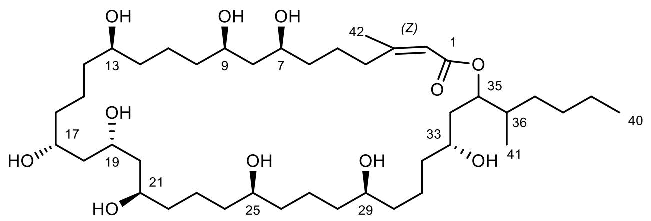

Table S3: ^{1}H and ^{13}C NMR analysis of caylobolide B (**1**) in pyridine-*d*5 at 700 MHz and 151 MHz respectively.

Position, type	δC (176 MHz, pyr-*d*5)	δH (700 MHz, pyr-*d*5)
**1 C**	167.23	-
**2 CH**	117.53	5.93
**3 C**	160.78	-
**4 CH_2**	33.89	2.62; 3.10
**5 CH_2**	25.14	1.77; 1.93
**6 CH_2**	38.99	1.76
**7 CHOH**	71.81	4.18
**8 CH_2**	45.05	1.83; 1.90
**9 CHOH**	72.04	4.23
**10 CH_2**	39.34	1.80
**11 CH_2**	22.87	1.82; 2.07
**12 CH_2**	38.67	1.77
**13 CHOH**	71.17	3.95
**14 CH_2**	38.5	1.79
**15 CH_2**	22.66	1.94
**16 CH_2**	38.82	1.81; 1.94
**17 CHOH**	68.73	4.44
**18 CH_2**	44.96	2.09
**19 CHOH**	69.26	4.75
**20 CH_2**	45.37	2.04; 2.15
**21 CHOH**	70.89	4.29
**22 CH_2**	39.05	1.81; 1.90
**23 CH_2**	22.39	1.89; 1.96
**24 CH_2**	38.55	1.78
**25 CHOH**	71.2	3.93
**26 CH_2**	38.73	1.74
**27 CH_2**	23.21	1.80; 2.12
**28 CH_2**	38.83	1.73
**29 CHOH**	71.5	3.92
**30 CH_2**	38.96	1.76
**31 CH_2**	23.21	1.80; 2.12
**32 CH_2**	39.48	1.76; 1.79
**33 CHOH**	68.16	4.01
**34 CH_2**	41.03	1.89; 1.98
**35 CHOR**	74.44	5.70
**36 CH**	37.91	1.84
**37 CH_2**	33.42	1.19; 1.55
**38 CH_2**	30.08	1.24; 1.31
**39 CH_2**	23.49	1.24
**40 CH_3**	14.57	0.84
**41 CH_3**	15.3	1.04
**42 CH_3**	25.25	1.82

Table S4: 13C chemical shift comparison between caylobolide B and iso-caylobolide B in pyridine-*d*$_5$. >0.10 ppm differences highlighted in red.

Position, type	Atom	iso-CB δC (ppm)	CB δC (ppm)	ΔδC
**1 C**	1	167.23	167.13	0.10
**2 CH**	2	117.53	117.69	-0.16
**3 C**	3	160.78	160.57	0.21
**4 CH_2**	4	33.89	33.91	-0.02
**5 CH_2**	5	25.14	25.13	0.01
**6 CH_2**	6	38.99	38.99	0.00
**7 CHOH**	7	71.81	71.81	0.00
**8 CH_2**	8	45.05	45.03	0.02
**9 CHOH**	9	72.04	72.01	0.03
**10 CH_2**	10	39.34	39.32	0.02
**11 CH_2**	11	22.87	22.84	0.03
**12 CH_2**	12	38.67	38.66	0.01
**13 CHOH**	13	71.17	71.17	0.00
**14 CH_2**	14	38.5	38.51	-0.01
**15 CH_2**	15	22.66	22.66	0.00
**16 CH_2**	16	38.82	38.82	0.00
**17 CHOH**	17	68.73	68.73	0.00
**18 CH_2**	18	44.96	44.98	-0.02
**19 CHOH**	19	69.26	69.25	0.01
**20 CH_2**	20	45.37	45.38	-0.01
**21 CHOH**	21	70.89	70.89	0.00
**22 CH_2**	22	39.05	39.06	-0.01
**23 CH_2**	23	22.39	22.38	0.01
**24 CH_2**	24	38.55	38.57	-0.02
**25 CHOH**	25	71.2	71.2	0.00
**26 CH_2**	26	38.73	38.71	0.02
**27 CH_2**	27	23.21	23.23	-0.02
**28 CH_2**	28	38.83	38.8	0.03
**29 CHOH**	29	71.5	71.5	0.00
**30 CH_2**	30	38.96	38.95	0.01
**31 CH_2**	31	23.21	23.23	-0.02
**32 CH_2**	32	39.48	39.57	-0.09
**33 CHOH**	33	68.16	67.98	0.18
**34 CH_2**	34	41.03	39.26	1.77
**35 CHOR**	35	74.44	74.92	-0.48
**36 CH**	36	37.91	37.51	0.40
**37 CH_2**	37	33.42	32.87	0.55
**38 CH_2**	38	30.08	30.05	0.03
**39 CH_2**	39	23.49	23.49	0.00
**40 CH_3**	40	14.57	14.55	0.02
**41 CH_3**	41	15.3	15.62	-0.32
**42 CH_3**	42	25.25	25.25	0.00

3.4.6 ^{1}H NMR (DMSO-d6, 600 MHz)

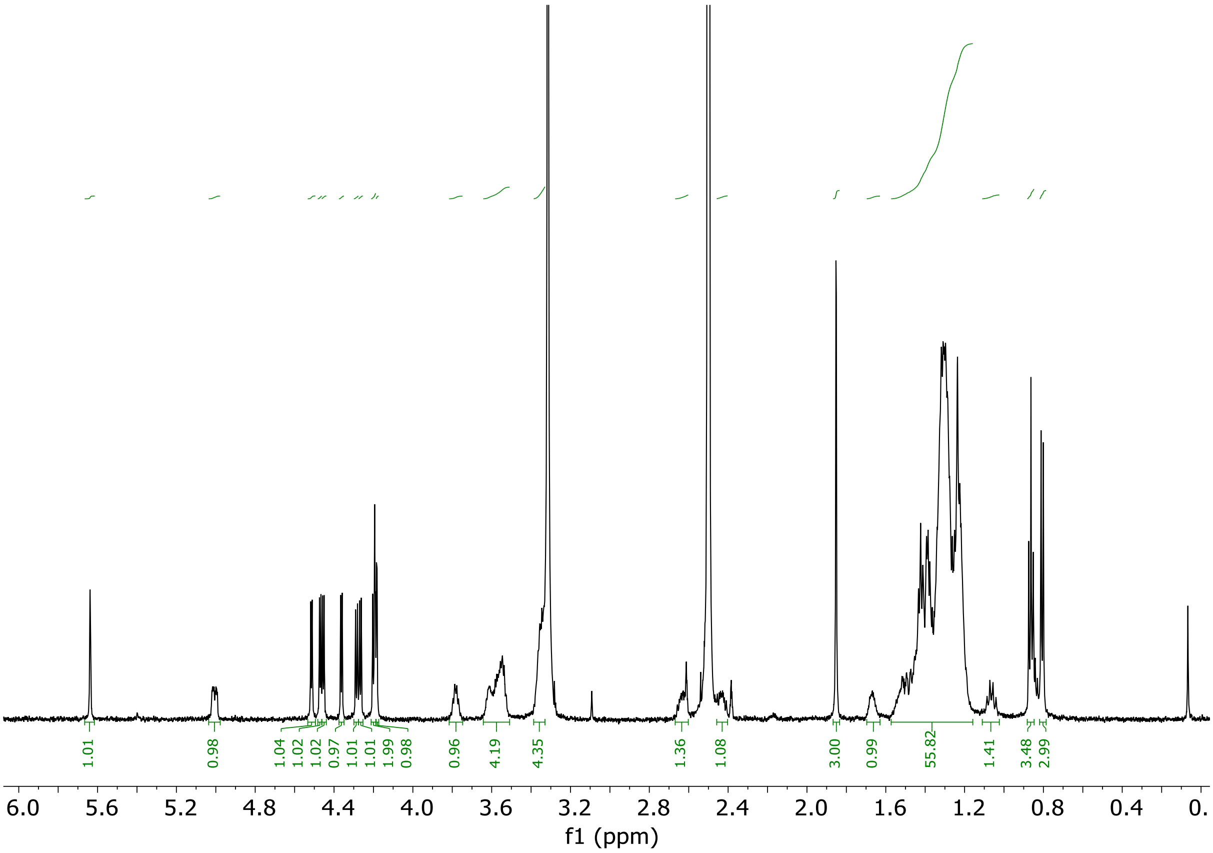

Figure S27: ^{1}H NMR spectrum (600 MHz, DMSO-d_6) of caylobolide B (**1**).

3.4.7 HSQC NMR (DMSO-d6, 600 MHz)

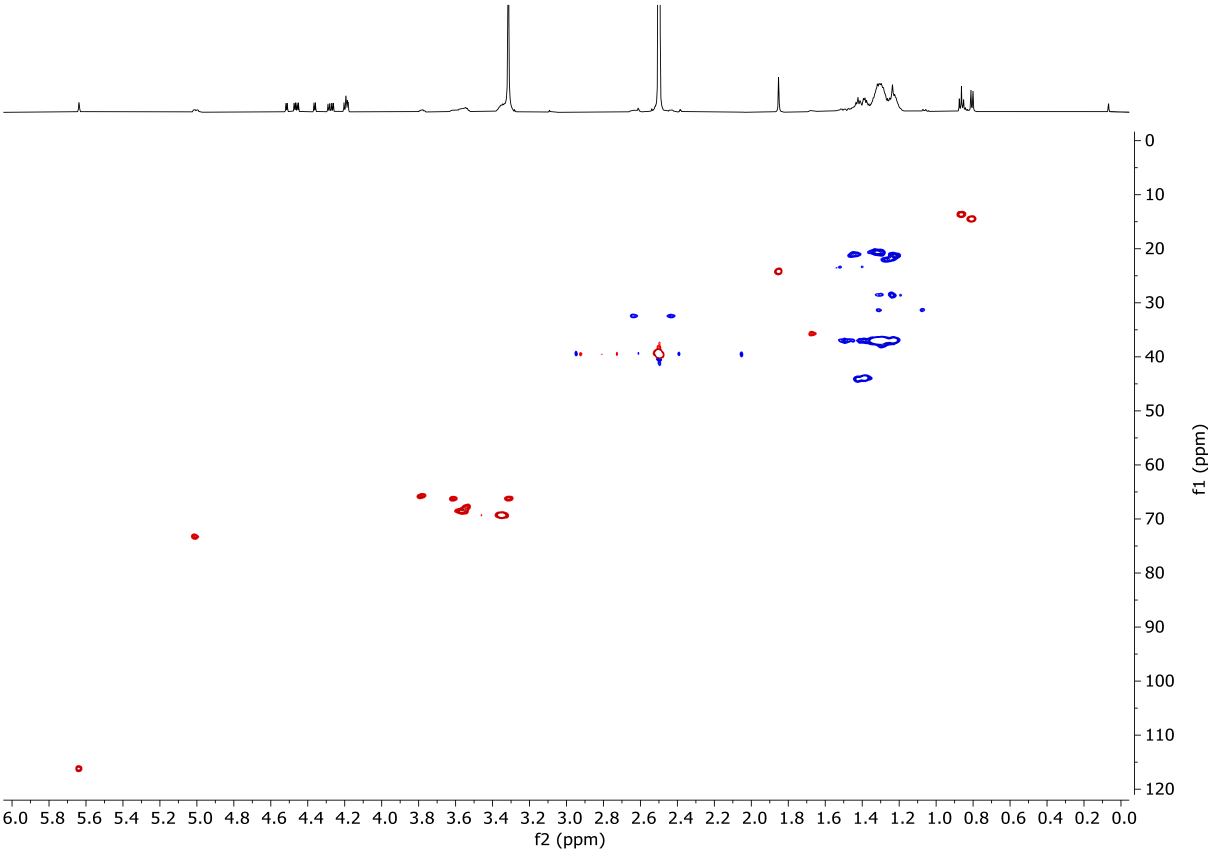

Figure S28: HSQC spectrum (600 MHz, DMSO-d_6) of caylobolide B (**1**).

3.4.8 COSY NMR (DMSO-d6, 600 MHz)

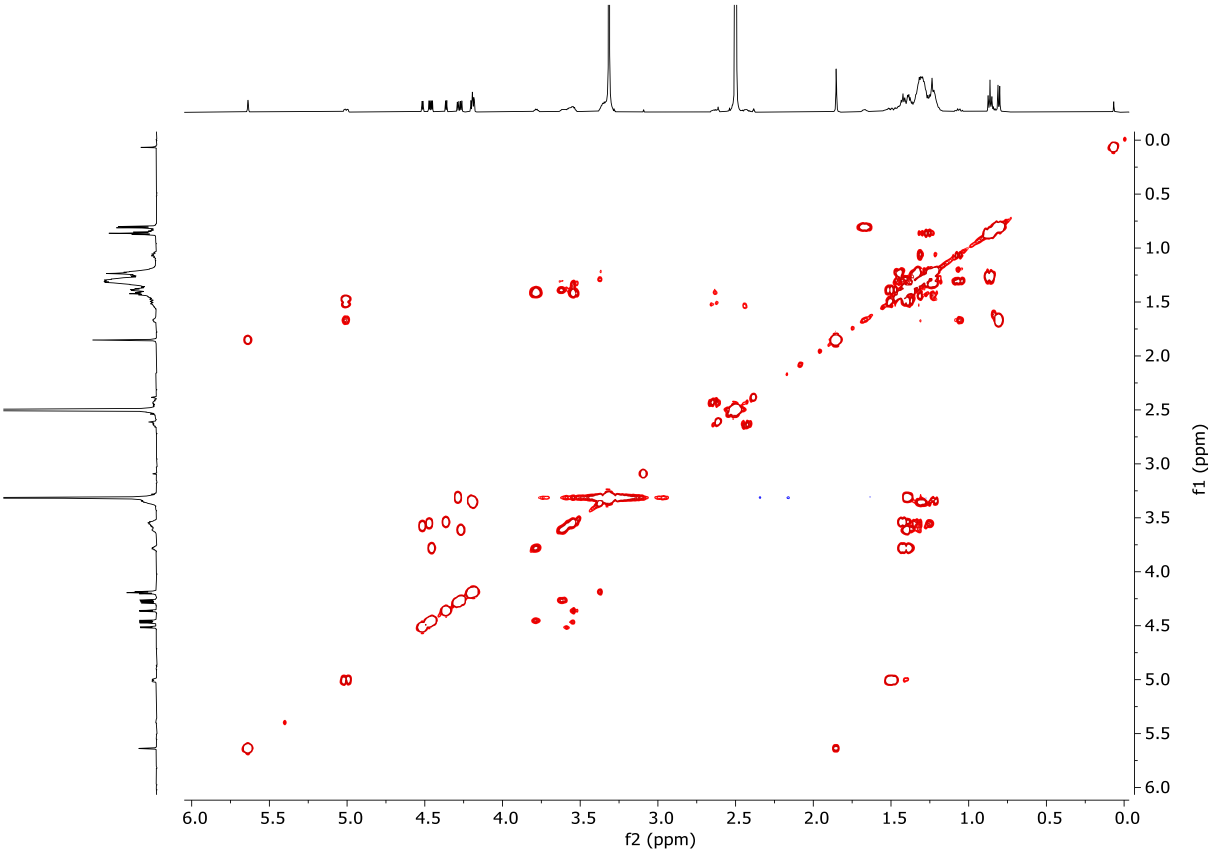

Figure S29: COSY NMR spectrum (600 MHz, DMSO-d_6) of caylobolide B (**1**).

3.4.9 HMBC NMR (DMSO-d6, 600 MHz)

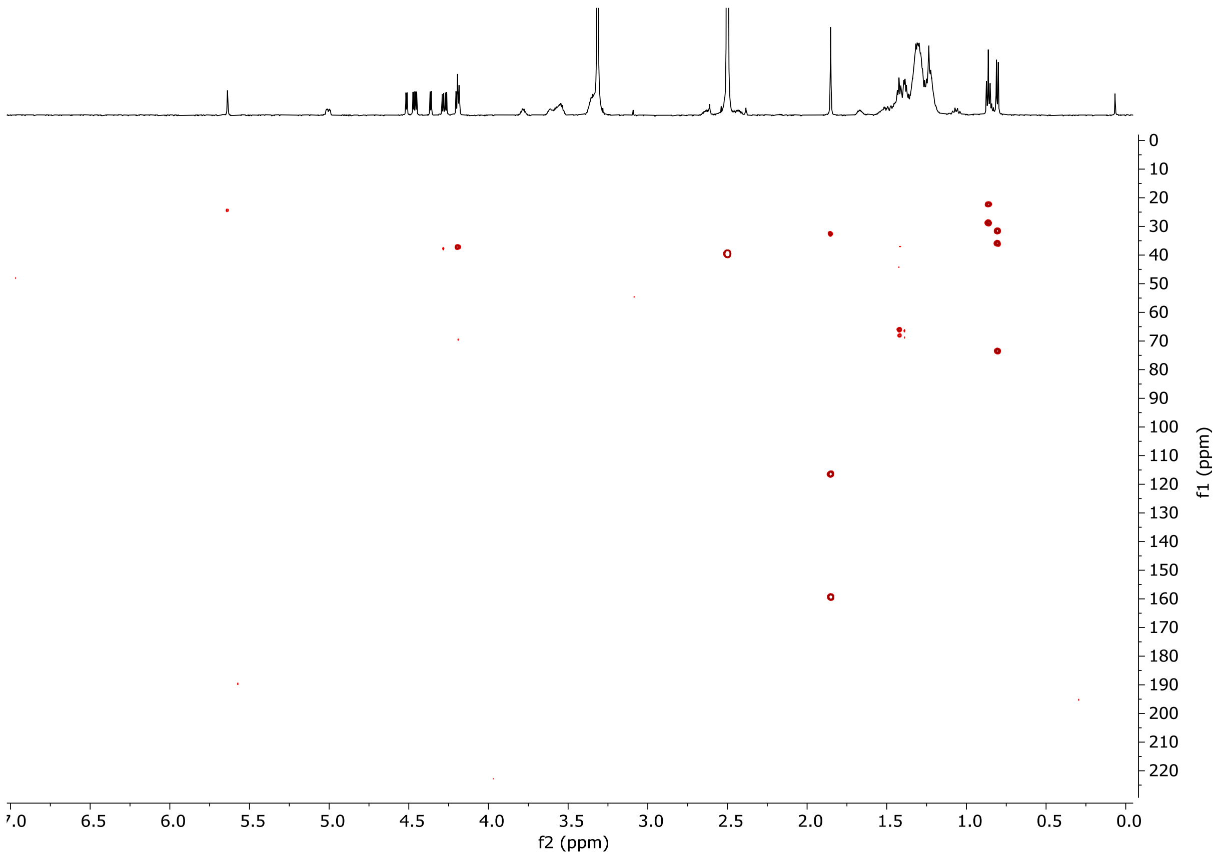

Figure S30: HMBC spectrum (600 MHz, DMSO-d_6) of caylobolide B (**1**).

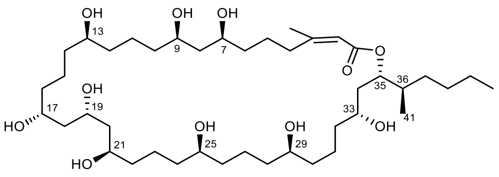

Table S5: ^{1}H and ^{13}C NMR analysis of caylobolide B in DMSO-d_6.

Position	δ_C, type	δ_H (*J* in Hz)	H-H COSY	HMBC
**1**	Unassigned, C			
**2**	116.1, CH	5.64, d (1.1)	42	42
**3**	159.5, C			
**4**	32.5, CH_2	a: 2.64<br>b: 2.44	5<br>5	
**5**	23.5, CH_2	a: 1.52<br>b: 1.40	4a, 4b<br>4a, 4b, 6a	
**6**	37.00, CH_2	a: 1.36<br>b: 1.28	5b, 7<br>7	
**7**	68.6, CH	3.58	OH_h, 6, 8	
**8**	44.0, CH_2	1.38	7, 9	
**9**	68.8, CH	3.56	OH_g, 8, 10	
**10**	37.4, CH_2	a: 1.26<br>b:1.24	9, 11a<br>9	
**11**	21.1, CH_2	a: 1.45<br>b: 1.24	10a, 12	
**12**	36.90, CH_2	1.30	11a, 13	
**13**	69.4 CH	3.36	OH_a, 12, 14	
**14**	36.97, CH_2	1.24	13	
**15**	20.5-20.9, CH_2	1.17-1.38		
**16**	37.4, CH_2	a:1.32<br>b:1.28		
**17**	66.4, CH	3.62	OH_c, 16a,18	
**18**	44.0, CH_2	1.39	17, 19	
**19**	65.9, CH	3.79, dq (11.6, 6)	OH_f, 18, 20a, 20b	
**20**	44.3, CH_2	a: 1.42<br>b: 1.39	19, 21<br>19, 21	19, 21
**21**	67.9, CH	3.54	OH_e, 20a, 20b, 22	
**22**	37.09, CH_2	1.32	21	
**23**	20.5-20.9, CH_2	1.17-1.38		
**24**	36.9, CH_2	1.28	25	
**25**	69.5, CH	3.35	OH_b, 24, 26	
**26**	36.5, CH_2	a: 1.32<br>b: 1.22	25	
**27**	20.4-21.5 CH_2	1.20-1.36		
**28**	36.6-37.9, CH_2	1.21-1.32	29<br>29	
**29**	69.5, CH	3.35	28, 30	
**30**	36.6-37.9, CH_2	1.21-1.32	29<br>29	
**31**	20.8, CH_2	a: 1.46<br>b: 1.33	32a ,32b	
**32**	37.4, CH_2	a: 1.30<br>b: 1.28	31, 33<br>31, 33	
**33**	66.5, CH	3.32	OH_d, 34, 32	
**34**	37.1, CH_2	a: 1.48<br>b: 1.40	33, 35<br>33, 35	
**35**	73.5, CH	5.01, ddd (11.8, 4.15 2.2)	34a,34b, 36	
**36**	35.8, CH	1.67, m	35, 37a, 37b, 41	
**37**	31.4, CH_2	a: 1.31<br>b:1.07	36, 38<br>36, 38	
**38**	28.7, CH_2	a: 1.31<br>b: 1.20	37a, 37b<br>37a, 37b	
**39**	22.1, CH_2	a: 1.27<br>b :1.24	40<br>40	

**40**	13.8, CH_3	0.86	39a, 39b	38 39
**41**	14.6, CH_3	0.80	36	35, 36, 37
**42**	24.3, CH_3	1.85, d (1.2)	2	2, 3, 4
**OH_a**	4.18		13	12, 14
**OH_b**	4.19		25	24, 26
**OH_c**	4.27		17	16, 18
**OH_d**	4.28		33	32, 34
**OH_e**	4.37		21	
**OH_f**	4.45		19	18, 20
**OH_g**	4.47		9	8, 10
**OH_h**	4.51		7	6, 8
**OHi**	4.20		29	27, 28

3.5 *Iso*-caylobolide B-OAc NMR Analysis

3.5.1 ^{1}H NMR (DMSO-d6, 600 MHz)

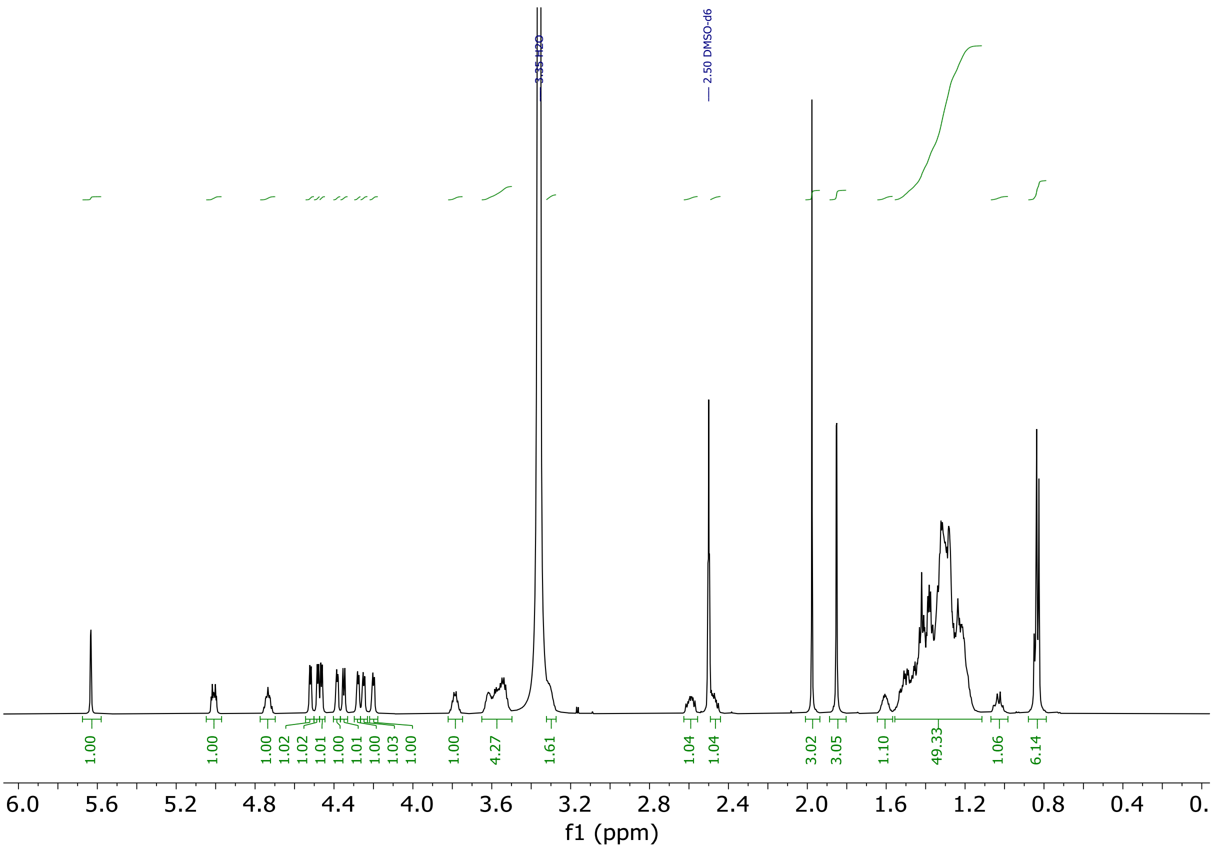

Figure S31: ^{1}H NMR spectrum (600 MHz, DMSO-d_6) of *iso*-caylobolide B-OAc (**4**).

3.5.2 HSQC NMR (DMSO-d6, 600 MHz)

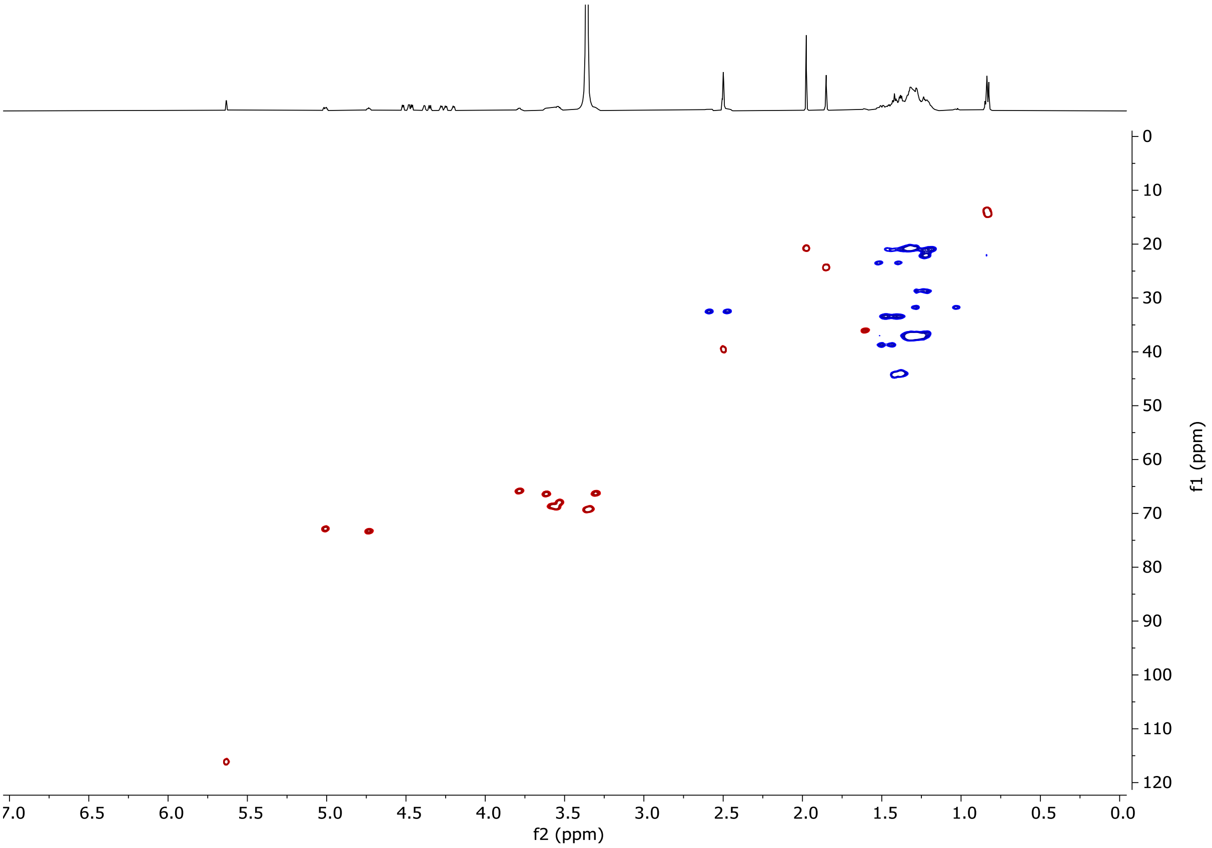

Figure S32: HSQC spectrum (600 MHz, DMSO-d_6) of *iso*-caylobolide B-OAc (**4**).

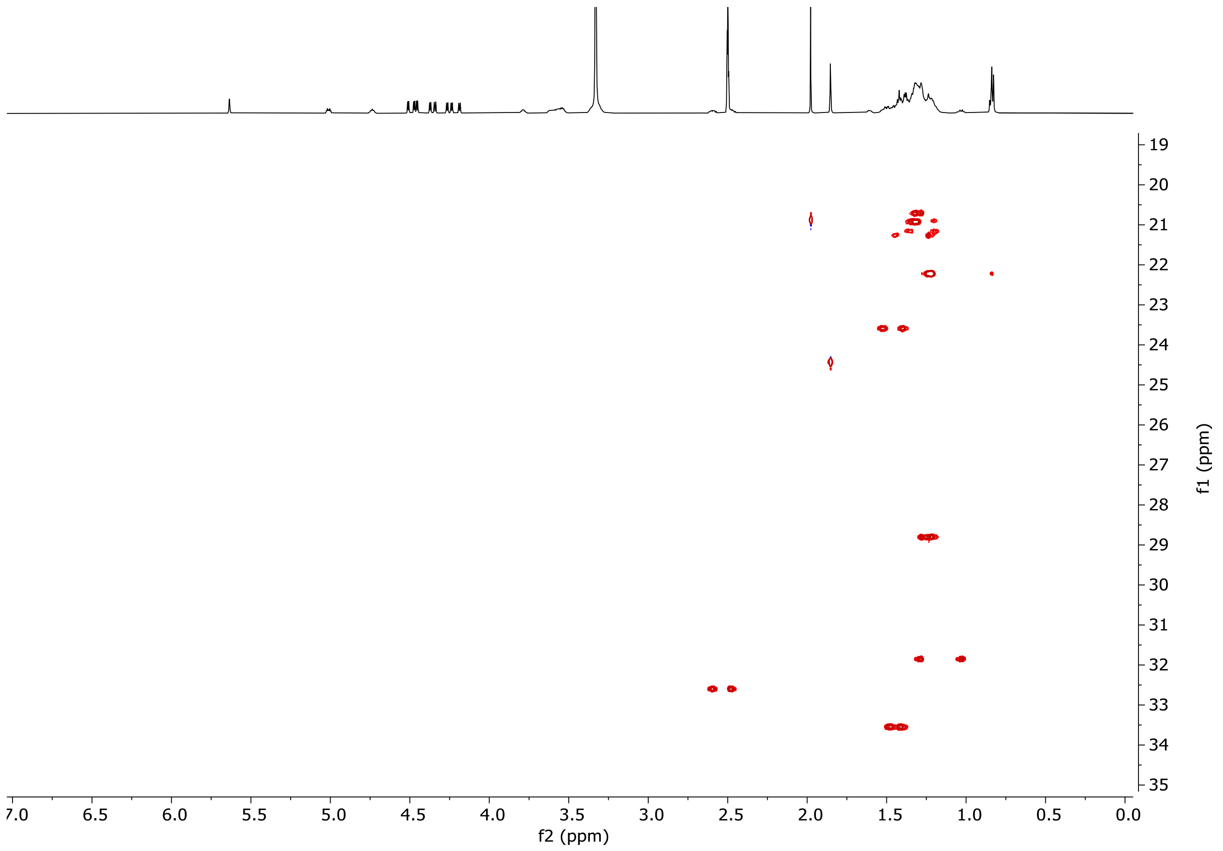

Figure S33: Band-selective HSQC spectrum (600 MHz, DMSO-d_6) of *iso*-caylobolide B-OAc (**4**). Spectrometer frequency (F2: 600.13 MHz, F1: 150.91 MHz), acquired size (t2: 704, t1: 320), spectral width (t2: 4854.4 Hz, t1: 2485.2 Hz), lowest frequency (t2: −631.6 Hz, t1: 2821.3 Hz).

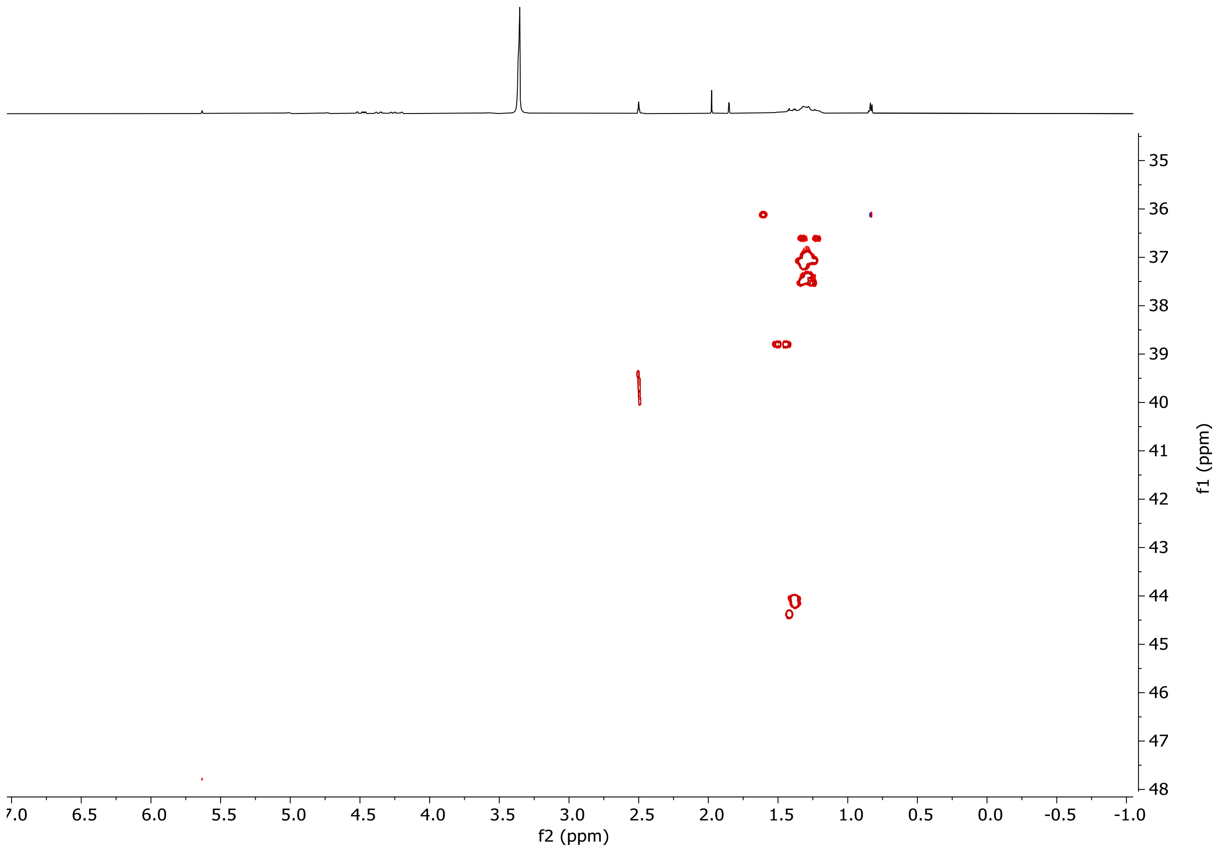

Figure S34: Band-selective HSQC spectrum (600 MHz, DMSO-d_6) of *iso*-caylobolide B-OAc (**4**). Spectrometer frequency (F2: 600.13 MHz, F1: 150.91 MHz), acquired size (t2: 2048, t1: 320), spectral width (t2: 4854.4 Hz, t1: 2066.4 Hz), lowest frequency (t2: −631.6 Hz, t1: 5184.1 Hz).

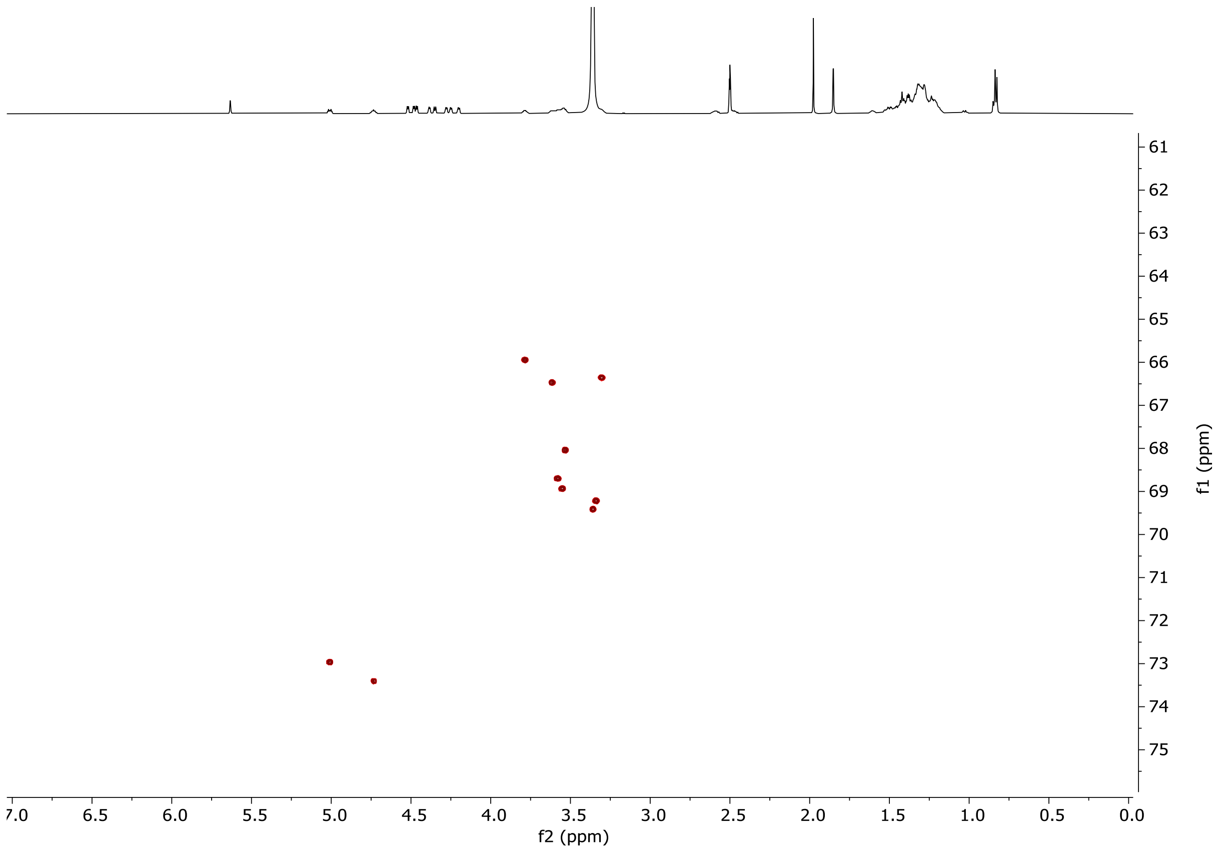

Figure S35: Band-selective HSQC spectrum (600 MHz, DMSO-d_6) of *iso*-caylobolide B-OAc (**4**). Spectrometer frequency (F2: 600.13 MHz, F1: 150.91 MHz), acquired size (t2: 2048, t1: 320), spectral width (t2: 4854.4 Hz, t1: 2320.4 Hz), lowest frequency (t2: −631.6 Hz, t1: 9146.5 Hz).

3.5.3 COSY NMR (DMSO-d6, 600 MHz)

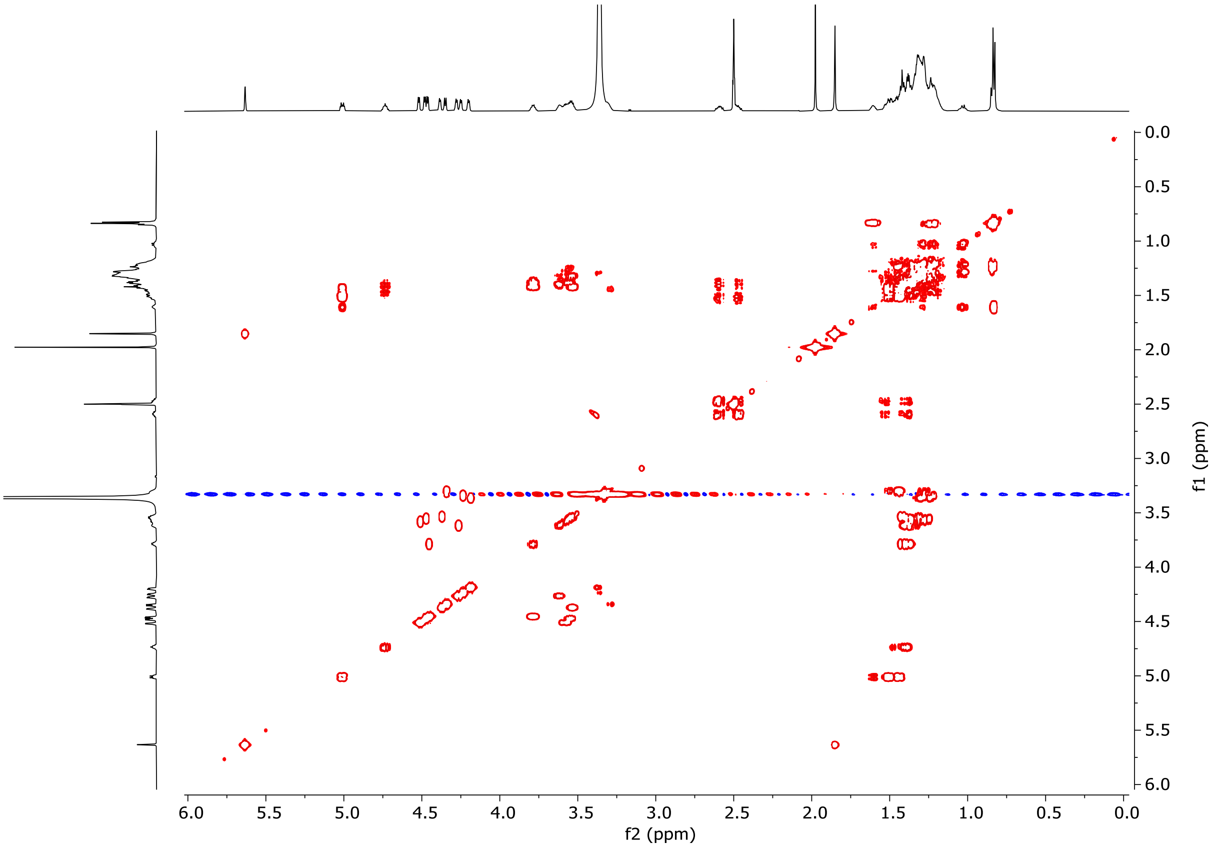

Figure S36: COSY NMR spectrum (600 MHz, DMSO-d_6) of *iso*-caylobolide B-OAc (**4**).

3.5.4 HMBC NMR (DMSO-d6, 600 MHz)

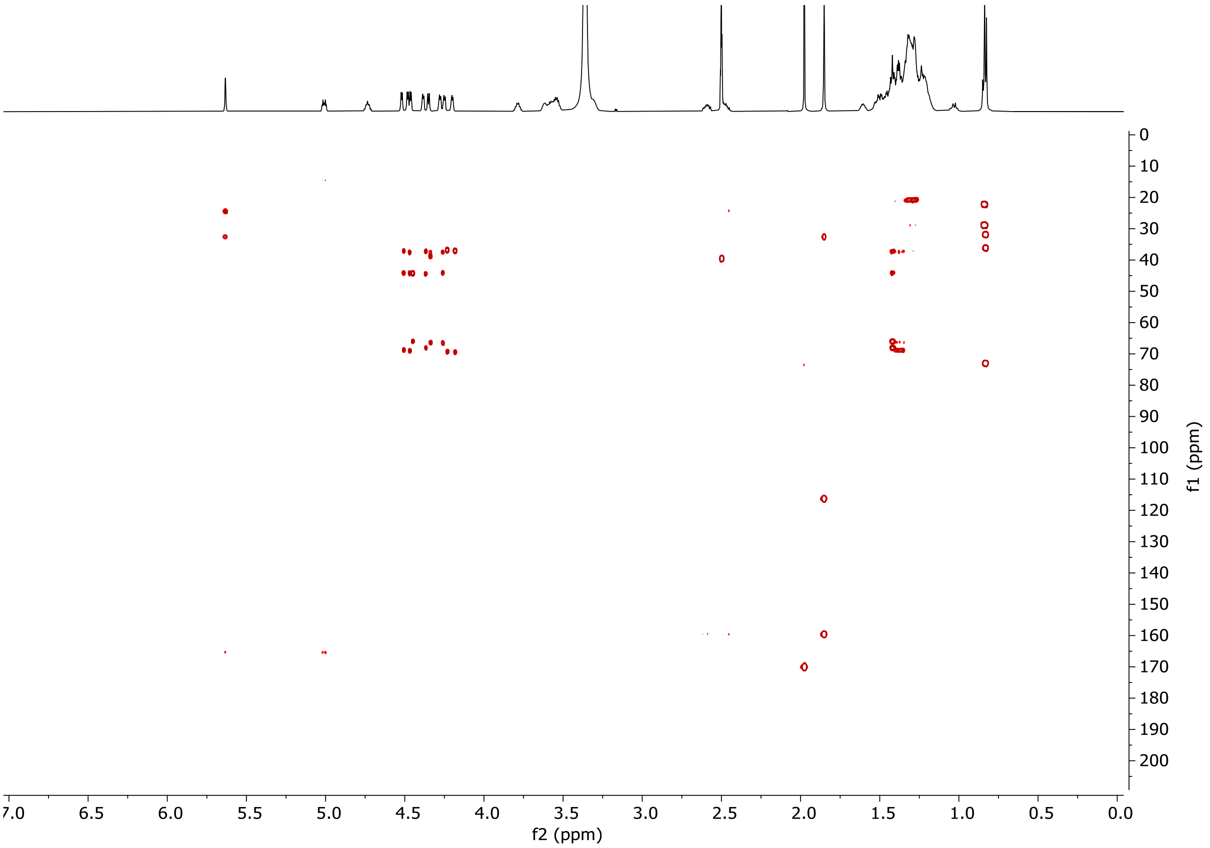

Figure S37: HMBC spectrum (600 MHz, DMSO-d_6) of *iso*-caylobolide B-OAc (**4**).

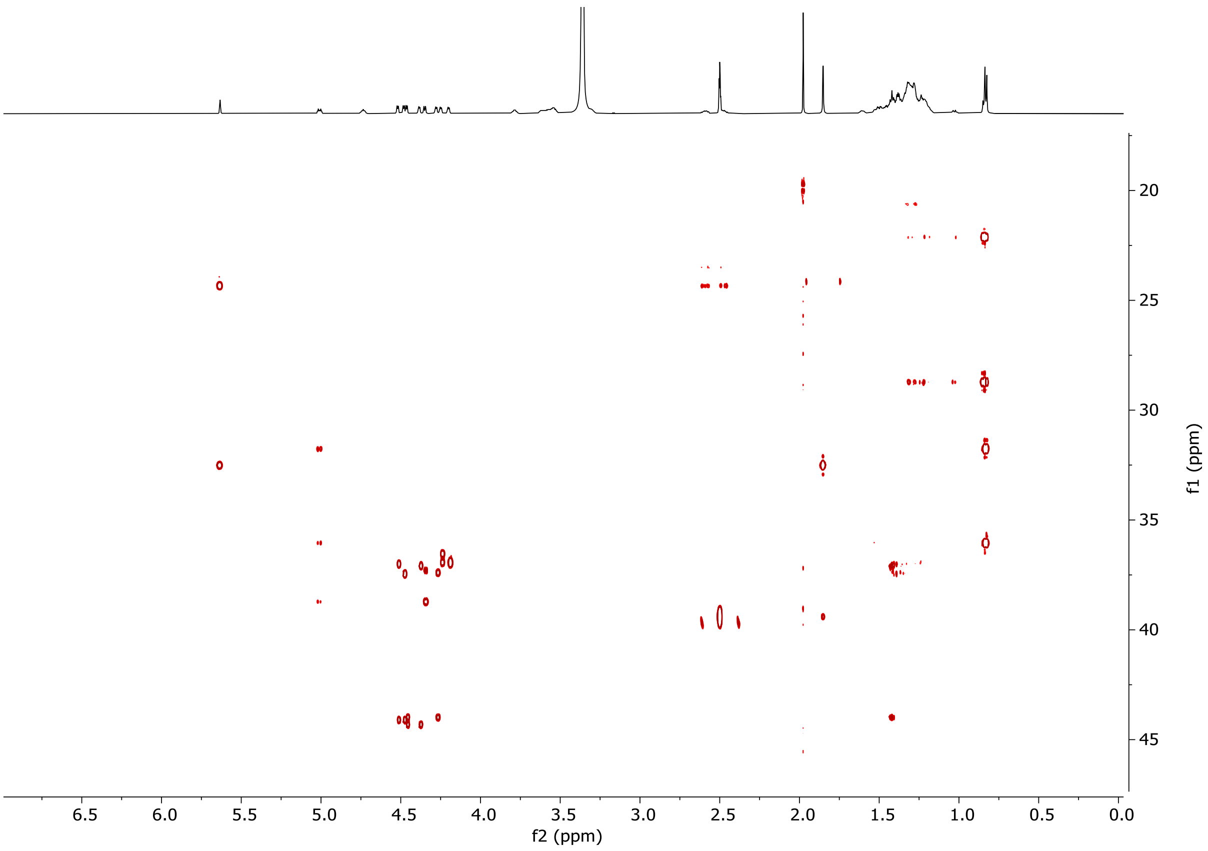

Figure S38: Band-selective HMBC spectrum (600 MHz, DMSO-d_6) of *iso*-caylobolide B-OAc (**4**). Spectrometer frequency (F2: 600.13 MHz, F1: 150.91 MHz), acquired size (t2: 2048, t1: 200), spectral width (t2: 4854.4 Hz, t1: 4532.8 Hz), lowest frequency (t2: −626.8 Hz, t1: 2712.1 Hz).

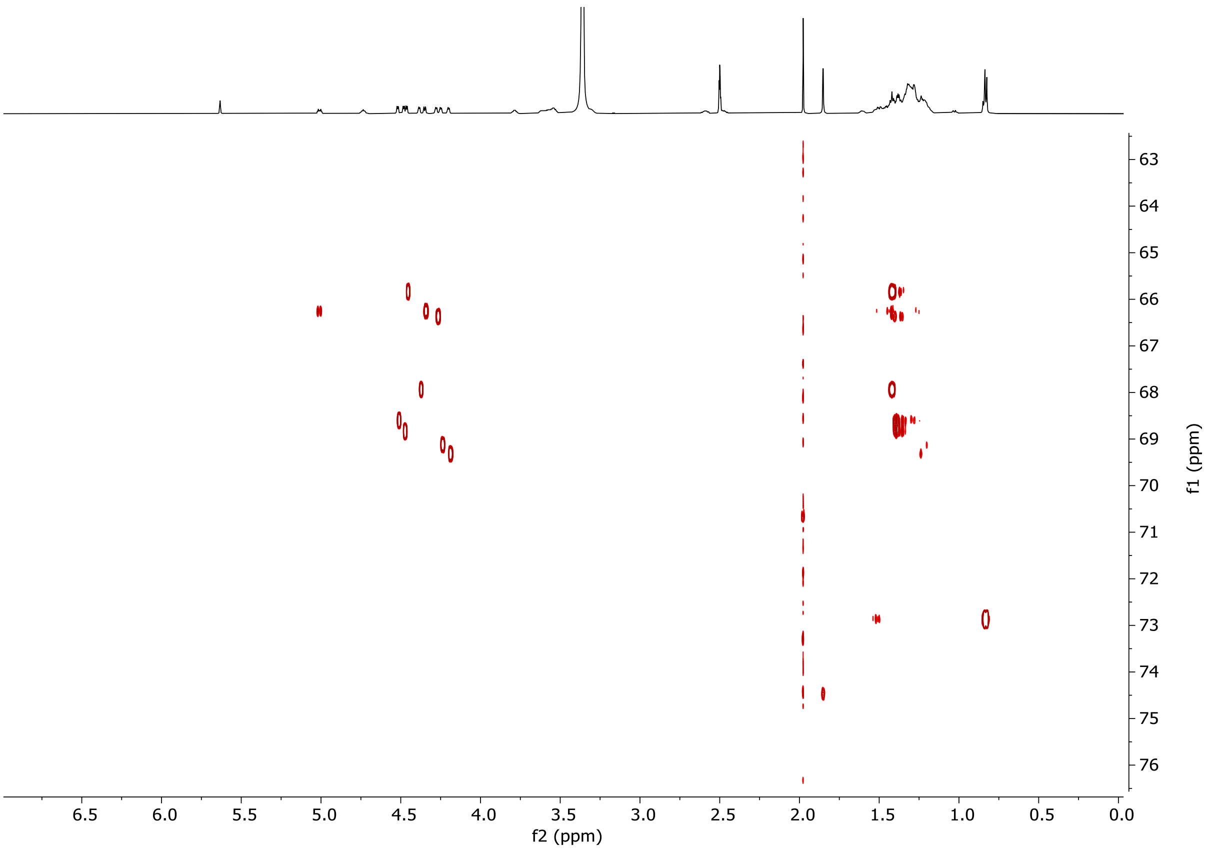

Figure S39: Band-selective HMBC spectrum (600 MHz, DMSO-d_6) of *iso*-caylobolide B-OAc (**4**). Spectrometer frequency (F2: 600.13 MHz, F1: 150.91 MHz), acquired size (t2: 2048, t1: 200), spectral width (t2: 4854.4 Hz, t1: 2106.1 Hz), lowest frequency (t2: −626.8 Hz, t1: 9514.7 Hz).

3.5.5 HSQC-TOCSY NMR (DMSO-d6, 600 MHz)

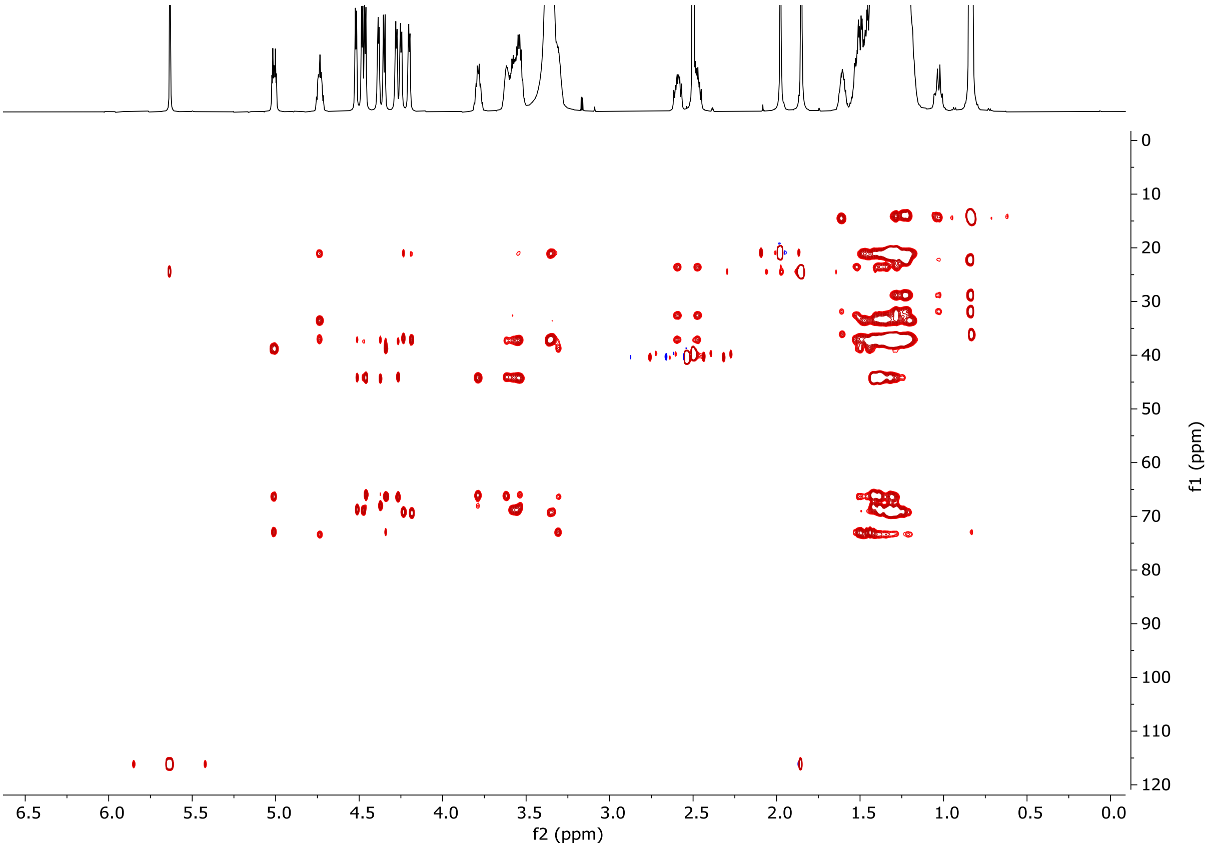

Figure S40: HSQC-TOCSY spectrum (600 MHz, DMSO-d_6) of *iso*-caylobolide B-OAc (**4**).

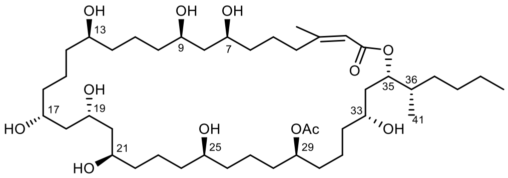

Table S6: ^{1}H and ^{13}C NMR analysis of *iso*-caylobolide B-OAc in DMSO-d_6.

Position	δ_C, type	δ_H (*J* in Hz)	H-H COSY	HMBC	1D selective TOCSY (key correlations)
**1**	165.2, C				
**2**	116.1, CH	5.64, d (1.2)	42	1, 3, 4, 42	
**3**	159.5, C				
**4**	32.5, CH_2	a: 2.60<br>b: 2.48	5<br>5	3, 5, 42<br>3, 5, 42	
**5**	23.5, CH_2	a: 1.53<br>b: 1.41	4a, 4b<br>4a, 4b		
**6**	37.00, CH_2	a:1.36<br>b:1.28	7<br>7		
**7**	68.6, CH	3.58	OH_h, 6, 8		
**8**	44.1, CH_2	1.38	7, 9	6, 7, 9, 10	
**9**	68.8, CH	3.56	OH_g, 8, 10		
**10**	37.5, CH_2	a: 1.26<br>b:1.24	9<br>9		
**11**	21.2, CH_2	a: 1.45<br>b: 1.24			
**12**	36.90, CH_2	1.30	13		
**13**	69.3 CH	3.36	OH_a, 12, 14		
**14**	37.01, CH_2	1.24	13	13	
**15**	20.5-20.9, CH_2	1.20-1.39			
**16**	37.4, CH_2	a:1.32<br>b:1.28			
**17**	66.4, CH	3.62	OH_c, 18		
**18**	43.9, CH_2	1.39	17, 19		
**19**	65.9, CH	3.79, dq (11.6, 6)	OH_f, 18, 20a, 20b	17	
**20**	44.3, CH_2	a: 1.42<br>b: 1.39	19, 21<br>19, 21	18, 22	
**21**	67.9, CH	3.54	OH_e, 20a, 20b, 22		
**22**	37.10, CH_2	1.32	21		
**23**	20.5-20.9, CH_2	1.20-1.39			
**24**	36.95, CH_2	1.28	25		
**25**	69.2 CH	3.35	OH_b, 24, 26b		
**26**	36.5, CH_2	a:1.33<br>b:1.23	 25		
**27**	21.0, CH_2	a:1.36<br>b:1.21			
**28**	33.4, CH_2	a: 1.49<br>b: 1.42	29<br>29		
**29**	73.3, CH	4.74, tt (7, 5)	28, 30		25, 28, 30, 33
**30**	33.4, CH_2	a:1.44<br>b: 1.39	29<br>29		
**31**	20.8, CH_2	a:1.46<br>b:1.33	32b<br>32a, 32b		
**32**	37.3, CH_2	a:1.30<br>b:1.28	31b, 33<br>31a, 31b, 33		
**33**	66.3, CH	3.31	OH_d, 34a, 34b, 32		
**34**	38.7, CH_2	a: 1.51<br>b: 1.45	33, 35<br>33, 35		
**35**	72.8, CH	5.01, dt (9.2, 3.5)	34a,34b, 36	1, 33, 34, 36, 37	
**36**	36.0, CH	1.61, m	35, 37, 41		

**37**	31.8, CH_2	a: 1.30<br>b:1.03	36, 38<br>36, 38	36, 38, 39	
**38**	28.7, CH_2	a: 1.28<br>b: 1.22	37a, 37b<br>37a, 37b		
**39**	22.1, CH_2	a: 1.25<br>b :1.22	40<br>40		
**40**	13.7, CH_3	0.84	39a, 39b	38 39	
**41**	14.4, CH_3	0.83	36	35, 36, 37	
**42**	24.3, CH_3	1.85, d (1.2)	2	2, 3, 4	
**43**	169.9, C				
**44**	20.8, CH_3	1.98		43	
**OH_a**	4.18		13	12, 13, 14	12, 14
**OH_b**	4.23		25	24, 25, 26	22, 24, 28
**OH_c**	4.26		17	16, 17, 18	14, 18
**OH_d**	4.34		33	32, 33, 34	30
**OH_e**	4.37		21	20, 21, 22	20, 22, 24
**OH_f**	4.45		19	18, 19, 20	18, 20
**OH_g**	4.47		9	8, 9, 10	12
**OH_h**	4.51		7	6, 7, 8	4

3.6 Caylobolide B-OAc NMR Analysis

3.6.1 ^{1}H NMR (DMSO-d6, 600 MHz)

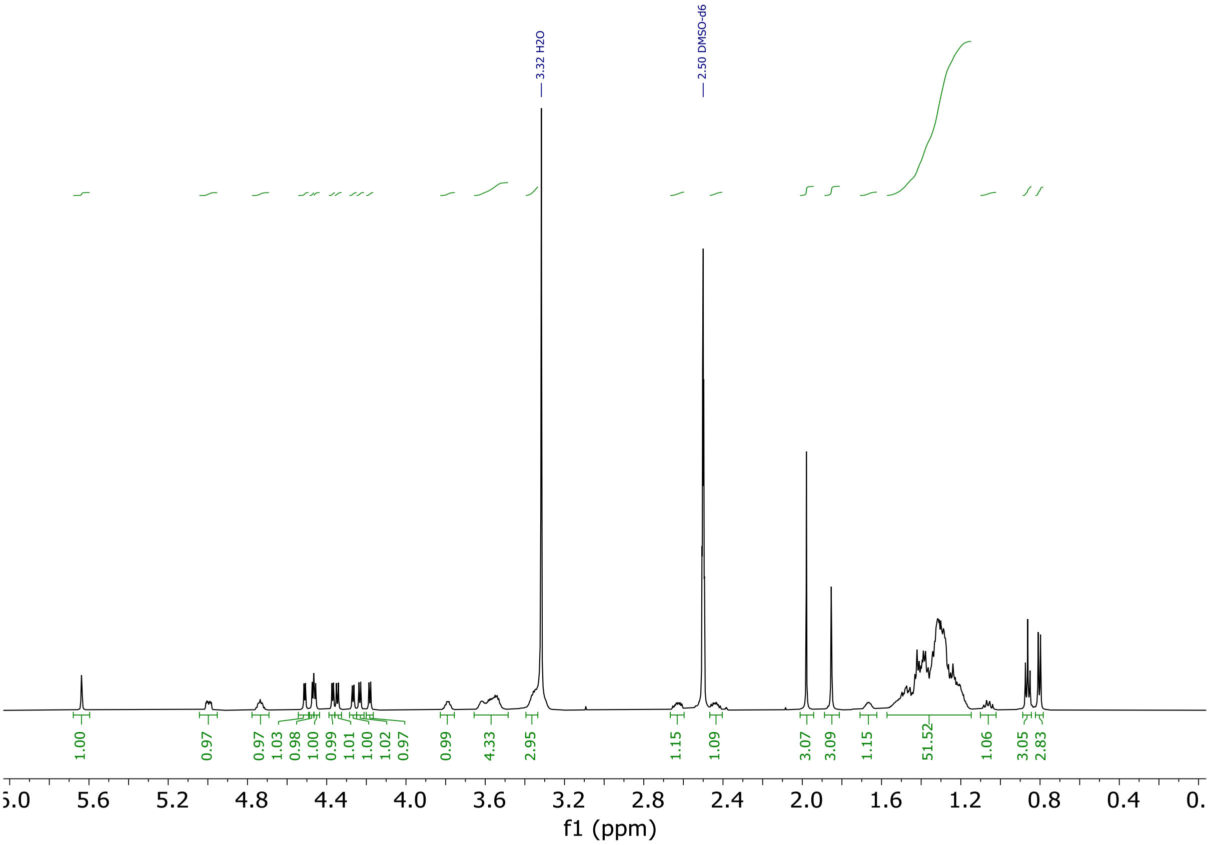

Figure S41: ^{1}H NMR spectrum (600 MHz, DMSO-d_6) of caylobolide B-OAc (**3**).

3.6.2 HSQC NMR (DMSO-d6, 600 MHz)

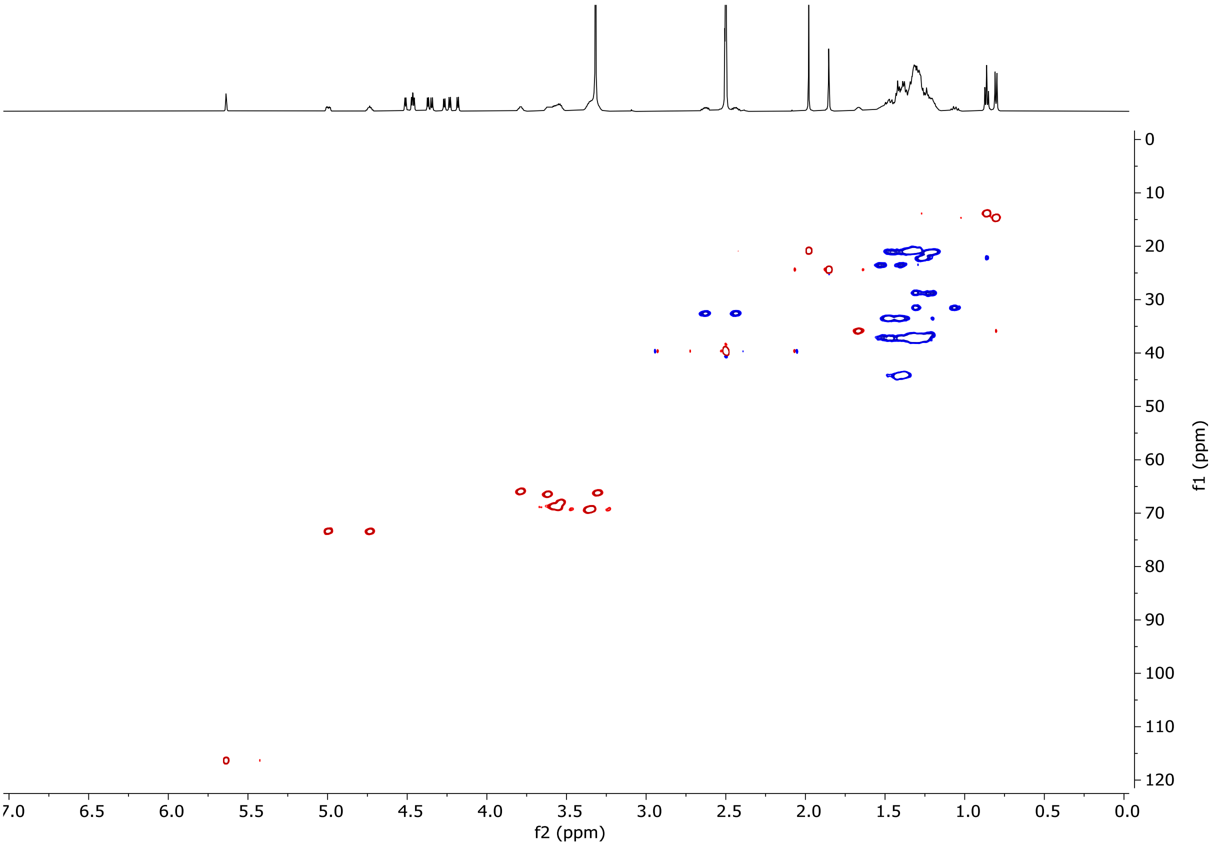

Figure S42: HSQC NMR spectrum (600 MHz, DMSO-d$_6$) of caylobolide B-OAc (**3**).

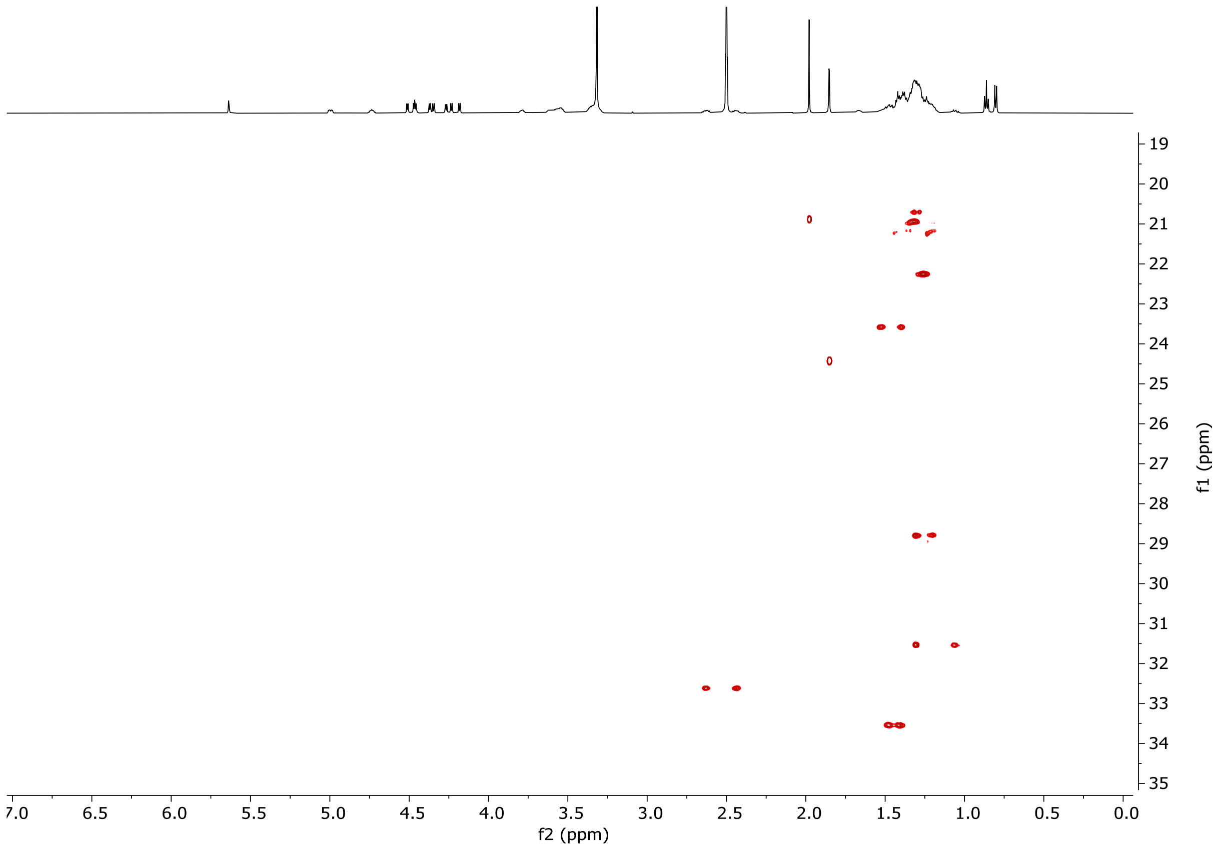

Figure S43: Band-selective HSQC spectrum (600 MHz, DMSO-d_6) of caylobolide B-OAc (**3**). Spectrometer frequency (F2: 600.13 MHz, F1: 150.91 MHz), acquired size (t2: 704, t1: 320), spectral width (t2: 4854.4 Hz, t1: 2485.2 Hz), lowest frequency (t2: −631.6 Hz, t1: 2821.3 Hz).

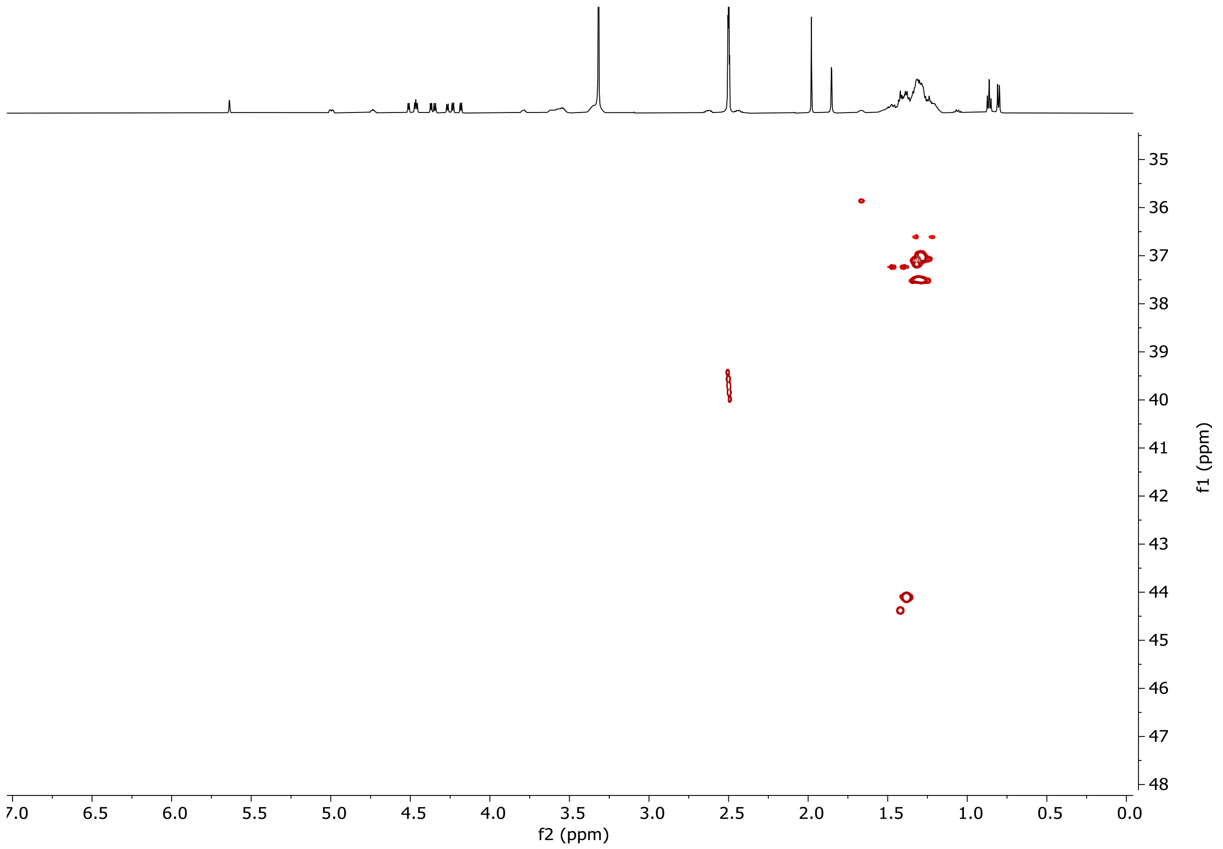

Figure S44: Band-selective HSQC spectrum (600 MHz, DMSO-d_6) of caylobolide B-OAc (**3**). Spectrometer frequency (F2: 600.13 MHz, F1: 150.91 MHz), acquired size (t2: 2048, t1: 320), spectral width (t2: 4854.4 Hz, t1: 2066.4 Hz), lowest frequency (t2: −631.6 Hz, t1: 5184.1 Hz).

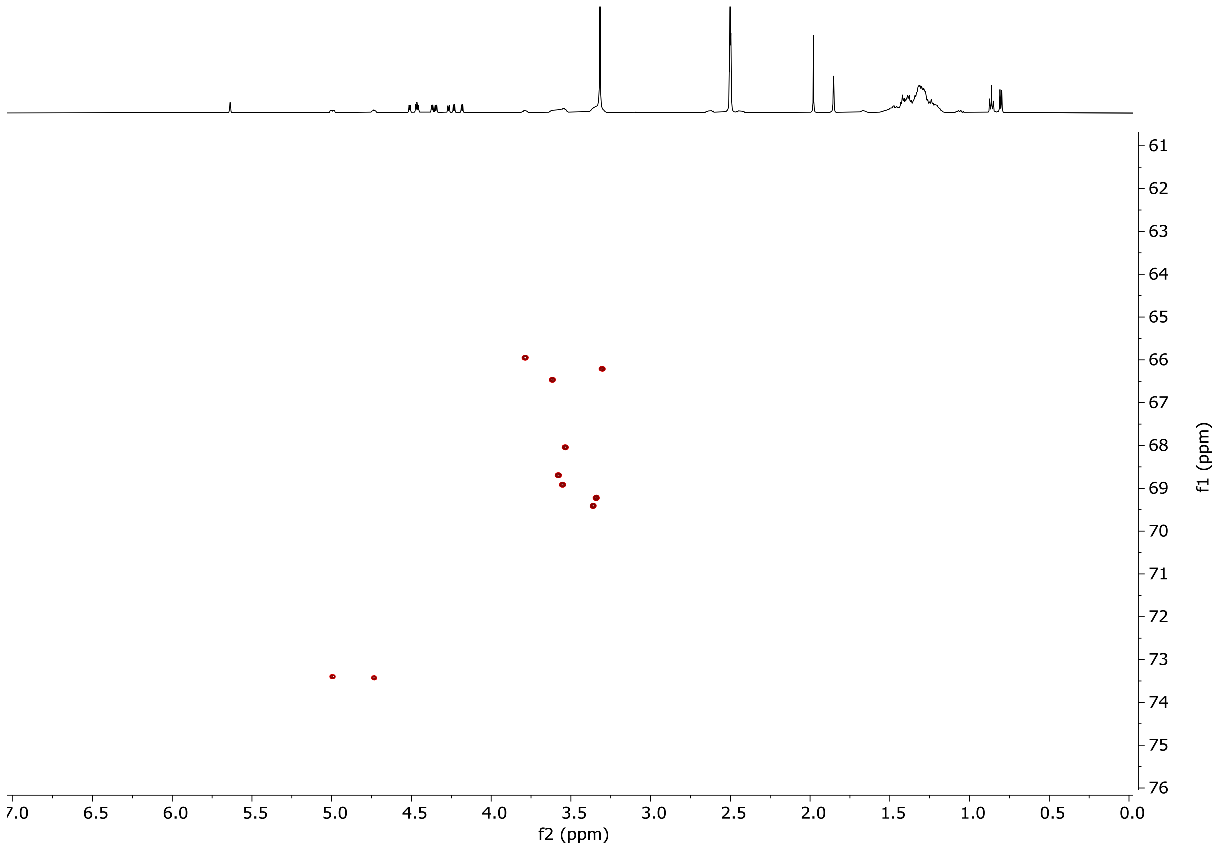

Figure S45: Band-selective HSQC spectrum (600 MHz, DMSO-d_6) of caylobolide B-OAc (**3**). Spectrometer frequency (F2: 600.13 MHz, F1: 150.91 MHz), acquired size (t2: 2048, t1: 320), spectral width (t2: 4854.4 Hz, t1: 2320.4 Hz), lowest frequency (t2: −631.6 Hz, t1: 9146.5 Hz).

3.6.3 COSY NMR (DMSO-d6, 600 MHz)

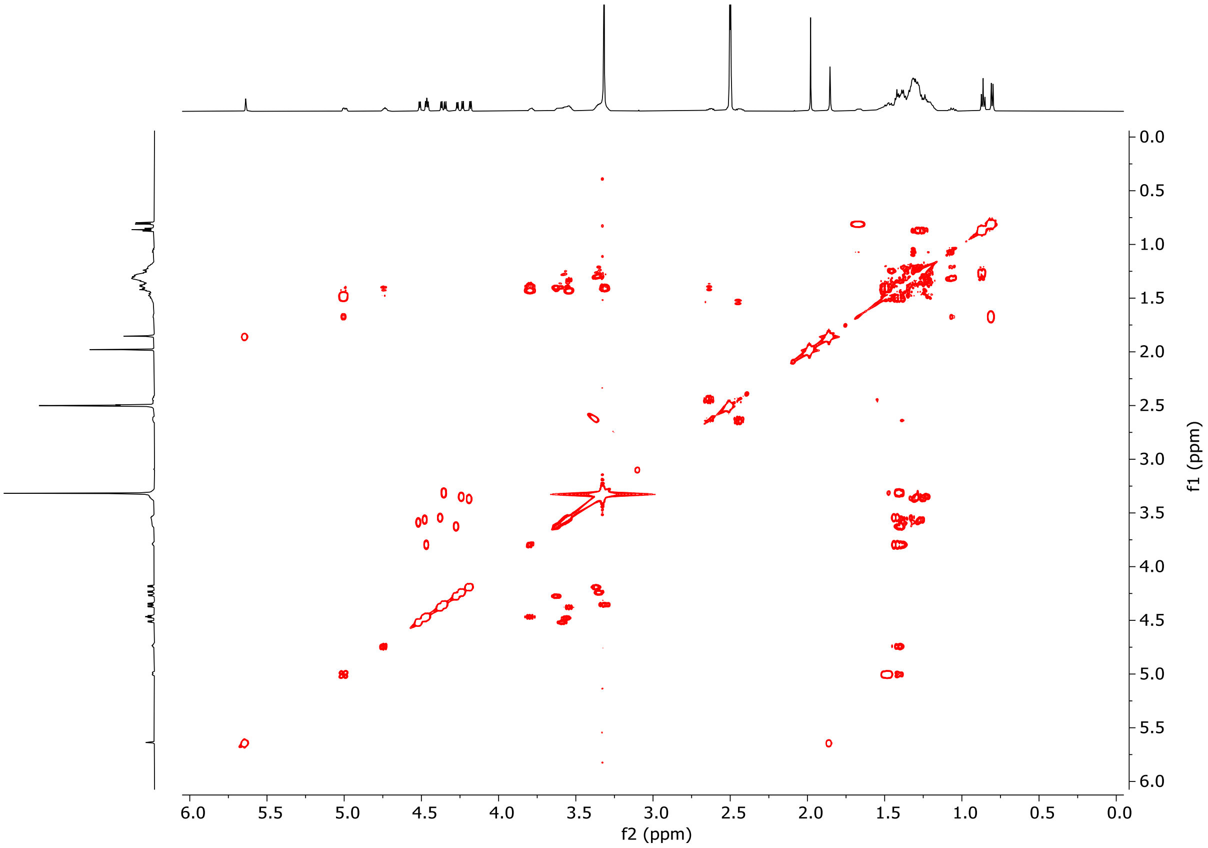

Figure S46: COSY spectrum (600 MHz, DMSO-d_6) of caylobolide B-OAc (**3**).

3.6.4 HMBC NMR (DMSO-d6, 600 MHz)

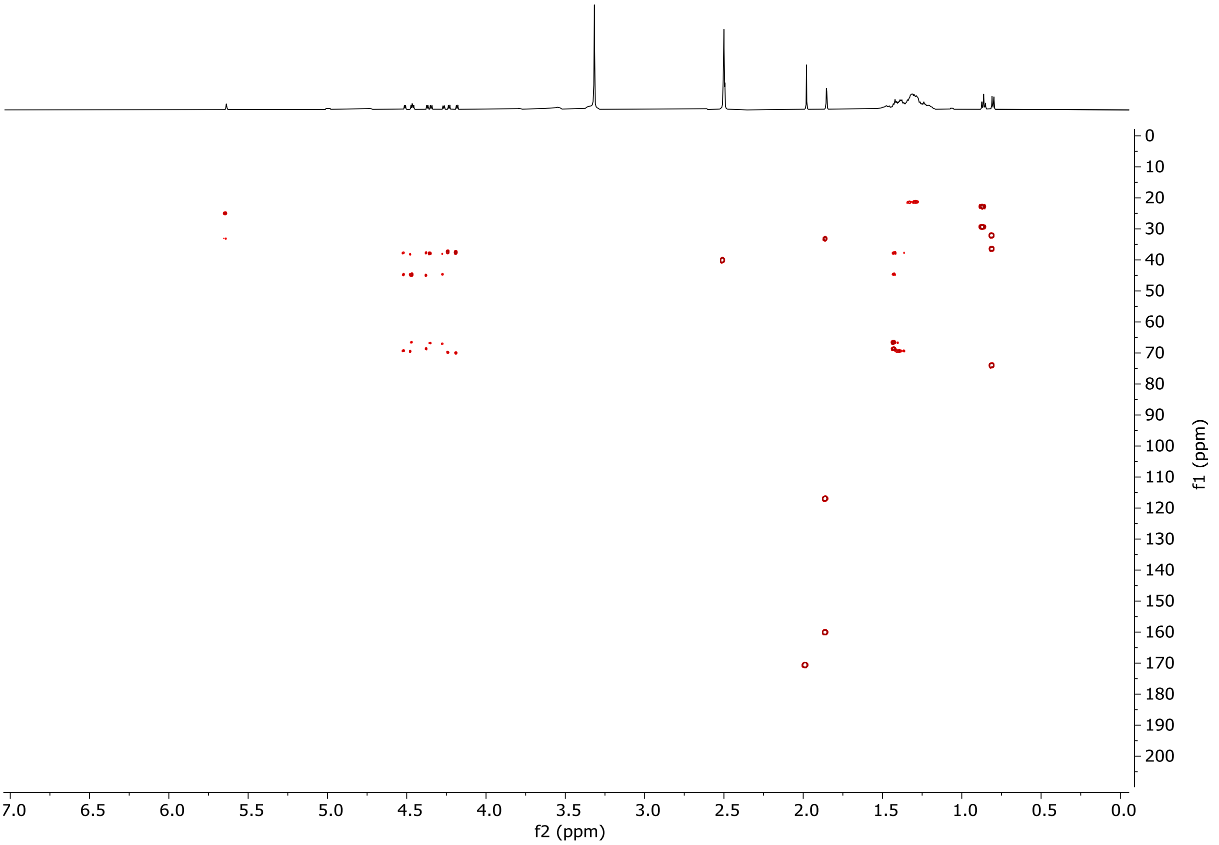

Figure S47: HMBC spectrum (600 MHz, DMSO-d_6) of caylobolide B-OAc (**3**).

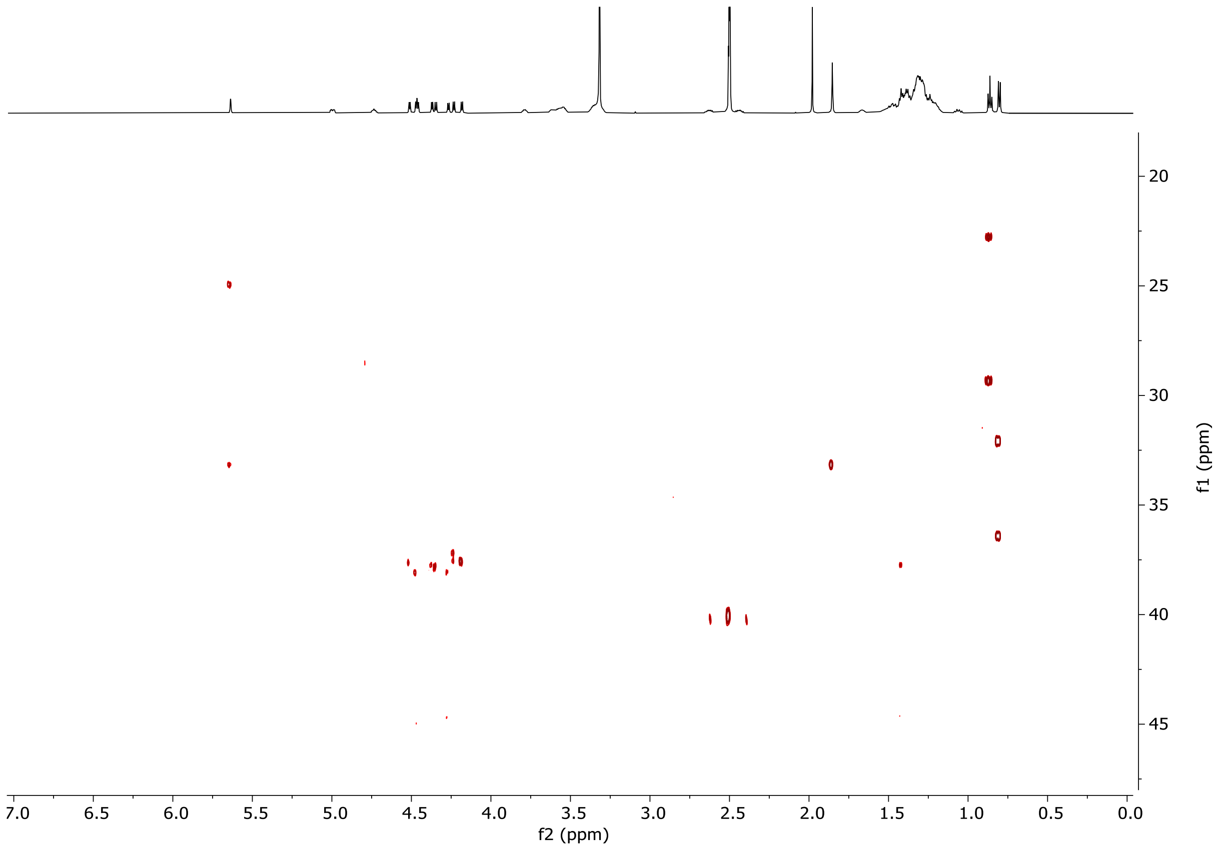

Figure S48: Band-selective HMBC spectrum (600 MHz, DMSO-d_6) of caylobolide B-OAc (**3**). Spectrometer frequency (F2: 599.48 MHz, F1: 150.74 MHz), acquired size (t2: 2047, t1: 256), spectral width (t2: 4795.4 Hz, t1: 4524.9 Hz), lowest frequency (t2: −626.8 Hz, t1: 2712.1 Hz).

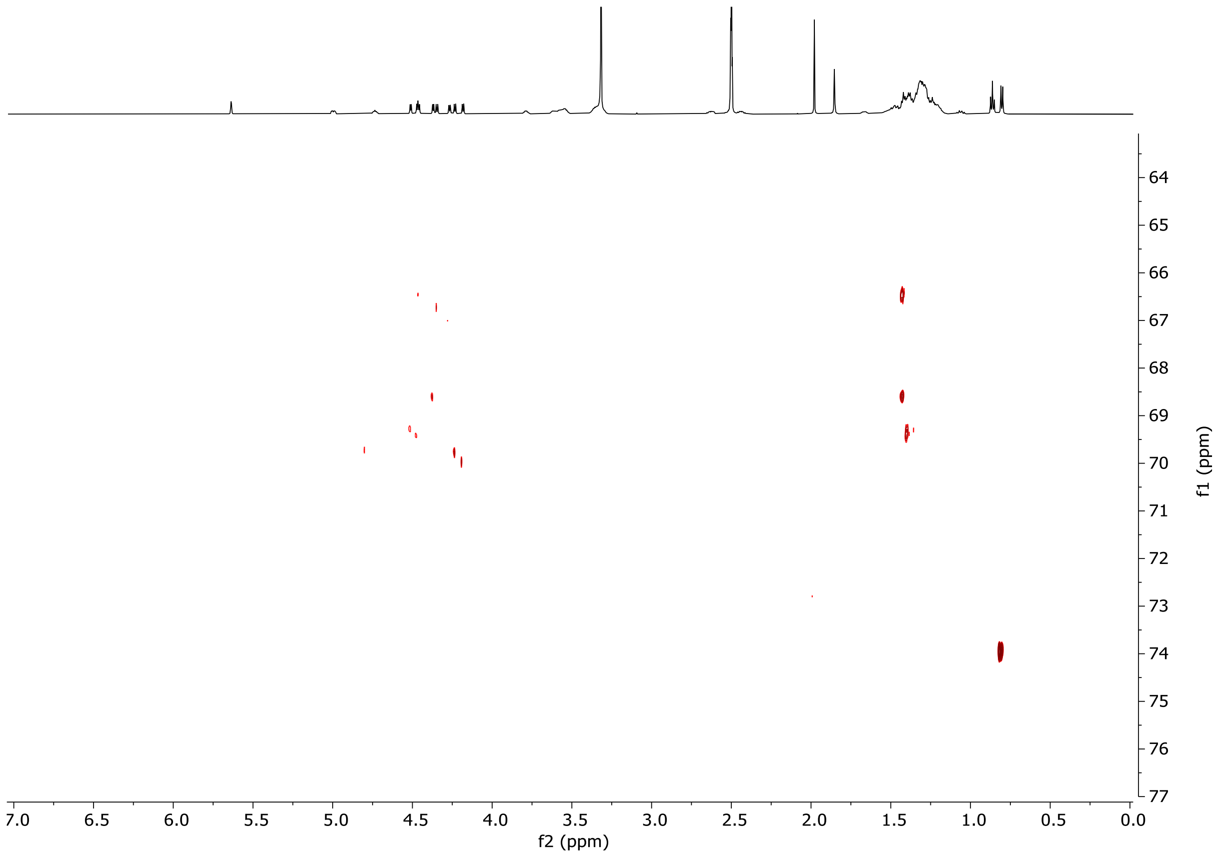

Figure S49: Band selective HMBC spectrum (600 MHz, DMSO-d_6) of caylobolide B-OAc (**3**).

3.6.5 HSQC-TOCSY NMR (DMSO-d6, 600 MHz)

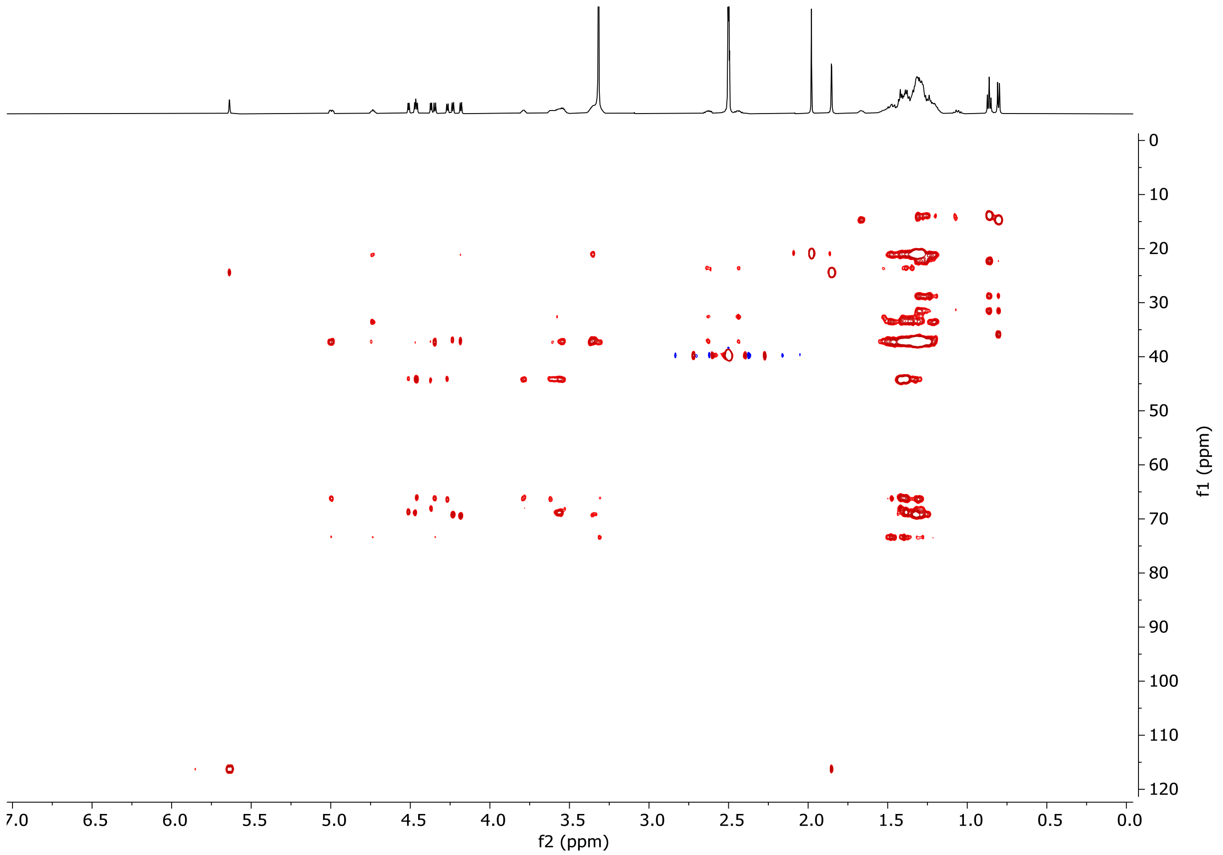

Figure S50: HSQC-TOCSY spectrum (600 MHz, DMSO-d_6) of caylobolide B-OAc (**3**).

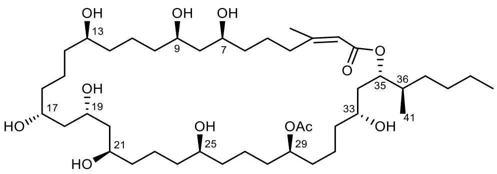

Table S7: ^{1}H and ^{13}C NMR analysis of caylobolide B-OAc in DMSO-d_6.

Position	δ_C, type	δ_H (J in Hz)	H-H COSY	HMBC	1D selective TOCSY (key correlations)
**1**	165.2, C				
**2**	116.1, CH	5.64, d (1.2)	42	1, 3, 4, 42	
**3**	159.5, C				
**4**	32.5, CH_2	a: 2.63	5		
		b: 2.44	5	3	
**5**	23.5, CH_2	a: 1.52	4a, 4b		
		b: 1.40	4a, 4b		
**6**	37.0, CH_2	a: 1.36	7	7	
		b: 1.28	7		
**7**	68.6, CH	3.58	OH_h, 6, 8		
**8**	44.0, CH_2	1.38	7, 9		
**9**	68.8, CH	3.56	OH_g, 8, 10		
**10**	37.4, CH_2	a: 1.26	9		
		b:1.24	9		
**11**	21.1, CH_2	a: 1.45			
		b: 1.24			
**12**	36.90, CH_2	1.30	13		
**13**	69.3 CH	3.36	OH_a, 12, 14		
**14**	36.97, CH_2	1.24	13		
**15**	20.5-20.9, CH_2	1.17-1.38			
**16**	37.39, CH_2	1.32			
		1.28			
**17**	66.4, CH	3.62	OH_c, 18		
**18**	44.0, CH_2	1.38	17, 19		
**19**	65.9, CH	3.79, dq (11.6, 6)	OH_f, 18, 20a, 20b	17	
**20**	44.3, CH_2	a: 1.42	19, 21	18, 22	
		b: 1.39	19, 21		
**21**	67.9, CH	3.54	OH_e, 20a, 20b, 22		
**22**	37.09, CH_2	1.32	21		
**23**	20.5-20.9, CH_2	1.17-1.38			
**24**	36.95, CH_2	1.28	25		
**25**	69.2, CH	3.35	OH_b, 24, 26		
**26**	36.5, CH_2	a: 1.32			
		b: 1.22	25		
**27**	21.0 CH_2	a: 1.36			
		b: 1.20			
**28**	33.4, CH_2	a: 1.48	29		
		b: 1.41	29		
**29**	73.4, CH	4.74, tt (7, 5)	28, 30		25, 28, 30, 33
**30**	33.4, CH_2	a:1.44	29		
		b: 1.39	29		
**31**	20.8, CH_2	a: 1.46			
		b: 1.33	32a ,32b		
**32**	37.37, CH_2	a: 1.30	31, 33		
		b: 1.28	31, 33		
**33**	66.1, CH	3.31	OH_d, 34a, 34b, 32		
**34**	37.15, CH_2	a: 1.48	33, 35		
		b: 1.40	33, 35		
**35**	73.3, CH	5.00, ddd (11.8, 4.15 2.2)	34a,34b, 36		
**36**	35.8, CH	1.67, m	35, 37a, 37b, 41		
**37**	31.4, CH_2	a: 1.31	36, 38		
		b:1.06	36, 38		

**38**	28.7, CH_2	a: 1.31<br>b: 1.20	37a, 37b<br>37a, 37b		
**39**	22.1, CH_2	a: 1.27<br>b :1.24	40<br>40		
**40**	13.8, CH_3	0.86	39a, 39b	38 39	
**41**	14.6, CH_3	0.80	36	35, 36, 37	
**42**	24.3, CH_3	1.85, d (1.2)	2	2, 3, 4	
**43**	169.9, C				
**44**	20. 8, CH_3	1.98		43	
**OH_a**	4.18		13	12, 13, 14	12, 14
**OH_b**	4.23		25	24, 25, 26	22, 24, 28
**OH_c**	4.27		17	16, 17, 18	14, 18
**OH_d**	4.35		33	32, 33, 34	30
**OH_e**	4.37		21	20, 21, 22	20, 22, 24
**OH_f**	4.46		19	18, 19, 20	18, 20
**OH_g**	4.47		9	8, 9, 10	12
**OH_h**	4.51		7	6, 7, 8	4

3.4 Bioactivity and SAR of Caylobolide B Analogues

3.4.1 MTT Cell Viability Assay

HCT116 cells were cultured and maintained in Dulbecco's modified Eagle medium (DMEM, Invitrogen) supplemented with 10% fetal bovine serum (FBS; HyClone, Logan, UT) and 1% antibiotic-antimycotic (Invitrogen) at 37 °C in a humidified atmosphere with 5% CO_2. HCT116 cells were seeded in 96-well plates at densities of 9000 cells per well in 100 µL, respectively. After 16 h of incubation, the cells were treated with various concentrations of compounds or a solvent control (DMSO, 0.5% final concentration). Following 48 h of incubation, cells were treated with 3-(4,5-dimethylthiazol-2-yl)-2,5-diphenyltetrazolium bromide according to the manufacturer's instructions (Promega). The experiment was done as technical triplicates.

3.4.2 S. Cerevisiae Growth Inhibition Assay

S. cerevisiae cells (Y7092) were grown to logarithmic phase in Yeast–peptone–dextrose (YPD). The YPD medium is composed of 2 % glucose (Sigma-Aldrich), 1 % yeast extract (Fisher BioReagents) and 2 % bacto peptone (Fisher BioReagents). Cells were diluted to 5 x 10^4 cells/mL in media, then 100 µL was added to each well of a 96 well plate. Compounds were added in 1 µL of DMSO and plates incubated for 24 h at 30 °C before measuring the OD_{600} on the SpectraMAX M5 plate reader with 1 % DMSO serving as the solvent control. The experiment was done as technical triplicates.

3.4.3 Chemogenomic Homologous Deletion Profiling (HOP) Assay

A deletion library pool containing 4,300 haploid *pdr1Δpdr3ΔxxxΔ* strains (generously provided by Dr. Maya Schuldiner, Weizmann Institute of Science) was screened at concentrations of compounds that inhibited growth by 20%. This was accomplished through

a growth inhibition assay to establish the minimum inhibitory concentration (MIC) using 96-well plates. A series of concentrations around the MIC was selected for further testing in 24-well plates, conducted in two stages at a culture density of 0.03125. In the first stage, cultures were incubated with the compounds or 1% DMSO for 12 hours at 30 °C with shaking. The growth was then measured using a SpectraMax M5 plate reader at OD600, and the cultures were re-inoculated at the same density as the initial phase. After an additional 10-hour incubation, final growth measurements were taken, and wells exhibiting approximately 20% growth inhibition were selected for further analysis. Cells were harvested, and genomic DNA was extracted following the GeneJet DNA purification protocol. Two PCR reactions were performed, as outlined by the Gresham lab. The first reaction amplified the UPTAG and DNTAG (barcodes), while the second incorporated the P5 adapter sequence necessary for Illumina sequencing. The library was quantified using the PicoGreen kit (Invitrogen) and diluted to 50 nM. It was then submitted to the UF ICBR NextGen DNA Sequencing core facility, where quality control was conducted using the Qubit 3.0 Fluorimeter for quantification and the Agilent 2200 TapeStation System for assessing the size and integrity of the library. The pooled library underwent further evaluation through qPCR, allowing for precise quantification of the library based on the presence of the adapter sequence. Finally, the library was sequenced on the MiSeq platform using a 1 × 61 cycle protocol with the 1 × 150 V3 kit.

3.4.4 Bioinformatic analysis of HOP assay

The bioinformatic analysis of the HOP assay involved several key steps to identify and quantify barcodes. Initially, sequence reads were demultiplexed using the fastx_toolkit, allowing for one mismatch in the sample index. Subsequently, the UPTAG and DNTAG F-PRIMER sequences were identified with the AGREP command in Linux, permitting two mismatches. Barcode sequences were extracted from reads positioned between 21 and 40, following the identified primers. These barcodes were then aligned to a reference pool that included UP Barcode 1-3, DN Barcode 1-3, and all revised barcode sequences, allowing for two mismatches using blastn. The read counts from multiple original barcodes were aggregated, and average read counts from both revised and original barcodes were used for further analysis. Barcodes with read counts exceeding 10 in at least one sample were retained for differential analysis. Finally, the exact test in edgeR was employed to perform separate differential analyses for UP and DN barcodes, with differentially expressed ORFs identified using thresholds of FDR < 0.05 and fold change > 2.

3.4.5 Gene Ontology Term Analysis

Genes were annotated based on the Saccharomyces Genome Database (SGD).[9]

Table S8. GO term enrichment (cellular component) of UPtag and DNtag hits sensitive to isocaylobolide B (log_2 fold-change ≤ –1.5; FDR < 0.05), analyzed using the SGD

**Gene Ontology term**	**Cluster frequency**	**Genome frequency**	**Corrected P-value**	**Genes annotated to the term**
Plasma membrane	31 of 275 genes, 11.3%	388 of 7166 genes, 5.4%	0.01113	AST1, RIM8, MID1, CIS3, DFG16, SCS2, SFH5, PFA5, MTL1, SKM1, PTR2, AQY2, LSP1, ARN1, SGE1, MSB3, AZR1, PRM6, MSO1, ZRT1, OSH3, SST2, DCW1, SLM2, BMH1, HXT3, SIP3, AVO2, RHO5, PMA2, GEF1
Cell periphery	45 of 275 genes, 16.4%	683 of 7166 genes, 9.5%	0.02868	BMH1, SEI1, DCW1, YNL190W, SST2, ZRT1, MET5, MSO1, PMA2, FHN1, GEF1, WHI2, DFP3, SKM1, AQY2, MTL1, AIP1, CIS3, RIM8, SFH5, OPI3, DTR1, MSB3, DPL1, SLM2, OSH3, RVS167, PRM6, RHO5, SPI1, SIP3, AVO2, HXT3, PTR2, PFA5, MID1, AST1, SCS2, DFG16, YSP2, ENT4, AZR1, LSP1, SGE1, ARN1

Table S9. GO term enrichment (biological process) of DNtag hits sensitive to isocaylobolide B (log_2 fold-change ≤ –3; FDR < 0.05), analyzed using the SGD.

**Gene Ontology term**	**Cluster frequency**	**Genome frequency**	**Corrected P-value**	**Genes annotated to the term**
Positive regulation	3 of 88 genes,	7 of 7166 genes,	0.02260	GIC2, AIP5, RHO4

of actin filament bundle assembly	3.4%	0.1%		
Positive regulation of cytoskeleton organization	5 of 88 genes, 5.7%	41 of 7166 genes, 0.6%	0.04928	GIC2, AIP5, TUS1, MYO3, RHO4

Table S10. GO term enrichment (cellular component) of the 304 sensitive mutants common between isocaylobolide B and amantelide A ($\log_2$ fold-change < –1; FDR < 0.05) analyzed using the SGD.

Gene Ontology term	Cluster frequency	Genome frequency	Corrected P-value	Genes annotated to the term
Cell periphery	50 of 304 genes, 16.4%	683 of 7166 genes, 9.5%	0.01197	WSC3, ZRT1, AZR1, FHN1, MET5, APM4, MST27, PFA5, MTL1, PUG1, BMH2, YSP2, SEI1, ARK1, RHO5, APS2, AQY2, ATO2, HXT3, FLO10, PTR2, JSN1, SKM1, OSH3, DUR3, GEF1, CIS3, SGE1, MSB3, DCW1, AIP1, SNQ2, DTR1, DPL1, YNL190W, SPI1, SPS2, CWH43, MSO1, AST1, ENT4, MDM1, SST2, GND2, FAT1, DIA3, AVO2, CCW14, OPI3, LSP1
Plasma membrane	33 of 304 genes, 10.9%	388 of 7166 genes, 5.4%	0.01483	MSB3, DCW1, SGE1, PUG1, BMH2, CIS3, MST27, APM4, MTL1, PFA5, AZR1, DUR3, GEF1, ZRT1, OSH3, SKM1, WSC3, PTR2, HXT3, LSP1, FAT1, ATO2, AVO2, AQY2, SST2, GND2, APS2, AST1, RHO5, MSO1, CWH43, SPS2, SNQ2

4 SYNTHETIC PROCEDURES

General Procedure 1 (GP1) – α-Sulfinylbenzoate Preparation

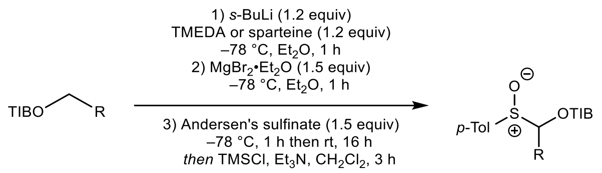

According to a modified literature procedure[10], TMEDA or sparteine (1.20 equiv) was added to a flame dried two-neck round bottomed flask under nitrogen, followed by the TIB ester (1.00 equiv). The mixture was degassed by stirring under vacuum for 30 min, then dry Et_2O (0.30 M *wrt* TIB ester) was added, and the reaction mixture was cooled to −78 °C. To the reaction mixture, *s*-BuLi (1.30 M hexane, 1.20 equiv) was added via syringe pump (1.00 mL/min). The reaction mixture was stirred for 1 h at −78 °C. In a separate flame dried two-neck round bottomed flask under nitrogen with a condenser containing a stirred suspension of Mg turnings (4.00 equiv) in Et_2O (2.00 M), was added dibromoethane (1.50 equiv) dropwise (*CAUTION: exothermic reaction*). After gas evolution ceased, the $MgBr_2 \cdot Et_2O$ solution was stirred for 30 min at room temperature (*formed 2 layers*, *top colourless*, *bottom grey*). The solution was then transferred to the main reaction vessel via syringe pump (1.00 mL/min). The reaction mixture was stirred at −78 °C for 2 h. To this reaction mixture, Andersen’s sulfinate (1.50 equiv) dissolved in THF (1.00 M) was added dropwise with a syringe pump (0.50 mL/min) and the reaction mixture was stirred 1 h at −78 °C, then 16 h at room temperature. The reaction mixture was quenched with 2 M HCl, the layers separated, and the organic layer washed with 2 M HCl (4x). The combined aqueous phases were washed with Et_2O (3x), then the combined organic layers were washed with saturated $NaHCO_3$ and brine, dried over anhydrous Na_2SO_4, filtered, and concentrated under reduced pressure.

To facilitate the chromatographic separation of the desired sulfoxide from menthol, silylation of menthol was performed as follows. The crude mixture was stirred for 2 h under vacuum, then dissolved in CH_2Cl_2. Triethylamine (1.50 equiv) was added followed by the dropwise addition of trimethylsilyl chloride TMSCl *or* triethylsilyl chloride TESCl (1.30 equiv) and the mixture was stirred for 3 h at room temperature. The reaction mixture was then diluted with Et_2O and water, the layers separated, and the organic layers were dried over anhydrous

Na_2SO_4, filtered and concentrated under reduced pressure. The crude residue was purified by automated flash column chromatography to afford the desired products.

Stereochemistry at sulfur determined by (+) or (−)-Andersen's sulfinate. Stereochemistry α-to sulfur determined by (+) or (−)-sparteine.

General Procedure 2 (GP2) – Sulfoxide Homologation

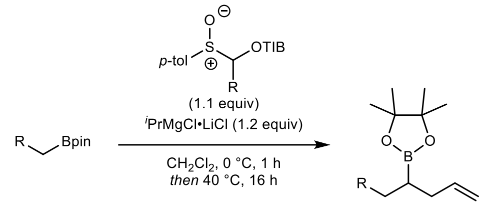

*i*PrMgCl·LiCl (1.14 M in THF, 1.20 equiv) was added dropwise to a mixture of boronic ester (1.00 equiv) and sulfoxide (1.10 equiv) in CH_2Cl_2 (0.20 M *wrt* boronic ester) at 0 °C and the resulting solution was stirred for 1 h at the same temperature (*pale yellow solution*). After warming to room temperature, the reaction mixture was heated to 40 °C for 3 h (*turbid white solution*). The reaction mixture was then cooled to room temperature and was quenched with saturated aqueous NH_4Cl. The aqueous phase was extracted with Et_2O (3x). The combined organic layers were dried over anhydrous Na_2SO_4 and filtered over a short pad of silica deactivated with Et_2O:Et_3N 1% in order to remove the residual TIB acid.

C−B stereochemistry of the product is determined by the stereochemistry of the α sulphur stereocentre of the sulfoxide starting material.

General Procedure 3 (GP3) – Hydroboration

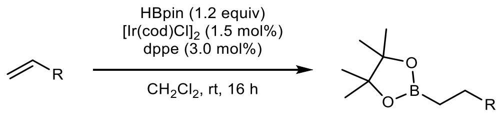

According to a literature procedure.[11] Pinacolborane (1.20 equiv *wrt* alkene) and alkene (1.00 equiv) were added successively to a solution of $[Ir(cod)Cl]_2$ (0.015 equiv) and 1,2-bis(diphenylphosphino)ethane (dppe) (0.030 equiv) in CH_2Cl_2 (0.30 M) at room temperature. The reaction mixture was then stirred for 16 h at room temperature. The reaction was quenched with methanol, diluted with water and extracted with Et_2O (3x). The organic layers were dried over $MgSO_4$ and evaporated under reduced pressure. The crude residue was purified by automated flash column chromatography to afford pure product.

General Procedure 4 (GP4) – Boronic Ester Oxidation

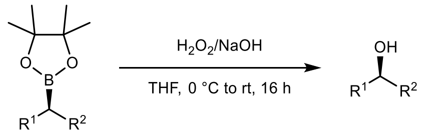

To a solution of boronic ester in THF (0.20 M *wrt* boronic ester), a preformed solution of 3 M NaOH and H_2O_2 30% v/v (2:1, total volume equal to that of THF) was added dropwise at 0 °C. The reaction mixture was vigorously stirred at room temperature for 16 h. Sat. aq. $Na_2S_2O_3$ was added dropwise at 0 °C under stirring and the layers were separated. The aqueous phase was extracted with Et_2O (3x). The combined organic layers were dried over anhydrous Na_2SO_4, filtered and concentrated under reduced pressure.

GP4.1 HPLC samples: The crude residue was analysed by chiral HPLC, further details given in the characterisation section.

GP4.2 Synthetic intermediates: The crude mixture was purified by automated flash column chromatography to afford pure product.

General Procedure 5 (GP5) – TES Protection of Alcohols

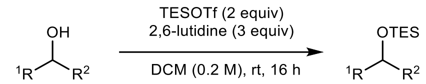

To a suspension of alcohol (1.00 equiv) in CH_2Cl_2 (0.20 M), 2,6-lutidine (3 equiv *wrt* alcohol) was added at room temperature. To the reaction mixture, triethylsilyl trifluoromethanesulfonate TESOTf (2.00 equiv *wrt* alcohol) was added dropwise at 0 °C. The resulting solution was stirred for 16 h at room temperature. Sat. aq. $NaHCO_3$ was added, and the layers were separated. The aqueous phase was extracted with CH_2Cl_2 (3x). The combined organic layers were dried over anhydrous Na_2SO_4, filtered and concentrated under reduced pressure. The crude residue was purified by automated flash column chromatography to afford pure product.

General Procedure 6 (GP6) – Yamaguchi Esterification

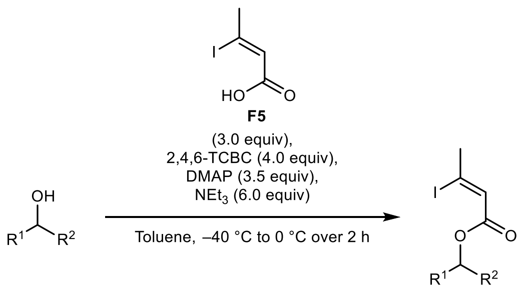

According to a modified procedure, to a solution of alcohol (1.0 equiv) in toluene (0.05 M) was added triethylamine (6.0 equiv) and carboxylic acid **F5** (3.0 equiv) as a solution in toluene (0.35 M *wrt* acid **F5**). The reaction mixture was cooled to −40 °C. At this temperature, 4-dimethylamino pyridine (3.5 equiv) was added as a solution in toluene (0.45 M), followed by 2,4,6-trichlorobenzoyl chloride (37 mg, 0.15 mmol, 4.0 equiv) dissolved in toluene (0.4 M). The resulting reaction mixture was warmed from −40 °C to 0 °C over two hours. The reaction mixture was quenched by the addition of saturated aqueous $NaHCO_3$ and extracted with ethyl acetate (3x). The combined organic layers were dried over anhydrous Na_2SO_4, filtered and concentrated under reduced pressure. The crude residue was purified by automated flash column chromatography to afford pure product.

General Procedure 7 (GP7) – One-pot Hydroboration-Suzuki Cross Coupling

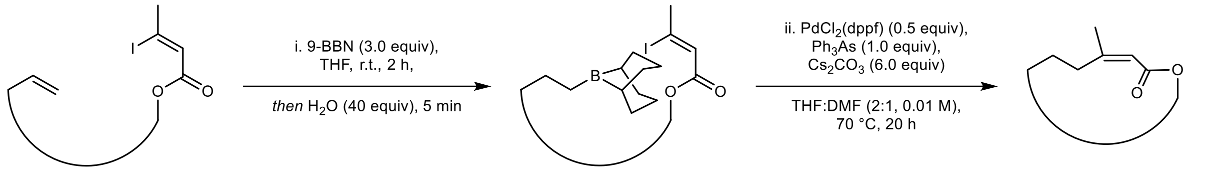

According to a modified literature procedure.[8] To a solution of alkene (1.00 equiv) in dry and degassed THF (0.05 M) was added 9-BBN (0.50 M THF solution, 3.00 equiv) and the reaction mixture was stirred for 2 h at room temperature. HPLC-grade H_2O (40 equiv) was then added to quench the excess 9-BBN, and the reaction mixture was stirred for 5 min, before dry and degassed THF (0.30 mL) was added. The resulting solution was added via syringe pump (0.05 mL/min) to a mixture of $PdCl_2$(dppf) (0.5 equiv), Ph_3As (1.0 equiv), and Cs_2CO_3 (6.0 equiv) in a dry and degassed mixture of THF:DMF 2:1 (1.0 mL) at 70 °C. The resulting reaction mixture was stirred for 20 h at 70 °C, then it was quenched at room temperature by the addition of saturated sodium hydrogen carbonate solution and extracted (3x) with a 1:1 mixture of hexane:Et_2O. The combined organic layers were dried over anhydrous Na_2SO_4, filtered over a short pad of silica and concentrated under reduced pressure. The crude residue was purified by automated flash column chromatography to afford pure product.

5 PREPARATION AND CHARACTERISATION DATA

Note: The synthesis of fragments 1, 2 and 3 were previously described in our synthesis of caylobolide A,[7] *and are reproduced here as the same material was utilised towards caylobolide B.*

But-3-en-1-yl 2,4,6-triisopropylbenzoate - S1

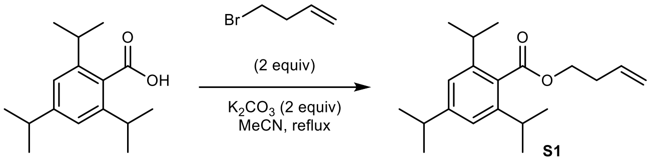

Potassium carbonate (22.3 g, 161 mmol, 2 equiv) was added to a stirred solution of 2,4,6-triisopropylbenzoic acid (20.0 g, 80.5 mmol, 1 equiv) in MeCN (244 mL, 0.33 M). The mixture was stirred vigorously for 10 min then 4-bromobut-1-ene (16.3 mL, 161 mmol, 2 equiv) was added. The resulting mixture was refluxed for 16 h. The reaction mixture was cooled to room temperature and the undissolved K_2CO_3 filtered off and washed with ethyl acetate. The filtrate was evaporated under reduced pressure and the resulting residue was dissolved in ethyl acetate (200 mL), washed with water (2 x 100 mL) and brine (100 mL) before drying over $MgSO_4$. The crude residue was purified by flash column chromatography to give the title compound as a colourless oil (23.1 g, 95%).

All recorded spectroscopic data matched that previously reported[10]

^{1}H NMR (400 MHz, $CDCl_3$) δ 7.01 (s, 2H, *H*-Ar), 5.84 (ddt, *J* = 17.0, 10.3, 6.7 Hz, 1H, C*H*-alkene), 5.16 (ddt, *J* = 17.2, 1.6, 1.6 Hz, 1H, C*H-trans* alkene), 5.10 (ddt, *J* = 10.2, 1.4, 1.4 Hz, 1H, C*H-cis* alkene), 4.38 (t, *J* = 6.7 Hz, 2H, O-CH_2), 2.88 (m, 3H, C*H*-*i*Pr), 2.50 (m, 2H, CH_2), 1.25 (m, 18H, CH_3-*i*Pr). See spectrum.

^{13}C NMR (101 MHz, $CDCl_3$) δ 171.02, 150.24, 144.93, 134.16, 130.65, 120.99, 117.44, 77.48, 77.16, 76.84, 64.19, 34.57, 33.20, 31.60, 24.29, 24.09. See spectrum.

IR (ν_{max}/cm^{-1}, neat): 2961, 2932, 2870, 1725 (C=O), 1607, 1462, 1379, 1321, 1251, 1142, 1077.

(1*R*)-1-(*p*-Tolylsulfinyl)but-3-en-1-yl 2,4,6-triisopropylbenzoate (20) & (1*S*)-1-(*p*-tolylsulfinyl)but-3-en-1-yl 2,4,6-triisopropylbenzoate (S2)

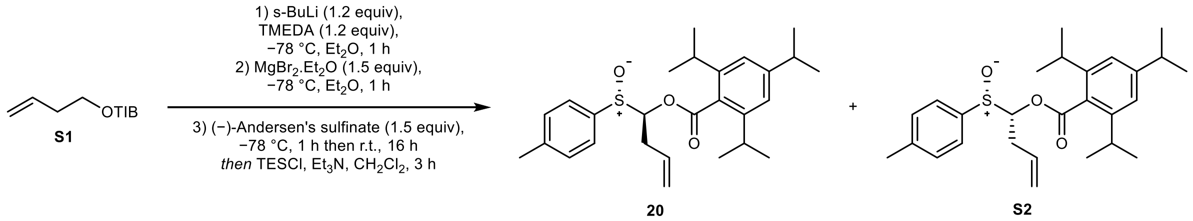

Prepared following **GP1** using alkene **S1** (10.0 g, 33.1 mmol), TMEDA (4.61 g, 5.95 mL, 39.7 mmol), *s*-BuLi (1.3 M in hexanes) (30.5 mL, 39.7 mmol), (–)-Andersen's sulfinate (14.6 g, 49.6 mmol), magnesium turnings (3.21 g, 132 mmol), dibromoethane (9.32 g, 4.29 mL, 49.6 mmol). The reaction mixture was quenched with 2 M HCl (100 mL), the layers separated, and the organic layer washed with 2 M HCl (4 x 50 mL). The combined aqueous phases were washed with Et_2O (3 x 100 mL), then the combined organic layers were washed with saturated $NaHCO_3$ (150 mL) and brine (150 mL), dried over anhydrous Na_2SO_4, filtered, and concentrated under reduced pressure.

TES protection of menthol to facilitate in separation

Et_3N (5.02 g, 6.91 mL, 49.6 mmol), trimethylsilyl chloride (4.67 g, 5.45 mL, 43.0 mmol). The reaction mixture was then diluted with Et_2O (70 mL) and water (100 mL), the layers separated, and the organics were dried over anhydrous Na_2SO_4, filtered and concentrated under reduced pressure. The crude residue was purified by automated flash column chromatography in two batches (Biotage HC-100 g, hexane: ethyl acetate 0-20%) to afford the title compounds:

All recorded spectroscopic data matched that previously reported[10]

Less polar ***syn*** (**S2**) (3.00 g, 21%, >20:1 *d.r.*, ≥99:1 *e.r.*) as a crystalline white solid.

***syn*-S2**

^{1}H NMR (400 MHz, $CDCl_3$) δ: 7.67 (d, J = 8.0 Hz, 2H, Ar-H), 7.38 (d, J = 8.0 Hz, 2H, Ar-H), 7.04 (s, 2H, 14-H), 5.75 (dd, J_1 = 10.0 Hz, J_2 = 3.0 Hz, 1H, 5-H), 5.67 (m, 1H, 7-H), 5.11–5.06 (m, 2H, 7-H + 8-H), 2.91 (app sept, J = 6.6 Hz, 3H, 12-H + 16-H), 2.72 (m, 1H, 6-H), 2.44 (s, 3H, 1-H), 2.42 (m, 1H, 6-H), 1.28–1.23 (m, 18H, CH3 × 6) ppm See spectrum.

^{13}C NMR (101 MHz, $CDCl_3$) δ: 170.1 (CO), 150.7 (Ar-C), 145.1 (2C, Ar-C × 2), 141.6 (Ar-C), 137.2 (Ar-C), 131.3 (Ar-C), 130.0 (2C, Ar-C × 2), 128.7 (7-C), 124.3 (2C, Ar-C × 2), 120.9 (2C, Ar-C × 2), 119.2 (8-C), 91.3 (5-C), 34.3 (16-C), 31.4 (6-C), 27.7 (2C, 12-C), 24.3 (2C, CH-CH3 × 2), 24.1 (2C, CH-CH3 × 2), 23.8 (2C, CH-CH3 × 2), 21.3 (1-C) ppm See spectrum.

More polar ***anti*** (**20**) (6.75 g, 46%, >20:1 *d.r.*, ≥99:1 *e.r.*) as a pale orange oil.

***anti*-S2**

^{1}**H NMR** (400 MHz, $CDCl_3$) δ: 7.55 (d, J = 8.1 Hz, 2H, Ar-H), 7.32 (d, J = 8.1 Hz, 2H, Ar-H), 7.02 (s, 2H, 14-H), 6.07 (dd, J_1 = 8.9 Hz, J_2 = 3.6 Hz, 1H, 5-H), 5.78 (m, 1H, 7-H), 5.15 (m, 2H, 7-H + 8-H), 2.90 (sept, J = 7.0 Hz, 1H, 16-H), 2.83 (sept, J = 6.7 Hz, 2H, 12-H × 2), 2.70 (m, 1H, 6-H), 2.42 (s, 3H, 1-H), 2.08 (m, 1H, 6-H), 1.26–1.18 (m, 18H, CH3 × 6) ppm See spectrum.

^{13}**C NMR** (101 MHz, $CDCl_3$) δ: 169.1 (CO), 150.6 (Ar-C), 145.2 (2C, Ar-C × 2), 142.1 (Ar-C), 136.0 (Ar-C), 131.3 (Ar-C), 129.8 (2C, Ar-C × 2), 128.8 (7-C), 125.4 (2C, Ar-C × 2), 120.9 (2C, Ar-C × 2), 119.5 (8-C), 87.2 (5-C), 34.3 (16-C), 31.8 (6-C), 31.3 (2C, 12-C), 24.4 (2C, CH-CH3 × 2), 24.0 (2C, CH-CH3 × 2), 23.8 (2C, CH-CH3 × 2), 21.4 (1-C) ppm See spectrum.

Fragment 1 (14)

hex-5-en-1-yl 2,4,6-triisopropylbenzoate – 18

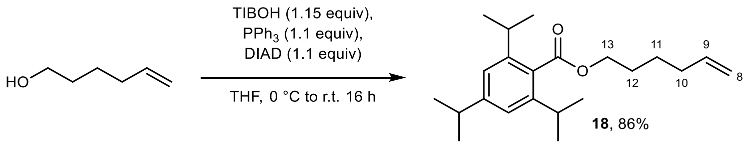

To a stirred suspension of PPh_3 (2.88 g, 10.9 mmol, 1.10 equiv) and DIAD (2.22 g, 10.9 mmol, 1.10 equiv) in THF (20 mL, 0.50 M) at 0 °C (ice bath), hex-5-en-1-ol (1.00 g, 9.98 mmol, 1.00 equiv) and 2,4,6-triisopropylbenzoic acid (2.85 g, 11.5 mmol, 1.15 equiv) were added. The reaction mixture was stirred at r.t. for 16 h. The volatiles were removed *in vacuo* and the crude residue was partitioned between Et_2O (40 mL) and sat. aq. $NaHCO_3$ (20 mL). The organic layer was then washed with water (20 mL) and brine (20 mL) and dried over $MgSO_4$. The crude mixture was purified by normal phase flash column chromatography (hexane:ethyl acetate 9:1) to afford the title compound (2.83 g, 86% yield) as a colourless oil.

^{1}**H NMR (400 MHz, $CDCl_3$) δ** 7.01 (s, 2H, *H*-Ar), 5.80 (ddt, *J* = 16.9, 10.1, 6.6 Hz, 1H, *H*-9), 5.02 (dq, *J* = 17.1, 1.7 Hz, 1H, C*H*-10-*cis* alkene), 4.97 (ddt, *J* = 10.1, 2.2, 1.2 Hz, 1H, C*H*-10-*trans* alkene), 4.31 (t, *J* = 6.6 Hz, 2H, *H*-13), 2.86 (m, 3H, C*H*-*i*Pr), 2.10 (tdt, *J* = 7.9, 6.6, 1.4 Hz, 2H, *H*-8), 1.82 – 1.68 (m, 2H, *H*-12), 1.56 – 1.47 (m, 2H, *H*-11), 1.25 (d, *J* = 6.7 Hz, 18H, CH_3-*i*Pr). See spectrum.

^{13}**C NMR (101 MHz, $CDCl_3$) δ** 171.16 (*C*O-benzoate), 150.20 (*C*-*para*), 144.87 (*C*-*ortho*), 138.39 (*C*H-9 alkene), 130.81 (*C*-*ipso*), 120.99 (*C*H-*meta*), 115.00 (*C*H_2-8 alkene), 64.94

(CH_2-13), 34.57 (*C*H-*i*Pr *para*), 33.40 (*C*H_2-10), 31.64 (*C*H-*i*Pr *ortho*), 28.22 (*C*H_2-12), 25.47 (*C*H_2-11), 24.29 (*C*H_3-*i*Pr *ortho*), 24.10 (*C*H_3-*i*Pr *para*). See spectrum.

HRMS (m/z): (ESI) calculated for $C_{22}H_{34}O_2Na$ $[M+Na]^+$ 353.2451, found 353.2449.

TLC: ***R*f** = 0.48 (95:5 hexane:ethyl acetate, stained with *p*-anisaldehyde, product visible under UV lamp).

IR (ν_{max}/cm^{-1}, neat): 2960, 2931, 2870, 1725 (C=O), 1607, 1461, 1363, 1250, 1189, 1137, 1075.

(*S*)-5,6-bis(4,4,5,5-tetramethyl-1,3,2-dioxaborolan-2-yl)hexyl 2,4,6-triisopropylbenzoate - 19

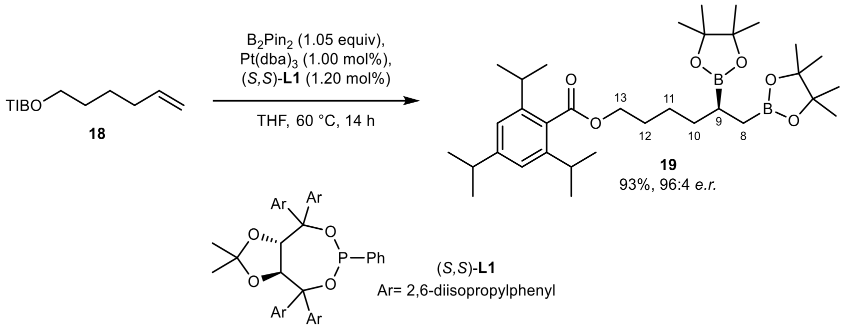

According to a modified literature procedure[2]. To a flame dried Schlenk tube equipped with a magnetic stir bar was added Pt(dba)$_3$ (27.7 mg, 30.3 µmol, 1.00 mol%), (*S*,*S*)-**L1** (33.0 mg, 36.3 µmol, 1.20 mol%) and B_2Pin_2 (807 mg, 3.18 mmol, 1.05 equiv) under a nitrogen atmosphere. After addition of the solids, the Schlenk was evacuated and refilled with nitrogen three times. Then, THF (3.0 mL, 1.0 M *wrt* alkene **18**) was added via syringe. The solution was heated to 80 °C in an oil bath for 30 minutes. The reaction mixture was then cooled to room temperature and alkene **18** (1.00 g, 3.03 mmol, 1.00 equiv) was added neat. The reaction mixture was stirred for 14 h at 60 °C. The reaction mixture was then cooled to room temperature and the solvent was evaporated under reduced pressure. The crude mixture was purified by automated flash column chromatography (Biotage HC-50 g, pentane: Et_2O 0-10%) to afford the title compound (1.65 g, 93%) as a colourless oil.

^{1}H NMR (400 MHz, CDCl$_3$) δ 6.99 (s, 2H, *H*-Ar), 4.27 (t, *J* = 6.9 Hz, 2H, *H*-13), 2.86 (m, 3H, C*H*-*i*Pr), 1.71 (m, C*H*$_2$-13), 1.57 – 1.28 (m, 6H, C*H*$_2$-10 & C*H*$_2$-11 & C*H*$_2$-12), 1.26 – 1.16 (m, 42H, C*H*$_3$-*i*Pr and C*H*$_3$-Bpin), 1.14 – 1.05 (m, 1H, CH-9), 0.91 – 0.75 (m, 2H, CH$_2$-8). See spectrum.

^{13}C NMR (101 MHz, CDCl$_3$) δ 171.16 (*C*O-benzoate), 150.07 (*C*-TIB-*para*), 144.87 (*C*-TIB-*ortho*), 130.93 (*C*-TIB-*ipso*), 120.92 (*C*H-TIB-*meta*), 83.00 (*C*-Bpin), 82.97 (*C*-Bpin), 65.28 (*C*H$_2$-13), 34.57 (*C*H-*i*Pr *para*), 33.38 (*C*H$_2$-12), 31.59 (*C*H-*i*Pr *ortho*), 28.99 (*C*H$_2$-11), 25.42 (*C*H$_2$-10), 25.03 (*C*H$_3$-Bpin), 24.97 (*C*H$_3$-Bpin), 24.93 (*C*H$_3$-Bpin), 24.86 (*C*H$_3$-Bpin), 24.30 (*C*H$_3$-*i*Pr *ortho*), 24.11 (*C*H$_3$-*i*Pr *para*). See spectrum.

HRMS (m/z): (MALDI) calculated for $C_{34}H_{58}B_2O_6Na$ $[M+Na]^+$ 607.4323, found 607.4328.

TLC: R_f = 0.16 (95:5 hexane:ethyl acetate, stained with *p*-anisaldehyde, product visible under UV lamp).

IR (ν_{max}/cm^{-1}, neat): 2962, 2930, 2870, 1722 (C=O), 1606, 1462, 1369, 1312, 1251, 1138, 1076, 968, 846.

$[\alpha]_D^{25}$: 4 (c = 1, $CHCl_3$).

The racemic bis-boronic ester was prepared by diboration using a method described in the literature for similar compounds.[12]

The enantioenriched bis-boronic ester and racemic bis-boronic ester were both oxidised according to ***GP4.1*** *for chiral HPLC analysis.*

HPLC on chiral stationary phase Daicel Chiralpak-IB column (25 cm), 98:2 *n*-hexane:IPA, flow rate: 1 mL/min., r.t., 210 nm, Retention times: 38.47 min. (*S*, major) and 37.80 min. (*R*, minor).

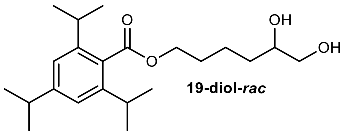

19-diol-*rac*

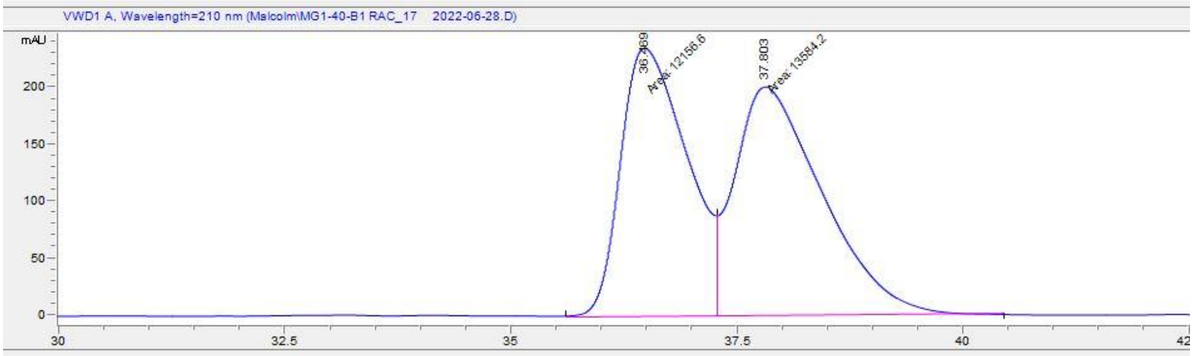

#	Time	Type	Area	Height	Width	Area%	Symmetry
1	36.469	MF	12156.6	236.7	0.8559	47.227	0.566
2	37.803	FM	13584.2	201.8	1.1218	52.773	0.517

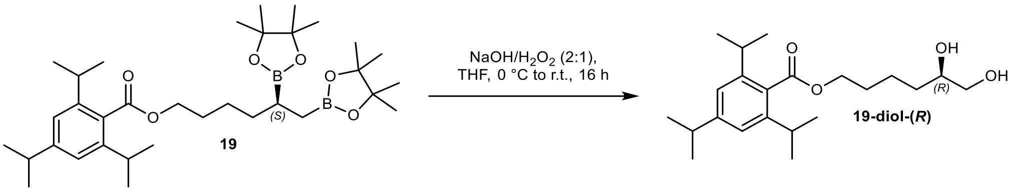

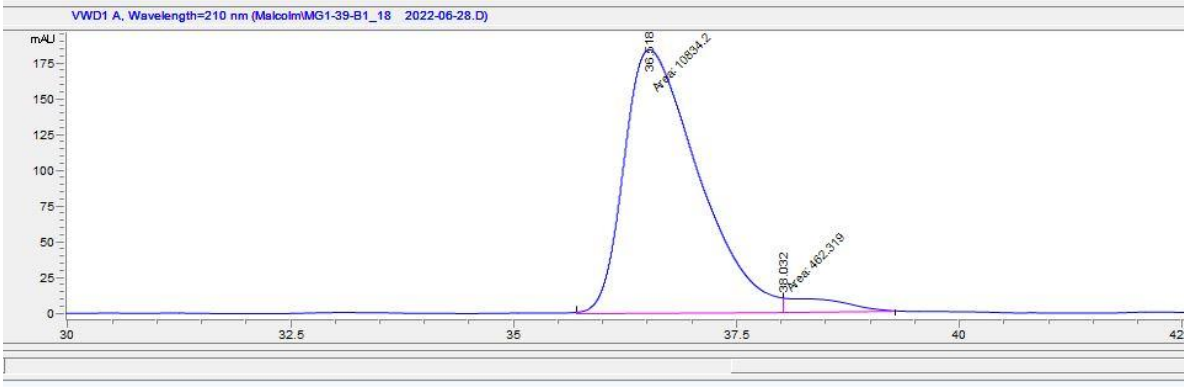

#	Time	Type	Area	Height	Width	Area%	Symmetry
1	36.518	MM	10834.2	186	0.9708	95.907	0.502
2	38.032	MM	462.3	10.3	0.526	4.093	0

4-((4*R*,6*S*)-6-allyl-2,2-dimethyl-1,3-dioxan-4-yl)butyl 2,4,6-triisopropylbenzoate – 14

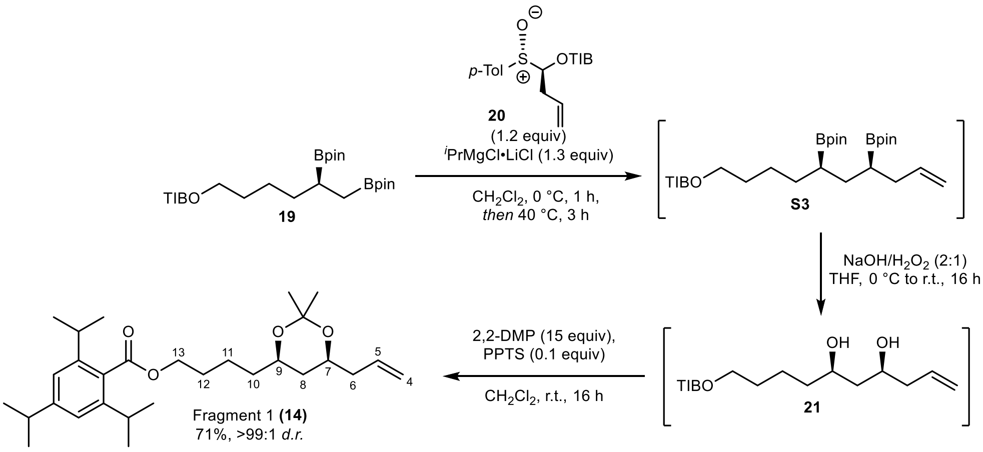

Boronic ester homologation

Prepared following General Procedure 2 using boronic ester **19** (270 mg, 0.462 mmol), sulfoxide **20** (244 mg, 0.55 mmol), *i*PrMgCl·LiCl (1.2 M in THF, 0.50 mL, 0.60 mmol) in CH_2Cl_2 (8.5 mL). The reaction mixture was then cooled to room temperature and was quenched with saturated aqueous NH_4Cl (5 mL). The aqueous phase was extracted with Et_2O (3 x 15 mL). The combined organic layers were dried over anhydrous Na_2SO_4 and filtered over a short pad of silica approximately 3 cm in depth, pre-mixed as a slurry with Et_2O:Et_3N (20 mL) 1% in order to deactivate the silica to remove residual TIB acid.

The organic layers were concentrated under reduced pressure and the crude mixture was engaged in the next step without further purification.

Boronic ester oxidation

Crude 1,3-bis-boronic ester **S3** was oxidised according to General Procedure 4 using THF (2.3 mL, 0.20 M *wrt* bis-boronic ester), a preformed solution of NaOH 3M (1.5 mL), and H_2O_2 30% v/v (0.76 mL). Sat. aq. $Na_2S_2O_3$ (1.5 mL) was added dropwise at 0 °C under stirring and the layers were separated. The aqueous phase was extracted with Et_2O (3 x 4.5 mL). The combined organic layers were dried over anhydrous Na_2SO_4, filtered and concentrated under reduced pressure. The crude mixture was purified by automated flash column chromatography (Biotage HC-10 g, pentane: ethyl acetate 0-100%) to afford crude diol **21** and was used in the following step without further purification.

1,3-Diol acetonide protection

To a solution of crude diol **21** in CH_2Cl_2 (4.6 mL, 0.10 M) was added 2,2-dimethoxypropane (0.72 g, 0.85 mL, 6.9 mmol, 15 equiv) and pyridinium *p*-toluene sulfonate (12 mg, 46 µmol, 0.1 equiv). The reaction mixture was stirred for 16 h at r.t. Sat. aq. $NaHCO_3$ (5 mL) was added, and the layers were separated. The aqueous phase was extracted with CH_2Cl_2 (3 x 10 mL)). The combined organic layers were dried over anhydrous Na_2SO_4, filtered and concentrated under reduced pressure. The crude mixture was purified by normal phase flash column chromatography (Biotage HC-25 g, pentane:Et_2O 0-20%) to afford the title compound as a mixture of diastereomers at C9. The product was repurified by reverse phase preparative HPLC (Teledyne ACCQprep HP150, 21.2 mL/min, Agilent 5 Prep-C18, 250 x 21.2 mm column, water: acetonitrile 85-92%) to afford the title compound as a single diastereomer (150 mg, 71%) as a colourless oil.

Note: due to acetonide lability under acidic conditions, it is recommended to use $CDCl_3$ filtered over basic alumina or CD_2Cl_2 as solvent for NMR analysis.

^{1}H NMR (400 MHz, $CDCl_3$) δ 7.00 (s, 2H, *H*-Ar), 5.80 (dddd, *J* = 16.9, 10.2, 7.5, 6.5 Hz, 1H, *H*-5), 5.13 – 5.01 (m, 2H, C*H*$_2$-4-alkene), 4.30 (t, *J* = 6.5 Hz, 2H, *H*-13), 3.86 (dtd, *J* = 11.5, 6.3, 2.5 Hz, 1H, *H*-7), 3.79 (dddd, *J* = 11.5, 6.8, 4.7, 2.4 Hz, 1H, *H*-9), 2.87 (m, 3H, C*H*-*i*Pr), 2.30 (m, 1H, *H*-6’), 2.14 (m, 1H, *H*-6’’), 1.73 (m, 2H, *H*-12), 1.60 – 1.42 (m, 6H, *H*-8’ & *H*-10 & *H*-11), 1.42 – 1.40 (m, 3H, CH_3-acetonide), 1.38 (d, *J* = 0.8 Hz, 3H, CH_3-acetonide), 1.24 (d, *J* = 6.9 Hz, 18H, C*H*$_3$-*i*Pr), 1.12 (m, *H*-8’’). See spectrum.

^{13}C NMR (101 MHz, CDCl$_3$) δ 171.11 (*C*O-benzoate), 150.21 (*C-para*), 144.85 (*C-ortho*), 134.35 (*C*H-5 alkene), 130.81 (*C-ipso*), 120.98 (*C*H-*meta*), 117.20 (*C*H$_2$-4 alkene), 98.58 (CO *syn*-acetonide), 68.91 (*C*H-9), 68.78 (*C*H-7), 65.03 (*C*H$_2$-13), 41.02 (*C*H$_2$-6), 36.57 (*C*H$_2$-8), 36.19 (*C*H$_2$-10), 34.57 (*C*H-*i*Pr *para*), 31.64 (*C*H-*i*Pr *ortho*), 30.38 (*C*H$_3$-*syn*-acetonide), 28.77 (*C*H$_2$-12), 24.30 (*C*H$_3$-*i*Pr *ortho*), 24.10 (*C*H$_3$-*i*Pr *para*), 21.84 (*C*H$_2$-11), 19.91 (*C*H$_3$-*syn*-acetonide). See spectrum.

HRMS (m/z): (ESI) calculated for $C_{29}H_{47}O_4$ [M+H]$^+$ 459.3469, found 459.3490.

TLC: R_f = 0.24 (95:5 hexane:ethyl acetate, stained with *p*-anisaldehyde, product visible under UV lamp).

IR (ν$_{max}$/cm^{-1}, neat): 3076, 2961, 2940, 2869, 1725 (C=O), 1643 (C=C), 1462, 1379, 1251, 1138, 1077, 877.

$[\alpha]_D^{25}$: −4 (*c* = 1, CHCl$_3$).

Fragment 2 (15)

(*S*)-3,4-Bis(4,4,5,5-tetramethyl-1,3,2-dioxaborolan-2-yl)butyl 2,4,6-triisopropylbenzoate – S5

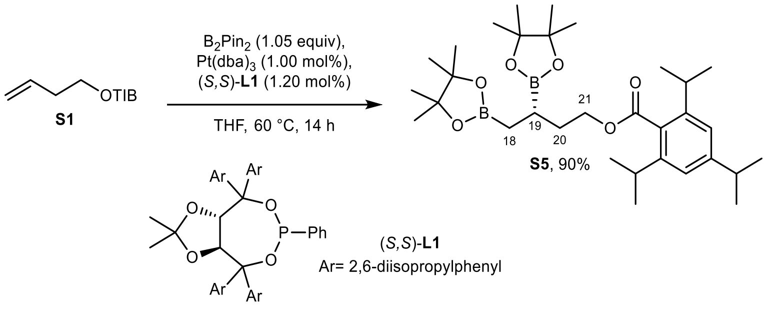

According to the modified literature procedure[2], to a flame dried Schlenk tube equipped with a magnetic stir bar, Pt(dba)$_3$ (151 mg, 165 μmol, 1.00 mol%), (*S*,*S*)-**L1** (180 mg, 198 μmol, 1.20 mol%) and B$_2$Pin$_2$ (4.41 g, 17.4 mmol, 1.05 equiv) was added under a nitrogen atmosphere. After the addition of solids, the Schlenk tube was evacuated and refilled with nitrogen three times. Then, THF (1.00 M *wrt* the alkene) was added via syringe. The solution was heated to 80 °C in an oil bath for 30 minutes. The reaction mixture was then cooled to room temperature and alkene **S1** (5.00 g, 16.5 mmol, 1.00 equiv) was added neat. The reaction mixture was stirred for 14 h at 60 °C. The reaction mixture was then cooled to room temperature and the solvent was evaporated under reduced pressure. The crude mixture was

purified by automated flash column chromatography (Biotage HC-50 g, pentane: Et_2O 0-10%) to afford the title compound (8.24 g, 90%) as a colourless oil.

All recorded spectroscopic data matched that previously reported[8]

TLC: R_f = 0.40 (85:15 pentane:Et_2O, stained with *p*-anisaldehyde).

^{1}H NMR (400 MHz, $CDCl_3$) δ 6.98 (s, 2H, *H*-Ar), 4.33 (t, *J* = 6.8, 2H, *H*-21), 2.87 (hept, *J* = 6.9 Hz, 3H, C*H*-*i*Pr), 1.90 (ddt, *J* = 13.6, 8.4, 6.8 Hz, 1H, *H*-20'), 1.73 (dq, *J* = 13.8, 7.0 Hz, 1H, *H*-20''), 1.24 (d, *J* = 6.9 Hz, 18H, CH_3-*i*Pr), 1.21 (m, 24H, CH_3-Bpin), 0.94 – 0.84 (m, 3H, *H*-18 & *H*-19). See spectrum.

^{13}C NMR (101 MHz, $CDCl_3$) δ 171.13 (*C*O-benzoate), 149.98 (*C*H-TIB-*para*), 144.88 (*C*H-TIB-*ortho*), 131.10 (*C*H-TIB-*ipso*), 120.88 (*C*H-TIB-*meta*), 83.14 (*C*-Bpin), 83.07 (*C*-Bpin), 64.64 (*C*H_2-21), 34.58 (*C*H-*i*Pr *para*), 32.19 (*C*H_2-20), 31.55 (*C*H-*i*Pr *ortho*), 25.00 (*C*H_3-Bpin), 24.95 (*C*H_3-Bpin), 24.87 (*C*H_3-Bpin), 24.32 (*C*H_3-*i*Pr), 24.30 (*C*H_3-*i*Pr), 24.11 (*C*H_3-*i*Pr). See spectrum.

Carbons next to boron (*C*H_2-18 & *C*H-19) *not observed due to quadrupolar relaxation.*

HRMS (m/z): (MALDI) calculated for $C_{32}H_{54}B_2O_6Na$ $[M+Na]^+$ 579.4010, found 579.4016.

IR (ν_{max}/cm^{-1}, neat): 2963, 2930, 2871, 1724 (C=O), 1463, 1371, 1317, 1251, 1140, 1077.

$[\alpha]_D^{25}$: 6 (*c* = 1, $CHCl_3$).

The racemic bis-boronic ester was prepared by diboration using a method described in the literature for similar compounds.[12]

The enantioenriched bis-boronic ester and racemic bis-boronic ester were both oxidised according to ***GP4.1*** *for chiral HPLC analysis.*

HPLC on chiral stationary phase Daicel Chiralpak-IB column (25 cm), 97:3 *n*-hexane:IPA, flow rate: 0.3 mL/min., rt, 210 nm, Retention times: 55.59 min. (*S*, major) and 58.95 min. (*R*, minor).

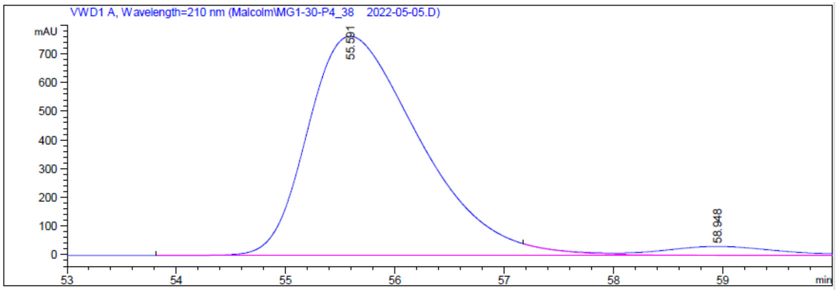

Peak #	RetTime [min]	Type	Width [min]	Area [mAU*s]	Height [mAU]	Area %
1	55.591	BV R	1.0970	5.45397e4	764.75079	95.7918
2	58.948	VB E	1.1421	2395.96533	31.24835	4.2082

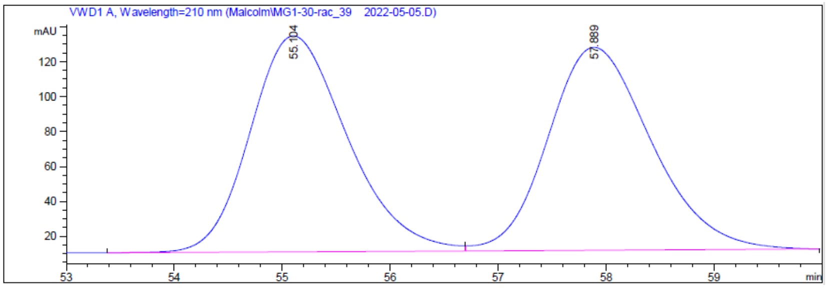

Peak #	RetTime [min]	Type	Width [min]	Area [mAU*s]	Height [mAU]	Area %
1	55.104	BV	0.9656	7732.78467	123.63711	49.9796
2	57.889	VBA	1.0246	7739.08398	116.18924	50.0204

(3*R*,5*R*)-3,5-Dihydroxyoct-7-en-1-yl 2,4,6-triisopropylbenzoate – S7

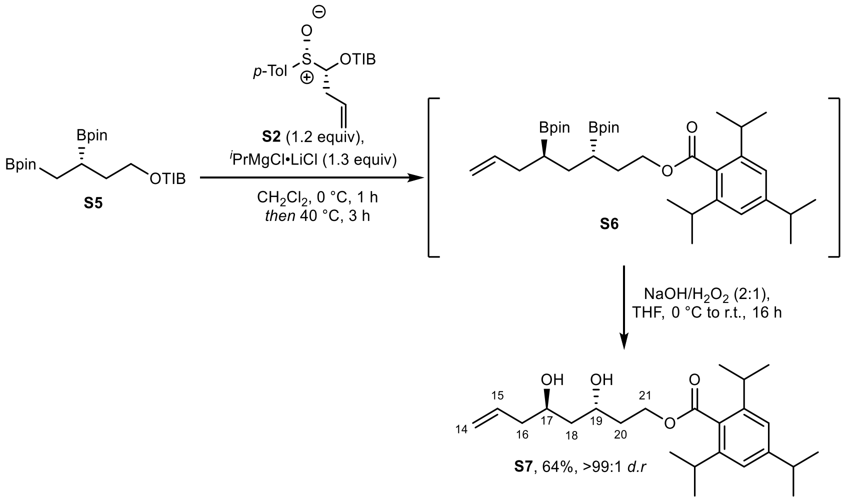

Prepared following General Procedure 2 using boronic ester **S5** (2.00 g, 3.59 mmol), sulfoxide **S2** (1.90 g, 4.31 mmol), and *i*PrMgCl·LiCl (1.2 M in THF, 3.89 mL, 4.67 mmol) in CH_2Cl_2 (18 mL). The reaction mixture was then cooled to room temperature and was quenched with saturated aqueous NH_4Cl (10 mL). The aqueous phase was extracted with

Et_2O (3 x 30 mL). The combined organic layers were dried over anhydrous Na_2SO_4 and filtered over a short pad of silica approximately 5 cm in depth, pre-mixed as a slurry with Et_2O:Et_3N (40 mL) 1% in order to deactivate the silica to remove residual TIB acid.

The organic layers were concentrated under reduced pressure and the crude mixture was engaged in the next step without further purification.

Crude 1,3-bis-boronic ester **S6** was oxidised according to General Procedure 4 using THF (18 mL, 0.20 M *wrt* bis-boronic ester **S6**), a preformed solution of 3 M NaOH (12.0 mL) and H_2O_2 30% v/v (6.0 mL). Sat. aq. $Na_2S_2O_3$ (12 mL) was added dropwise at 0 °C under stirring and the layers were separated. The aqueous phase was extracted with Et_2O (3 x 30 mL). The combined organic layers were dried over anhydrous Na_2SO_4, filtered and concentrated under reduced pressure. The crude mixture was purified by automated flash column chromatography (Biotage HC-100g, pentane: ethyl acetate 0-40%) to afford the title compound (904 mg, 64% yield over 2 steps, >99:1 *d.r.*) as a colourless oil.

All recorded spectroscopic data matched that previously reported[8]

^{1}H NMR (400 MHz, $CDCl_3$) δ 7.00 (s, 2H, *H*-Ar), 5.87 – 5.73 (m, 1H, *H*-15), 5.19 – 5.10 (m, 2H, *H*-14), 4.56 (ddd, *J* = 11.1, 7.4, 6.3 Hz, 1H, *H*-21), 4.40 (dt, *J* = 11.4, 5.8 Hz, 1H, *H*-21), 4.19 – 4.07 (m, 1H, *H*-19), 4.06 – 3.96 (m, 1H, *H*-17), 2.96 – 2.77 (m, 3H, *H*-iPr), 2.35 – 2.20 (m, 3H, *H*-16), 1.97 – 1.84 (m, 2H, *H*-20), 1.75 – 1.62 (m, 2H, *H*-18), 1.30 – 1.20 (m, 18H, CH_3-iPr). See spectrum.

^{13}C NMR (101 MHz, $CDCl_3$) δ 171.34 (*C*O-benzoate), 150.39 (*C-para*), 144.92 (*C-ortho*), 134.59 (*C*H-15), 130.46 (*C-ipso*), 121.02 (*C*H-Ar), 118.65 (*C*H_2-14), 68.26 (*C*H-17), 66.30 (*C*H_2-19), 62.32 (*C*H_2-21), 42.24 (*C*H_2-18), 42.13 (*C*H_2-16), 36.55 (*C*H_2-20), 34.57 (*C*-*i*Pr *para*), 31.70 (*C*H-*i*Pr *ortho*), 24.31 (*C*H_3-*i*Pr), 24.28 (*C*H_3-*i*Pr), 24.08 (*C*H_3-*i*Pr). See spectrum.

IR (ν_{max}/cm^{-1}, neat): 3454 (OH), 2970, 2928, 2869, 1738 (C=O), 1649 (C=C), 1463, 1365, 1085, 1042, 1023, 812.

2-((4*R*,6*R*)-6-Allyl-2,2-dimethyl-1,3-dioxan-4-yl)ethyl 2,4,6-triisopropylbenzoate – S8

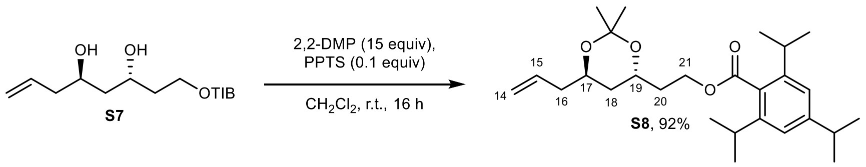

To a solution of diol **S7** (850 mg, 2.18 mmol, 1.00 equiv) in CH_2Cl_2 (22 mL, 0.10 M) was added 2,2-dimethoxypropane (3.40 g, 3.26 mmol, 15.0 equiv) and pyridinium *p*-toluene sulfonate (54.7 mg, 218 µmol 0.10 equiv). The reaction mixture was stirred for 16 h at ambient temperature. Saturated aqueous $NaHCO_3$ (10 mL) was added, and the layers were separated. The aqueous phase was extracted with CH_2Cl_2 (3 x 20 mL). The combined organic layers were dried over anhydrous Na_2SO_4, filtered and concentrated under reduced pressure. The crude mixture was purified by normal phase flash column chromatography (Biotage HC-50 g, *n*-hexane: ethyl acetate 0-10%) to afford the title compound (860 mg, 92%) as a colourless oil.

Note: due to acetonide lability under acidic conditions, it is recommended to use $CDCl_3$ filtered over basic alumina or CD_2Cl_2 as solvent for NMR analysis.

All recorded spectroscopic data matched that previously reported[8]

^{1}H NMR (400 MHz, $CDCl_3$) δ 7.01 (s, 2H, *H*-Ar), 5.79 (ddt, *J* = 17.1, 10.3, 6.8 Hz, 1H, *H*-15), 5.15 – 5.00 (m, 2H, *H*-14), 4.45 – 4.32 (m, 2H, *H*-21), 3.96 (m, 1H, *H*-19), 3.88 (m, 1H, *H*-17), 2.95 – 2.78 (m, 3H, *H*-iPr), 2.30 (ddd, *J* = 13.2, 6.7, 6.7 Hz, 1H, *H*-16'), 2.24 – 2.14 (, 1H, *H*-16''), 1.96 – 1.81 (m, 2H, *H*-20), 1.63 (dd, *J* = 7.7 Hz, 2H, *H*-18), 1.36 (s, 3H, CH_3-acetonide), 1.35 (s, 3H, CH_3-acetonide), 1.24 (d, *J* = 6.8 Hz, 18H, CH_3-iPr). See spectrum.

^{13}C NMR (101 MHz, $CDCl_3$) δ 171.06 (*C*O-benzoate), 150.29 (*C-para*), 144.88 (*C-ortho*), 134.46 (*C*H-15), 130.66 (*C-ipso*), 121.00 (*C*H-Ar), 117.12 (*C*H_2-14), 100.57 (*C*O *anti*-acetonide), 66.25 (*C*H-17), 63.62 (*C*H_2-19), 61.83 (*C*H_2-21), 40.26 (*C*H_2-16), 38.03 (*C*H_2-18), 35.02 (*C*H_2-20), 34.57 (*C*-*i*Pr *para*), 31.67 (*C*H-*i*Pr *ortho*), 24.91 (*C*H3-*anti*-acetonide), 24.88 (*C*H3-*anti*-acetonide), 24.36 (*C*H_3-*i*Pr), 24.25 (*C*H_3-*i*Pr), 24.09 (*C*H_3-*i*Pr). See spectrum.

2-((4*R*,6*R*)-2,2-Dimethyl-6-(3-(4,4,5,5-tetramethyl-1,3,2-dioxaborolan-2-yl)propyl)-1,3-dioxan-4-yl)ethyl 2,4,6-triisopropylbenzoate - 15

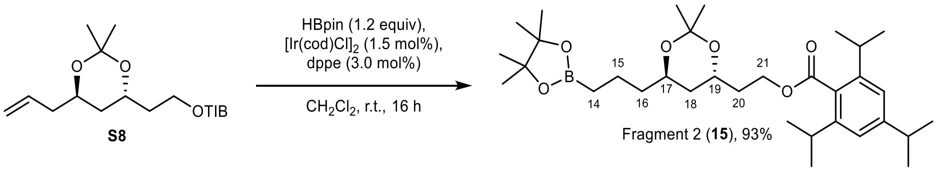

Prepared following General Procedure 3 using pinacolborane (446 mg, 0.505 mL, 3.48 mmol), alkene **S8** (1.00 g, 2.32 mmol), $[Ir(cod)Cl]_2$ (24 mg, 35 µmol) and 1,2-

bis(diphenylphosphino)ethane dppe (28 mg, 70 μmol) in CH_2Cl_2 (6.4 mL). The reaction was quenched with methanol (1 mL) diluted with water (5 mL) and extracted with Et_2O (3 x 10 mL). The organic layers were dried over $MgSO_4$ and evaporated under reduced pressure. The crude mixture was purified by normal phase flash column chromatography (Biotage HC-50 g, pentane: Et_2O 0-10%) to afford the title compound (1.21 g, 93%) as a colourless oil.

All recorded spectroscopic data matched that previously reported[8]

^{1}H NMR (400 MHz, $CDCl_3$) δ 7.00 (s, 2H, *H*-Ar), 4.48 – 4.28 (m, 2H, *H*-21), 4.03 – 3.87 (m, 1H, *H*-19), 3.87 – 3.71 (m, 1H, *H*-17), 2.86 (hept, *J* = 6.9 Hz, 3H, *H*-iPr), 1.99 – 1.78 (m, 2H, *H*-20), 1.68 – 1.36 (m, 6H, *H*-15, *H*-16, *H*-18),1.34 (s, 3H, CH_3-acetonide), 1.32 (s, 3H, CH_3-acetonide), 1.28 – 1.20 (m, 30H, CH_3-*i*Pr & CH_3-Bpin), 0.78 (t, *J* = 7.4 Hz, 2H, *H*-14). See spectrum.

^{13}C NMR (101 MHz, $CDCl_3$) δ 171.08 (*C*O-benzoate), 150.27 (*C-para*), 144.89 (*C-ortho*), 130.71 (*C-ipso*), 121.01 (*C*H-Ar), 100.45 (*C*O *anti*-acetonide), 83.08 (*C*-Bpin), 66.54 (*C*H-17), 63.64 (*C*H-19), 61.92 (*C*H_2-21), 38.78 (*C*H_2-16), 38.76 (*C*H_2-18), 35.07 (*C*H_2-20), 34.59 (*C*-*i*Pr *para*), 31.67 (*C*H-*i*Pr *ortho*), 24.99 (*C*H_3-Bpin), 24.97 (*C*H_3-Bpin), 24.94 (*C*H_3-*anti*-acetonide), 24.86 (*C*H_3-*anti*-acetonide), 24.36 (*C*H_3-*i*Pr), 24.26 (*C*H_3-*i*Pr), 24.10 (*C*H_3-*i*Pr), 19.86 (*C*H_2-15). See spectrum.

Carbon next to boron (*C*H_2-14) *not observed due to quadrupolar relaxation.*

HRMS (m/z): (ESI) calculated for $C_{33}H_{55}BO_6Na$ $[M+Na]^+$ 581.3984, found 581.4000.

IR (ν_{max}/cm^{-1}, neat): 2961, 2934, 2871, 1726 (C=O), 1607, 1379, 1250, 1144, 1076.

Fragment 3 (16)

Propane-1,3-diyl bis(2,4,6-triisopropylbenzoate) – S9

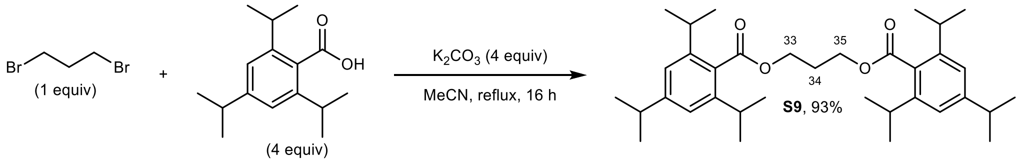

Potassium carbonate (21.9 g, 159 mmol, 4.00 equiv) was added to a stirred solution of 2,4,6-triisopropylbenzoic acid (39.4 g, 159 mmol, 4.00 equiv) in MeCN (120 mL, 0.33 M). The mixture was stirred vigorously for 10 min then 1,3-dibromopropane (8.00 g, 4.02 mL, 39.6

mmol, 1.00 equiv) was added. The resulting mixture was refluxed for 16 h. The reaction mixture was cooled to room temperature and undissolved K_2CO_3 was removed by filtration and washed with ethyl acetate (60 mL). The filtrate was evaporated under reduced pressure, and the resulting residue was dissolved in ethyl acetate (200 mL), washed with water (2 x 100 mL) and brine (100 mL), before drying over $MgSO_4$. The crude residue was purified by flash column chromatography (4% Et_2O in pentane) to give the title compound (19.8 g, 93%) as a white amorphous solid.

^{1}H NMR (400 MHz, $CDCl_3$) δ 7.03 (s, 4H, *H*-Ar), 4.44 (t, *J* = 6.4 Hz, 4H, *H*-33 & *H*-35), 2.88 (m, 6H, C*H*-*i*Pr), 2.19 (p, *J* = 6.4 Hz, 2H, *H*-34), 1.27 (dd, *J* = 6.9, 1.0 Hz, 36H, CH_3-*i*Pr). See spectrum.

^{13}C NMR (101 MHz, $CDCl_3$) δ 170.85 (*C*O-benzoate), 150.41 (*C*-*para*), 144.94 (*C*-*ortho*), 130.38 (*C*-*ipso*), 121.04 (*C*H-*meta*), 61.58 (*C*H_2-33 & *C*H_2-35), 34.58 (*C*H-*i*Pr *para*), 31.75 (*C*H-*i*Pr *ortho*), 28.34 (*C*H_2-34), 24.30 (*C*H_3-*i*Pr *ortho*), 24.08 (*C*H_3-*i*Pr *para*). See spectrum.

HRMS (m/z): (ESI) calculated for $C_{35}H_{52}O_4Na$ $[M+Na]^+$ 559.3758, found 559.3759.

TLC: *R*f = 0.48 (85:15 hexane:ethyl acetate, stained with ceric ammonium molybdate (CAM))

IR (ν_{max}/cm^{-1}, neat): 2961, 2930, 2908, 2870, 1727 (C=O), 1606, 1461, 1248, 1068.

(1*R*)-1-(*p*-tolylsulfinyl)propane-1,3-diyl bis(2,4,6-triisopropylbenzoate) – S10 and (1*S*)-1-(*p*-tolylsulfinyl)propane-1,3-diyl bis(2,4,6-triisopropylbenzoate) – S11

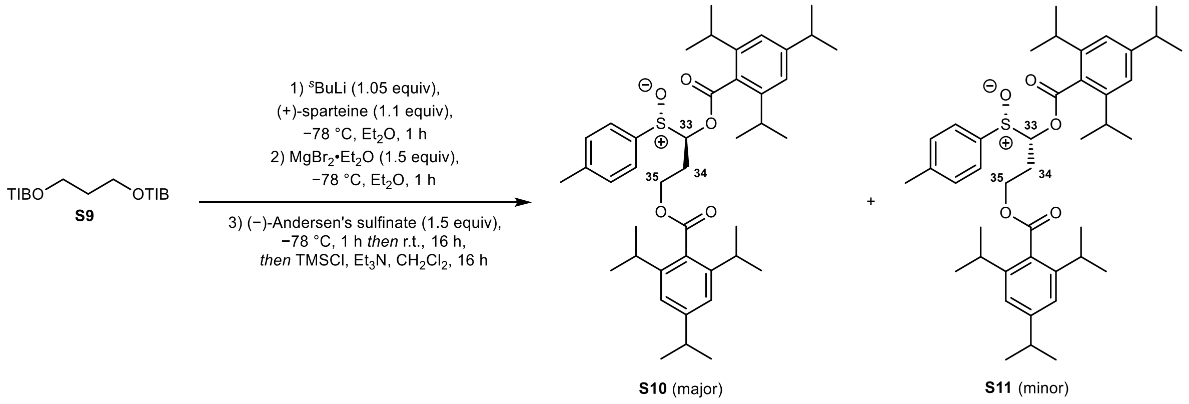

Prepared following General Procedure 1 using TIB ester **S9** (10.0 g, 18.6 mmol), (+)-sparteine (4.80 g, 20.5 mmol, 4.71 mL), *s*-BuLi (1.30 M in hexanes, 15.0 mL, 19.6 mmol), (−)-Andersen's sulfinate (8.23 g, 27.9 mmol), magnesium turnings (1.81 g, 74.5 mmol), dibromoethane (5.25 g, 27.9 mmol, 2.42 mL), Et_3N (2.82 g, 27.9 mmol, 3.89 mL), and

trimethylsilyl chloride (2.63 g, 24.2 mmol, 3.07 mL). The crude residue was purified by automated flash column chromatography (Biotage HC-200 g, *n*-hexane: ethyl acetate 0-10%) to give a mixture of diastereomers (9:1 *d.r.*) (11.2 g, 89%) as a white amorphous solid. The *syn* and *anti* diastereomers were inseparable by normal phase column chromatography. 2.10 g was purified portion-wise (*ca*. 80 mg injections) by reverse phase preparative HPLC to give:

Major *anti* diastereomer (**S10**) (1.80 g, 14% yield, 86% recovery, >99:1 *d.r.*) as a white amorphous solid.

^{1}H NMR (400 MHz, $CDCl_3$) δ 7.46 (d, *J* = 8.2 Hz, 2H, *H*-Ar), 7.22 (d, *J* = 7.8 Hz, 2H, *H*-Ar), 6.95 (d, *J* = 14.1 Hz, 4H, *H*-Ar), 6.05 (dd, *J* = 9.1, 3.6 Hz, 1H, *H*-33), 4.42 – 4.21 (m, 2H, *C*H_2-35), 2.90 – 2.62 (m, 6H, C*H*-*i*Pr), 2.33 (s, 3H, C*H_3*-tolyl), 2.31 – 2.21 (m, 1H, *H*-34'), 1.67 (ddt, *J* = 14.6, 9.1, 5.7 Hz, 1H, *C*H_2-34''), 1.25 – 1.07 (m, 36H, C*H_3*-*i*Pr). See spectrum.

^{13}C NMR (101 MHz, $CDCl_3$) δ 170.64 (*C*O-benzoate), 169.04 (*C*O-benzoate), 151.06 (*C*-TIB-*para*), 150.53 (*C*-TIB-*para*), 145.38 (*C*-TIB-*ortho*), 145.00 (*C*-TIB-*ortho*), 142.44 (*C*-tolyl-*ipso*), 135.95 (*C*-tolyl-*para*), 130.08 (*C*H-tolyl-*meta*), 130.02 (*C*-TIB-*ipso*), 128.76 (*C*-TIB-*ipso*), 125.64 (*C*H-tolyl-*ortho*), 121.24 (*C*H-TIB-*meta*), 121.04 (*C*H-TIB-*meta*), 84.99 (*C*H-33), 60.19 (*C*H_2-35), 34.59 (*C*H-*i*Pr *para*), 34.57 (*C*H-*i*Pr *para*), 31.95 (*C*H-*i*Pr *ortho*), 31.78 (*C*H-*i*Pr *ortho*), 27.26 (*C*H_2-34), 24.75 (*C*H_3-*i*Pr), 24.30 (*C*H_3-*i*Pr), 24.26 (*C*H_3-*i*Pr), 24.21 (*C*H_3-*i*Pr), 24.09 (*C*H_3-*i*Pr), 24.04 (*C*H_3-*i*Pr), 21.60 (*C*H_3-tolyl). See spectrum.

HRMS (m/z): (ESI) calculated for $C_{42}H_{58}O_5SNa$ $[M+Na]^+$ 697.3897, found 697.3876.

TLC: *R*$_f$ = 0.44 (90:10 pentane:Et_2O, stained with *p*-anisaldehyde).

IR (ν_{max}/cm^{-1}, neat): 2961, 2929, 2870, 1731 (C=O), 1606, 1461, 1384, 1248, 1039 (S=O).

$[\alpha]_D^{25}$: −22 (*c* = 1, $CHCl_3$).

Minor *syn* diastereomer (**S11**) (170 mg, 1.4% yield, 8% recovery) as a white amorphous solid in a 78:22 *dr* (*syn/anti*). Analysed without further purification.

^{1}H NMR (400 MHz, $CDCl_3$) δ 7.64 (d, *J* = 7.9 Hz, 2H, *H*-Ar), 7.33 (d, *J* = 8.2 Hz, 2H, *H*-Ar), 7.07 (s, 2H, *H*-Ar), 6.98 (s, 2H, *H*-Ar), 5.82 (dd, *J* = 10.3, 3.1 Hz, 1H, *H*-33), 4.41 – 4.27 (m, 2H, *C*H_2-35), 3.03 – 2.79 (m, 4H, C*H*-*i*Pr), 2.66 (hept, *J* = 6.8 Hz, 2H, C*H*-*i*Pr), 2.45 – 2.32 (m, 4H, C*H_3*-tolyl & *H*-34'), 2.15 – 2.07 (m, 1H, *H*-34''), 1.34 – 1.22 (m, 24H, C*H_3*-*i*Pr), 1.18 (d, *J* = 6.8 Hz, 6H, C*H_3*-*i*Pr), 1.13 (d, *J* = 6.8 Hz, 6H, C*H_3*-*i*Pr). See spectrum.

^{13}C NMR (101 MHz, CDCl$_3$) δ 170.44 (*C*O-benzoate), 170.29 (*C*O-benzoate), 151.15 (*C*-TIB-*para*), 150.39 (*C*-TIB-*para*), 145.49 (*C*-TIB-*ortho*), 144.95 (*C*-TIB-*ortho*), 141.89 (*C*-tolyl-*ipso*), 137.03 (*C*-tolyl-*para*), 130.28 (*C*H-tolyl-*meta*), 130.00 (*C*-TIB-*ipso*), 128.63 (*C*-TIB-*ipso*), 124.27 (*C*H-tolyl-*ortho*), 121.19 (*C*H-TIB-*meta*), 120.94 (*C*H-TIB-*meta*), 89.60 (*C*H-33), 60.16 (*C*H$_2$-35), 34.59 (*C*H-*i*Pr *para*), 34.52 (*C*H-*i*Pr *para*), 31.86 (*C*H-*i*Pr *ortho*), 31.69 (*C*H-*i*Pr *ortho*), 24.55 (*C*H$_3$-*i*Pr), 24.32 (*C*H$_3$-*i*Pr), 24.27 (*C*H$_3$-*i*Pr), 24.23 (*C*H$_3$-*i*Pr), 24.03 (*C*H$_3$-*i*Pr), 22.76 (*C*H$_3$-*i*Pr), 21.54 (*C*H$_2$-34). See spectrum.

HRMS (m/z): (ESI) calculated for $C_{42}H_{58}O_5SNa$ $[M+Na]^+$ 697.3897, found 697.3884.

TLC: $\boldsymbol{R_f}$ = 0.44 (90:10 pentane:Et_2O, stained with *p*-anisaldehyde).

IR (ν_{max}/cm^{-1}, neat): 2962, 2926, 2870, 1730 (C=O), 1606, 1460, 1363, 1233, 1033 (S=O).

LCMS trace to show the major **(S10)** and minor **(S11)** diastereomers upon trapping with (−)-Andersen's sulfinate showing a *d.r.* of *ca*. 9:1.

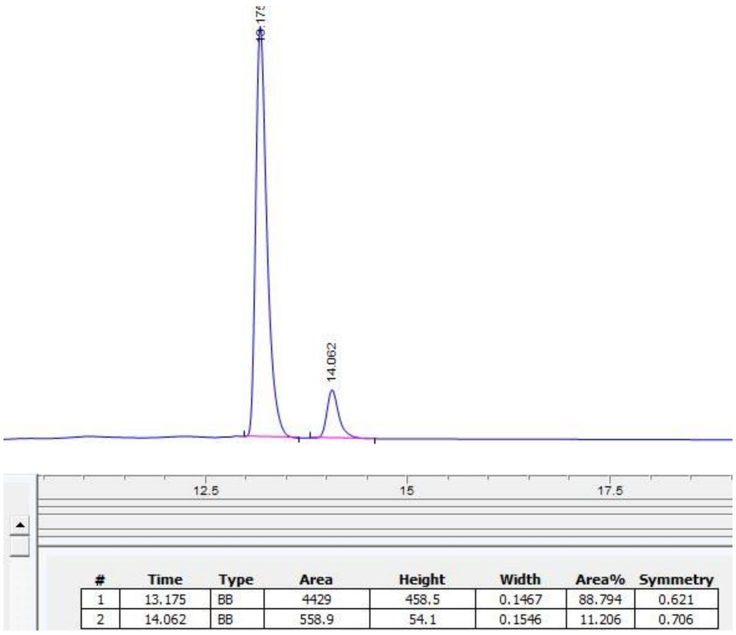

#	Time	Type	Area	Height	Width	Area%	Symmetry
1	13.175	BB	4429	458.5	0.1467	88.794	0.621
2	14.062	BB	558.9	54.1	0.1546	11.206	0.706

^{1}H NMR of *H*-33 for the major **(S10)** and minor **(S11)** diastereomers upon trapping with (−)-Andersen's sulfinate showing a *d.r.* of *ca*. 9:1. ^{1}H atoms at this position of *anti* α-sulfinyl benzoates possess higher chemical shifts relative to the corresponding *anti* α-sulfinyl benzoates and is diagnostic to assign relative stereochemical configuration at *H*-33.

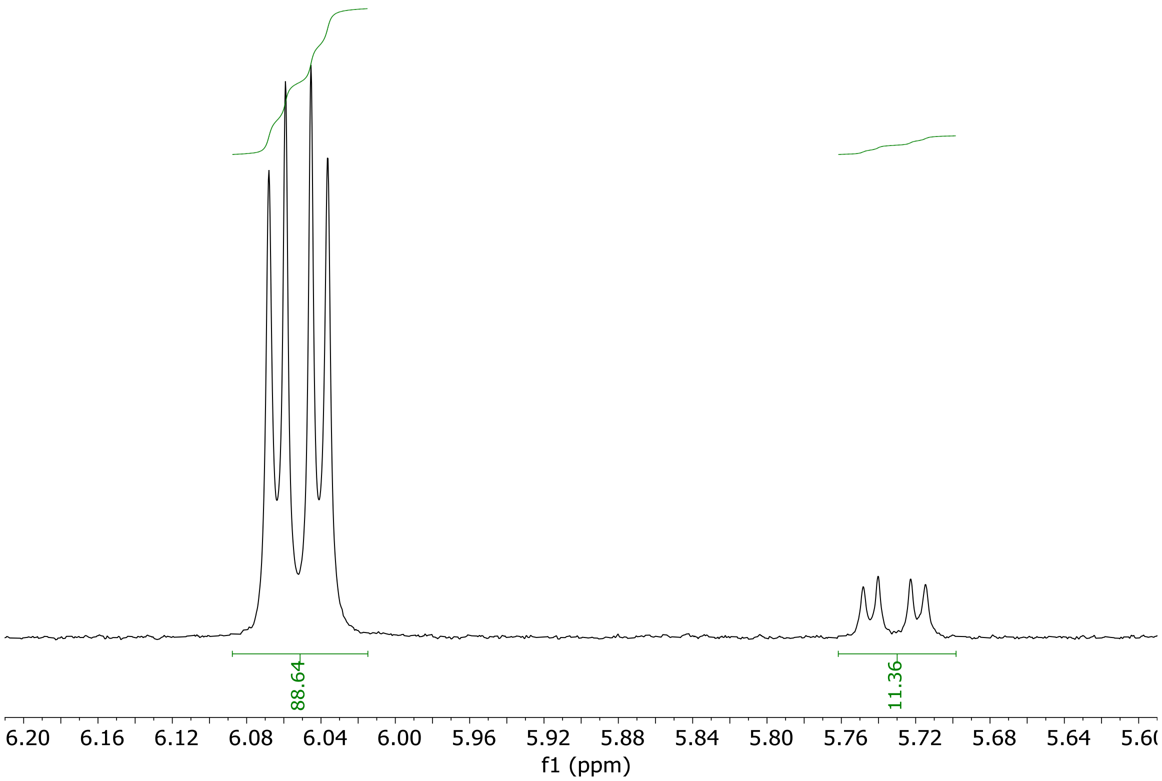

Reverse phase preparative HPLC purification enabled the isolation of **S10** with no minor diastereomer detected by ^{1}H NMR.

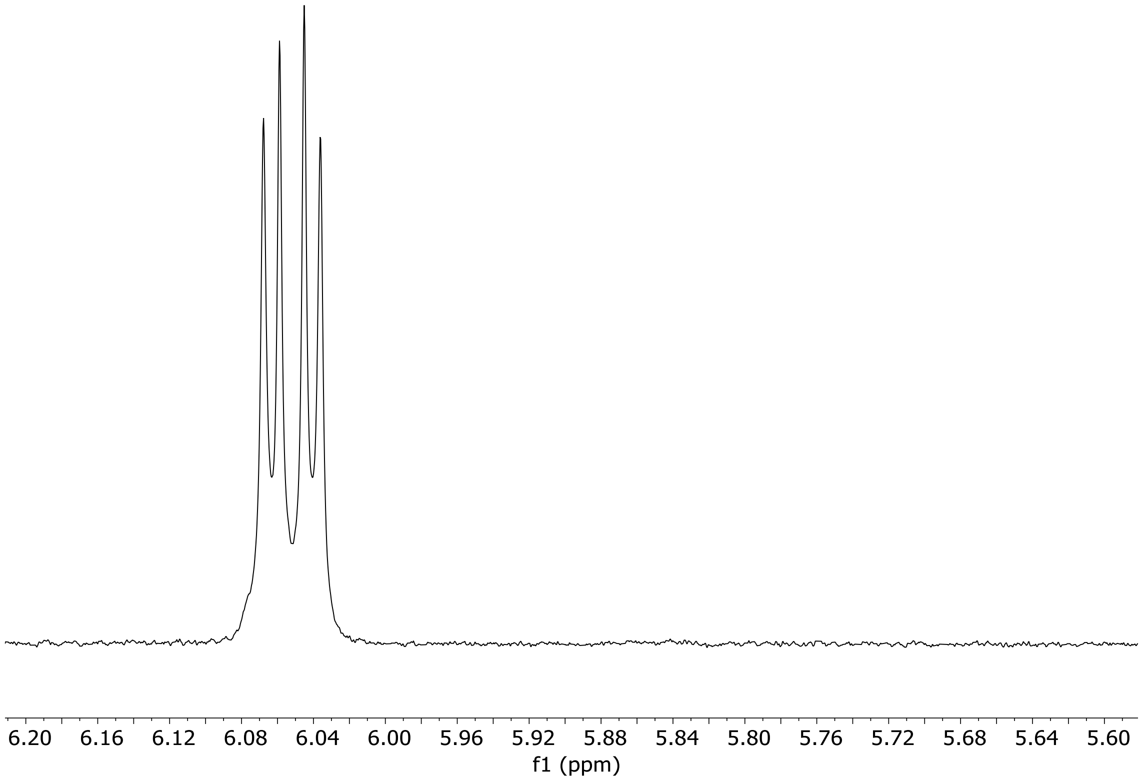

(*R*)-3-(4,4,5,5-Tetramethyl-1,3,2-dioxaborolan-2-yl)hex-5-en-1-yl 2,4,6-triisopropylbenzoate – S12

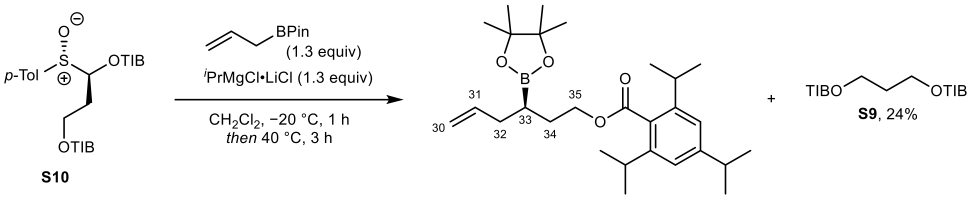

S12, 74%, >99:1 *d.r.*

*i*PrMgCl·LiCl (1.30 M in THF) (2.44 mL, 1.30 equiv) was added dropwise to a mixture of allylboronic acid pinacol ester (534 mg, 3.17 mmol, 1.00 equiv) and sulfoxide **S10** (1.65 g, 2.44 mmol, 1.10 equiv) in CH_2Cl_2 (12 mL, 0.20 M *wrt* boronic ester) at –20 °C and the resulting solution was stirred for 1 h at the same temperature (*pale yellow solution*). After warming to room temperature, the reaction mixture was heated at 40 °C for 3 h (*turbid white solution*). The reaction mixture was then cooled to room temperature and quenched with saturated aqueous NH_4Cl (5 mL). The aqueous phase was extracted with Et_2O (3 x 10 mL). The combined organic layers were dried over anhydrous Na_2SO_4 and filtered over a short pad of silica approximately 3 cm in depth, pre-mixed as a slurry with Et_2O:Et_3N (20 mL) 1% in order to deactivate the silica to remove residual TIB acid. The crude mixture was purified by normal phase flash column chromatography (Biotage HC-50 g, *n*-hexane: ethyl acetate 0-30%) to afford an inseperable mixture of the title compound and bis-TIB ester **S9** (1.15 g, 74% desired + 24% bis TIB ester). The mixture was taken through crude to the next step without further purification.

^{1}H NMR (400 MHz, $CDCl_3$) δ 6.99 (s, 2H, *H*-Ar), 5.79 (ddt, *J* = 17.0, 10.1, 6.9 Hz, 1H, *H*-31), 5.03 (ddd, *J* = 17.0, 2.5, 1.1 Hz, 1H, *H*-30 *cis*), 4.96 (ddt, *J* = 10.1, 2.1, 1.1 Hz, 1H, *H*-30 *trans*), 4.39 – 4.26 (m, 2H, *H*-35), 2.94 – 2.78 (m, *J* = 7.0 Hz, 3H, C*H*-*i*Pr), 2.30 – 2.12 (m, 2H, *H*-32), 1.91 – 1.72 (m, 2H, *H*-34), 1.27 – 1.23 (m, 18H, CH_3-*i*Pr), 1.22 (s, 12H, CH_3-Bpin). See spectrum.

^{13}C NMR (101 MHz, $CDCl_3$) δ 171.10 (*C*O-benzoate), 150.10 (*C*-TIB-*para*), 144.88 (*C*-TIB-*ortho*), 138.05 (*C*H-30), 130.95 (*C*-TIB-*ipso*), 120.93 (*C*H-TIB-*meta*), 115.57 (*C*H_2-31), 83.36 (*C*-Bpin), 64.58 (*C*-35), 35.27 (*C*H_2-32), 34.58 (*C*H-*i*Pr *para*), 31.59 (*C*H-*i*Pr *ortho*), 29.53 (*C*H_2-34), 24.98 (*C*H_3-Bpin), 24.91 (*C*H_3-Bpin), 24.30 (*C*H_3-*i*Pr), 24.11 (*C*H_3-*i*Pr). See spectrum.

Carbon next to boron (*C*-33) *not observed due to quadrupolar relaxation.*

HRMS (m/z): (ESI) calculated for $C_{28}H_{45}BO_4Na$ $[M+Na]^+$ 479.3303, found 479.3298.

TLC: $\boldsymbol{R_f}$ = 0.32 (90:10 pentane:Et_2O, stained with *p*-anisaldehyde).

IR (ν_{max}/cm^{-1}, neat): 2962, 2929, 2870, 1725 (C=O), 1607, 1462, 1380, 1323, 1250, 1140, 1075.

$[\alpha]_D^{25}$: −6 (*c* = 1, $CHCl_3$).

(*R*)-3,6-Bis(4,4,5,5-tetramethyl-1,3,2-dioxaborolan-2-yl)hexyl 2,4,6-triisopropylbenzoate – S13

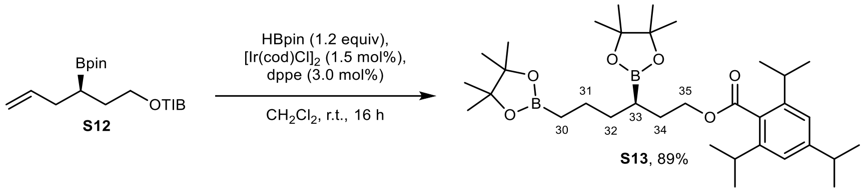

Prepared following General Procedure 3 using pinacolborane (345 mg, 0.391 mL, 2.69 mmol), alkene **S12** (820 mg, 1.80 mmol), $[Ir(cod)Cl]_2$ (18.1 mg, 26.9 μmol) and 1,2-bis(diphenylphosphino)ethane dppe (21.5 mg, 53.9 μmol). The reaction was quenched with methanol (1 mL) diluted with water (5 mL) and extracted with Et_2O (3 x 10 mL). The organic layers were dried over $MgSO_4$ and evaporated under reduced pressure. The crude mixture was purified by normal phase flash column chromatography (Biotage HC-50 g, pentane: Et_2O 0-25%) to afford the title compound (932 mg, 89%) as a colourless oil.

(*Starting material quantities calculated based on 72% by mass of the 1.14 g starting material being alkene* ***S12****, the remaining 28% was bis-TIB ester* ***S9*** *which was unreactive under iridium catalysed hydroboration conditions*)

^{1}H NMR (400 MHz, $CDCl_3$) δ 6.98 (s, 2H, *H*-Ar), 4.30 (t, *J* = 7.0 Hz, 2H, *H*-35), 2.87 (h, *J* = 6.9 Hz, 3H, C*H*-*i*Pr), 1.89 – 1.72 (m, 2H, CH_2-34), 1.52 – 1.33 (m, 4H, CH_2-32 and CH_2-31), 1.23 (42H, CH_3-*i*Pr and CH_3-Bpin), 1.17 – 1.07 (m, 1H, C*H*-33), 0.76 (t, *J* = 7.2 Hz, 2H, CH_2-30). See spectrum.

^{13}C NMR (101 MHz, $CDCl_3$) δ 171.11 (*C*O-benzoate), 150.03 (*C*-TIB-*para*), 144.88 (*C*-TIB-*ortho*), 131.01 (*C*-TIB-*ipso*), 120.90 (*C*H-TIB-*meta*), 83.15 (*C*-Bpin), 82.97 (*C*-Bpin), 64.87 (*C*-35), 34.58 (*C*H-*i*Pr *para*), 33.85 (*C*H_2-32), 31.56 (*C*H-*i*Pr *ortho*), 29.93 (*C*H_2-34), 24.95 (*C*H_3-Bpin), 24.87 (*C*H_3-Bpin), 24.33 (*C*H_3-*i*Pr), 24.30 (*C*H_3-*i*Pr), 24.11 (*C*H_3-*i*Pr), 23.58 (*C*H_2-31). See spectrum.

Carbons next to boron (*C*-30 & *C*-33) *not observed due to quadrupolar relaxation.*

HRMS (m/z): (MALDI) calculated for $C_{34}H_{58}B_2O_6Na$ $[M+Na]^+$ 607.4323, found 607.4319.

TLC: ***R*f** = 0.33 (90:10 pentane:Et_2O, stained with *p*-anisaldehyde).

IR (ν_{max}/cm^{-1}, neat): 2962, 2929, 2870, 1724 (C=O), 1607, 1462, 1371, 1319, 1250, 1143, 1076, 967.

$[\alpha]_D^{25}$: −2 (*c* = 1, $CHCl_3$).

(3*R*,7*S*)-3,7-Bis(4,4,5,5-tetramethyl-1,3,2-dioxaborolan-2-yl)dec-9-en-1-yl 2,4,6-triisopropylbenzoate – S14

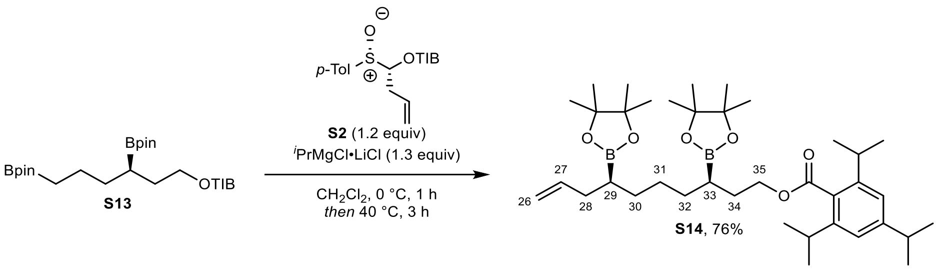

Prepared following General Procedure 2 using boronic ester **S13** (900 mg, 1.54 mmol), sulfoxide **S2** (814 mg, 1.84 mmol), and *i*PrMgCl·LiCl (1.20 M in THF, 1.67 mL, 2.00 mmol). The reaction mixture was then cooled to room temperature and was quenched with saturated aqueous NH_4Cl (5 mL). The aqueous phase was extracted with Et_2O (3 x 15 mL). The combined organic layers were dried over anhydrous Na_2SO_4 and filtered over a short pad of silica approximately 3 cm in depth, pre-mixed as a slurry with Et_2O:Et_3N (30 mL) 1% in order to deactivate the silica to remove residual TIB acid. The crude mixture was purified by normal phase flash column chromatography (Biotage HC-50 g, pentane: Et_2O 0-30%) to afford the title compound (745 mg, 76%) as a colourless oil.

^{1}H NMR (400 MHz, $CDCl_3$) δ 6.98 (s, 2H, *H*-Ar), 5.79 (ddt, *J* = 17.1, 10.2, 6.9 Hz, 1H, *H*-27), 4.99 (ddt, *J* = 17.1, 2.7, 1.5 Hz, 1H, *H*-26 *cis*), 4.91 (ddt, *J* = 10.1, 2.2, 1.1 Hz, 1H, *H*-26 *trans*), 4.30 (t, *J* = 7.0 Hz, 2H, *H*-35), 2.86 (hept, *J* = 7.0 Hz, 3H C*H*-*i*Pr), 2.13 (m, 2H, *H*-28), 1.87 – 1.71 (m, 2H, *H*-34), 1.51 – 1.27 (m, 6H, *H*-30, *H*-31, *H*-32), 1.24-1.21 (m, 42H, C*H*3-*i*Pr & C*H*3-Bpin), 1.16 – 0.99 (m, 2H, *H*-29 & *H*-33). See spectrum.

^{13}C NMR (101 MHz, $CDCl_3$) δ 171.10 (*C*O-benzoate), 150.05 (*C*-TIB-*para*), 144.88 (*C*-TIB-*ortho*), 138.84 (*C*H-26), 130.99 (*C*-TIB-*ipso*), 120.90 (*C*H-TIB-*meta*), 114.85 (*C*H_2-27), 83.16 (*C*-Bpin), 83.06 (*C*-Bpin), 64.84 (*C*-35), 35.51 (*C*H_2-28), 34.57 (*C*H-*i*Pr *para*), 31.56

(*C*H-*i*Pr *ortho*), 31.46 (*C*H_2-30 *or* *C*H_2-32), 31.20 (*C*H_2-30 *or* *C*H_2-32), 30.08 (*C*H_2-34), 28.58 (*C*H_2-31), 24.98 (*C*H_3-Bpin), 24.96 (*C*H_3-Bpin), 24.89 (*C*H_3-Bpin), 24.32 (*C*H_3-*i*Pr), 24.31 (*C*H_3-*i*Pr), 24.11 (*C*H_3-*i*Pr). See spectrum.

Carbons next to boron (*C*-29 & *C*-33) *not observed due to quadrupolar relaxation.*

HRMS (m/z): (MALDI) calculated for $C_{38}H_{64}B_2O_6Na$ $[M+Na]^+$ 661.4794, found 661.4787.

TLC: ***R*f** = 0.62 (85:15 pentane:Et_2O, stained with *p*-anisaldehyde).

IR (ν_{max}/cm^{-1}, neat): 2963, 2926, 2869, 1723 (C=O), 1606, 1462, 1380, 1317, 1250, 1141, 1076, 737.

$[\alpha]_D^{25}$: −2 (*c* = 1, $CHCl_3$).

(3*R*,7*R*)-3,7,10-Tris(4,4,5,5-tetramethyl-1,3,2-dioxaborolan-2-yl)decyl 2,4,6-triisopropylbenzoate – S15

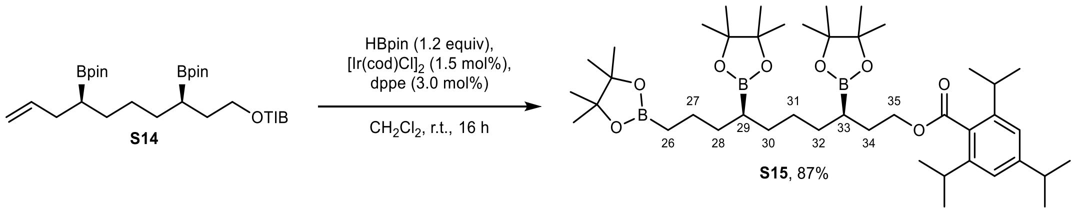

Prepared following General Procedure 3 using pinacolborane (210 mg, 239 µL, 1.64 mmol), alkene **S14** (700 mg, 1.10 mmol), $[Ir(cod)Cl]_2$ (11.1 mg, 16.4 µmol) and 1,2-bis(diphenylphosphino)ethane dppe (13.1 mg, 32.9 µmol). The reaction was quenched with methanol (0.5 mL) diluted with water (3 mL) and extracted with Et_2O (3 x 5 mL). The organic layers were dried over $MgSO_4$ and evaporated under reduced pressure. The crude mixture was purified by normal phase flash column chromatography (Biotage HC-50 g, pentane: Et_2O 0-25%) to afford the title compound (735 mg, 87%) as a colourless oil.

^{1}H NMR (400 MHz, $CDCl_3$) δ 6.98 (s, 2H, *H*-Ar), 4.29 (t, *J* = 7.0 Hz, 2H, *H*-35), 2.85 (hept, *J* = 7.0 Hz, 3H, C*H*-*i*Pr), 1.87 – 1.70 (m, 2H, C*H*$_2$-34), 1.50 – 1.27 (m, 10H, C*H*$_2$-27, 28, 30, 31 & 32), 1.27 – 1.15 (m, 54H, C*H*$_3$-*i*Pr and C*H*$_3$-Bpin), 1.15 – 1.02 (m, 1H, C*H*-29 *or* 33), 0.93 (s, 1H, C*H*-29 *or* 33), 0.78 – 0.69 (m, 2H, C*H*-26). See spectrum.

^{13}C NMR (101 MHz, $CDCl_3$) δ 171.10 (*C*O-benzoate), 150.02 (*C*-TIB-*para*), 144.88 (*C*-TIB-*ortho*), 131.01 (*C*-TIB-*ipso*), 120.89 (*C*H-TIB-*meta*), 83.13 (*C*-Bpin), 82.90 (*C*-Bpin), 82.84 (*C*-Bpin), 64.88 (*C*-35), 34.57 (*C*H-*i*Pr *para*), 34.24 (*C*H_2-28), 31.65 (*C*H_2-30 *or* 32), 31.59

(CH_2-30 *or* 32), 31.55 (*C*H-*i*Pr *ortho*), 30.11 (*C*H_2-34), 28.83 (*C*H_2-31), 24.95 (*C*H_3-), 24.88 (*C*H_3-Bpin), 24.32 (*C*H_3-*i*Pr), 24.30 (*C*H_3-*i*Pr), 24.11 (*C*H_3-*i*Pr), 23.79 (*C*H_2-27). See spectrum.

Carbons next to boron (*C*-26, *C*-29 & *C*-33) *not observed due to quadrupolar relaxation.*

HRMS (m/z): (MALDI) calculated for $C_{44}H_{77}B_3O_8Na$ $[M+Na]^+$ 789.5811, found 789.5821.

TLC: R_f = 0.44 (95:15 pentane:Et_2O, stained with *p*-anisaldehyde).

IR (ν_{max}/cm^{-1}, neat): 2974, 2927, 2870, 1725 (C=O), 1607, 1462, 1379, 1317, 1252, 1144, 1076, 968.

$[\alpha]_D^{25}$: 0 (*c* = 1, $CHCl_3$).

(3*R*,7*R*,11*S*)-3,7,11-Tris(4,4,5,5-tetramethyl-1,3,2-dioxaborolan-2-yl)tetradec-13-en-1-yl 2,4,6-triisopropylbenzoate – S16

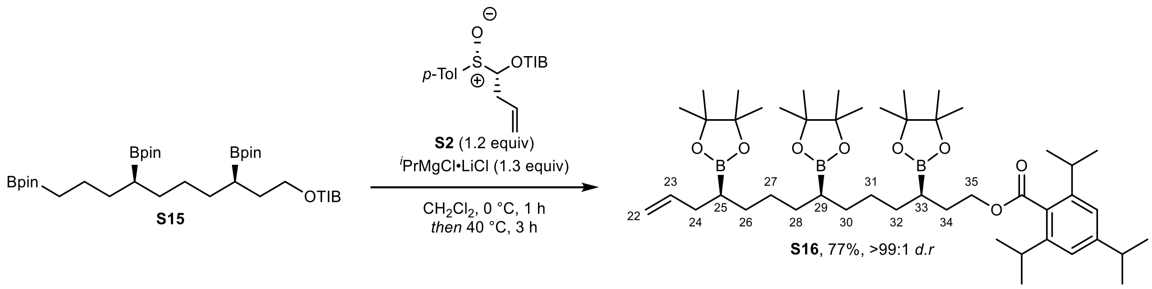

Prepared following General Procedure 2 using boronic ester **S15** (700 mg, 0.913 mmol), *syn* sulfoxide **S2** (482 mg, 1.10 mmol), *i*PrMgCl·LiCl (1.20 M in THF, 0.99 mL, 1.19 mmol). The reaction mixture was then cooled to room temperature and was quenched with saturated aqueous NH_4Cl (4 mL). The aqueous phase was extracted with Et_2O (3 x 12 mL). The combined organic layers were dried over anhydrous Na_2SO_4 and filtered over a short pad of silica approximately 3 cm in depth, pre-mixed as a slurry with Et_2O:Et_3N (30 mL) 1% in order to deactivate the silica to remove residual TIB acid. The crude mixture was purified by normal phase flash column chromatography (Biotage HC-50 g, pentane: Et_2O 0-40%) to afford the title compound (580 mg, 77%) as a colourless oil.

^{1}H NMR (400 MHz, $CDCl_3$) δ 6.98 (s, 2H, *H*-Ar), 5.79 (ddt, *J* = 17.0, 10.2, 6.9 Hz, 1H, *H*-23), 5.03 – 4.95 (m, 1H *H*-22 *cis*), 4.91 (ddt, *J* = 10.1, 2.3, 1.1 Hz, 1H, *H*-22 *trans*), 4.34 – 4.24 (m, 2H, , *H*-35), 2.94 – 2.78 (m, *J* = 6.9 Hz, 3H, C*H*-*i*Pr), 2.12 (m, 1.4 Hz, 2H, *H*-24), 1.87 – 1.69 (m, 2H, *H*-34), 1.50 – 1.26 (m, 12H, *H*-26, *H*-27, *H*-28, *H*-30, *H*-31 & *H*-32),

1.26 – 1.14 (m, 54H, CH_3-*i*Pr & CH_3-Bpin), 1.13 – 0.87 (m, 3H, *H*-25 *H*-29 & *H*-33). See spectrum.

^{13}C NMR (101 MHz, $CDCl_3$) δ 171.09 (*C*O-benzoate), 150.03 (*C*-TIB-*para*), 144.88 (*C*-TIB-*ortho*), 138.94 (*C*H-22), 131.02 (*C*-TIB-*ipso*), 120.90 (*C*H-TIB-*meta*), 114.76 (*C*H_2-23), 83.13 (*C*-Bpin), 83.01 (*C*-Bpin), 82.85 (*C*-Bpin), 64.88 (*C*-35), 35.58 (*C*H_2-24), 34.58 (*C*H-*i*Pr *para*), 31.75 (*C*H_2-26 *or* *C*H_2-28 *or* *C*H_2-30 *or* *C*H_2-32), 31.66 (*C*H_2-26 *or* *C*H_2-28 *or* *C*H_2-30 *or* *C*H_2-32), 31.62 (*C*H_2-26 *or* *C*H_2-28 *or* *C*H_2-30 *or* *C*H_2-32), 31.56 (*C*H-*i*Pr *ortho*), 31.31 (*C*H_2-26 *or* *C*H_2-28 *or* *C*H_2-30 *or* *C*H_2-32), 30.13 (*C*H_2-34), 28.81 (*C*H_2-27 *or* *C*H_2-31), 28.80 (*C*H_2-27 *or* *C*H_2-31), 25.00 (*C*H_3-Bpin), 24.98 (*C*H_3-Bpin), 24.96 (*C*H_3-), 24.89 (*C*H_3-Bpin), 24.33 (*C*H_3-*i*Pr), 24.31 (*C*H_3-*i*Pr), 24.12 (*C*H_3-*i*Pr). See spectrum.

Carbons next to boron (*C*-25, *C*-29 & *C*-33) *not observed due to quadrupolar relaxation.*

HRMS (m/z): (MALDI) calculated for $C_{48}H_{83}B_3O_8Na$ $[M+Na]^+$ 843.6282, found 843.6298.

TLC: $\boldsymbol{R_f}$ = 0.23 (90:10 hexane:ethyl acetate, stained with *p*-anisaldehyde).

IR (ν_{max}/cm^{-1}, neat): 2962, 2930, 2870, 1722 (C=O), 1606, 1462, 1369, 1312, 1251, 1138, 1076.

$[\boldsymbol{\alpha}]_{\mathbf{D}}^{\mathbf{25}}$: −6 (*c* = 1, $CHCl_3$).

(3*R*,7*R*,11*R*)-3,7,11,14-Tetrakis(4,4,5,5-tetramethyl-1,3,2-dioxaborolan-2-yl)tetradecyl 2,4,6-triisopropylbenzoate - 16

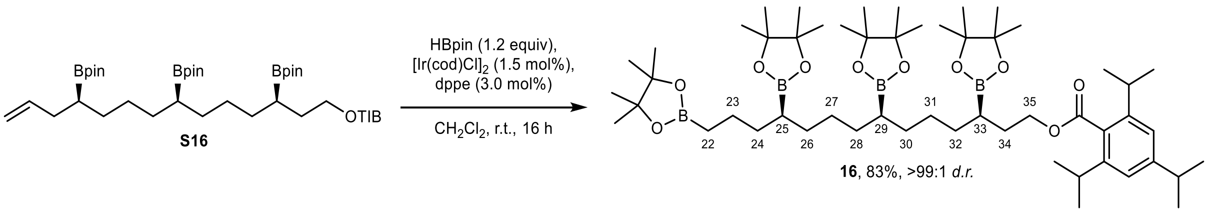

Prepared following General Procedure 3 using pinacolborane (128 mg, 146 µL, 1.01 mmol), alkene **S16** (550 mg, 0.670 mmol), $[Ir(cod)Cl]_2$ (6.8 mg, 10 µmol) and 1,2-bis(diphenylphosphino)ethane dppe (8.0 mg, 20 µmol). The reaction was quenched with methanol (0.3 mL) diluted with water (2 mL) and extracted with Et_2O (3 x 4 mL). The organic layers were dried over $MgSO_4$ and evaporated under reduced pressure. The crude mixture was purified by normal phase flash column chromatography (Biotage HC-50 g, pentane: Et_2O 10-35%) and then normal phase preparative HPLC (Teledyne ACCQPrep 150, hexane: ethyl acetate 10-35%) to afford the title compound (530 mg, 83%) as a colourless oil.

^{1}H NMR (400 MHz, CDCl$_3$) δ 6.98 (s, 2H, *H*-Ar), 4.29 (t, *J* = 7.0 Hz, 2H, *H*-35), 2.95 – 2.76 (m, *J* = 6.9 Hz, 3H, C*H*-*i*Pr), 1.78 (m, 2H, C*H*$_2$-34), 1.52 – 1.26 (m, 15H, C*H*$_2$-23, 24, 26, 27, 28, 30, 31 & 32), 1.26 – 1.14 (m, 66H, C*H*$_3$-*i*Pr & C*H*$_3$-Bpin), 1.14 – 0.92 (m, 3H, C*H*-25, 29 & 33), 0.79 – 0.67 (m, 2H, C*H*-22). See spectrum.

^{13}C NMR (101 MHz, CDCl$_3$) δ 171.09 (*C*O-benzoate), 150.02 (*C*-TIB-*para*), 144.89 (*C*-TIB-*ortho*), 131.03 (*C*-TIB-*ipso*), 120.89 (*C*H-TIB-*meta*), 83.12 (*C*-Bpin), 82.88 (*C*-Bpin), 82.82 (*C*-Bpin), 82.80 (*C*-Bpin), 64.89 (*C*-35), 34.58 (*C*H-*i*Pr *para*), 34.32 (*C*H$_2$-24), 31.81 (2*C*H$_2$-26, 28, 30 *or* 32), 31.75 (*C*H$_2$-26, 28, 30 *or* 32), 31.65 (*C*H$_2$-26, 28, 30 *or* 32), 31.55 (*C*H-*i*Pr *ortho*), 30.14 (*C*H$_2$-34), 29.09 (*C*H$_2$-27 *or* 31), 28.82 (*C*H$_2$-27 *or* 31) , 24.99 (*C*H$_3$-Bpin), 24.97 (*C*H$_3$-Bpin), 24.89 (*C*H$_3$-Bpin), 24.33 (*C*H$_3$-*i*Pr), 24.31 (*C*H$_3$-*i*Pr), 24.12 (*C*H$_3$-*i*Pr), 23.84 (*C*H$_2$-23). See spectrum.

Carbons next to boron (C-22, C-25, C-29 & C-33) not observed due to quadrupolar relaxation.

HRMS (m/z): (MALDI) calculated for $C_{54}H_{96}B_4O_{10}Na$ $[M+Na]^+$ 971.7299, found 971.7306.

TLC: ***R*$_f$** = 0.31 (90:10 hexane:ethyl acetate, stained with *p*-anisaldehyde).

IR (ν_{max}/cm^{-1}, neat): 2975, 2925, 2867, 1725 (C=O), 1606, 1462, 1379, 1315, 1251, 1144, 1076, 968.

$[\alpha]_D^{25}$: −6 (*c* = 1, CHCl$_3$).

Fragment 4 (17)

Ethyl 2,4,6-triisopropylbenzoate – S17

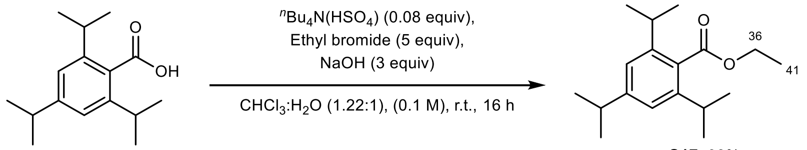

S17, 93%

According to a literature procedure.[13] A biphasic mixture of 2,4,6-triisopropylbenzoic acid (17.4 g, 70.0 mmol, 1.00 equiv), tetrabutylammonium hydrogen sulphate (1.90 g, 5.60 mmol, 8.00 mol%), sodium hydroxide (8.40 g, 210 mmol, 3.00 equiv), and ethyl bromide (38.1 g, 26.1 ml, 350 mmol, 5.00 equiv) in chloroform (350 mL) and water (286 mL) was stirred vigorously for 16 h at room temperature. The layers were separated, and the aqueous phase was extracted with CH_2Cl_2 (3 x 50 mL). The combined organic layers were washed with

brine (150 ml), dried over anhydrous $MgSO_4$ and then concentrated under reduced pressure to give the title compound (17.9 g, 93%) as a colourless oil.

All recorded spectroscopic data matched that previously reported[13]

^{1}H NMR (400 MHz, $CDCl_3$) δ 7.01 (s, 2H, *H*-Ar), 4.38 (q, *J* = 7.1 Hz, 2H, *H*-36), 2.95 – 2.81 (m, 3H, C*H*-*i*Pr), 1.37 (t, *J* = 7.1 Hz, 3H, *H*-41), 1.25 (dd, *J* = 6.9, 3.1 Hz, 18H, C*H*3-*i*Pr). See spectrum.

^{13}C NMR (101 MHz, $CDCl_3$) δ 170.97 (*C*O-benzoate), 150.21 (*C*-*para*), 144.87 (*C*-*ortho*), 130.78 (*C*-*ipso*), 120.97 (*C*H-*meta*), 60.90 (*C*H2-36), 34.58 (*C*H-*i*Pr *para*), 31.57 (*C*H-*i*Pr *ortho*), 24.25 (*C*H3-*i*Pr *ortho*), 24.10 (*C*H3-*i*Pr *para*), 14.40 (*C*-41). See spectrum.

(*S*)-1-(Trimethylstannyl)ethyl 2,4,6-triisopropylbenzoate - S18

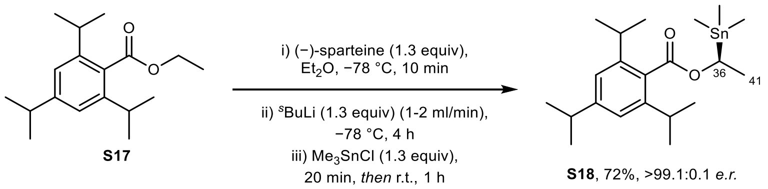

According to a literature procedure.[6b] To a solution of ethyl 2,4,6-triisopropylbenzoate **S17** (10.0 g, 36.2 mmol, 1.00 equiv) and (−)-sparteine (11.0 g, 10.8 mL, 47.0 mmol, 1.50 equiv) in Et_2O (181 mL, 0.30 M) was added *s*-BuLi (1.30 M solution in hexanes, 36.2 mL, 47.0 mmol, 1.30 equiv) dropwise (1-2 mL/min) at −78 °C and the reaction mixture was stirred at the same temperature for 3 h. Trimethyl tin chloride (1.00 M in hexane, 47.0 mL, 47.0 mmol, 1.30 equiv) was added dropwise (1-2 mL/min) to the reaction mixture at −78 °C. The reaction mixture was then stirred for 0.5 h at −78 °C before being warmed to room temperature and stirred for 1 h. The reaction was quenched by the addition of 2 M HCl aq. (100 mL) and extracted with ethyl acetate (200 mL). The organic layer was extracted with 2M HCl aq. (3 x 50 mL). The organic layer was concentrated under reduced pressure and the crude residue was purified by recrystallisation from hot MeOH (3 mL/g, over three crops) to afford the title compound (11.5 g, 72%, 99.9:0.1 *e.r.*) as a white crystalline solid.

All recorded spectroscopic data matched that previously reported[6b]

^{1}H NMR (400 MHz, $CDCl_3$) δ 6.99 (s, 2H, *H*-Ar), 5.04 (q, *J* = 7.6 Hz, 1H, *H*-36), 2.87 (m, 3H, C*H*-*i*Pr), 1.59 (d, *J* = 7.6 Hz, 3H, *H*-41), 1.24 (d, *J* = 6.9 Hz, 18H, C*H*3-*i*Pr), 0.18 (s, 9H, *H*3-SnC). *Sn satellite peaks ignored for simplicity.* See spectrum.

^{13}C NMR (101 MHz, $CDCl_3$) δ 171.30 (*C*O-benzoate), 149.93 (*C-para*), 144.83 (*C-ortho*), 130.80 (*C-ipso*), 120.80 (*C*H-*meta*), 67.04 (*C*H-36), 34.41 (*C*H-*i*Pr *para*), 31.36 (*C*H-*i*Pr *ortho*), 24.35 (CH$_3$-*i*Pr *ortho*), 24.10 (CH$_3$-*i*Pr *ortho*), 23.96 (CH$_3$-*i*Pr *para*), 19.22 (*C*-41), -9.89 (*C*Sn). See spectrum.

IR (ν_{max}/cm^{-1}, neat): 2978, 2956, 2926, 2872, 2857, 1463, 1370, 1312, 1229, 1141, 859.

HPLC on chiral stationary phase Daicel Chiralpak-IB column (25 cm), 100% *n*-hexane, flow rate: 0.9 mL/min., rt, 230 nm, Retention times: 9.00 min. (*S*, major) and 11.58 min. (*R*, minor).

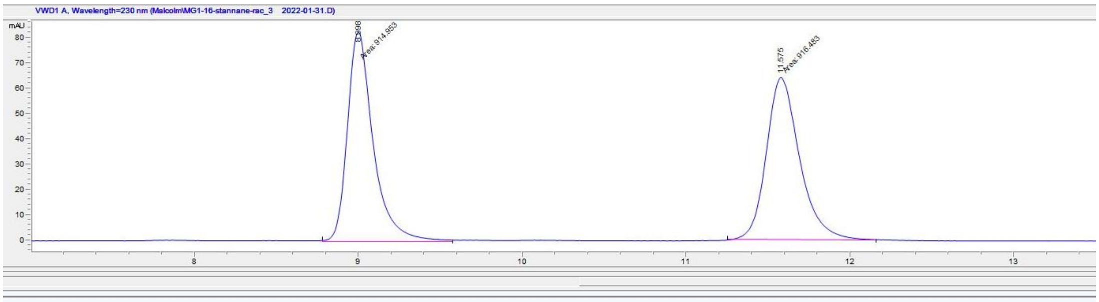

#	Time	Type	Area	Height	Width	Area%	Symmetry
1	8.998	MM	915	82.8	0.1842	49.958	0.685
2	11.575	MM	916.5	64.3	0.2376	50.042	0.768

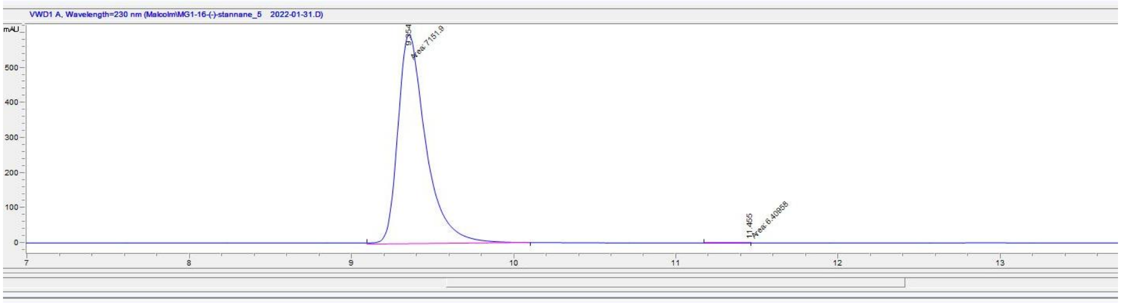

#	Time	Type	Area	Height	Width	Area%	Symmetry
1	9.354	MM	7151.9	594.2	0.2006	99.910	0.583
2	11.455	MM	6.4	7E-1	0.1529	0.090	2.88E-3

(*R*)-2-(Hexan-2-yl)-4,4,5,5-tetramethyl-1,3,2-dioxaborolane – (*R*)-17

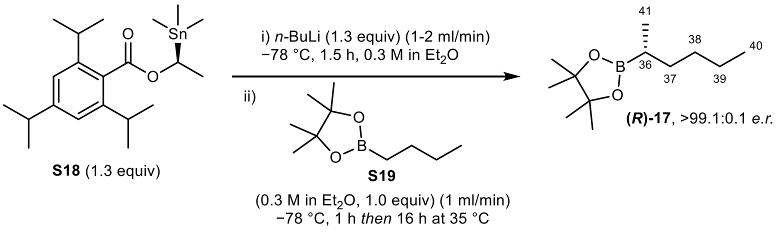

n-BuLi (1.30 M in hexanes, 11.0 mL, 17.7 mmol, 1.30 equiv) was added dropwise to a solution of stannane **S18** (7.75 g, 17.7 mmol, 1.30 equiv) in Et_2O (0.30 M) at −78 °C and the reaction mixture was stirred for 1.5 h at the same temperature. A solution of commercially available *n*-butylboronic acid pinacol ester **S19** (2.50 g, 2.87 mL, 13.6 mmol, 1.00 equiv) in

Et_2O (0.30 M) was added dropwise (1.00 mL/min) to the reaction mixture at −78 °C and the resulting mixture was stirred for 1 h at −78 °C, then it was warmed up to room temperature and heated for 16 h at 35 °C. Water (35 mL) and Et_2O (70 mL) were added and the mixture was separated. The organic layer was washed with water (3 x 35 mL) and dried over anhydrous $MgSO_4$. A portion of the material was purified by normal phase preparative HPLC eluting with hexane:ethyl acetate (0-3%) to afford the title compound as a colourless oil.

^{1}H NMR (400 MHz, $CDCl_3$) δ 1.51 – 1.39 (m, 1H, *H*-37'), 1.32 – 1.24 (m, 5H, C*H*$_2$-38, C*H*$_2$-39 & *H*-37'), 1.23 (s, 12H, C*H*$_3$-Bpin), 1.04 – 0.92 (m, 4H, C*H*-36 & C*H*$_3$-41), 0.90 – 0.84 (m, 3H, C*H*$_3$-40). See spectrum.

^{13}C NMR (101 MHz, $CDCl_3$) δ 82.89 (*C*-Bpin), 33.07 (*C*H$_2$-37), 31.40 (*C*H$_3$-38), 24.90 (C*H*$_3$-Bpin), 24.87 (C*H*$_3$-Bpin), 23.06 (*C*H$_3$-39), 15.66 (*C*H$_3$-41), 14.27 (*C*H$_3$-40). See spectrum.

HRMS (m/z): (EI) calculated for $C_{12}H_{25}BO_2$ $[M+H]^+$ 212.1942, found 212.1939.

$[\alpha]_D^{25}$: −2 (c = 1, $CHCl_3$).

(*R*)-1-(Trimethylstannyl)ethyl 2,4,6-triisopropylbenzoate – S20

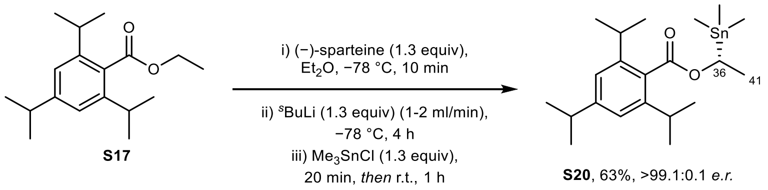

Prepared following the same procedure as described for (*S*) enantiomer **S18** using ethyl 2,4,6-triisopropylbenzoate **S17** (5.00 g, 18.1 mmol), (+)-sparteine (5.51 g, 5.40 mL, 23.5 mmol), trimethyltin chloride (1.00 M in hexanes, 23.5 mL, 23.5 mmol), and *s*-BuLi (1.30 M in hexanes, 18.1 mL, 23.5 mmol) in Et_2O (90 mL). The reaction was quenched by the addition of 2 M HCl aq. (50 mL) and extracted with ethyl acetate (100 mL). The organic layer was extracted with 2M HCl aq. (3 x 25 mL). The crude mixture was purified by recrystallisation from MeOH (3 mL/g, over 3 crops) to afford the title compound (4.97 g, 63%, 99.9:0.1 *e.r.*) as a white crystalline solid.

HPLC on chiral stationary phase Daicel Chiralpak-IB column (25 cm), 100% *n*-hexane, flow rate: 0.9 mL/min., rt, 230 nm, Retention times: 9.00 min. (*S*, major) and 11.58 min. (*R*, minor).

Both ¹H and ¹³C spectroscopic data was identical to that of its enantiomer **S18**

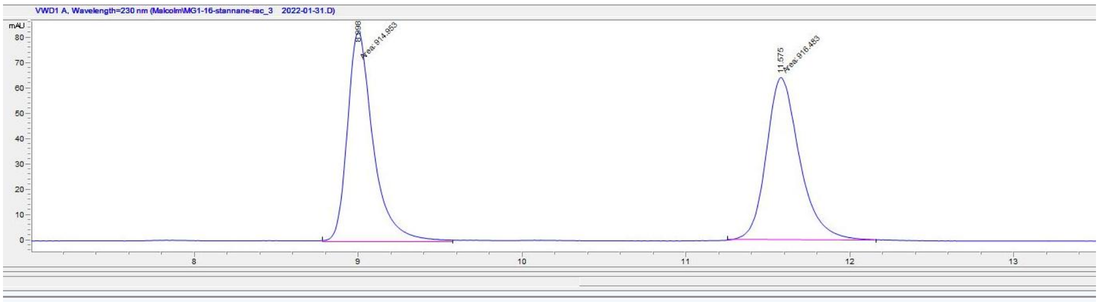

#	Time	Type	Area	Height	Width	Area%	Symmetry
1	8.998	MM	915	82.8	0.1842	49.958	0.685
2	11.575	MM	916.5	64.3	0.2376	50.042	0.768

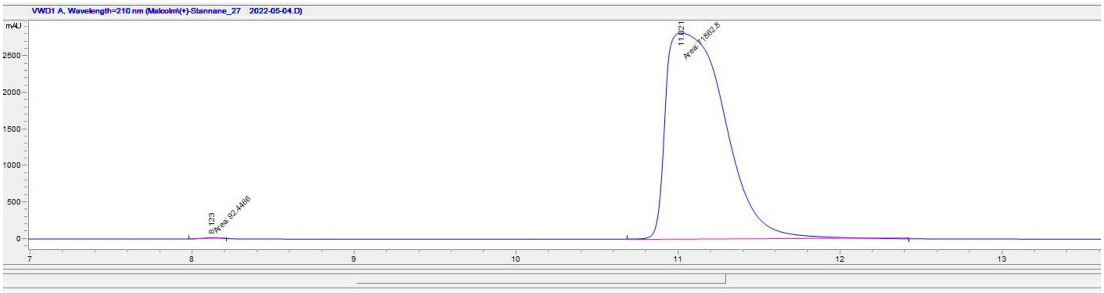

#	Time	Type	Area	Height	Width	Area%	Symmetry
1	8.123	MM	92.4	13.6	0.1134	0.128	1.466
2	11.021	MM	71862.8	2833	0.4228	99.872	0.348

(*S*)-2-(Hexan-2-yl)-4,4,5,5-tetramethyl-1,3,2-dioxaborolane – (*S*)-17

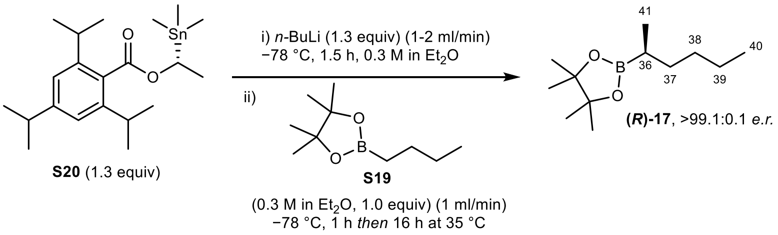

Prepared following the same procedure as described for (*R*) enantiomer **S19** using stannane **S20** (4.65 g, 10.6 mmol), *n*-butylboronic acid pinacol ester **S19** (1.50 g, 1.72 mL, 8.15 mmol), and *n*-BuLi (1.6 M in hexanes, 6.62 mL, 10.6 mmol). Water (20 mL) and Et_2O (45 mL) were added and the mixture was separated. The organic layer was washed with water (3 x 20 mL) and dried over anhydrous $MgSO_4$. A portion of the material was purified by normal phase preparative HPLC eluting with hexane:ethyl acetate (0-3%) to afford the title compound as a colourless oil.

*The (S)-enantiomer **(S)-17** had identical ¹H and ¹³C spectroscopic data as its enantiomer **(R)-17***

HRMS (m/z): (EI) calculated for $C_{12}H_{25}BO_2$ $[M+H]^+$ 212.1942, found 212.1939.

$[\alpha]_D^{25}$: 2 (c = 1, $CHCl_3$).

Caylobolide B fragment coupling

2-((6*S*)-6-((*S*)-7-((4*R*,6*S*)-6-allyl-2,2-dimethyl-1,3-dioxan-4-yl)-4-hydroxyheptyl)-2,2-dimethyl-1,3-dioxan-4-yl)ethyl 2,4,6-triisopropylbenzoate – S22

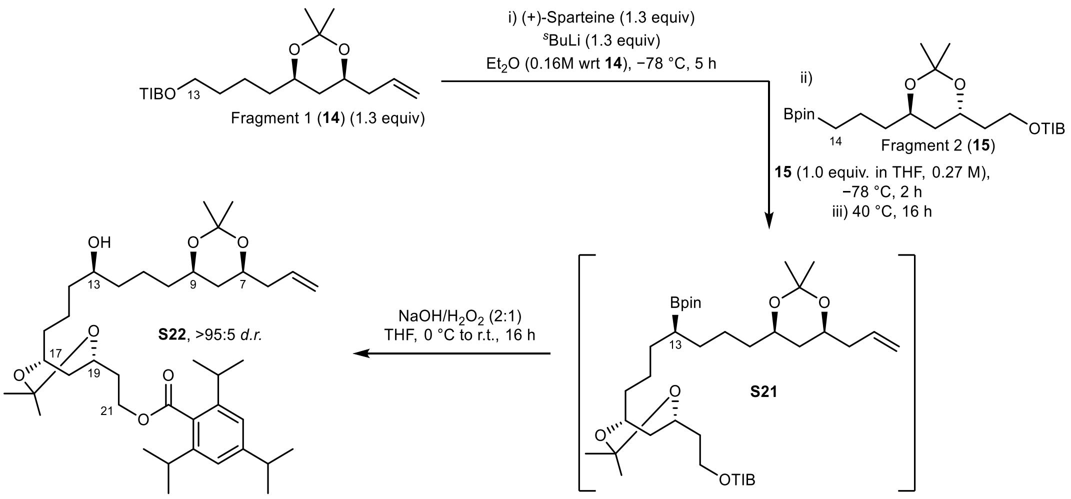

To a solution of **14** (299 mg, 0.501 mmol, 1.30 equiv) and (+)-sparteine (138 mg, 136 µL, 0.652 mmol, 1.30 equiv) in Et_2O (4.1 mL, 0.16 M) was added *s*-BuLi (1.42 M in hexanes) (469 µL, 0.652 mmol, 1.30 equiv) dropwise at −78 °C and the reaction mixture was stirred at the same temperature for 5 h. A solution of **15** (280 mg, 0.501 mmol) in THF (0.27 M, 1.86 mL) was then added dropwise (1 mL/min) to the reaction mixture at −78 °C and the reaction mixture was stirred for 2 h at −78 °C. After this time the reaction mixture was heated to 40 °C for 16 h. A 2:1 solution of aqueous sodium hydroxide (3 M) and 30 % aqueous hydrogen (3 mL total volume) was added directly to the reaction mixture at 0 °C, and the reaction mixture was then stirred at r.t. for 16 h. The reaction mixture was quenched by the addition of saturated sodium thiosulfate (2 mL) and was extracted with Et_2O (3 x 20 mL). The organic layers were combined, and washed with brine (5 mL), then dried over anhydrous Na_2SO_4 and concentrated under reduced pressure. The crude residue was purified by automated flash column chromatography (Biotage HC-10 g, pentane: Et_2O 0-50%) to afford the title compound (281 mg, 85%) as a colourless oil.

^{1}H NMR (600 MHz, $CDCl_3$) δ 7.00 (s, 2H, *H*-Ar), 5.80 (ddt, *J* = 17.2, 10.2, 7.0 Hz, 1H, *H*-5), 5.13 – 5.01 (m, 2H, *H*-4-alkene), 4.45 – 4.31 (m, 2H, *H*-21), 3.94 (tdd, *J* = 9.3, 6.0, 4.4 Hz, 1H, *H*-19), 3.90 – 3.83 (m, 1H, *H*-7), 3.80 (m, 2H, *H*-9 & *H*-17), 3.59 (dt, *J* = 7.6, 4.3 Hz,

1H, *H*-13), 2.86 (3H, C*H*-*i*Pr), 2.30 (ddd, *J* = 12.6, 6.3, 6.3, 1H, *H*-6'), 2.14 (ddd, *J* = 14.1, 7.0, 7.0 Hz, 1H, *H*-6''), 1.95 – 1.82 (m, 2H, *H*-20), 1.68 – 1.56 (m, 2H, *H*-12), 1.56 – 1.43 (m, 15H, *H*-8' & *H*-10 & *H*-11 & *H*-12 & *H*-14 & *H*-15 & *H*-16 & *H*-18), 1.43 (s, 3H, CH_3-acetonide), 1.39 (s, 3H, CH_3-acetonide), 1.34 (s, 3H, CH_3-acetonide), 1.33 (s, 3H, CH_3-acetonide), 1.24 (m, 18H, CH_3-*i*Pr), 1.18 – 1.08 (m, 1H, *H*-8''). See spectrum.

^{13}C NMR (151 MHz, $CDCl_3$) δ 171.05 (*C*O-benzoate), 150.28 (*C*-*para*), 144.84 (*C*-*ortho*), 134.37 (*C*H-5 alkene), 130.63 (*C*-*ipso*), 120.99 (*C*-*meta*), 117.19 (*C*H_2-4 alkene), 100.53 (CO *anti*-acetonide), 98.58 (CO *syn*-acetonide), 71.74 (*C*H-13), 69.02 (*C*H-9), 68.79 (*C*H-7), 66.74 (*C*H-17), 63.63 (*C*H-19), 61.83 (*C*H_2-21), 41.01 (*C*H_2-6), 38.82 (*C*H-18), 37.46 (*C*H_2-10 *or* CH_2-12 *or* CH_2-14 *or* CH_2-16 *or* CH_2-18), 37.37 (*C*H_2-10 *or* CH_2-12 *or* CH_2-14 *or* CH_2-16 *or* CH_2-18), 36.52 (*C*H_2-8), 36.42 (*C*H_2-10 *or* CH_2-12 *or* CH_2-14 *or* CH_2-16 *or* CH_2-18), 35.88 (*C*H_2-10 *or* CH_2-12 *or* CH_2-14 *or* CH_2-16 *or* CH_2-18), 35.02 (*C*H_2-20), 34.55 (*C*H-*i*Pr *para*), 31.65 (*C*H-*i*Pr *ortho*), 30.38 (*C*H_3-*syn*-acetonide), 24.84 (*C*H_3-*anti*-acetonide), 24.80 (*C*H_3-*anti*-acetonide), 24.35 (*C*H_3-*i*Pr), 24.24 (*C*H_3-*i*Pr), 24.08 (*C*H_3-*i*Pr), 21.79 (*C*-15), 21.19 (*C*-11), 19.95 (*C*H_3-*syn*-acetonide). See spectrum.
HRMS (m/z): (ESI) calculated for $C_{40}H_{67}O_7$ $[M+H]^+$ 659.4881, found 659.4869.

TLC: *R*f Fragment 1 (**14**) = 0.57, ***R*f** Fragment 2 (**15**) = 0.37 ***R*f S21** = 0.27, ***R*f S22** = 0.05 (90:10 hexane:ethyl acetate, stained with *p*-anisaldehyde).

IR (ν_{max}/cm^{-1}, neat): 3455 (OH), 3074, 2960, 2938, 2868, 1727 (C=O), 1643 (C=C), 1607, 1379, 1225, 1138, 1033.

$[\alpha]_D^{25}$: +12 (*c* = 1, $CHCl_3$).

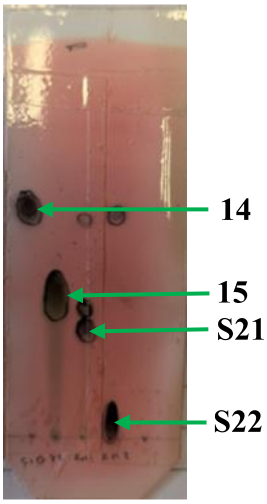

2-((6*S*)-6-((*S*)-7-((4*R*,6*S*)-6-allyl-2,2-dimethyl-1,3-dioxan-4-yl)-4-((triethylsilyl)oxy)heptyl)-2,2-dimethyl-1,3-dioxan-4-yl)ethyl 2,4,6-triisopropylbenzoate – S23

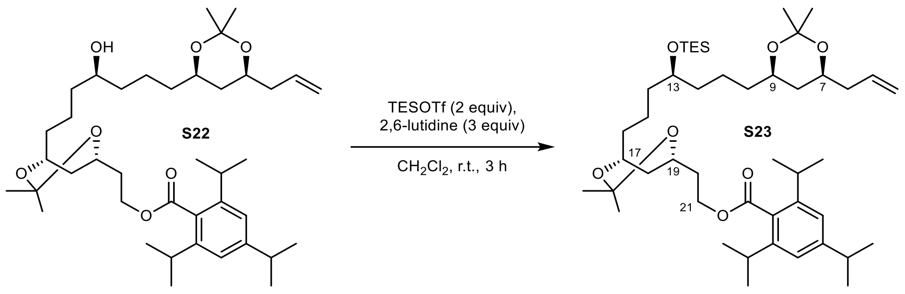

Prepared following General Procedure 5 using alcohol **S22** (270 mg, 0.410 mmol), TESOTf (217 mg, 186 µL, 0.819 mmol), and 2,6-lutidine (130 mg, 141 µL, 1.23 mmol). Sat. aq. $NaHCO_3$ (3 mL) was added, and the layers were separated. The aqueous phase was extracted with CH_2Cl_2 (3 x 6 mL). The combined organic layers were dried over anhydrous Na_2SO_4, filtered and concentrated under reduced pressure. The crude mixture was purified by normal phase flash column chromatography (Biotage HC-10 g, pentane:ethyl acetate 0-10%) to afford the title compound (303 mg, 96%) as a colourless oil.

^{1}H NMR (500 MHz, $CDCl_3$) δ 7.00 (s, 2H, *H*-Ar), 5.80 (ddt, *J* = 17.2, 10.0, 6.9 Hz, 1H, *H*-5), 5.13 – 5.00 (m, 2H, *H*-4-alkene), 4.45 – 4.32 (m, 2H, *H*-21), 4.01 – 3.91 (m, 1H, *H*-19), 3.86 (dtd, *J* = 12.2, 6.3, 2.4 Hz, 1H, *H*-7), 3.78 (m, 2H, *H*-9 & *H*-17), 3.63 (h, *J* = 5.0 Hz, 1H, *H*-13), 2.86 (dp, *J* = 23.7, 6.9 Hz, 3H, C*H*-*i*Pr), 2.31 (dtd, *J* = 14.0, 6.2, 1.5 Hz, 1H, *H*-6'), 2.20 – 2.10 (m, 1H, *H*-6''), 1.96 – 1.81 (m, 2H, *H*-20), 1.60 (m, 2H, *H*-12), 1.55 – 1.26 (m, 27H, *H*-8' & *H*-10 & *H*-11 & *H*-12 & *H*-14 & *H*-15 & *H*-16 & *H*-18 & CH_3-acetonide), 1.24 (d, *J* = 7.0, 18H, CH_3-*i*Pr), 1.16 – 1.07 (m, 1H, *H*-8''), 1.00 – 0.92 (m, 9H, CH_3-OTES), (m, 6H, CH_2-OTES). See spectrum.

^{13}C NMR (126 MHz, $CDCl_3$) δ 171.06 (*C*O-benzoate), 150.28 (*C*-*para*), 144.85 (*C*-*ortho*), 134.40 (*C*H-5 alkene), 130.65 (*C*-*ipso*), 120.99 (*C*-*meta*), 117.16 (*C*H_2-4 alkene), 100.49 (CO *anti*-acetonide), 98.55 (CO *syn*-acetonide), 72.31 (*C*H-13), 69.00 (*C*H-9), 68.81 (*C*H-7), 66.70 (*C*H-17), 63.63 (*C*H-19), 61.87 (*C*H_2-21), 41.05 (*C*H_2-6), 38.87 (*C*H-18), 37.29 (*C*H_2-10 *or* CH_2-12 *or* CH_2-14 *or* CH_2-16), 37.12 (*C*H_2-10 *or* CH_2-12 *or* CH_2-14 *or* CH_2-16), 36.71 (*C*H_2-8), 36.59 (*C*H_2-10 *or* CH_2-12 *or* CH_2-14 *or* CH_2-16 *or* CH_2-18), 36.20 (*C*H_2-10 *or* CH_2-12 *or* CH_2-14 *or* CH_2-16 *or* CH_2-18), 35.04 (*C*H_2-20), 34.57 (*C*H-*i*Pr *para*), 31.66 (*C*H-*i*Pr *ortho*), 30.40 (*C*H_3-*syn*-acetonide), 24.84 (*C*H_3-*anti*-acetonide), 24.72 (*C*H_3-*anti*-acetonide),

24.35 (CH_3-*i*Pr), 24.24 (CH_3-*i*Pr), 24.09 (CH_3-*i*Pr), 21.62 (*C*-15), 20.88 (*C*-11), 19.95 (CH_3-*syn*-acetonide), 7.11 (OTES-CH_3), 5.24 (OTES-CH_2). See spectrum.

HRMS (m/z): (ESI) calculated for $C_{46}H_{80}O_7SiNa$ $[M+Na]^+$ 795.5566, found 795.5563.

TLC: R_f = 0.57 (90:10 hexane:ethyl acetate, stained with *p*-anisaldehyde, product visible under UV lamp).

IR (ν_{max}/cm^{-1}, neat): 3075, 2958, 2939, 2874, 1727 (C=O), 1643 (C=C), 1607, 1461, 1379, 1225, 1137, 1076, 1017 (Si-O), 742 (Si-C).

$[\alpha]_D^{25}$: −10 (*c* = 1, $CHCl_3$).

(1R)-2-((6*S*)-6-((*S*)-7-((4*R*,6*S*)-6-allyl-2,2-dimethyl-1,3-dioxan-4-yl)-4-((triethylsilyl)oxy)heptyl)-2,2-dimethyl-1,3-dioxan-4-yl)-1-(p-tolylsulfinyl)ethyl 2,4,6-triisopropylbenzoate – 22

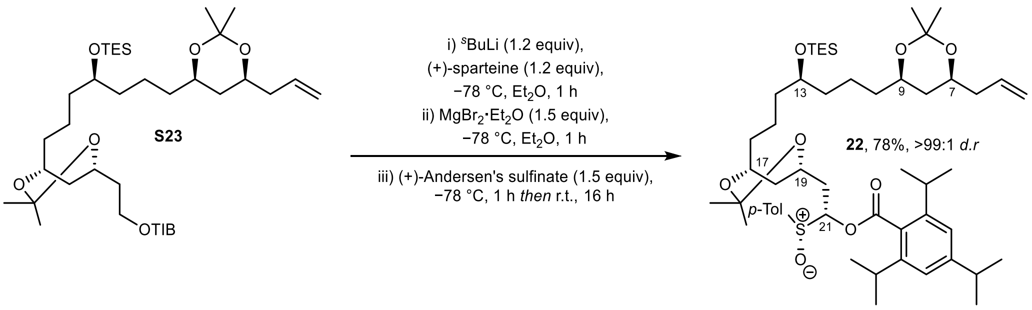

Prepared following General Procedure 1 using **S23** (300 mg, 0.388 mmol), (+)-sparteine (109 mg, 107 µL, 0.466 mmol), *s*-BuLi (1.29 M in hexanes, 360 µL, 0.466 mmol), (+)-Andersen's sulfinate (171 mg, 0.582 mmol), magnesium turnings (75.4 mg, 3.10 mmol), dibromoethane (219 mg, 101 µL, 1.16 mmol). The reaction mixture was quenched with water (3 mL), the layers separated, and the organic layer washed with water (3 x 3 mL). The combined aqueous phases were washed with Et_2O (3 x 10 mL), then the combined organic layers were washed with saturated $NaHCO_3$ and brine, dried over anhydrous Na_2SO_4, filtered, and concentrated under reduced pressure.

Sparteine was not recovered through acidification due to risk of cleaving the acid labile TES and acetonide protecting groups.

Et_3N (59 mg, 81 µL, 0.58 mmol) and trimethylsilyl chloride (55 mg, 64 µL, 0.50 mmol). The reaction mixture was then diluted with Et_2O (10 mL) and water (5 mL), the layers separated, and the aqueous layer extracted with Et_2O (2 x 10 mL). The organic layers were dried over

anhydrous Na_2SO_4, filtered and concentrated under reduced pressure. The crude residue was purified by normal phase preparative HPLC (hexane: ethyl acetate 0-20%) to afford the title compound (276 mg, 78%) as a colourless oil.

^{1}H NMR (600 MHz, $CDCl_3$) δ 7.65 (d, *J* = 8.2 Hz, 2H, *H*-Ar), 7.39 – 7.32 (m, 2H, *H*-Ar), 7.04 (s, 2H, *H*-Ar), 5.85 – 5.74 (m, 2H, *H*-5 & *H*-21), 5.11 – 5.01 (m, 2H, *H*-4-alkene), 3.85 (dtd, *J* = 11.5, 6.3, 2.4 Hz, 1H, *H*-7), 3.77 (dddd, *J* = 11.7, 6.9, 5.1, 2.4 Hz, 1H, *H*-9), 3.64 (ddd, *J* = 10.0, 6.2, 3.2 Hz, 1H, *H*-17), 3.60 (q, *J* = 5.5 Hz, 1H, *H*-13), 3.50 (ddt, *J* = 9.4, 7.8, 6.0 Hz, 1H, H-19), 2.96 (h, *J* = 6.8 Hz, 2H, C*H*-*i*Pr *ortho*), 2.91 (h, *J* = 7.0 Hz, 1H, C*H*-*i*Pr *para*), 2.42 (s, 3H, CH_3-tolyl), 2.30 (dtt, *J* = 14.0, 6.2, 1.5 Hz, 1H, *H*-6'), 2.13 (dddd, *J* = 18.2, 10.9, 6.2, 3.5 Hz, 2H, *H*-6'' & *H*-20'), 1.98 (ddd, *J* = 14.9, 8.0, 6.7 Hz, 1H, *H*-20''), 1.52 – 1.30 (m, 20H, *H*-8' & *H*-10 & *H*-11 & *H*-12 & *H*-14 & *H*-15 & *H*-16 & *H*-18 & CH_3-acetonide), 1.30 – 1.24 (m, 22H, CH_3-*i*Pr & CH_3-acetonide), 1.22 (s, 3H, CH_3-acetonide), 1.11 (m, 1H, *H*-8''), 1.00 (s, 3H, CH_3-acetonide), 0.94 (t, *J* = 7.9 Hz, 9H, CH_3-OTES), 0.57 (q, *J* = 7.9 Hz, 6H, CH_2-OTES). See spectrum.

^{13}C NMR (151 MHz, $CDCl_3$) δ 170.21 (*C*O-benzoate), 151.04 (*C*H-TIB-*para*), 145.43 (*C*H-TIB-*ortho*), 141.68 (*C*-tolyl-*ipso*), 137.54 (*C*-tolyl-*para*), 134.35 (*C*H-5 alkene), 130.19 (*C*H-tolyl-*meta*), 128.79 (*C*H-TIB-*ipso*), 124.40 (*C*H-tolyl-*ortho*), 121.12 (TIB-*C*H-*meta*), 117.12 (CH_2-4 alkene), 100.41 (CO *anti*-acetonide), 98.50 (CO *syn*-acetonide), 90.06 (*C*H-21), 72.23 (*C*H-13), 68.95 (*C*H-9), 68.77 (*C*H-7), 66.39 (*C*H-17), 63.87 (*C*H-19), 41.01 (CH_2-6), 38.55 (CH_2-18), 37.24 (CH_2-10 *or* CH_2-12 *or* CH_2-14 *or* CH_2-16), 37.05 (CH_2-10 *or* CH_2-12 *or* CH_2-14 *or* CH_2-16), 36.67 (CH_2-8), 36.55 (CH_2-10 *or* CH_2-12 *or* CH_2-14 *or* CH_2-16), 36.09 (CH_2-10 *or* CH_2-12 *or* CH_2-14 *or* CH_2-16), 34.57 (*C*H-*i*Pr *para*), 31.64 (*C*H-*i*Pr *ortho*),, 30.36 (CH_3-*syn*-acetonide), 30.26 (CH_2-20), 24.50 (CH_3-*anti*-acetonide), 24.46 (CH_3-*i*Pr), 24.38 (CH_3-*i*Pr), 24.32 (CH_3-*anti*-acetonide), 24.02 (CH_3-*i*Pr), 21.56 (*C*-15), 21.53 (CH_3-tolyl), 20.85 (*C*-11), 19.91 (CH_3-*syn*-acetonide), 7.06 (OTES-CH_2), 5.19 (OTES-CH_2). See spectrum.

HRMS (m/z): (nanospray) calculated for $C_{53}H_{86}O_8SSiNa$ $[M+Na]^+$ 933.5710, found 933.5720.

TLC: ***R*f** = 0.47 (90:10 hexane:ethyl acetate, stained with *p*-anisaldehyde).

IR (ν_{max}/cm^{-1}, neat): 3075, 2960, 2937, 2871, 1736 (C=O), 1642 (C=C), 1606, 1460, 1379, 1230, 1044 (S=O), 1017 (Si-O), 740 (Si-C).

$[\alpha]_D^{25}$: −62 (*c* = 1, $CHCl_3$).

(3*R*,7*R*,11*S*,15R)-16-((4*R*,6*S*)-6-((R)-7-((4*R*,6*S*)-6-allyl-2,2-dimethyl-1,3-dioxan-4-yl)-4-((triethylsilyl)oxy)heptyl)-2,2-dimethyl-1,3-dioxan-4-yl)-3,7,11,15-tetrakis(4,4,5,5-tetramethyl-1,3,2-dioxaborolan-2-yl)hexadecyl 2,4,6-triisopropylbenzoate – S24

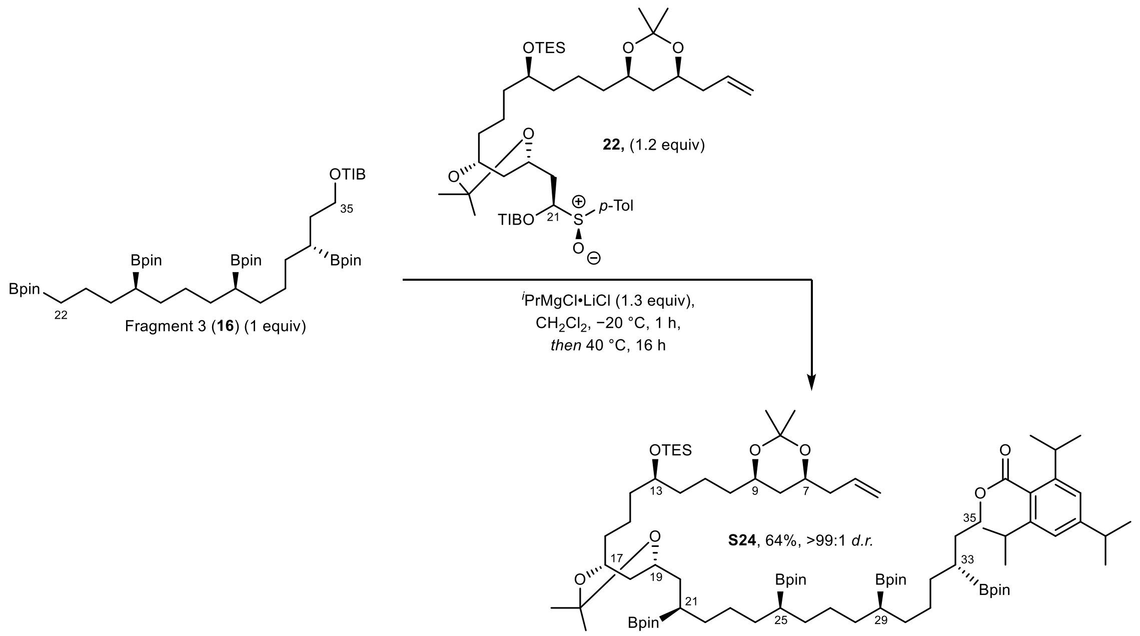

Prepared following General Procedure 2 using **16** (108 mg, 0.113 mmol), sulfoxide **22** (125 mg, 0.124 mmol), and *i*PrMgCl·LiCl (1.3 M in THF, 148 µL, 0.192 mmol). The reaction mixture was then cooled to room temperature and was quenched with saturated aqueous NH_4Cl (2 mL). The aqueous phase was extracted with Et_2O (3 x 4 mL). The combined organic layers were dried over anhydrous Na_2SO_4 and filtered over a short pad of silica approximately 2 cm in depth, pre-mixed as a slurry with Et_2O:Et_3N (20 mL) 1% in order to deactivate the silica to remove residual TIB acid. The crude mixture was purified by normal phase flash column chromatography (Biotage HC-10 g, hexane: ethyl acetate 0-10%) to afford the title compound (108 mg, 64%) as a colourless oil.

^{1}H NMR (600 MHz, $CDCl_3$) δ 6.98 (s, 2H, *H*-Ar), 5.85 – 5.76 (m, 1H, *H*-5 alkene), 5.11 – 5.02 (m, 2H, *H*-4 alkene), 4.29 (td, *J* = 6.8, 2.1 Hz, 2H, *H*-35), 3.89 – 3.83 (m, 1H, *H*-7), 3.81 – 3.68 (m, 3H, *H*-9, *H*-17, *H*-19), 3.67 – 3.58 (m, 1H, *H*-13), 2.91 – 2.81 (m, 3H, C*H*-*i*Pr), 2.37 – 2.25 (m, 1H, *H*-6’), 2.17 – 2.11 (m, 1H, *H*-6’’), 1.85 – 1.71 (m, 2H, *H*-34), 1.66 – 1.05 (m, 114H, *C*H_2-alkyls, C*H*$_3$-acetonide, C*H*$_3$-*i*Pr & C*H*$_3$-Bpin), 0.95 (t, *J* = 7.9 Hz, 9H, C*H*$_3$-OTES), 0.58 (q, *J* = 7.9 Hz, 6H, C*H*$_2$-OTES). See spectrum.

^{13}C NMR (126 MHz, $CDCl_3$) δ 171.05 (*C*O-benzoate), 149.98 (*C*-*para*), 144.83 (*C*-*ortho*), 134.37 (*C*H-5 alkene), 130.98 (*C*-*ipso*), 120.85 (*C*-*meta*), 117.12 (*C*H_2-4 alkene), 100.29 (CO

anti-acetonide), 98.51 (CO *syn*-acetonide), 83.08 (*C*-Bpin), 82.88 (*C*-Bpin), 82.78 (*C*-Bpin), 82.77 (*C*-Bpin), 72.34 (*C*H-13), 68.97 (*C*H-9), 68.78 (*C*H-7), 66.78 (*C*H-17), 66.34 (*C*H-19), 64.84 (*C*H-35), 41.02 (CH_2-6), 39.39 (*C*H-18), 37.60 (CH_2-12, 14, 16 *or* 20), 37.26 (CH_2-12, 14, 16 *or* 20), 37.15 (CH_2-12, 14, 16 *or* 20), 36.68 (CH_2-12, 14, 16 *or* 20), 36.56 (CH_2-8), 36.24 (CH_2-10), 34.54 (*C*H-*i*Pr *para*), 32.00 (CH_2-22, 24, 26, 28, 30 *or* 32), 31.86 (CH_2-22, 24, 26, 28, 30 *or* 32), 31.81 (CH_2-22, 24, 26, 28, 30 *or* 32), 31.79 (CH_2-22, 24, 26, 28, 30 *or* 32), 31.70 (CH_2-22, 24, 26, 28, 30 *or* 32), 31.60 (CH_2-22, 24, 26, 28, 30 *or* 32), 31.52 (*C*H-*i*Pr *ortho*), 30.37 (CH_3-*syn*-acetonide), 30.08 (CH_2-34), 29.02 (CH_2-23 *or* 27 *or* 31), 28.79 (CH_2-23 *or* 27 *or* 31), 28.70 (CH_2-23 *or* 27 *or* 31), 25.12 (CH_3), 24.95 (CH_3), 24.94 (CH_3), 24.93 (CH_3), 24.89 (CH_3), 24.86 (CH_3), 24.84 (CH_3), 24.81 (CH_3), 24.30 (CH_3-*i*Pr), 24.28 (CH_3-*i*Pr), 24.09 (CH_3-*i*Pr), 21.70 (*C*-15), 20.85 (*C*-11), 19.93 (CH_3-*syn*-acetonide), 7.08 (OTES-CH_3), 5.21 (OTES-CH_2). See spectrum.

HRMS (m/z): (nanospray) calculated for $C_{84}H_{152}B_4O_{15}SiNa$ $[M+Na]^+$ 1496.1170, found 1496.1201.

TLC: R_f = 0.57 (90:10 hexane:ethyl acetate, stained with *p*-anisaldehyde).

IR (ν_{max}/cm^{-1}, neat): 2973, 2923, 2866, 1725 (C=O), 1606, 1461, 1380, 1315, 1252, 1144, 1033, 854.

$[\alpha]_D^{25}$: −8 (*c* = 1, $CHCl_3$).

(3*R*,7*R*,11*S*,15R)-16-((4*R*,6*S*)-6-((R)-7-((4*R*,6*S*)-6-allyl-2,2-dimethyl-1,3-dioxan-4-yl)-4-((triethylsilyl)oxy)heptyl)-2,2-dimethyl-1,3-dioxan-4-yl)-3,7,11,15-tetrakis(4,4,5,5-tetramethyl-1,3,2-dioxaborolan-2-yl)hexadecyl 2,4,6-triisopropylbenzoate - 23

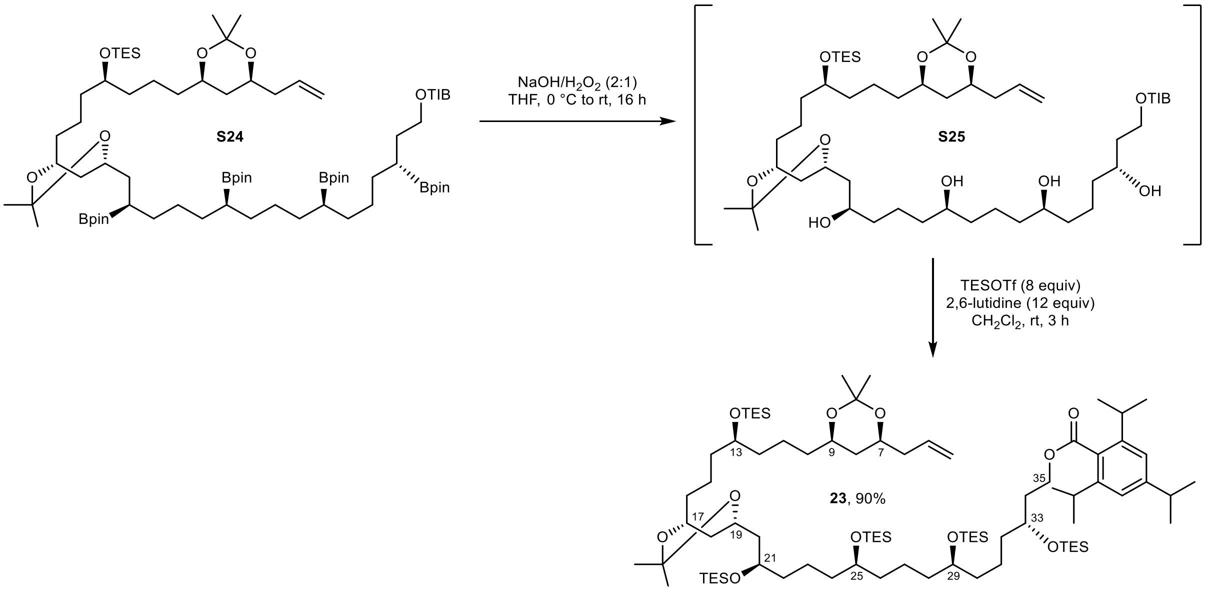

Prepared following General Procedure 4 using tetraboronic ester **S24** (108 mg, 73.3 μmol). The crude material containing **S25** was silyl protected according to General Procedure 5 using, TESOTf (155 mg, 133 μL, 0.586 mmol) and 2,6-lutidine (94.2 mg, 1.57 mmol, 101 μL). Sat. aq. $NaHCO_3$ (2 mL) was added, and the layers were separated. The aqueous phase was extracted with CH_2Cl_2 (3 x 4 mL). The combined organic layers were dried over anhydrous Na_2SO_4, filtered and concentrated under reduced pressure. The crude mixture was purified by normal phase flash column chromatography (Biotage HC-10 g, pentane: ethyl acetate 0-10%) to afford the title compound (97 mg, 90%) as a colourless oil.

^{1}H NMR (600 MHz, $CDCl_3$) δ 7.00 (s, 2H, *H*-Ar), 5.86 – 5.74 (m, 1H, *H*-5 alkene), 5.12 – 5.02 (m, 2H, *H*-4 alkene), 4.42 (ddd, *J* = 10.9, 7.5, 5.7 Hz, 1H, *H*-35'), 4.34 (ddd, *J* = 10.8, 7.2, 7.2 Hz, 1H, *H*-35''), 3.95 – 3.70 (m, 6H, *H*-7, *H*-9, *H*-17, *H*-19, *H*-21, *H*-33), 3.68 – 3.57 (m, 3H, *H*-13, *H*-25, *H*-29), 2.30 (dddd, *J* = 13.6, 8.8, 6.8, 3.6 Hz, 1H, *H*-6'), 2.18 – 2.11 (m, 1H, *H*-6''), 1.93 – 1.79 (m, 2H, *H*-34), 1.73 (ddd, *J* = 13.5, 7.7, 5.6 Hz, 1H, *H*-18'), 1.60 – 1.26 (m, 47H, CH_2-alkyls, CH_3-acetonide, CH_3-*i*Pr), 1.24 (d, *J* = 6.9 Hz, 18H, CH_3-*i*Pr), 1.00 – 0.91 (m, 45H, CH_3-OTES), 0.64 – 0.55 (m, 30H, CH_2-OTES). See spectrum.

^{13}C NMR (151 MHz, $CDCl_3$) δ 171.02 (*C*O-benzoate), 150.15 (*C-para*), 144.88 (*C-ortho*), 134.38 (*C*H-5 alkene), 130.75 (*C-ipso*), 120.93 (*C-meta*), 117.14 (*C*H_2-4 alkene), 100.17 (CO *anti*-acetonide), 98.53 (CO *syn*-acetonide), 72.52 (*C*H-25), 72.44 (*C*H-29), 72.36 (*C*H-13), 69.54 (*C*H-33), 69.18 (*C*H-21), 69.00 (*C*H-9), 68.81 (*C*H-7), 66.68 (*C*H-17), 63.76 (*C*H-19),

62.30 (*C*H-35), 43.78 (*C*H_2-20), 41.05 (*C*H_2-6), 39.33 (α-*C*H_2), 38.08 (α-*C*H_2), 37.75 (α-*C*H_2), 37.69 (α-*C*H_2), 37.67 (α-*C*H_2), 37.62 (α-*C*H_2), 37.60 (α-*C*H_2), 37.30 (α-*C*H_2), 37.17 (α-*C*H_2), 36.71 (α-*C*H_2), 36.58 (α-*C*H_2), 36.27 (α-*C*H_2), 36.11 (α-*C*H_2), 34.55 (*C*H-*i*Pr *para*), 31.63 (*C*H-*i*Pr *ortho*), 30.38 (*C*H_3-*syn*-acetonide), 24.86 (*C*H_3-*anti*-acetonide), 24.81 (*C*H_3-*anti*-acetonide), 24.35 (*C*H_3-*i*Pr), 24.21 (*C*H_3-*i*Pr), 24.08 (*C*H_3-*i*Pr), 21.70 (β-*C*H_2), 21.47 (β-*C*H_2), 21.29 (β-*C*H_2), 21.11 (β-*C*H_2), 20.88 (β-*C*H_2), 19.93 (*C*H_3-*syn*-acetonide), 7.11 (OTES-*C*H_3), 7.09 (OTES-*C*H_3), 7.03 (OTES-*C*H_3), 5.28 (OTES-*C*H_2), 5.27 (OTES-*C*H_2), 5.23 (OTES-*C*H_2), 5.20 (OTES-*C*H_2). *See spectrum*.

HRMS (m/z): (nanospray) calculated for $C_{84}H_{164}O_{11}Si_5Na$ $[M+Na]^+$ 1512.1018, found 1512.1030.

TLC: R_f = 0.29 (95:5 hexane:ethyl acetate, stained with *p*-anisaldehyde)

IR (ν_{max}/cm^{-1}, neat): 2954, 2913, 2876, 1728 (C=O), 1642 (C=C), 1607, 1460, 1378, 1240, 1075, 1007 (Si-O), 740 (Si-C).

$[\alpha]_D^{25}$: −10 (*c* = 1, $CHCl_3$).

Iso-caylobolide B (2) final steps

(5*S*,6*S*,8*R*,12*R*,16*S*,20R)-21-((4*S*,6*S*)-6-((*S*)-7-((4*R*,6*S*)-6-allyl-2,2-dimethyl-1,3-dioxan-4-yl)-4-((triethylsilyl)oxy)heptyl)-2,2-dimethyl-1,3-dioxan-4-yl)-5-methyl-8,12,16,20-tetrakis((triethylsilyl)oxy)henicosan-6-ol – 24

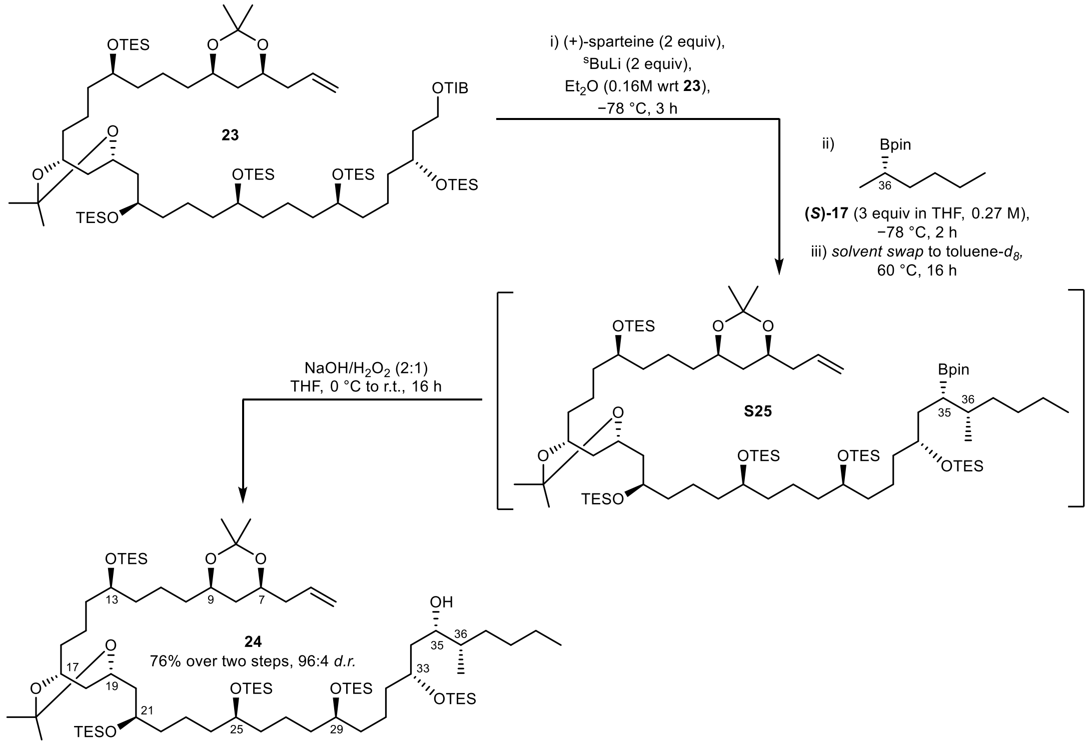

To a solution of **23** (60 mg, 40 μmol, 1.00 equiv) and (+)-sparteine (19 mg, 18 μL, 81 μmol, 2.0 equiv) in Et_2O (250 μL, 0.16 M) was added *s*-BuLi (1.31 M in hexanes, 61 μL, 81 μmol, 2.0 equiv) dropwise at −78 °C and the reaction mixture was stirred at the same temperature for 3 h. A solution of **(*S*)-17** (26 mg, 0.24 mmol) in THF (0.27 M, 450 μL) was added dropwise (1 mL/min) to the reaction mixture at –78 °C. The reaction mixture was then stirred for 2 h at −78 °C before the reaction mixture was warmed to room temperature, the solvent removed under vacuum and exchanged with toluene-d_8. After this time the reaction mixture was heated to 40 °C for 16 h. A 2:1 solution of aqueous sodium hydroxide (3 M) and 30 % aqueous hydrogen (0.5 mL total volume) was added directly to the reaction mixture at 0 °C, and the reaction mixture was then stirred at r.t. for 16 h. The reaction mixture was quenched by the addition of saturated sodium thiosulfate (0.5 mL) and was extracted with ethyl acetate (5 x 1 mL). The organic layers were combined, and washed with brine (1 mL), then dried over anhydrous Na_2SO_4 and concentrated under reduced pressure. The crude residue was purified by normal phase flash column chromatography (Biotage HC-25 g, pentane: ethyl acetate 0-15%) to afford the title compound (41 mg, 76% over two steps) as a colourless oil.

^{1}H NMR (600 MHz, $CDCl_3$) δ 5.85 – 5.74 (m, 1H, *H*-5 alkene), 5.08 (ddt, *J* = 17.2, 1.7, 1.6 Hz, 1H, *H*-4' *trans* alkene), 5.04 (ddt, *J* = 10.2, 2.1, 1.1 Hz, 1H, *H*-4' *cis* alkene), 4.02 – 3.96 (m, 1H, *H*-33), 3.92 – 3.87 (m, 1H, *H*-19), 3.88 – 3.81 (m, 2H, *H*-7 & *H*-35), 3.81 – 3.70 (m, 3H, *H*-9, *H*-17 & *H*-21), 3.67 – 3.59 (m, 3H, *H*-13, *H*-25 & *H*-29), 3.24 (d, *J* = 2.2 Hz, 1H, O-*H*), 2.30 (ddddd, *J* = 12.6, 6.2, 6.2, 1.4, 1.4 Hz, 1H, *H*-6'), 2.14 (ddddd, *J* = 14.1, 7.7, 6.5, 1.3, 1.3 Hz, 1H, *H*-6''), 1.73 (ddd, *J* = 13.5, 7.7, 5.7 Hz, 1H, *H*-20'), 1.65 (ddd, *J* = 14.3, 10.6, 3.7 Hz, 1H, *H*-34'), 1.62 – 1.42 (m, 16H, CH_2-alkyls), 1.42 (s, 3H, CH_3-*syn* acetonide), 1.42 – 1.38 (m, 12H, CH_2-alkyls & *H*-36), 1.38 (s, 3H, CH_3-*syn* acetonide), 1.38 – 1.32 (m, 9H, CH_2-alkyls), 1.31 (s, 3H, CH_3-*anti* acetonide), 1.31 (s, 3H, CH_3-*anti* acetonide), 1.30 – 1.18 (m, 7H, CH_2-alkyls), 1.14 – 1.07 (m, 2H, *H*-37' & C*H*'), 0.98 – 0.93 (m, 45H, CH_3-OTES), 0.90 – 0.87 (m, 6H, CH_3-40 & CH_3-41), 0.64 – 0.55 (m, 30H, CH_2-OTES). See spectrum.

^{13}C NMR (151 MHz, $CDCl_3$) δ 134.40 (*C*H-5 alkene), 117.15 (*C*H_2-4 alkene), 100.19 (CO *anti*-acetonide), 98.55 (CO *syn*-acetonide), 72.52 (*C*H-25), 72.38 (*C*H-29 & *C*H-13), 71.95 (*C*H-33), 71.63 (*C*H-35), 69.18 (*C*H-21), 69.01 (*C*H-9), 68.82 (*C*H-7), 66.69 (*C*H-17), 63.77 (*C*H-19), 43.79 (*C*H_2-20), 41.05 (*C*H_2-6), 39.34 (α-*C*H_2), 39.09 (*C*H-36), 38.22 (*C*H_2-34), 37.78 (α-*C*H_2), 37.69 (α-*C*H_2), 37.67 (α-*C*H_2), 37.57 (α-*C*H_2), 37.40 (α-*C*H_2), 37.31 (α-*C*H_2), 37.18 (α-*C*H_2), 36.72 (α-*C*H_2), 36.62 (α-*C*H_2), 36.59 (α-*C*H_2), 36.28 (α-*C*H_2), 32.68 (*C*H_2-37), 30.39 (*C*H_3-*syn*-acetonide), 29.88 (*C*H_2-38), 24.87 (*C*H_3-*anti*-acetonide), 24.82 (*C*H_3-*anti*-acetonide), 23.19 (*C*H_2-39), 21.74 (β-*C*H_2), 21.72 (β-*C*H_2), 21.50 (β-*C*H_2), 21.29 (β-*C*H_2), 20.89 (β-*C*H_2), 19.95 (*C*H_3-*syn*-acetonide), 14.42 (*C*H_3-40 *or* CH_3-41), 14.28 (*C*H_3-40 *or* CH_3-41), 7.12 (OTES-*C*H_3), 7.10 (OTES-*C*H_3), 6.99 (OTES-*C*H_3), 5.29 (OTES-*C*H_2), 5.24 (OTES-*C*H_2), 5.04 (OTES-*C*H_2). See spectrum.

HRMS (m/z): (ESI) calculated for $C_{74}H_{155}O_{10}Si_5$ $[M+H]^+$ 1344.0461, found 1344.0446.

TLC: *R*f S25 = 0.31, ***R*f 24** = 0.19 (95:5 hexane:ethyl acetate, stained with *p*-anisaldehyde).

IR (ν_{max}/cm^{-1}, neat): 3457 (OH), 2951, 2936, 2875, 1738 (C=O), 1643 (C=C), 1457, 1376, 1217, 1091, 1008 (Si-O), 724 (Si-O).

$[\alpha]_D^{25}$: −8 (*c* = 1, $CHCl_3$).

(5*S*,6*S*,8*R*,12*R*,16*S*,20R)-21-((4*S*,6*S*)-6-((*S*)-7-((4*R*,6*S*)-6-allyl-2,2-dimethyl-1,3-dioxan-4-yl)-4-((triethylsilyl)oxy)heptyl)-2,2-dimethyl-1,3-dioxan-4-yl)-5-methyl-8,12,16,20-tetrakis((triethylsilyl)oxy)henicosan-6-yl (Z)-3-iodobut-2-enoate – 26

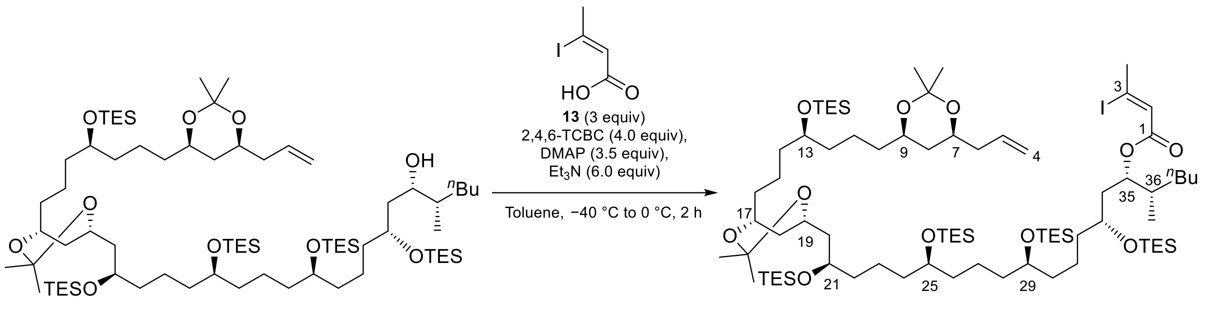

24 **26**, 72%

Prepared following General Procedure 6 using alcohol **24** (40 mg, 30 µmol) in toluene (0.60 mL), triethylamine (18 mg, 25 µL, 180 µmol), **13** (19 mg, 89 µmol) in toluene (0.25 mL), 4-dimethylamino pyridine (15 mg, 120 µmol) in toluene (0.25 mL), and 2,4,6-trichlorobenzoyl chloride (29 mg, 120 mmol). The reaction mixture was quenched by the addition of saturated aqueous $NaHCO_3$ (0.5 mL) and extracted with ethyl acetate (3 x 1 mL). The combined organic layers were dried over anhydrous Na_2SO_4, filtered and concentrated under reduced pressure. The crude mixture was purified by normal phase flash column chromatography (Biotage HC-10 g, pentane: ethyl acetate 0-15%) to afford the title compound (33 mg, 72%) as a colourless oil.

^{1}H NMR (600 MHz, $CDCl_3$) δ 6.27 (q, *J* = 1.5 Hz, 1H, *H*-2 alkene), 5.80 (ddt, *J* = 17.2, 10.2, 7.0 Hz, 1H, *H*-5 alkene), 5.10 – 5.06 (m, 1H, *H*-4’ *trans* alkene), 5.06 – 5.03 (m, 2H, *H*-4’ *cis* alkene & *H*-35), 3.93 – 3.88 (m, 1H, *H*-19), 3.88 – 3.83 (m, 1H, *H*-7), 3.81 – 3.71 (m, 3H, *H*-9, *H*-17 & *H*-21), 3.68 (dddd, *J* = 8.9, 5.5, 5.5, 3.3 Hz, 1H, *H*-33), 3.65 – 3.58 (m, 3H, *H*-13, *H*-25 & *H*-29), 2.72 (d, *J* = 1.4 Hz, 3H, C*H*3-42), 2.30 (ddddd, *J* = 12.5, 6.2, 6.2, 1.5, 1.5 Hz, 1H, *H*-6’), 2.14 (ddd, *J* = 14.1, 7.0, 7.0 Hz, 1H, *H*-6’’), 1.79 – 1.71 (m, 2H, *H*-20’ & *H*-36), 1.71 – 1.66 (m, 1H, C*H*2’), 1.59 – 1.43 (m, *C*H2-alkyls), 1.42 (s, 3H, C*H*3-*syn* acetonide), 1.42 – 1.39 (m, 11H, *C*H2-alkyls), 1.38 (s, 3H, C*H*3-*syn* acetonide), 1.38 – 1.32 (m, 10H, *C*H2-alkyls), 1.31 (s, 3H, C*H*3-*anti* acetonide), 1.31 (s, 3H, C*H*3-*anti* acetonide), 1.30 – 1.18 (m, 11H, *C*H2-alkyls), 1.15 – 1.01 (m, 3H, *H*-37’ & C*H*’), 0.98 – 0.92 (m, 45H, C*H*3-OTES), 0.90 – 0.85 (m, 6H, C*H*3-40 & C*H*3-41), 0.62 – 0.56 (m, 30H, C*H*2-OTES). See spectrum.

^{13}C NMR (151 MHz, $CDCl_3$) δ 163.99 (*C*OOR-1), 134.41 (*C*H-5 alkene), 126.29 (*C*H-2 alkene), 117.16 (*C*H_2-4 alkene), 112.37 (*C*-3 alkene), 100.19 (CO *anti*-acetonide), 98.55 (CO *syn*-acetonide), 75.84 (*C*H-35), 72.55 (*C*H-29), 72.53 (*C*H-25), 72.38 (*C*H-13), 69.49 (*C*H-33), 69.19 (*C*H-21), 69.01 (*C*H-9), 68.82 (*C*H-7), 66.70 (*C*H-17), 63.77 (*C*H-19), 43.80 (*C*H_2-20), 41.06 (*C*H_2-6), 39.35 (α-*C*H_2), 38.64 (α-*C*H_2), 37.95 (α-*C*H_2), 37.78 (α-*C*H_2), 37.68 (α-*C*H_2), 37.61 (α-*C*H_2), 37.32 (α-*C*H_2), 37.18 (α-*C*H_2), 36.75 (*C*H-36), 36.73 (α-*C*H_2), 36.64 (*C*H_3-42), 36.59 (α-*C*H_2), 36.28 (α-*C*H_2), 31.84 (*C*H_2-37), 30.40 (*C*H_3-*syn*-acetonide), 29.76 (*C*H_2-38), 24.87 (*C*H_3-*anti*-acetonide), 24.83 (*C*H_3-*anti*-acetonide), 23.05 (*C*H_2-39), 21.73 (β-*C*H_2), 21.49 (β-*C*H_2), 21.31 (β-*C*H_2), 20.94 (β-*C*H_2), 20.89 (β-*C*H_2), 19.95 (*C*H_3-*syn*-acetonide), 15.17 (*C*H_3-41), 14.21 (*C*H_3-40), 7.17 (OTES-*C*H_3), 7.14 (OTES-*C*H_3), 7.13 (OTES-*C*H_3), 7.11 (OTES-*C*H_3), 5.32 (OTES-*C*H_2), 5.30 (OTES-*C*H_2), 5.25 (OTES-*C*H_2). See spectrum.

HRMS (m/z): (nanospray) calculated for $C_{78}H_{157}IO_{11}Si_5Na$ $[M+Na]^+$ 1559.9515, found 1559.9528.

TLC: R_f = 0.26 (95:5 hexane:ethyl acetate, stained with *p*-anisaldehyde, product visible under UV lamp)

IR (ν_{max}/cm^{-1}, neat): 2952, 2937, 2913, 2876, 1728 (C=O), 1627 (C=C), 1459, 1378, 1239, 1175, 1121, 1016 (Si-C), 728 (Si-C).

$[\alpha]_D^{25}$: −6 (*c* = 1, $CHCl_3$).

(1*S*,5*S*,9*R*,13*S*,21*S*,23*R*,27*R*,31*S*,35*R*,37*S*,Z)-21-((*S*)-hexan-2-yl)-11,11,17,39,39-pentamethyl-5,23,27,31,35-pentakis((triethylsilyl)oxy)-10,12,20,38,40-pentaoxatricyclo[35.3.1.19,13]dotetracont-17-en-19-one – 28

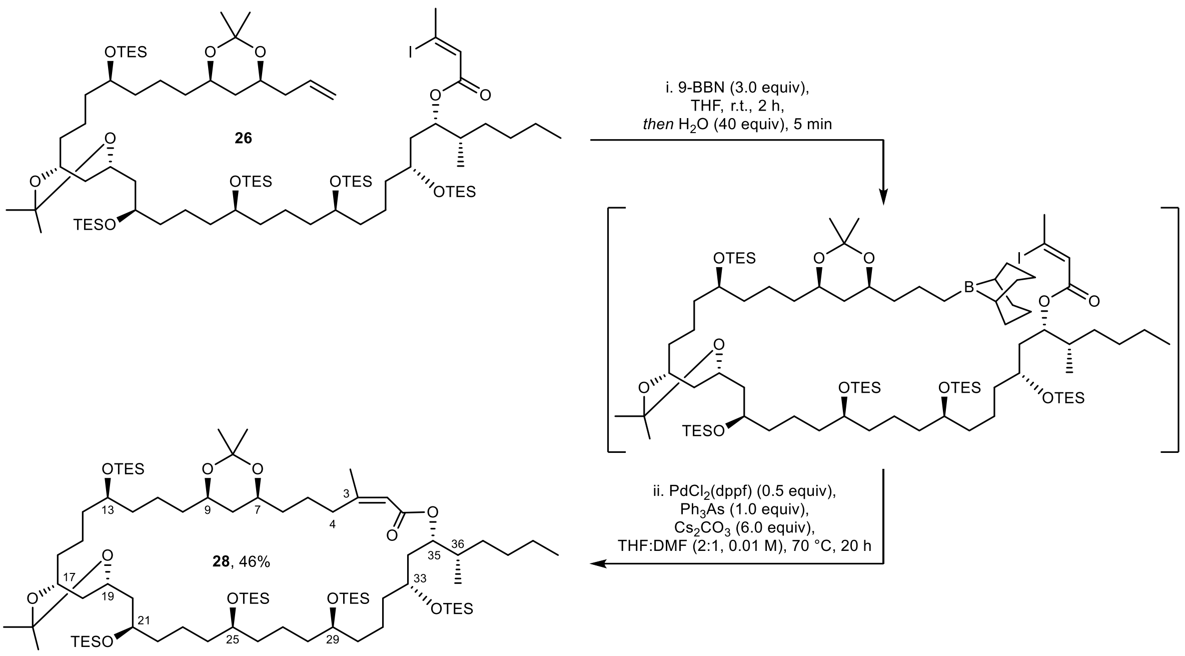

Prepared following General Procedure 7 using alkene **26** (10 mg, 6.5 μmol), 9-BBN (0.50 M THF solution, 19.5 μmol, 39 μL), H_2O (4.7 μL, 260 μmol), $PdCl_2$(dppf) (2.4 mg, 3.3 μmol), Ph_3As (2.0 mg, 6.5 μmol), and Cs_2CO_3 (13 mg, 39 μmol) in a dry and degassed mixture of THF:DMF 10:1 (1.0 mL) at 70 °C. The resulting reaction mixture was stirred for 20 h at 70 °C, then was quenched at room temperature by the addition of saturated sodium hydrogen carbonate solution (0.5 mL) and extracted with a 1:1 mixture of hexane:Et_2O (3 x 1 mL). The combined organic layers were dried over anhydrous Na_2SO_4, filtered over a short pad of silica and concentrated under reduced pressure. The crude mixture was purified by normal phase flash column chromatography (Biotage HC-10 g, pentane: ethyl acetate 0-15%) to afford the title compound (4.2 mg, 46%) as a colourless oil.

^{1}H NMR (600 MHz, $CDCl_3$) δ 5.64 (s, 1H, *H*-2 alkene), 4.97 (dt, *J* = 8.2, 3.7 Hz, 1H, *H*-35), 3.93 – 3.86 (m, 1H, *H*-19), 3.84 – 3.72 (m, 4H, *H*-7, *H*-9, *H*-17 & *H*-21), 3.71 – 3.66 (m, 1H, *H*-33), 3.63 (h, *J* = 5.7 Hz, 3H, *H*-13, *H*-25 & *H*-29), 2.87 (ddd, *J* = 11.0, 10.0, 5.2 Hz, 1H, *H*-4'), 2.38 (ddd, *J* = 11.5, 8.8, 4.9 Hz, 1H, *H*-4''), 1.88 (s, 3H, CH_3-42), 1.74 – 1.42 (m, 35H, *H*-36, CH_2-alkyls), 1.42 (s, 3H, CH_3-*syn* acetonide), 1.41 – 1.38 (m, 10H, CH_2-alkyls), 1.38 (s, 3H, CH_3-*syn* acetonide), 1.37 – 1.32 (m, 7H, CH_2-alkyls), 1.32 (s, 3H, CH_3-*anti* acetonide), 1.31 (s, 3H, CH_3-*anti* acetonide), 1.30 – 1.14 (m, 34H, CH_2-alkyls), 1.14 – 1.01

(m, 2H, *H*-37' & C*H*'), 0.99 – 0.91 (m, 45H, CH_3-OTES), 0.89 – 0.87 (m, 6H, CH_3-40 & CH_3-41), 0.64 – 0.55 (m, 30H, m, 30H, CH_2-OTES). See spectrum.

^{13}C NMR (151 MHz, CDCl$_3$) δ 166.00 (*C*OOR-1), 159.81 (*C*-3 alkene), 116.89 (*C*H-2 alkene), 100.15 (CO *anti*-acetonide), 98.47 (CO *syn*-acetonide), 73.92 (*C*H-35), 72.56 (*C*H-25), 72.44 (*C*H-13), 72.15 (*C*H-29), 69.75 (*C*H-33), 69.15 (*C*H-7, *C*H-9 *or* *C*H-21), 69.08 (2X *C*H-7, *C*H-9 *or* *C*H-21), 66.55 (*C*H-17), 63.87 (*C*H-19), 43.60 (*C*H_2-20), 39.14 (α-*C*H_2), 38.74 (α-*C*H_2), 38.44 (α-*C*H_2), 37.76 (α-*C*H_2), 37.65 (α-*C*H_2), 37.63 (α-*C*H_2), 37.14 (α-*C*H_2), 36.94 (α-*C*H_2), 36.87 (*C*H-36), 36.82 (α-*C*H_2), 36.69 (α-*C*H_2), 36.51 (α-*C*H_2), 36.19 (α-*C*H_2), 33.16 (*C*H_2-4), 32.25 (*C*H_2-37), 30.48 (*C*H_3-*syn*-acetonide), 29.75 (*C*H_2-38), 25.29 (*C*H_3-42), 25.00 (*C*H_3-*anti*-acetonide), 24.93 (*C*H_3-*anti*-acetonide), 23.97 (*C*H-5), 23.06 (*C*H_2-39), 21.41 (β-*C*H_2), 21.30 (β-*C*H_2), 21.02 (β-*C*H_2), 20.95 (β-*C*H_2), 20.86 (β-*C*H_2), 19.98 (*C*H_3-*syn*-acetonide), 14.93 (*C*H_3-41), 14.23 (*C*H_3-40), 7.22 (OTES-*C*H_3), 7.14 (OTES-*C*H_3), 7.13 (OTES-*C*H_3), 7.10 (OTES-*C*H_3), 5.32 (OTES-*C*H_2), 5.29 (OTES-*C*H_2), 5.20 (OTES-*C*H_2). See spectrum.

HRMS (m/z): (ESI) calculated for $C_{78}H_{159}O_{11}Si_5$ $[M+H]^+$ 1412.0723, found 1412.0696.

TLC: ***R*f** = 0.27 (95:5 hexane:ethyl acetate, stained with *p*-anisaldehyde)

IR (ν_{max}/cm^{-1}, neat): 2953, 2924, 2874, 2853, 1725 (C=O), 1648 (C=C), 1460, 1377, 1230, 1099, 1017 (Si-O), 729 (Si-C).

$[\alpha]_D^{25}$: −20 (*c* = 1, CHCl$_3$).

(8*S*,10*R*,14*S*,18*R*,20*R*,22*R*,26*S*,30*R*,34*R*,36*S*,Z)-36-((*S*)-hexan-2-yl)-8,10,14,18,20,22,26,30,34-nonahydroxy-4-methyloxacyclohexatriacont-3-en-2-one – 2

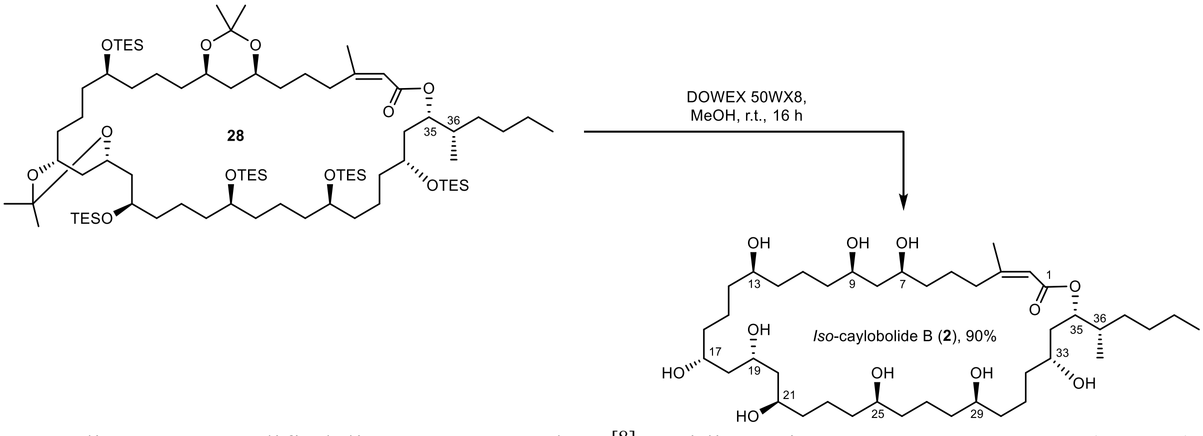

According to a modified literature procedure.[8] Acidic resin DOWEX-50W X8 (14 mg), previously washed with excess of MeOH, was added to a solution of protected *iso*-

caylobolide B **28** (3.5 mg, 7.5 µmol, 1.0 equiv) in MeOH (0.003 M, 0.8 mL). The reaction mixture was stirred at 16 h at r.t. then was filtered through a PTFE syringe filter. The resin was then washed with MeOH (1 mL). The combined organic layers were concentrated under a stream of nitrogen. The resulting residue was washed with HPLC-grade pentane (3 x 1 mL) and then dried under high vacuum to afford analytically pure *iso*-caylobolide B (**2**) (1.7 mg, 90%) as a white powder.

^{1}H NMR (600 MHz, Pyr) δ 5.93 (s, 1H, *H*-2 alkene), 5.75 – 5.68 (m, 1H, *H*-35), 4.80 – 4.72 (m, 1H, *H*-19), 4.49 – 4.42 (m, 1H, *H*-17), 4.33 – 4.26 (m, 1H, *H*-21), 4.26 – 4.21 (m, 1H, *H*-9), 4.21 – 4.14 (m, 1H, *H*-7), 4.06 – 3.99 (m, 1H, *H*-33), 3.99 – 3.88 (m, 3H, *H*-13, *H*-25, *H*-29), 3.15 – 3.07 (m, 1H, *H*-4'), 2.62 (dt, *J* = 12.9, 7.5 Hz, 1H, *H*-4''), 2.20 – 1.67 (m, 48H, CH_2-alkyls & CH_3-42), 1.60 – 1.51 (m, 1H, CH_2-37'), 1.40 – 1.17 (m, 5H, CH_2-37'', CH_2-38 & CH_2-39), 1.04 (d, *J* = 6.7 Hz, 3H, CH_3-41), 0.84 (t, *J* = 7.0 Hz, 3H, CH_3-40). *See spectrum*.

^{13}C NMR (151 MHz, Pyr) δ 167.25 (*C*OOR-1), 160.78 (*C*-3 alkene), 117.56 (*C*H-2 alkene), 74.46 (*C*H-35), 72.10 (*C*H-9), 71.87 (*C*H-7), 71.52 (*C*H-29), 71.22 (*C*H-25), 71.20 (*C*H-13), 70.96 (*C*H-21), 69.35 (*C*H-19), 68.77 (*C*H-17), 68.17 (*C*H-33), 45.38 (CH_2-20), 45.08 (CH_2-8), 44.98 (CH_2-18), 41.07 (CH_2-34), 39.53 (CH_2-32), 39.40 (CH_2-10), 39.11 (CH_2-22), 39.04 (CH_2-6), 39.02 (CH_2-30), 38.89 (CH_2-28), 38.86 (CH_2-16), 38.78 (CH_2-26), 38.73 (CH_2-12), 38.62 (CH_2-24), 38.56 (CH_2-14), 37.94 (*C*H-36), 33.91 (CH_2-4), 33.45 (CH_2-37), 30.11 (CH_2-38), 25.27 (CH_3-42), 25.16 (CH_2-5), 23.52 (CH_2-39), 23.26 (CH_2-27 & CH_2-31), 22.91 (CH_2-11), 22.70 (CH_2-15), 22.42 (CH_2-23), 15.33 (CH_3-41), 14.59 (CH_3-40). *See spectrum*.

HRMS (m/z): (ESI) calculated for $C_{42}H_{81}O_{11}$ $[M+H]^+$ 761.5773, found 761.5774.

TLC: R_f = 0.44 (80:20 dichloromethane:methanol, stained with *p*-anisaldehyde)

IR (ν_{max}/cm^{-1}, neat): 3305 (OH), 2924, 2858, 1738 (C=O), 1649 (C=C), 1455, 1366, 1217, 1121.

$[\alpha]_D^{25}$: −24 (*c* = 0.34, MeOH).

Comparison table of isolated and synthetic *iso*-caylobolide B (2)

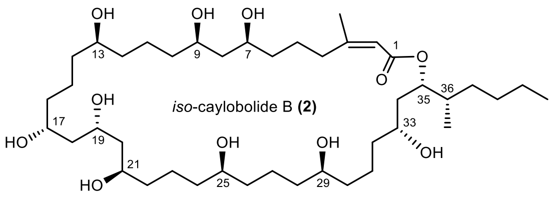

Table S8: ^{13}C NMR comparison of *iso*-caylobolide B (**2**) to synthetic *iso*-caylobolide B. Spectra recorded in pyridine-d_5, referenced to 123.87 ppm.

Position, type	Isolated δC (151 MHz, pyr-d_5)	Synthetic δC (151 MHz, pyr-d_5)	ΔδC (Isolation – synthetic)
**1 C**	167.23	167.25	-0.02
**2 CH**	117.53	117.56	-0.03
**3 C**	160.78	160.78	0
**4 CH_2**	33.89	33.91	-0.02
**5 CH_2**	25.14	25.16	-0.02
**6 CH_2**	38.99	39.04	-0.05
**7 CHOH**	71.81	71.87	-0.06
**8 CH_2**	45.05	45.08	-0.03
**9 CHOH**	72.04	72.1	-0.06
**10 CH_2**	39.34	39.4	-0.06
**11 CH_2**	22.87	22.91	-0.04
**12 CH_2**	38.67	38.73	-0.06
**13 CHOH**	71.17	71.2	-0.03
**14 CH_2**	38.5	38.56	-0.06
**15 CH_2**	22.66	22.7	-0.04
**16 CH_2**	38.82	38.86	-0.04
**17 CHOH**	68.73	68.77	-0.04
**18 CH_2**	44.96	44.98	-0.02
**19 CHOH**	69.26	69.35	-0.09
**20 CH_2**	45.37	45.38	-0.01
**21 CHOH**	70.89	70.96	-0.07
**22 CH_2**	39.05	39.11	-0.06
**23 CH_2**	22.39	22.42	-0.03
**24 CH_2**	38.55	38.62	-0.07
**25 CHOH**	71.2	71.22	-0.02
**26 CH_2**	38.73	38.78	-0.05
**27 CH_2**	23.21	23.26	-0.05
**28 CH_2**	38.83	38.89	-0.06
**29 CHOH**	71.5	71.52	-0.02
**30 CH_2**	38.96	39.02	-0.06
**31 CH_2**	23.21	23.26	-0.05
**32 CH_2**	39.48	39.53	-0.05
**33 CHOH**	68.16	68.17	-0.01
**34 CH_2**	41.03	41.07	-0.04
**35 CHOR**	74.44	74.46	-0.02
**36 CH**	37.91	37.94	-0.03
**37 CH_2**	33.42	33.45	-0.03
**38 CH_2**	30.08	30.11	-0.03
**39 CH_2**	23.49	23.52	-0.03
**40 CH_3**	14.57	14.59	-0.02
**41 CH_3**	15.3	15.33	-0.03
**42 CH_3**	25.25	25.27	-0.02

Caylobolide B (1) final steps

(5*R*,6*S*,8*R*,12*R*,16*S*,20R)-21-((4*S*,6*S*)-6-((*S*)-7-((4*R*,6*S*)-6-allyl-2,2-dimethyl-1,3-dioxan-4-yl)-4-((triethylsilyl)oxy)heptyl)-2,2-dimethyl-1,3-dioxan-4-yl)-5-methyl-8,12,16,20-tetrakis((triethylsilyl)oxy)henicosan-6-ol – 25

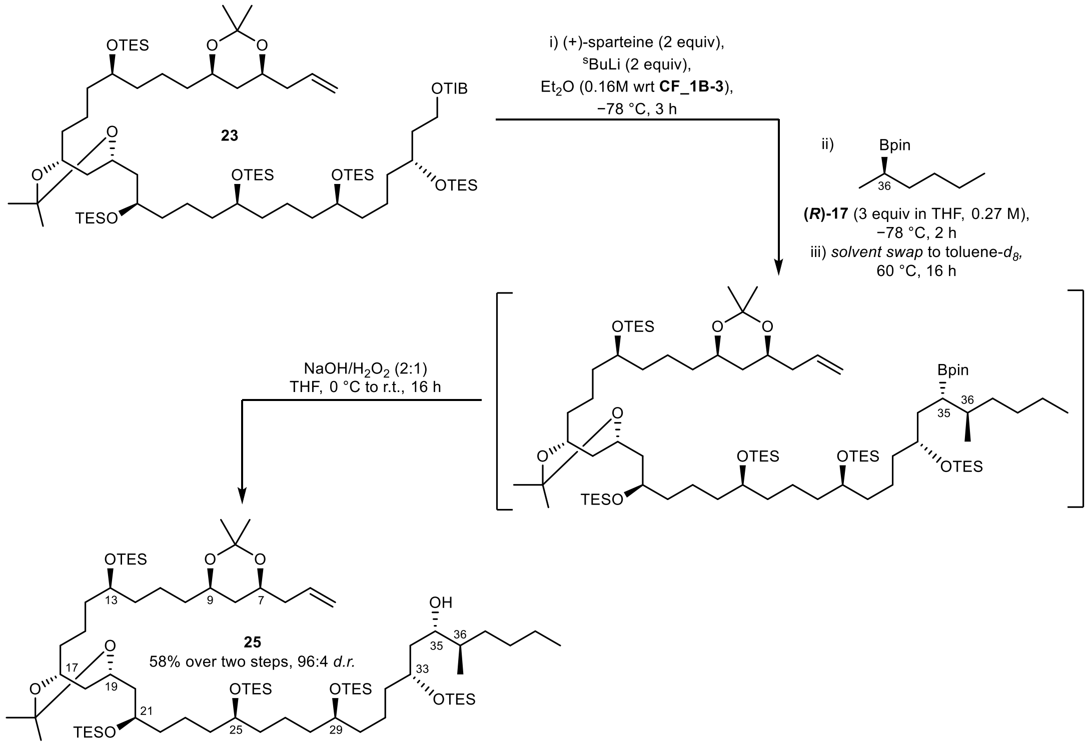

Prepared following the same procedure as described in the preparation of **24** using **23** (95 mg, 64 μmol), (+)-sparteine (30 mg, 29 μL, 130 μmol) in Et_2O (400 μL, 0.16 M), *s*-BuLi (1.31 M in hexanes, 97 μL,130 μmol), and **(*R*)-17** (41 mg, 190 μmol) in THF (710 μL, 0.27 M). A 2:1 solution of aqueous sodium hydroxide (3 M) and 30 % aqueous hydrogen (0.5 mL total volume) was added directly to the reaction mixture at 0 °C, and the reaction mixture was then stirred at r.t. for 16 h. The reaction mixture was quenched by the addition of saturated sodium thiosulfate (0.5 mL) and was extracted with ethyl acetate (5 x 1 mL). The organic layers were combined, and washed with brine (1 mL), then dried over anhydrous Na_2SO_4 and concentrated under reduced pressure. The crude residue was purified by normal phase flash column chromatography (Biotage HC-10 g, pentane: ethyl acetate 0-15%) to afford the title compound (50 mg, 58% over two steps) as a colourless oil.

^{1}H NMR (500 MHz, $CDCl_3$) δ 5.86 – 5.74 (m, 1H, *H*-5 alkene), 5.12 – 4.99 (m, 2H, C*H*$_2$-4 alkene), 4.05 – 3.96 (m, 1H, *H*-33), 3.95 – 3.82 (m, 2H, *H*-19 & *C*H-7), 3.82 – 3.69 (m, 4H, *C*H-9, *C*H-17, *C*H-21 & *H*-35), 3.68 – 3.56 (m, 3H, *H*-13, *H*-25 & *H*-29), 3.46 (s, 1H, O-*H*),

2.30 (ddddd, *J* = 14.0, 6.2, 6.2, 1.4, 1.4 Hz, 1H, *H*-6’), 2.18 – 2.10 (m, 1H, *H*-6’’), 1.73 (ddd, *J* = 13.5, 7.7, 5.7 Hz, 1H, *H*-20’), 1.63 – 1.42 (m, 18H, CH_2-alkyls), 1.42 (s, 3H, CH_3-*syn* acetonide), 1.42 – 1.38 (m, 10H, CH_2-alkyls), 1.38 (s, 3H, CH_3-*syn* acetonide), 1.38 – 1.32 (m, 10H, CH_2-alkyls), 1.31 (s, 3H, CH_3-*anti* acetonide), 1.30 (s, 3H, CH_3-*anti* acetonide), 1.30 – 1.15 (m, 12H, CH_2-alkyls), 1.15 – 1.04 (m, 3H, CH_2 & CH_2-37’), 1.00 – 0.92 (m, 45H, CH_3-OTES), 0.91 – 0.84 (m, 6H, CH_3-40 & CH_3-41), 0.65 – 0.54 (m, 30H, CH_2-OTES). See spectrum.

^{13}C NMR (126 MHz, CDCl$_3$) δ 134.39 (*C*H-5 alkene), 117.15 (*C*H$_2$-4 alkene), 100.19 (CO *anti*-acetonide), 98.54 (CO *syn*-acetonide), 72.52 (*C*H-25), 72.40 (*C*H-29), 72.37 (*C*H-13), 72.15 (*C*H-35), 72.03 (*C*H-33), 69.17 (*C*H-21), 69.00 (*C*H-9), 68.81 (*C*H-7), 66.69 (*C*H-17), 63.77 (*C*H-19), 43.78 (*C*H$_2$-20), 41.05 (*C*H$_2$-6), 39.34 (α-*C*H$_2$), 39.07 (*C*H-36), 37.77 (α-*C*H$_2$), 37.71 (α-*C*H$_2$), 37.67 (α-*C*H$_2$), 37.56 (α-*C*H$_2$), 37.39 (α-*C*H$_2$), 37.31 (α-*C*H$_2$), 37.17 (α-*C*H$_2$), 37.00 (α-*C*H$_2$), 36.72 (α-*C*H$_2$), 36.59 (α-*C*H$_2$), 36.55 (α-*C*H$_2$), 36.27 (α-*C*H$_2$), 32.16 (*C*H$_2$-37), 30.39 (*C*H$_3$-*syn*-acetonide), 29.72 (*C*H$_2$-38), 24.86 (*C*H$_3$-*anti*-acetonide), 24.82 (*C*H$_3$-*anti*-acetonide), 23.19 (*C*H$_2$-39), 21.82 (β-*C*H$_2$), 21.72 (β-*C*H$_2$), 21.48 (β-*C*H$_2$), 21.29 (β-*C*H$_2$), 20.89 (β-*C*H$_2$), 19.94 (*C*H$_3$-*syn*-acetonide), 15.00 (*C*H$_3$-41), 14.28 (*C*H$_3$-40), 7.11 (OTES-*C*H$_3$), 7.10 (OTES-*C*H$_3$), 7.09 (OTES-*C*H$_3$), 6.98 (OTES-*C*H$_3$), 5.29 (OTES-*C*H$_2$), 5.27 (OTES-*C*H$_2$), 5.24 (OTES-*C*H$_2$), 5.03 (OTES-*C*H$_2$). See spectrum.

HRMS (m/z): (ESI) calculated for $C_{74}H_{155}O_{10}Si_5$ $[M+H]^+$ 1344.0461, found 1344.0446.

TLC: *R*$_f$ 25 = 0.19 (95:5 hexane:ethyl acetate, stained with *p*-anisaldehyde).

IR (ν_{max}/cm^{-1}, neat): 3457 (OH), 2951, 2936, 2875, 1738 (C=O), 1643 (C=C), 1457, 1376, 1217, 1091, 1008 (Si-O), 724 (Si-O).

$[\alpha]_D^{25}$: −8 (*c* = 1, CHCl$_3$).

(5*R*,6*S*,8*R*,12*R*,16*S*,20R)-21-((4*S*,6*S*)-6-((*S*)-7-((4*R*,6*S*)-6-allyl-2,2-dimethyl-1,3-dioxan-4-yl)-4-((triethylsilyl)oxy)heptyl)-2,2-dimethyl-1,3-dioxan-4-yl)-5-methyl-8,12,16,20-tetrakis((triethylsilyl)oxy)henicosan-6-yl (Z)-3-iodobut-2-enoate – 27

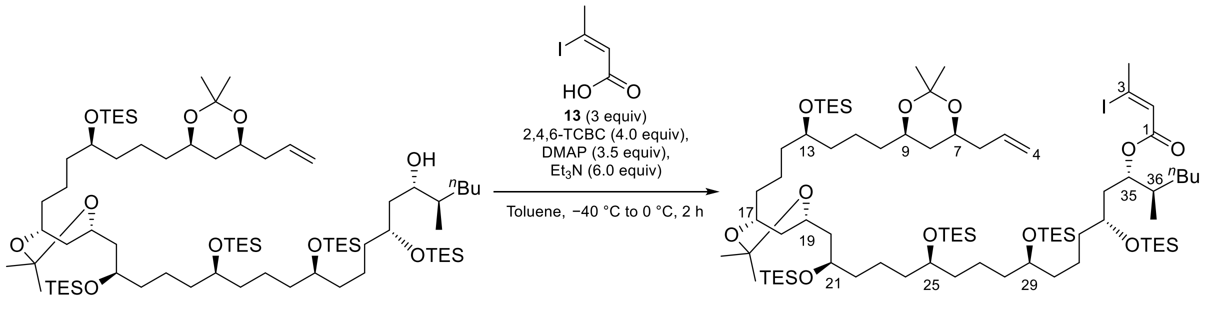

Prepared following General Procedure 6 using alcohol **25** (20 mg, 15 µmol) in toluene (0.23 mL), triethylamine (9.0 mg, 12 µL, 89 µmol), **13** (9.5 mg, 45 µmol) in toluene (0.1 mL), 4-dimethylamino pyridine (7.3 mg, 59 µmol) in toluene (0.1 mL), and 2,4,6-trichlorobenzoyl chloride (15 mg, 59 mmol). The reaction mixture was quenched by the addition of saturated aqueous $NaHCO_3$ (0.5 mL) and extracted with ethyl acetate (3 x 1 mL). The combined organic layers were dried over anhydrous Na_2SO_4, filtered and concentrated under reduced pressure. The crude mixture was purified by normal phase flash column chromatography (Biotage HC-10 g, hexane: ethyl acetate 0-15%). Due to coelution with impurities, the crude material was repurified by normal phase preparative HPLC (hexane:ethyl acetate 0-20%) to afford the title compound (12 mg, 21%) as a colourless oil.

^{1}H NMR (500 MHz, CDCl3) δ 6.27 (d, *J* = 1.5 Hz, 1H, *H*-2 alkene), 5.85 – 5.75 (m, 1H, *H*-5 alkene), 5.11 – 5.03 (m, 3H, C*H*$_2$-4 alkene & *H*-35), 3.94 – 3.83 (m, 2H, *H*-7 & *H*-19), 3.81 – 3.73 (m, 3H, *H*-9, *H*-17 & *H*-21), 3.73 – 3.67 (m, 1H, *H*-33), 3.67 – 3.59 (m, 3H, *H*-13, *H*-25 & *H*-29), 2.73 (d, *J* = 1.4 Hz, 3H, C*H*$_3$-42), 2.31 (ddddd, *J* = 14.1, 6.2, 6.2, 1.4, 1.4 Hz, 1H, *H*-6'), 2.18 – 2.11 (m, 1H, *H*-6''), 1.92 – 1.83 (m, 1H, *H*-36), 1.78 – 1.43 (m, 26H, *C*H$_2$-alkyls), 1.43 (s, 3H, C*H*$_3$-*syn* acetonide), 1.40 (s, 12H, *C*H$_2$-alkyls), 1.39 (s, 3H, C*H*$_3$-*syn* acetonide), 1.38 – 1.32 (m, 14H, *C*H$_2$-alkyls), 1.32 (s, 3H, C*H*$_3$-*anti* acetonide), 1.31 (s, 3H, C*H*$_3$-*anti* acetonide), 1.30 – 1.17 (m, 22H, *C*H$_2$-alkyls), 1.16 – 1.05 (m, 3H, *H*-37' & C*H*'), 0.99 – 0.92 (m, 45H, C*H*$_3$-OTES), 0.91 – 0.82 (m, 6H, C*H*$_3$-40 & C*H*$_3$-41), 0.63 – 0.55 (m, 30H, C*H*$_2$-OTES). See spectrum.

^{13}C NMR (126 MHz, CDCl$_3$) δ 163.92 (*C*OOR-1), 134.41 (*C*H-5 alkene), 126.29 (*C*H-2 alkene), 117.17 (*C*H$_2$-4 alkene), 112.40 (*C*-3 alkene), 100.20 (CO *anti*-acetonide), 98.56 (CO

syn-acetonide), 75.76 (*C*H-35), 72.56 (*C*H-29), 72.53 (*C*H-25), 72.38 (*C*H-13), 69.29 (*C*H-33), 69.19 (*C*H-21), 69.02 (*C*H-9), 68.82 (*C*H-7), 66.70 (*C*H-17), 63.78 (*C*H-19), 43.80 (CH_2-20), 41.06 (CH_2-6), 39.35 (α-CH_2), 38.75 (α-CH_2), 37.81 (α-CH_2), 37.78 (α-CH_2), 37.68 (α-CH_2), 37.60 (α-CH_2), 37.32 (α-CH_2), 37.19 (α-CH_2), 36.73 (α-CH_2), 36.67 (CH_3-42), 36.59 (α-CH_2), 36.28 (α-CH_2), 36.23 (α-CH_2), 36.14 (*C*H-36), 32.72 (CH_2-37), 30.40 (CH_3-*syn*-acetonide), 29.66 (CH_2-38), 24.87 (CH_3-*anti*-acetonide), 24.83 (CH_3-*anti*-acetonide), 23.10 (CH_2-39), 21.74 (β-CH_2), 21.48 (β-CH_2), 21.31 (β-CH_2), 20.94 (β-CH_2), 20.90 (β-CH_2), 19.96 (CH_3-*syn*-acetonide), 14.42 (CH_3-41), 14.19(CH_3-40), 7.18 (OTES-CH_3), 7.16 (OTES-CH_3), 7.15 (OTES-CH_3), 7.13 (OTES-CH_3), 7.11 (OTES-CH_3), 5.31 (OTES-CH_2), 5.30 (OTES-CH_2), 5.25 (OTES-CH_2), 5.21 (OTES-CH_2). See spectrum.

HRMS (m/z): (nanospray) calculated for $C_{78}H_{157}IO_{11}Si_5Na$ $[M+Na]^+$ 1559.9515, found 1559.9528.

TLC: $\boldsymbol{R_f}$ = 0.26 (95:5 hexane:ethyl acetate, stained with *p*-anisaldehyde, product visible under UV lamp)

IR (ν_{max}/cm^{-1}, neat): 2952, 2937, 2913, 2876, 1728 (C=O), 1627 (C=C), 1459, 1378, 1239, 1175, 1121, 1016 (Si-C), 728 (Si-C).

$[\alpha]_D^{25}$: −16 (c = 1, $CHCl_3$).

(1*S*,5*S*,9*R*,13*S*,21*S*,23*R*,27*R*,31*S*,35*R*,37*S*,Z)-21-((R)-hexan-2-yl)-11,11,17,39,39-pentamethyl-5,23,27,31,35-pentakis((triethylsilyl)oxy)-10,12,20,38,40-pentaoxatricyclo[35.3.1.19,13]dotetracont-17-en-19-one – 29

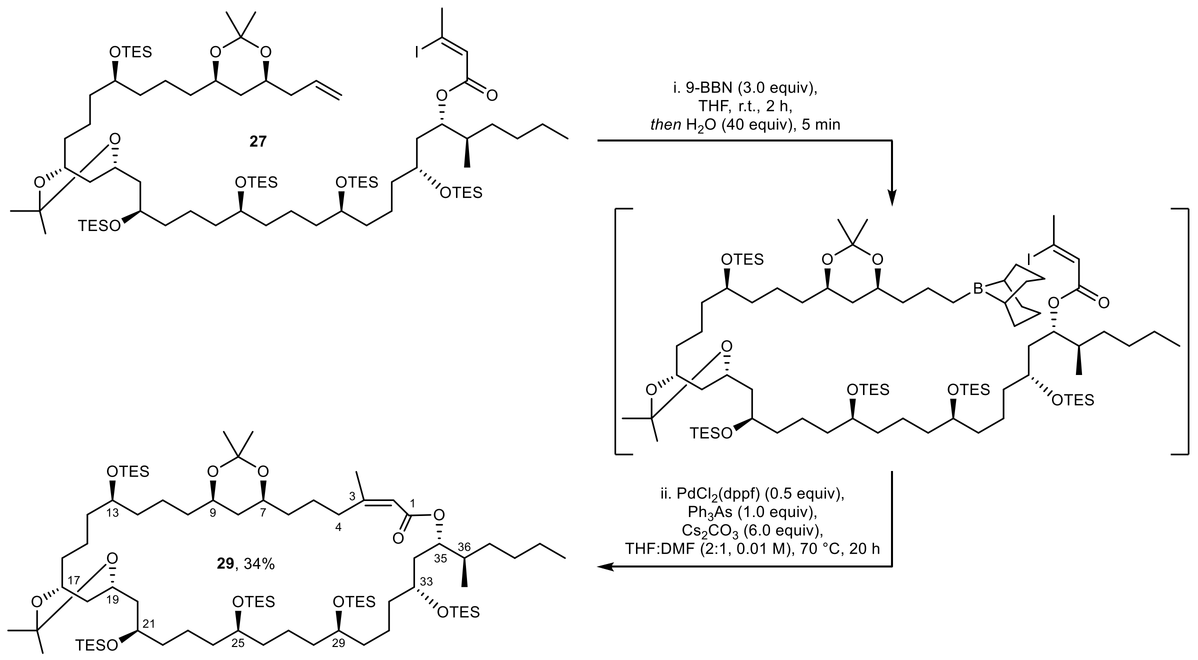

Prepared following General Procedure 7 using **27** (8.0 mg, 5.2 μmol), 9-BBN (0.50 M THF solution, 16 μmol, 31 μL), H_2O (3.7 μL, 210 μmol), $PdCl_2$(dppf) (1.9 mg, 2.6 μmol), Ph_3As (1.6 mg, 5.2 μmol), and Cs_2CO_3 (10 mg, 31 μmol) in a dry and degassed mixture of THF:DMF 10:1 (0.52 mL) at 70 °C. The resulting reaction mixture was stirred for 20 h at 70 °C, then was quenched at room temperature by the addition of saturated sodium hydrogen carbonate solution (0.5 mL) and extracted with a 1:1 mixture of hexane:Et_2O (3 x 1 mL). The combined organic layers were dried over anhydrous Na_2SO_4, filtered over a short pad of silica and concentrated under reduced pressure. The crude mixture was purified by normal phase flash column chromatography (Biotage HC-10 g, pentane: ethyl acetate 0-15%) and then repurified by normal phase preparative HPLC (hexane: ethyl acetate 0-20%) to afford the title compound (2.5 mg, 34%) as a colourless oil.

^{1}H NMR (500 MHz, $CDCl_3$) δ 5.64 (d, *J* = 1.6 Hz, 1H, *H*-2 alkene), 4.97 (dt, *J* = 9.4, 3.3 Hz, 1H, *H*-35), 3.94 – 3.84 (m, 1H, *H*-19), 3.85 – 3.72 (m, 4H, *H*-7, *H*-9, *H*-17 & *H*-21), 3.72 – 3.66 (m, 1H, *H*-33), 3.67 – 3.58 (m, 3H, *H*-13, *H*-25 & *H*-29), 2.93 – 2.84 (m, 1H, *H*-4'), 2.43 – 2.34 (m, 1H, *H*-4''), 1.88 (d, *J* = 1.3 Hz, 3H, CH_3-42), 1.82 – 1.42 (m, 37H, CH_2-alkyls & *H*-36), 1.42 (s, 3H, CH_3-*syn* acetonide), 1.40 (s, 11H, CH_2-alkyls), 1.37 (s, 3H, CH_3-*syn* acetonide), 1.37 – 1.32 (m, 10H, CH_2-alkyls), 1.32 (s, 3H, CH_3-*syn* acetonide), 1.31 (s,

3H, CH_3-*syn* acetonide), 1.30 – 1.17 (m, 25H, CH_2-alkyls), 1.14 – 1.03 (m, 2H, *H*-37' & C*H*'), 1.00 – 0.92 (m, 45H, CH_3-OTES), 0.92 – 0.83 (m, 6H, CH_3-40 & CH_3-41), 0.64 – 0.54 (m, 30H, CH_2-OTES). See spectrum.

^{13}C NMR (126 MHz, $CDCl_3$) δ 166.00 (*C*OOR-1), 159.68 (*C*-3 alkene), 117.04 (*C*H-2 alkene), 100.15 (CO *anti*-acetonide), 98.46 (CO *syn*-acetonide), 74.21 (*C*H-35), 72.54 (*C*H-25), 72.46 (*C*H-13), 72.16 (*C*H-29), 69.60 (*C*H-33), 69.14 (*C*H-7, *C*H-9 *or* *C*H-21), 69.05 (2X *C*H-7, *C*H-9 *or* *C*H-21), 66.56 (*C*H-17), 63.87 (*C*H-19), 43.60 (CH_2-20), 39.14 (α-CH_2), 38.56 (α-CH_2), 37.78 (α-CH_2), 37.75 (α-CH_2), 37.65 (α-CH_2), 37.62 (α-CH_2), 37.10 (α-CH_2), 36.96 (α-CH_2), 36.84 (α-CH_2), 36.68 (α-CH_2), 36.47 (*C*H-36) 36.44 (α-CH_2), 36.19 (α-CH_2), 33.07 (CH_2-4), 32.41 (CH_2-37), 30.47 (CH_3-*syn*-acetonide), 29.85 (CH_2-38), 25.25 (CH_3-42), 24.99 (CH_3-*anti*-acetonide), 24.91 (CH_3-*anti*-acetonide), 23.89 (*C*H-5), 23.12 (CH_2-39), 21.41 (β-CH_2), 21.29 (β-CH_2), 20.99 (β-CH_2), 20.83 (β-CH_2), 19.97 (CH_3-*syn*-acetonide), 14.78 (CH_3-41), 14.23 (CH_3-40), 7.23 (OTES-CH_3), 7.14 (OTES-CH_3), 7.13 (OTES-CH_3), 7.10 (OTES-CH_3), 5.32 (OTES-CH_2), 5.28 (OTES-CH_2), 5.20 (OTES-CH_2). See spectrum.
HRMS (m/z): (ESI) calculated for $C_{78}H_{159}O_{11}Si_5$ $[M+H]^+$ 1412.0723, found 1412.0696.
TLC: R_f = 0.27 (95:5 hexane:ethyl acetate, stained with *p*-anisaldehyde)

IR (ν_{max}/cm^{-1}, neat): 2953, 2924, 2874, 2853, 1725 (C=O), 1648 (C=C), 1460, 1377, 1230, 1099, 1017 (Si-O), 729 (Si-C).

$[\alpha]_D^{25}$: −20 (*c* = 1, $CHCl_3$).

(8*S*,10*R*,14*S*,18*R*,20*R*,22*R*,26*S*,30*R*,34*R*,36*S*,Z)-36-((R)-hexan-2-yl)-8,10,14,18,20,22,26,30,34-nonahydroxy-4-methyloxacyclohexatriacont-3-en-2-one – 1

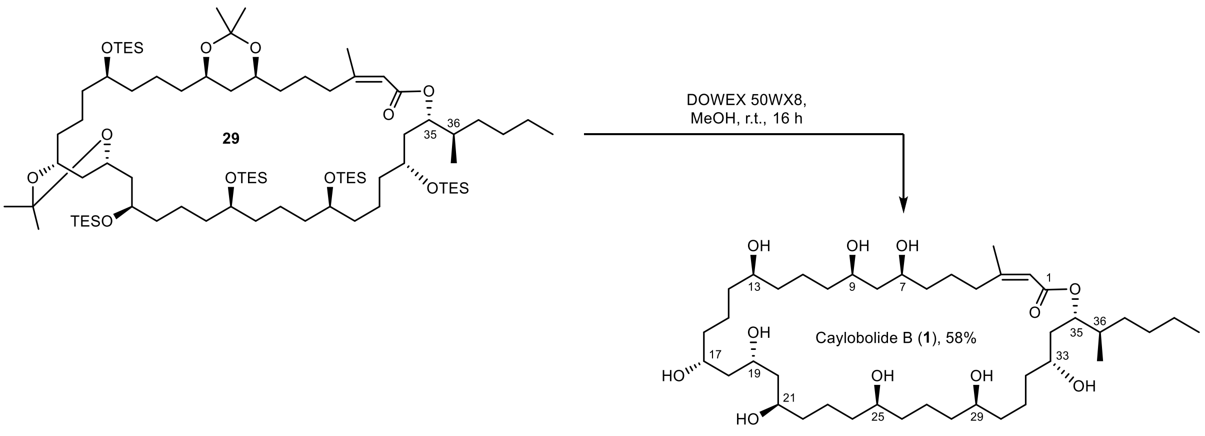

Prepared following the same procedure as described for the deprotection of **28** using **29** (2.0 mg, 1.4 μmol, 1 equiv) acidic resin DOWEX-50W X8 (11 mg), in MeOH (0.003 M, 0.47

mL). The crude mixture was purified by normal phase flash column chromatography (dichloromethane: MeOH 4-20%) to afford **1** as a white powder. Upon NMR and LCMS analysis it was identified that there were several unknown impurities present. The sample was repurified by reverse phase semi-preparative HPLC (water: acetonitrile, 35-65%, 4 ml/min, 10-minute gradient) which, upon evaporation and lyophilisation, afforded analytically pure synthetic caylobolide B (**1**) (570 µg, 58%) as a white film.

^{1}H NMR (700 MHz, Pyr) δ 6.23 (br.d, *J* = 3.9 Hz, 1H, O-*H*), 6.20 (br.d, *J* = 4.0 Hz, 1H, O-*H*), 6.14 (br.d, *J* = 3.7 Hz, 1H, O-*H*), 6.12 (br.d, *J* = 4.3 Hz, 1H, O-*H*), 6.00 (br.d, *J* = 4.8 Hz, 1H, O-*H*), 5.90 (s, 1H, *H*-2 alkene), 5.84 (br.d, *J* = 5.4 Hz, 1H, O-*H*), 5.80 – 5.70 (m, 3H, O-*H*), 5.67 (ddd, *J* = 10.3, 4.5, 2.3 Hz, 1H, *H*-35), 4.75 – 4.70 (m, 1H, *H*-19), 4.46 – 4.39 (m, 1H, *H*-17), 4.29 – 4.23 (m, 1H, *H*-21), 4.23 – 4.18 (m, 1H, *H*-9), 4.18 – 4.13 (m, 1H, *H*-7), 4.03 – 3.97 (m, 1H, *H*-33), 3.96 – 3.87 (m, 3H, *H*-13, *H*-25, *H*-29), 3.09 (ddd, *J* = 11.9, 9.7, 5.3 Hz, 1H, *H*-4'), 2.57 (ddd, *J* = 14.0, 8.8, 5.5 Hz, 1H, *H*-4''), 2.15 – 1.99 (m, 7H, CH_2-11', CH_2-18, CH_2-20, CH_2-27' & CH_2-31'), 1.98 – 1.66 (m, 39H, *C*H_2-alkyls, *C*H_3-42 & *C*H-36), 1.53 – 1.46 (m, 1H, CH_2-37'), 1.38 – 1.31 (m, 1H CH_2-38'), 1.26 – 1.16 (m, 4H, CH_2-37'', CH_2-38'' & CH_2-39), 0.97 (d, *J* = 6.7 Hz, 3H, *C*H_3-41), 0.80 (t, *J* = 7.0 Hz, 3H, *C*H_3-40). *See spectrum*.

^{13}C NMR (151 MHz, Pyr) δ 167.14 (*C*OOR-1), 160.57 (*C*-3 alkene), 117.71 (*C*H-2 alkene), 74.93 (*C*H-35), 72.05 (*C*H-9), 71.84 (*C*H-7), 71.51 (*C*H-29), 71.22 (*C*H-25), 71.18 (*C*H-13), 70.93 (*C*H-21), 69.30 (*C*H-19), 68.75 (*C*H-17), 67.99 (*C*H-33), 45.39 (*C*H_2-20), 45.05 (*C*H_2-8), 45.00 (*C*H_2-18), 39.60 (*C*H_2-32), 39.35 (*C*H_2-10), 39.28(*C*H_2-34), 39.10 (*C*H_2-22), 39.02 (*C*H_2-6), 38.99 (*C*H_2-30), 38.85 (*C*H_2-16 & *C*H_2-28), 38.75 (*C*H_2-26), 38.69 (*C*H_2-12), 38.61 (*C*H_2-24), 38.54 (*C*H_2-14), 37.53 (*C*H-36), 33.92 (*C*H_2-4), 32.89 (*C*H_2-37), 30.07 (*C*H_2-38), 25.26 (*C*H_3-42), 25.15 (*C*H_2-5), 23.51 (*C*H_2-39) (*C*H_2-39), 23.26 (*C*H_2-27 & *C*H_2-31), 22.87 (*C*H_2-11), 22.69 (*C*H_2-15), 22.40 (*C*H_2-23), 15.63 (*C*H_3-41), 14.56 (*C*H_3-40). *See spectrum*.

HRMS (m/z): (ESI) calculated for $C_{42}H_{81}O_{11}$ $[M+H]^+$ 761.5773, found 761.5772.

TLC: R_f = 0.44 (80:20 dichloromethane:methanol, stained with *p*-anisaldehyde)

IR (ν_{max}/cm^{-1}, neat): 3305 (OH), 2924, 2858, 1738 (C=O), 1649 (C=C), 1455, 1366, 1217, 1121.

Table S12: ^{13}C NMR comparison of caylobolide B (**1**) synthetic material to isolated. Spectra recorded in pyridine-d_5, referenced to 123.87 ppm.

**Position, type**	**Isolated δ^{13}C (151 MHz, pyr-*d_5*)**	**Synthetic δ^{13}C (151 MHz, pyr-*d_5*)**	**Δδ^{13}C (Isolation – synthetic)**
**1 C**	167.13	167.14	-0.01
**2 CH**	117.69	117.71	-0.02
**3 C**	160.57	160.57	0
**4 CH_2**	33.91	33.92	-0.01
**5 CH_2**	25.13	25.15	-0.02
**6 CH_2**	38.99	39.02	-0.03
**7 CHOH**	71.81	71.84	-0.03
**8 CH_2**	45.03	45.05	-0.02
**9 CHOH**	72.01	72.05	-0.04
**10 CH_2**	39.32	39.35	-0.03
**11 CH_2**	22.84	22.87	-0.03
**12 CH_2**	38.66	38.69	-0.03
**13 CHOH**	71.17	71.18	-0.01
**14 CH_2**	38.51	38.54	-0.03
**15 CH_2**	22.66	22.69	-0.03
**16 CH_2**	38.82	38.85	-0.03
**17 CHOH**	68.73	68.75	-0.02
**18 CH_2**	44.98	45	-0.02
**19 CHOH**	69.25	69.3	-0.05
**20 CH_2**	45.38	45.39	-0.01
**21 CHOH**	70.89	70.93	-0.04
**22 CH_2**	39.06	39.1	-0.04
**23 CH_2**	22.38	22.4	-0.02
**24 CH_2**	38.57	38.61	-0.04
**25 CHOH**	71.2	71.22	-0.02
**26 CH_2**	38.71	38.75	-0.04
**27 CH_2**	23.23	23.26	-0.03
**28 CH_2**	38.8	38.85	-0.05
**29 CHOH**	71.5	71.51	-0.01
**30 CH_2**	38.95	38.99	-0.04
**31 CH_2**	23.23	23.26	-0.03
**32 CH_2**	39.57	39.6	-0.03
**33 CHOH**	67.98	67.99	-0.01
**34 CH_2**	39.26	39.28	-0.02
**35 CHOR**	74.92	74.93	-0.01
**36 CH**	37.51	37.53	-0.02
**37 CH_2**	32.87	32.89	-0.02
**38 CH_2**	30.05	30.07	-0.02
**39 CH_2**	23.49	23.51	-0.02
**40 CH_3**	14.55	14.56	-0.01
**41 CH_3**	15.62	15.63	-0.01
**42 CH_3**	25.25	25.26	-0.01

5 REFERENCES

[1] L. A. Salvador, V. J. Paul, H. Luesch, *J. Nat. Prod.* **2010**, *73*, 1606-1609.

[2] L. T. Kliman, S. N. Mlynarski, J. P. Morken, *J. Am. Chem. Soc.* **2009**, *131*, 13210-13211.

[3] A. F. Burchat, J. M. Chong, N. Nielsen, *J. Organomet. Chem.* **1997**, *542*, 281-283.

[4] A. Krasovskiy, P. Knochel, *Synthesis* **2006**, *2006*, 0890-0891.

[5] P. Beak, N. A. Nikolic, *Org. Synth.* **1997**, 23.

[6] a) R. Larouche-Gauthier, C. J. Fletcher, I. Couto, V. K. Aggarwal, *Chem. Commun.* **2011**, *47*, 12592-12594; b) M. Burns, S. Essafi, J. R. Bame, S. P. Bull, M. P. Webster, S. Balieu, J. W. Dale, C. P. Butts, J. N. Harvey, V. K. Aggarwal, *Nature* **2014**, *513*, 183-188.

[7] M. R. P. George, M. Deering, D. Fiorito, K. Solomon, K. Tidgewell, A. Noble, C. P. Butts, V. K. Aggarwal, *Nat. Synth.* **2025**, *4,* 859-868.

[8] D. Fiorito, S. Keskin, J. M. Bateman, M. George, A. Noble, V. K. Aggarwal, *J. Am. Chem. Soc.* **2022**, *144*, 7995-8001.

[9] J. M. Cherry, E. L. Hong, C. Amundsen, R. Balakrishnan, G. Binkley, E. T. Chan, K. R. Christie, M. C. Costanzo, S. S. Dwight, S. R. Engel, D. G. Fisk, J. E. Hirschman, B. C. Hitz, K. Karra, C. J. Krieger, S. R. Miyasato, R. S. Nash, J. Park, M. S. Skrzypek, M. Simison, S. Weng, E. D. Wong, *Nucleic Acids Res* **2012**, *40*, D700-D705.

[10] G. Casoni, M. Kucukdisli, J. M. Fordham, M. Burns, E. L. Myers, V. K. Aggarwal, *J. Am. Chem. Soc.* **2017**, *139*, 11877-11886.

[11] Y. Yamamoto, R. Fujikawa, T. Umemoto, N. Miyaura, *Tetrahedron* **2004**, *60*, 10695-10700.

[12] A. Bonet, C. Pubill-Ulldemolins, C. Bo, H. Gulyás, E. Fernández, *Angew. Chem. Int. Ed.* **2011**, *50*, 7158-7161.

[13] P. Beak, L. G. Carter, *J. Org. Chem.* **1981**, *46*, 2363-2373.

6 SPECTRAL APPENDIX

^{1}H NMR (400 MHz, $CDCl_3$) of compound **S1**. *See procedure*.

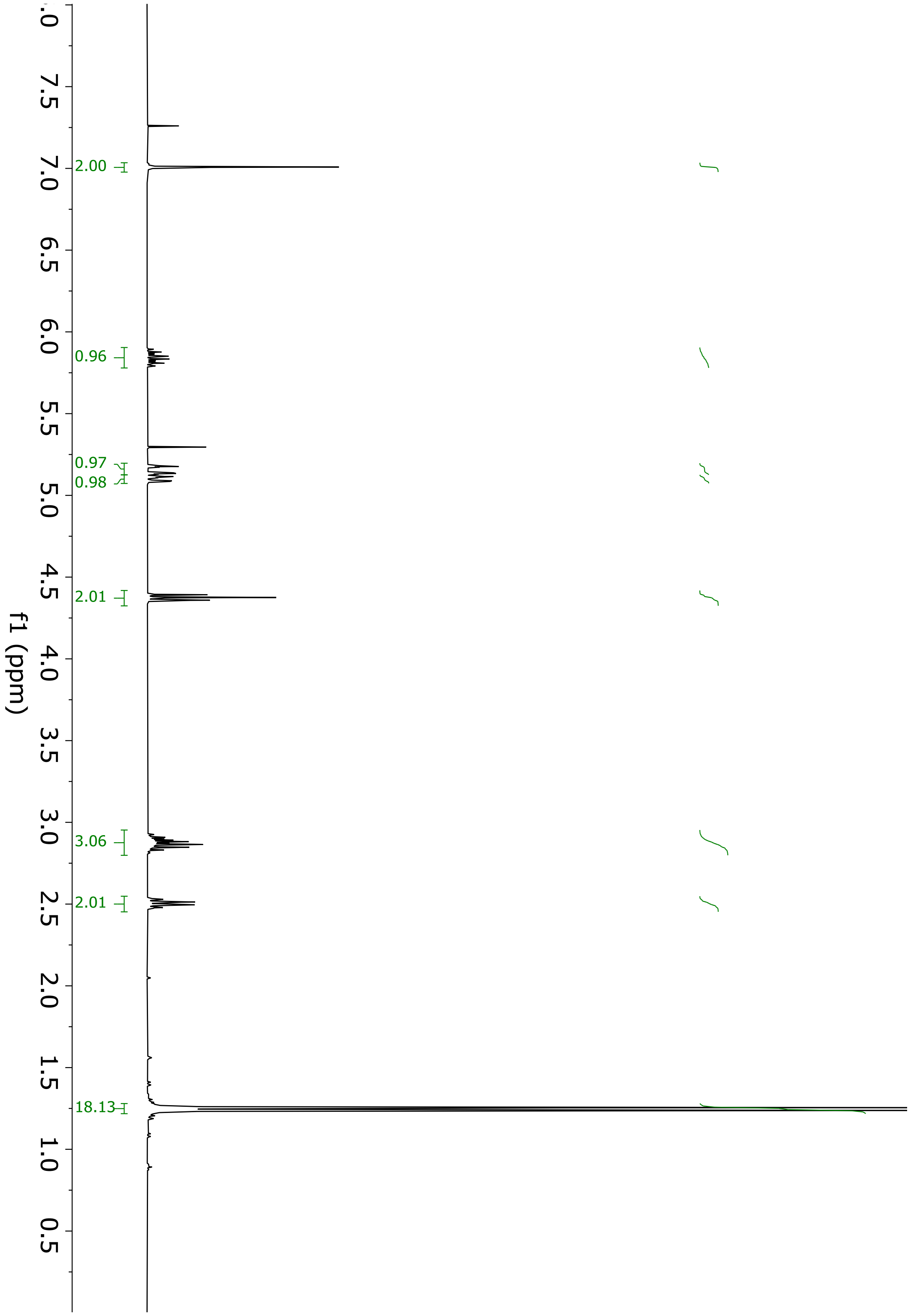

^{13}C NMR (101 MHz, $CDCl_3$) of compound **S1**. *See procedure*.

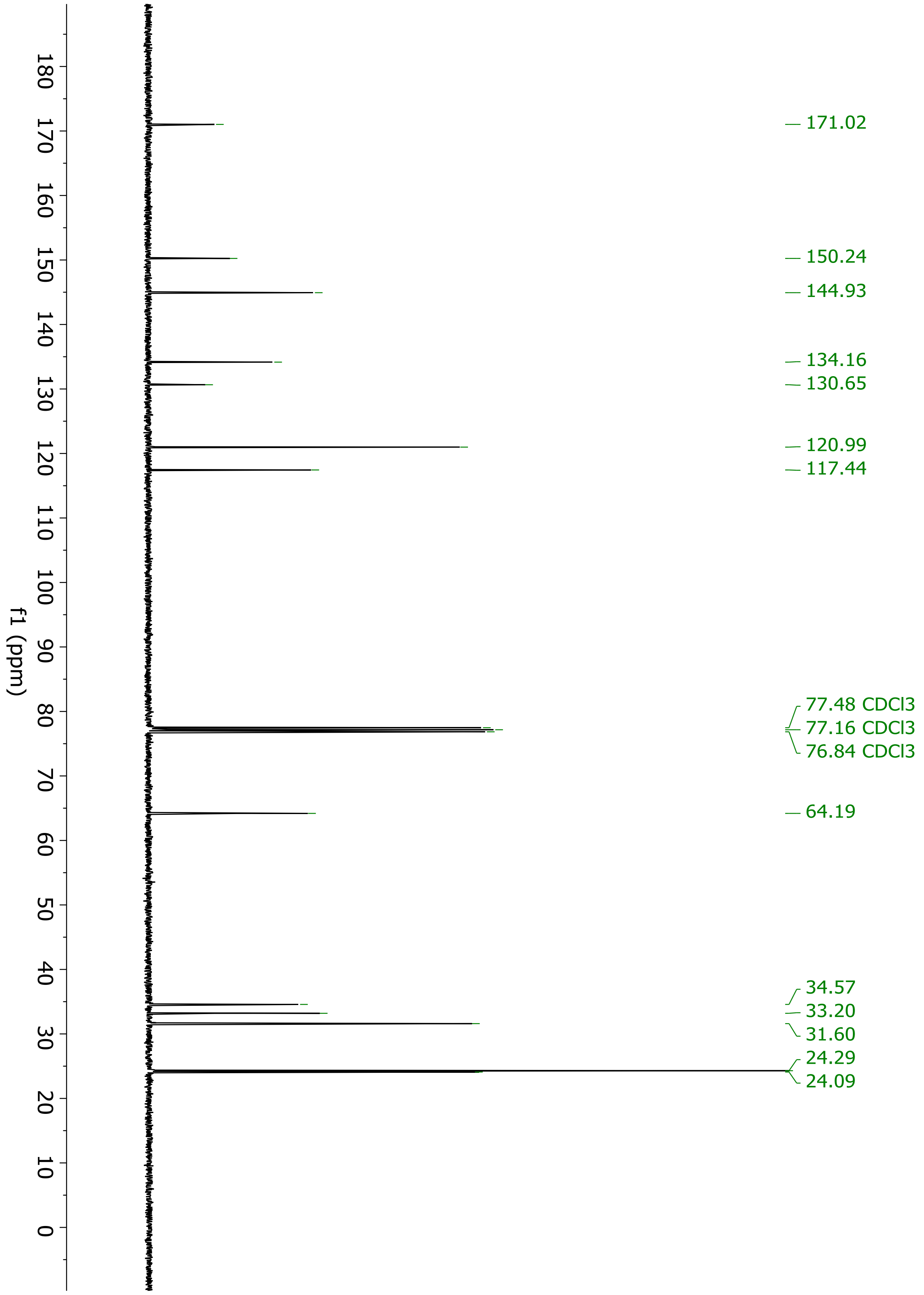

^{1}H NMR (400 MHz, CDCl3) of compound **S2**. *See procedure.*

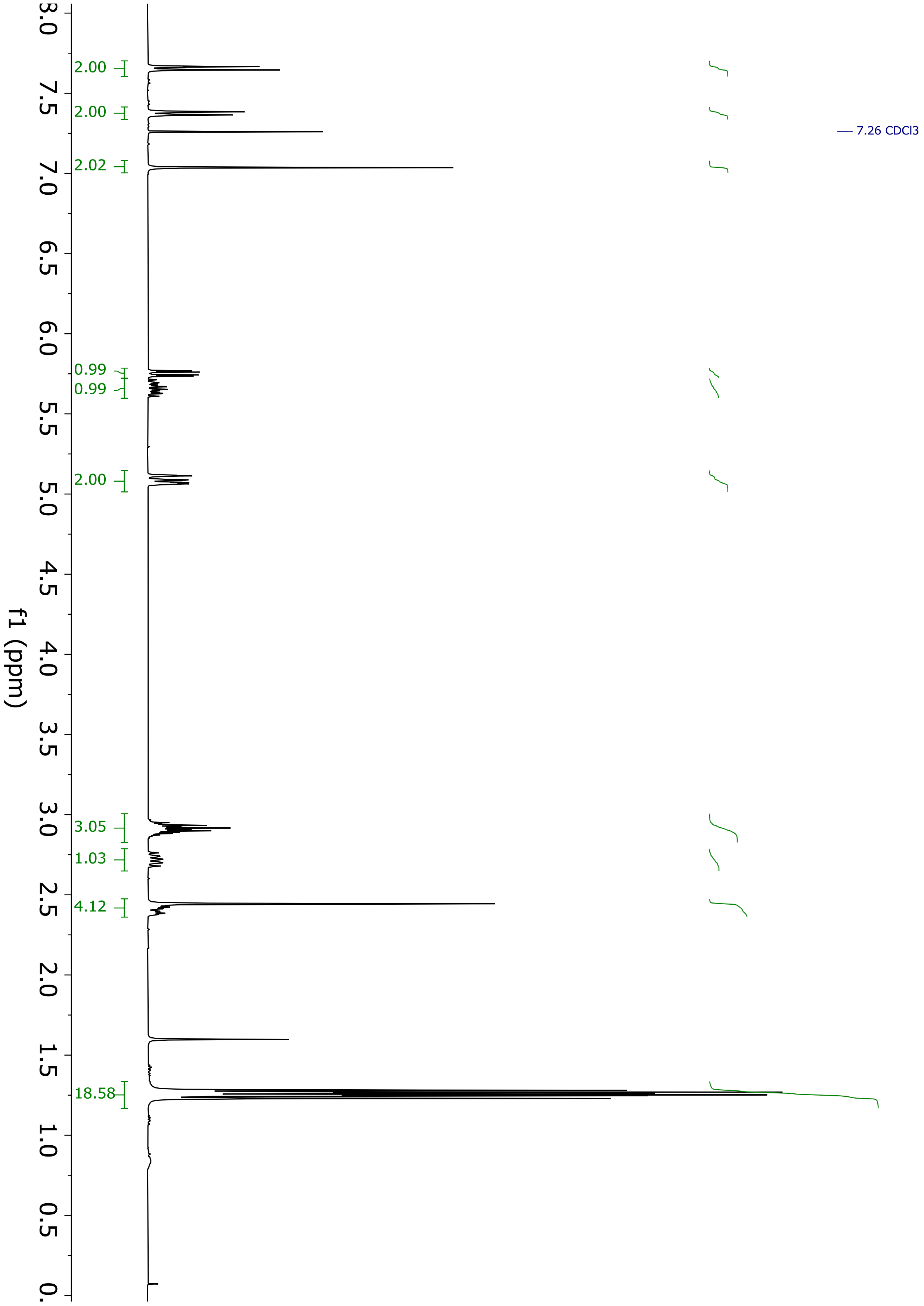

^{13}C NMR (101 MHz, $CDCl_3$) of compound **S2**. See procedure.

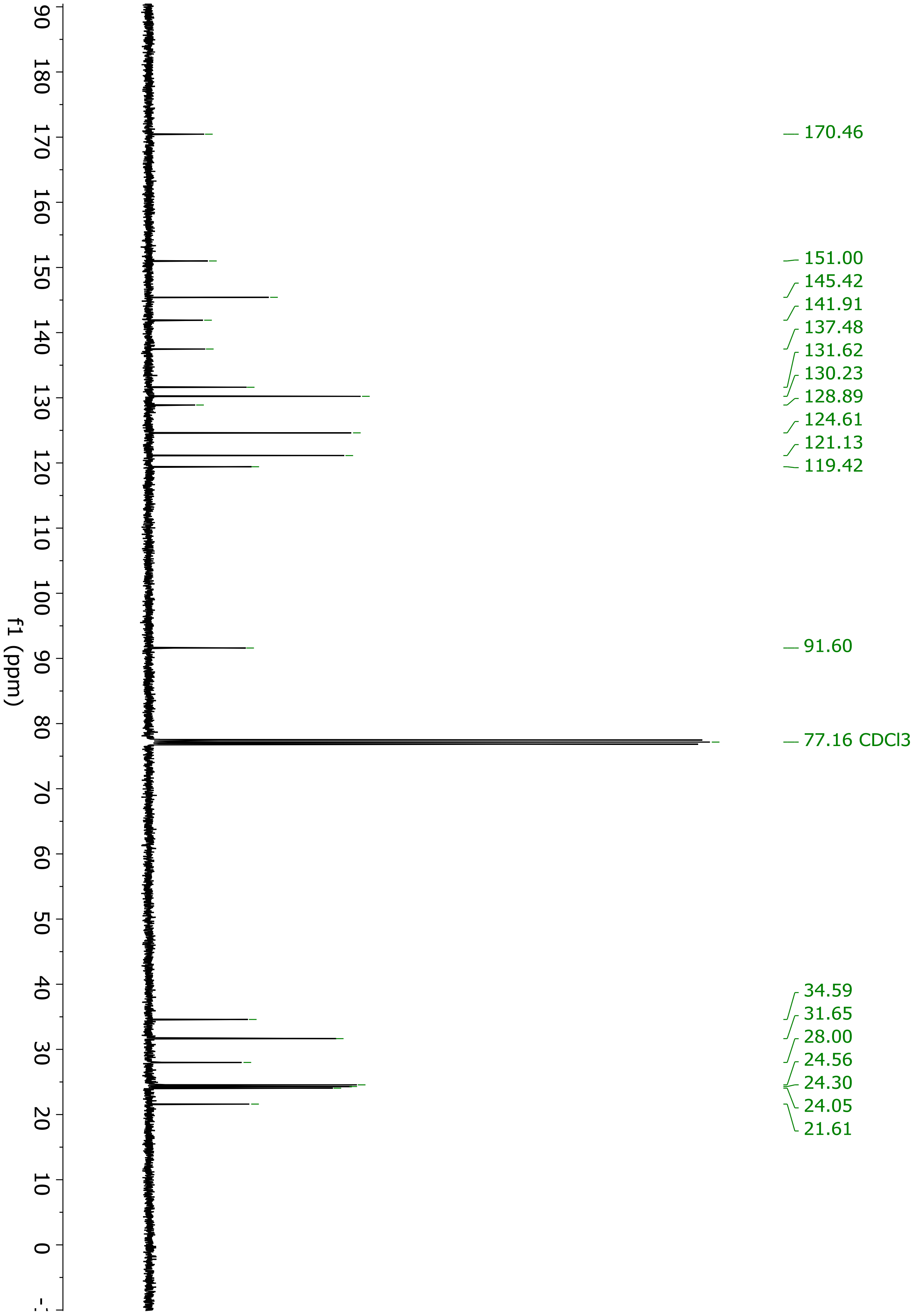

^{1}H NMR (400 MHz, CDCl3) of compound **20**. See procedure.

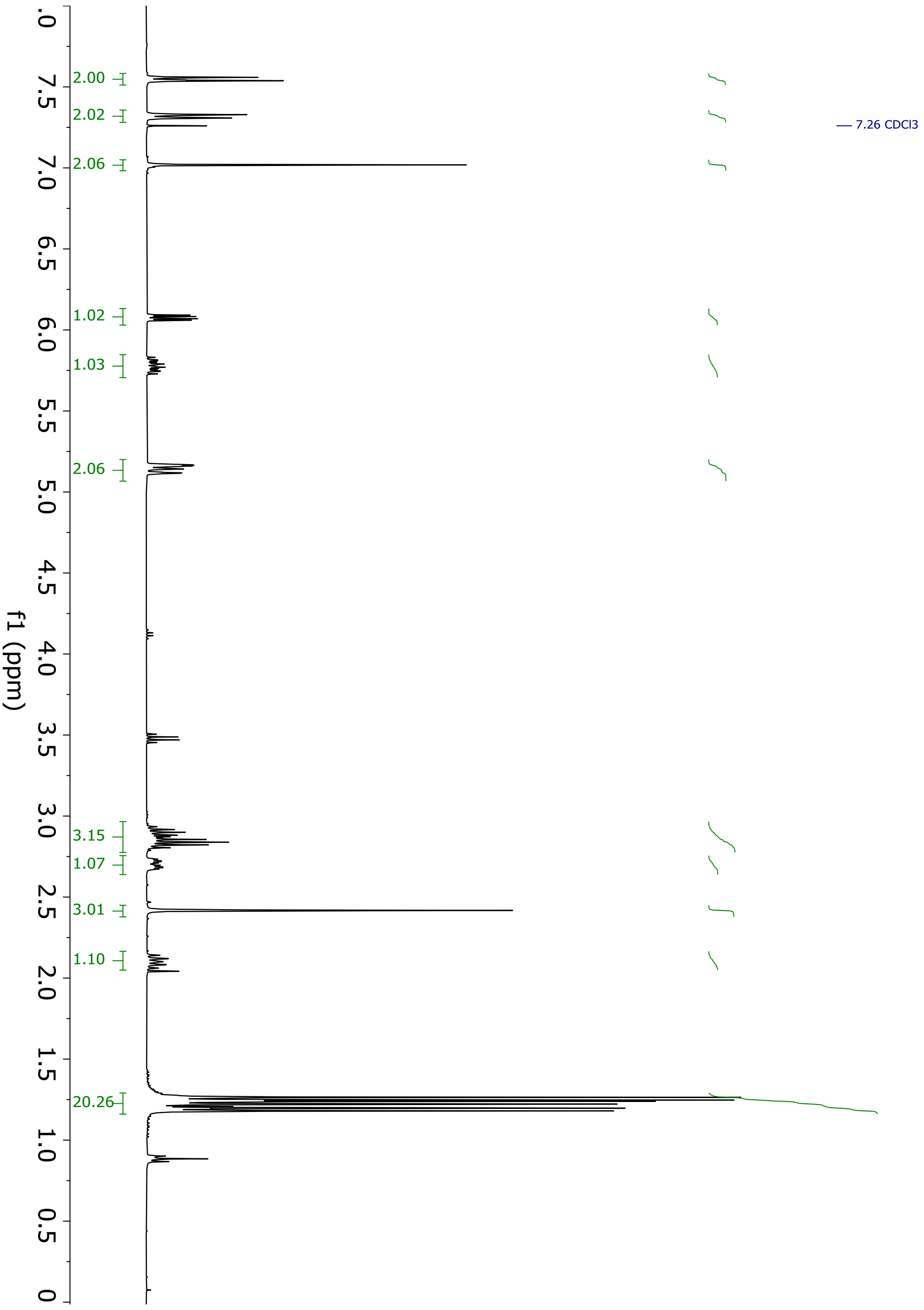

^{13}C NMR (101 MHz, $CDCl_3$) of compound **20**. *See procedure.*

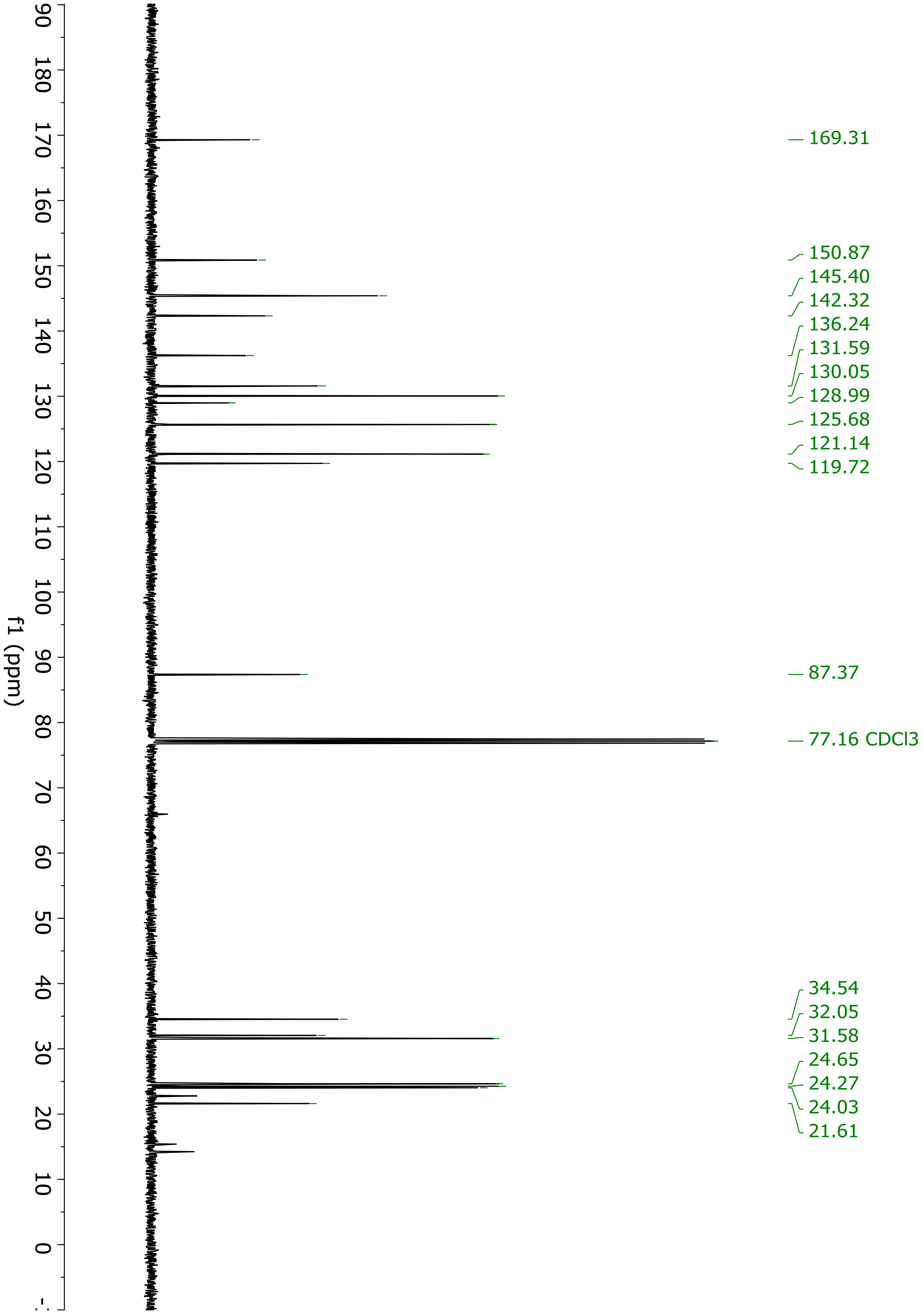

^{1}H NMR (400 MHz, $CDCl_3$) of compound **18**. *See procedure*.

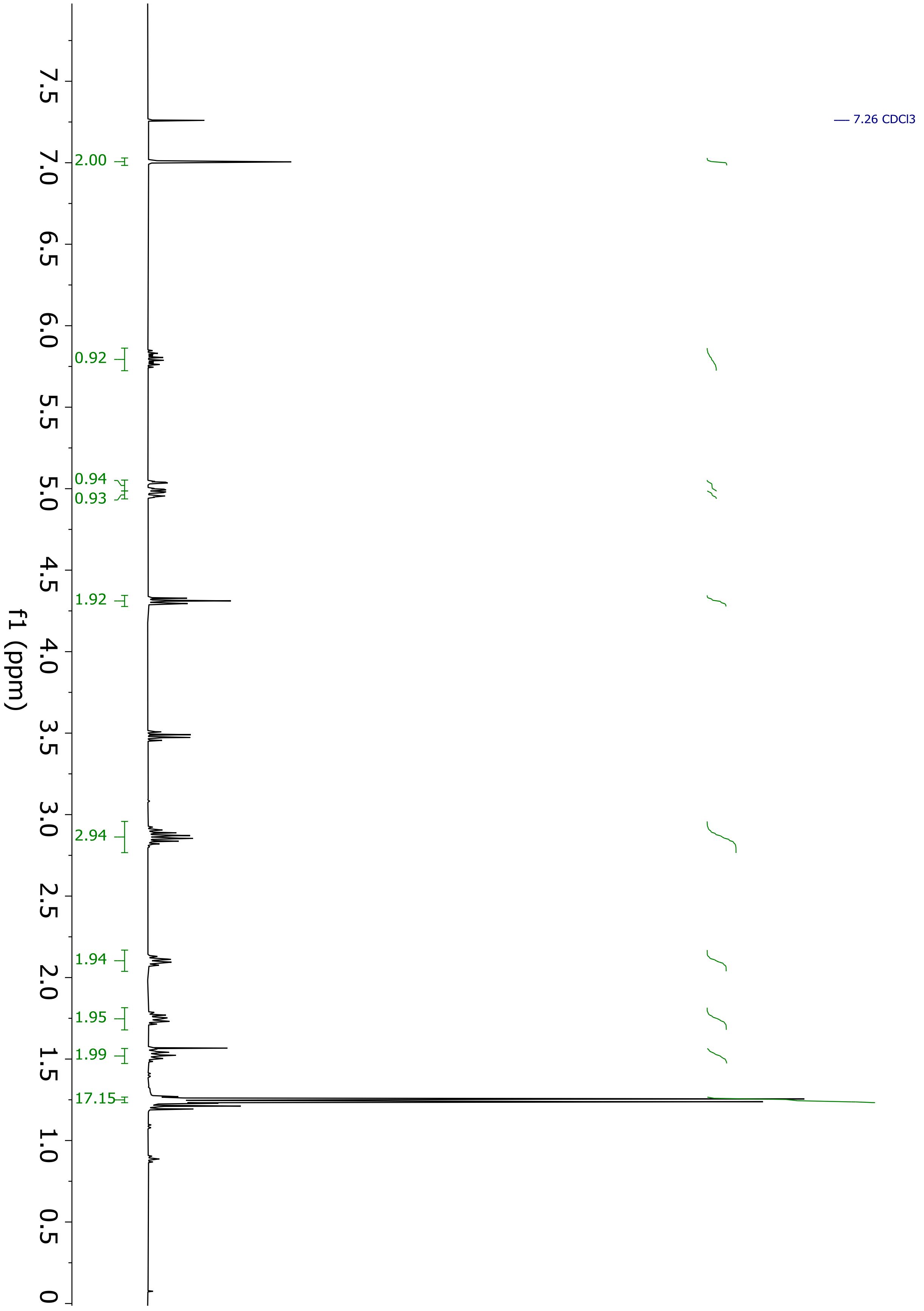

^{13}C NMR (101 MHz, $CDCl_3$) of compound **18**. *See procedure*.

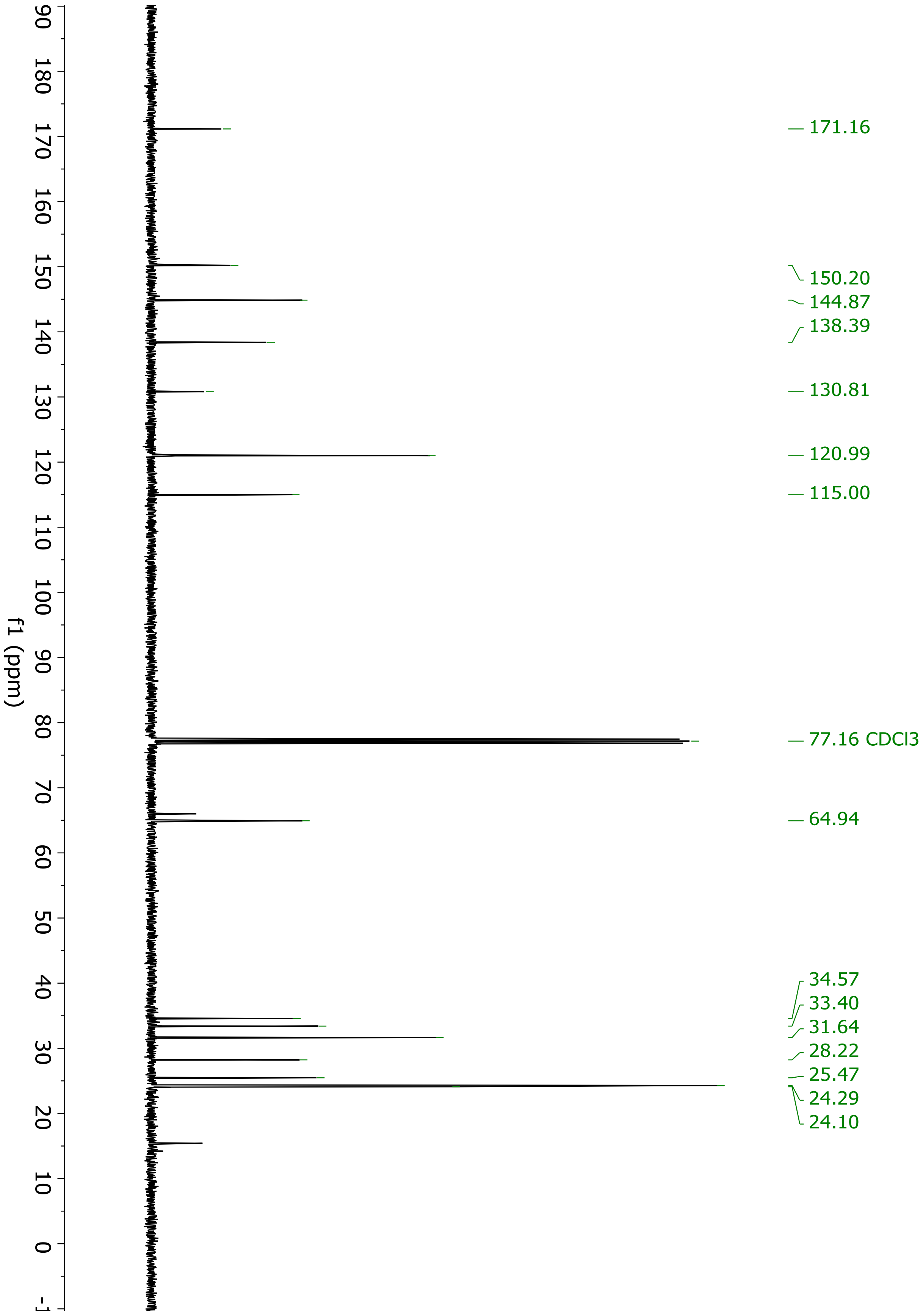

^{1}H NMR (400 MHz, $CDCl_3$) of compound **19**. *See procedure*.

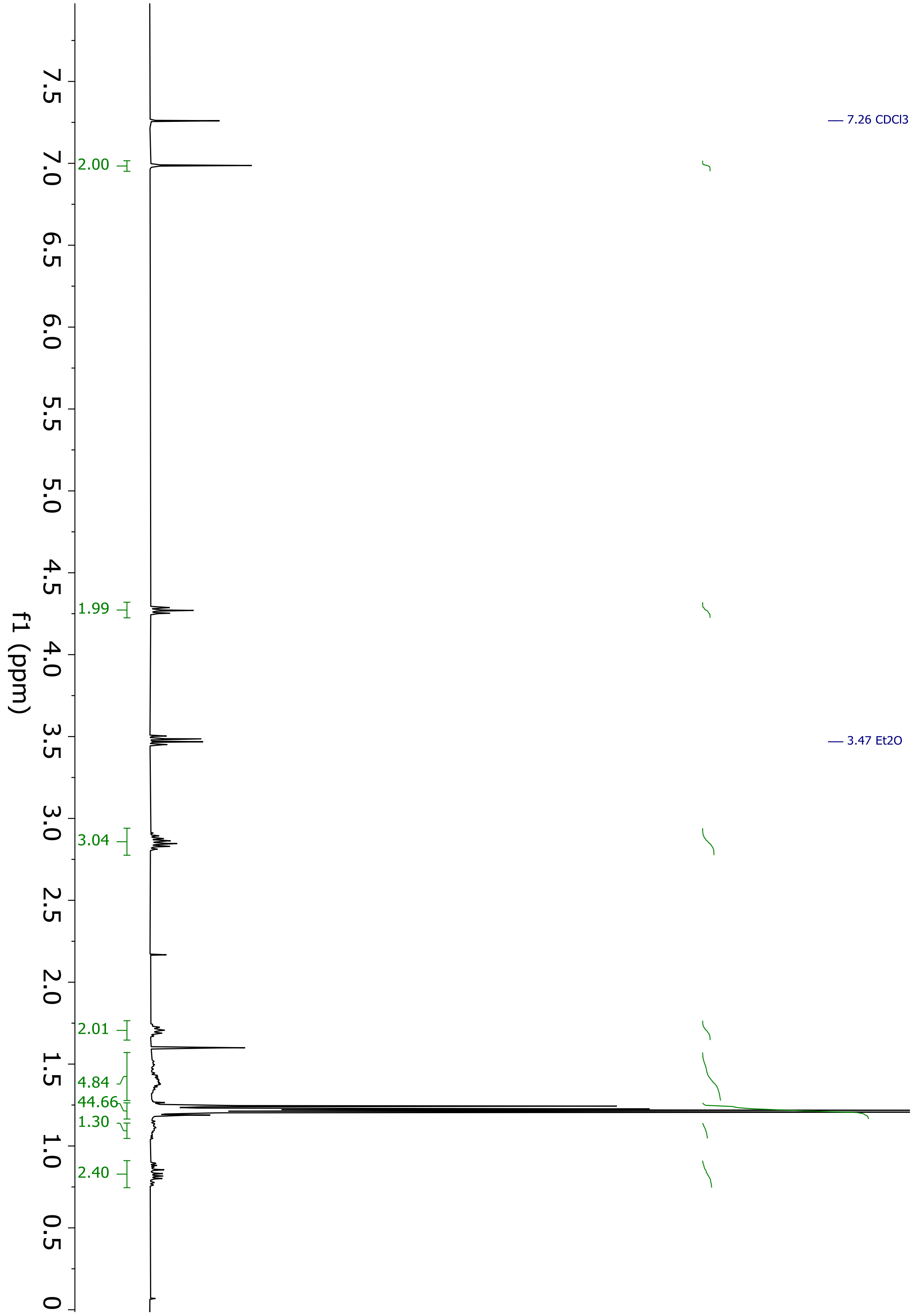

^{13}C NMR (101 MHz, $CDCl_3$) of compound **19**. *See procedure.*

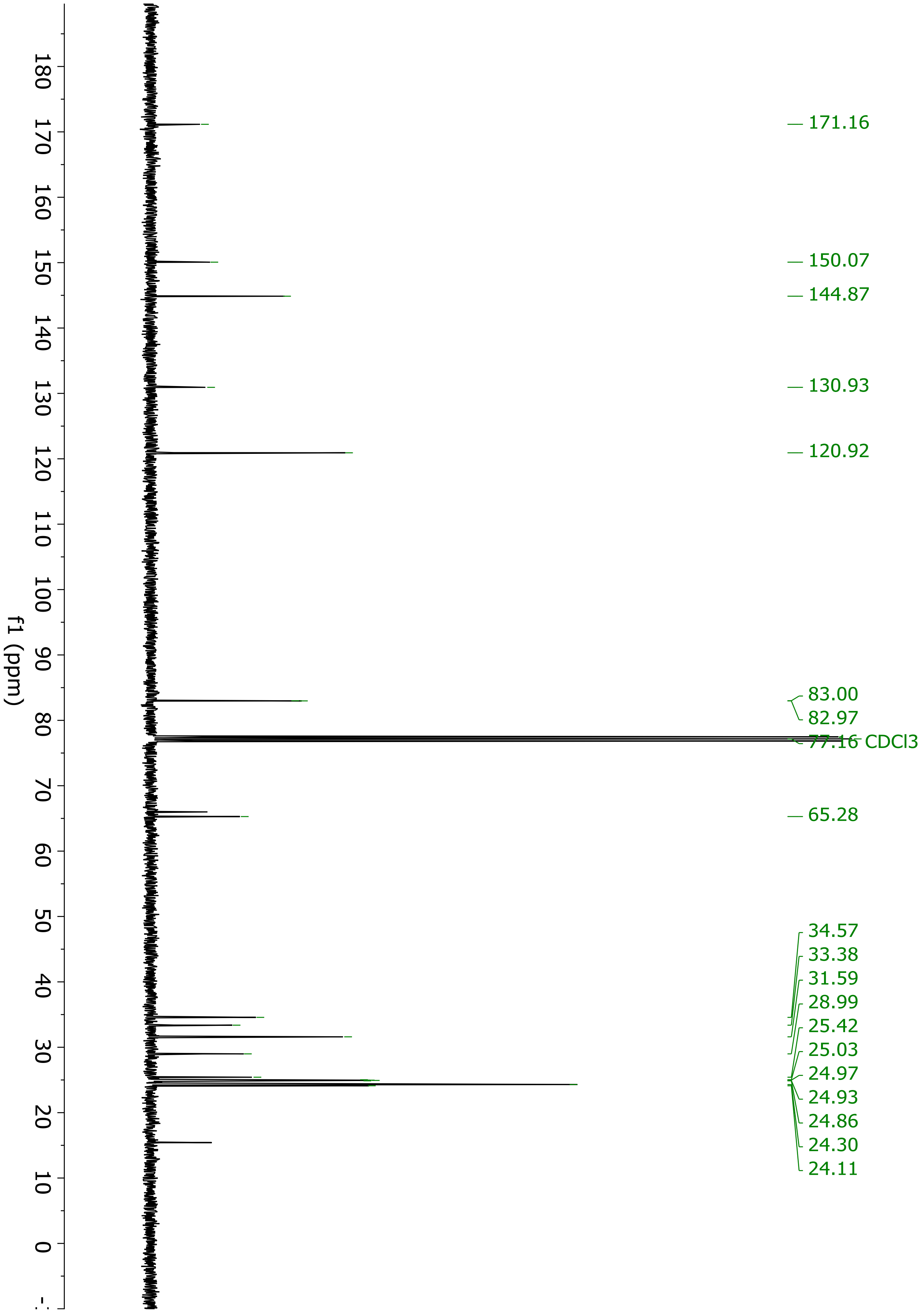

^{1}H NMR (400 MHz, $CDCl_3$) of compound **14**. See procedure.

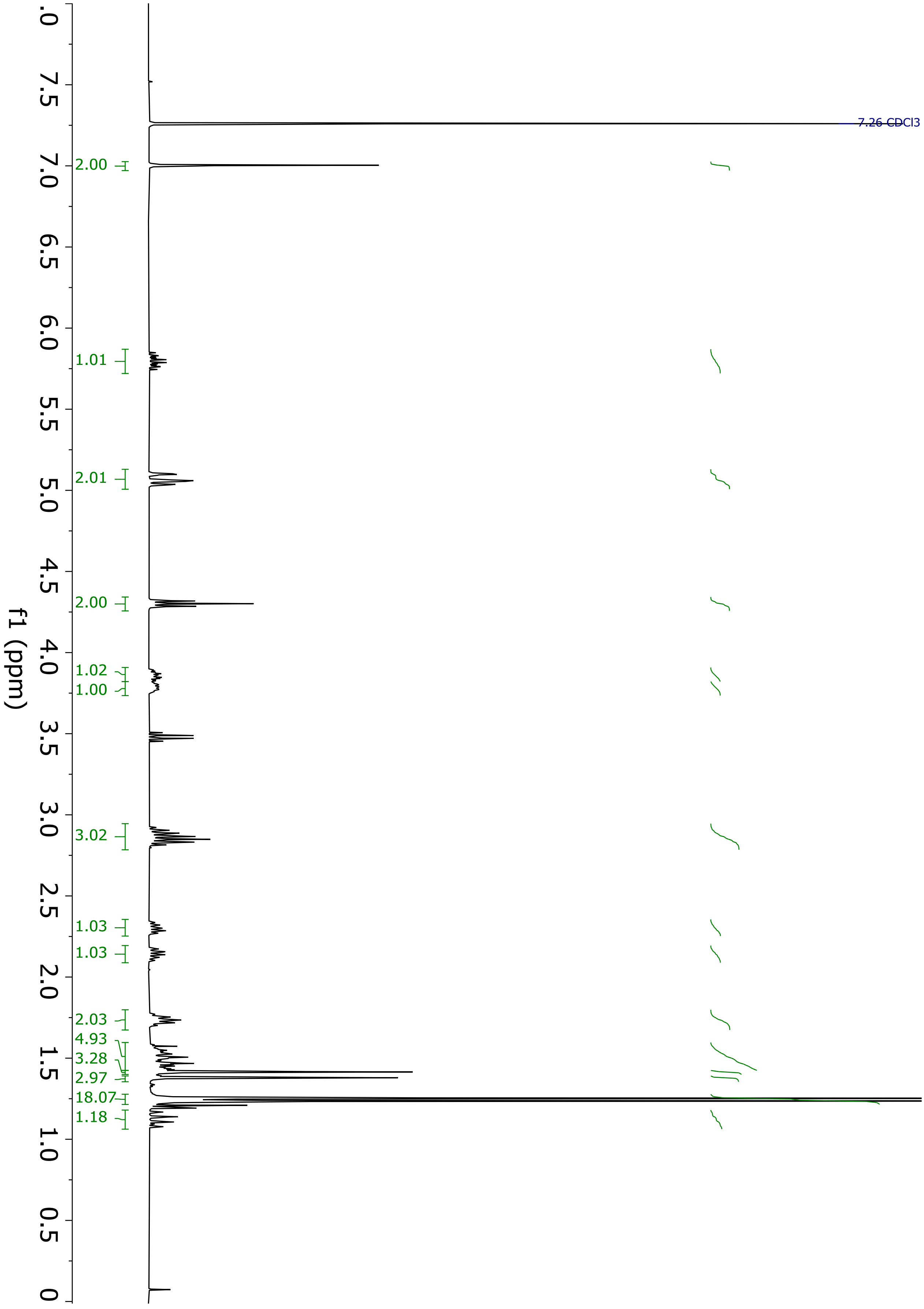

^{13}C NMR (101 MHz, $CDCl_3$) of compound **14**. See procedure.

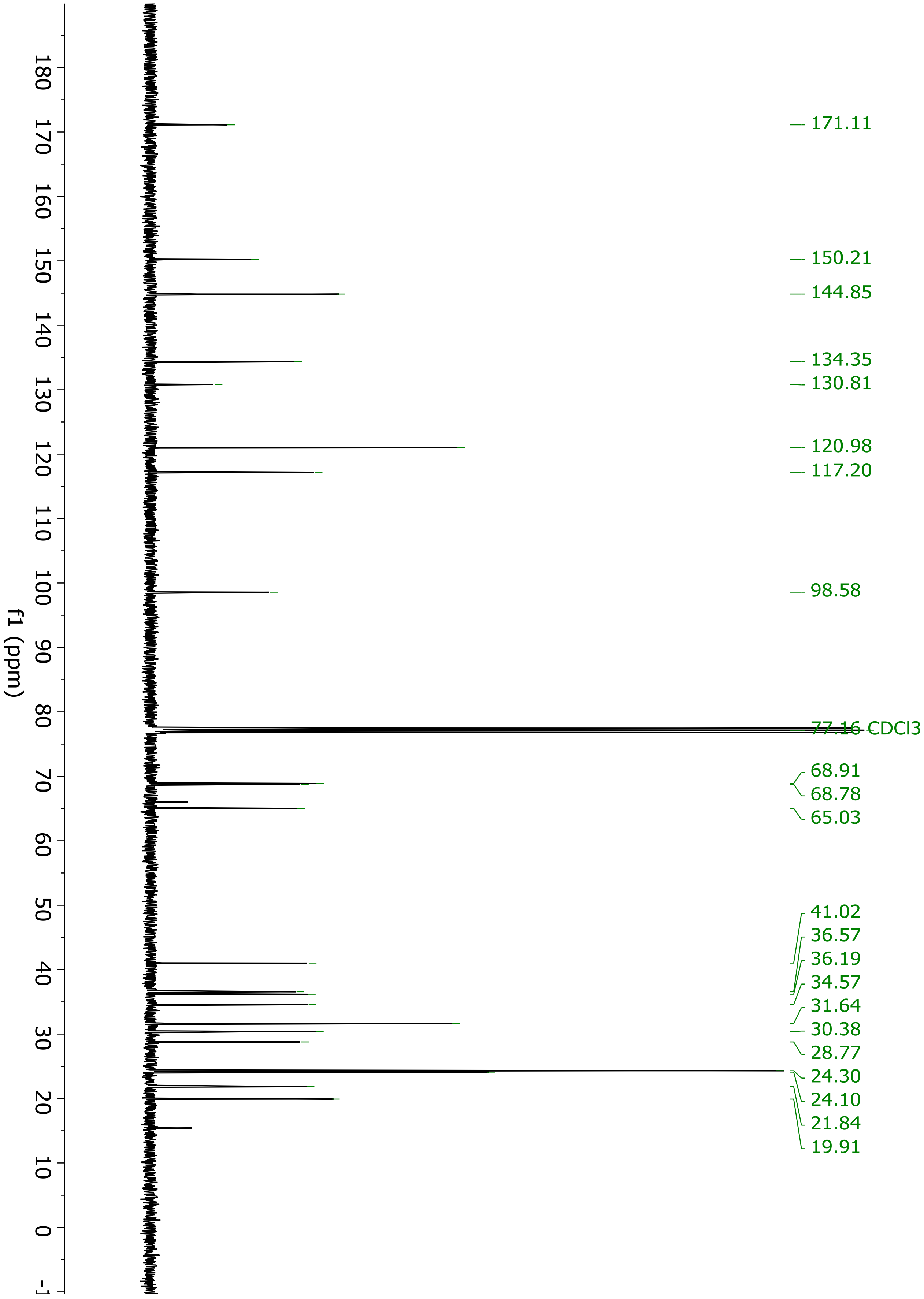

^{1}H NMR (400 MHz, $CDCl_3$) of compound **S5**. *See procedure.*

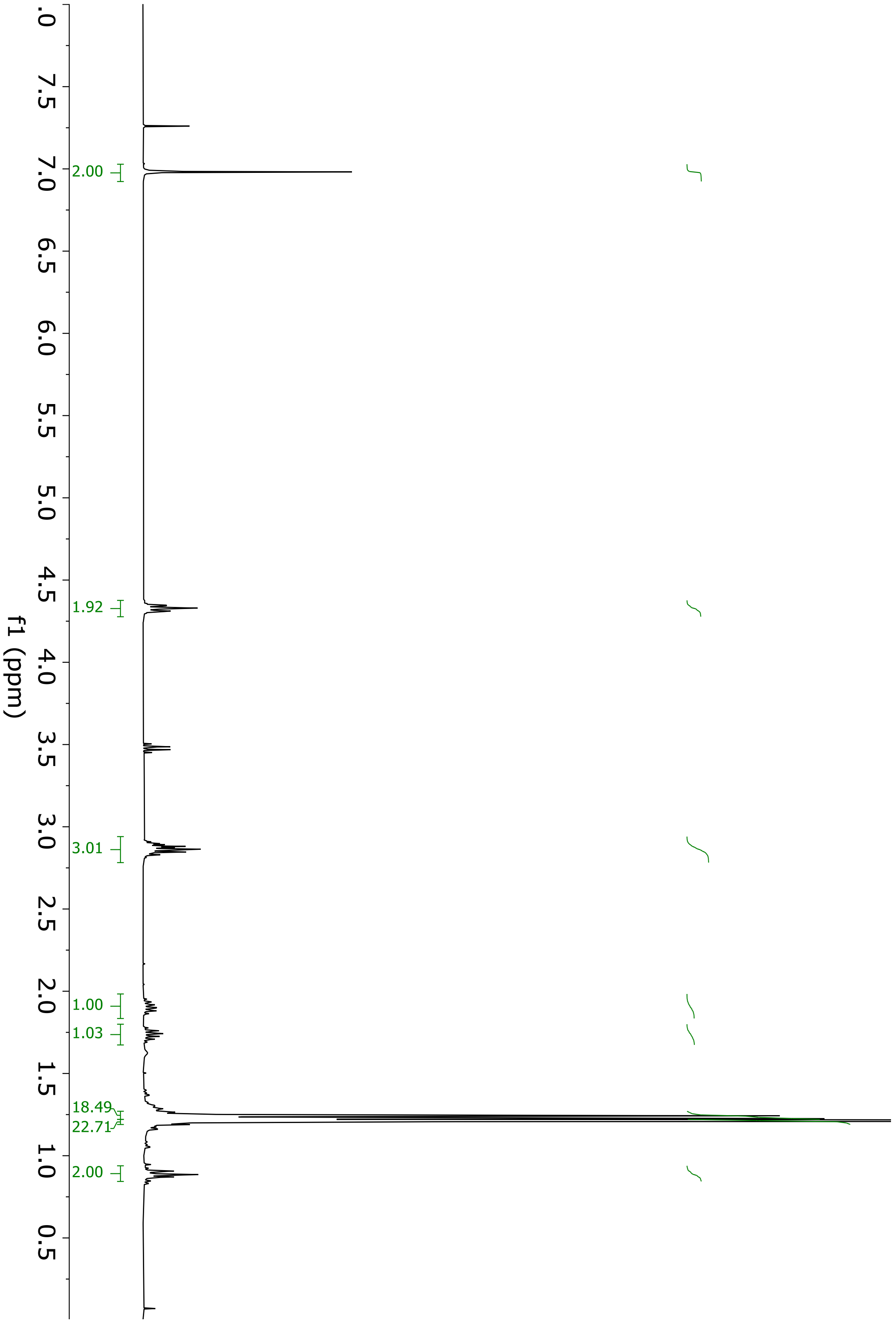

^{13}C NMR (101 MHz, $CDCl_3$) of compound **S5**. *See procedure*.

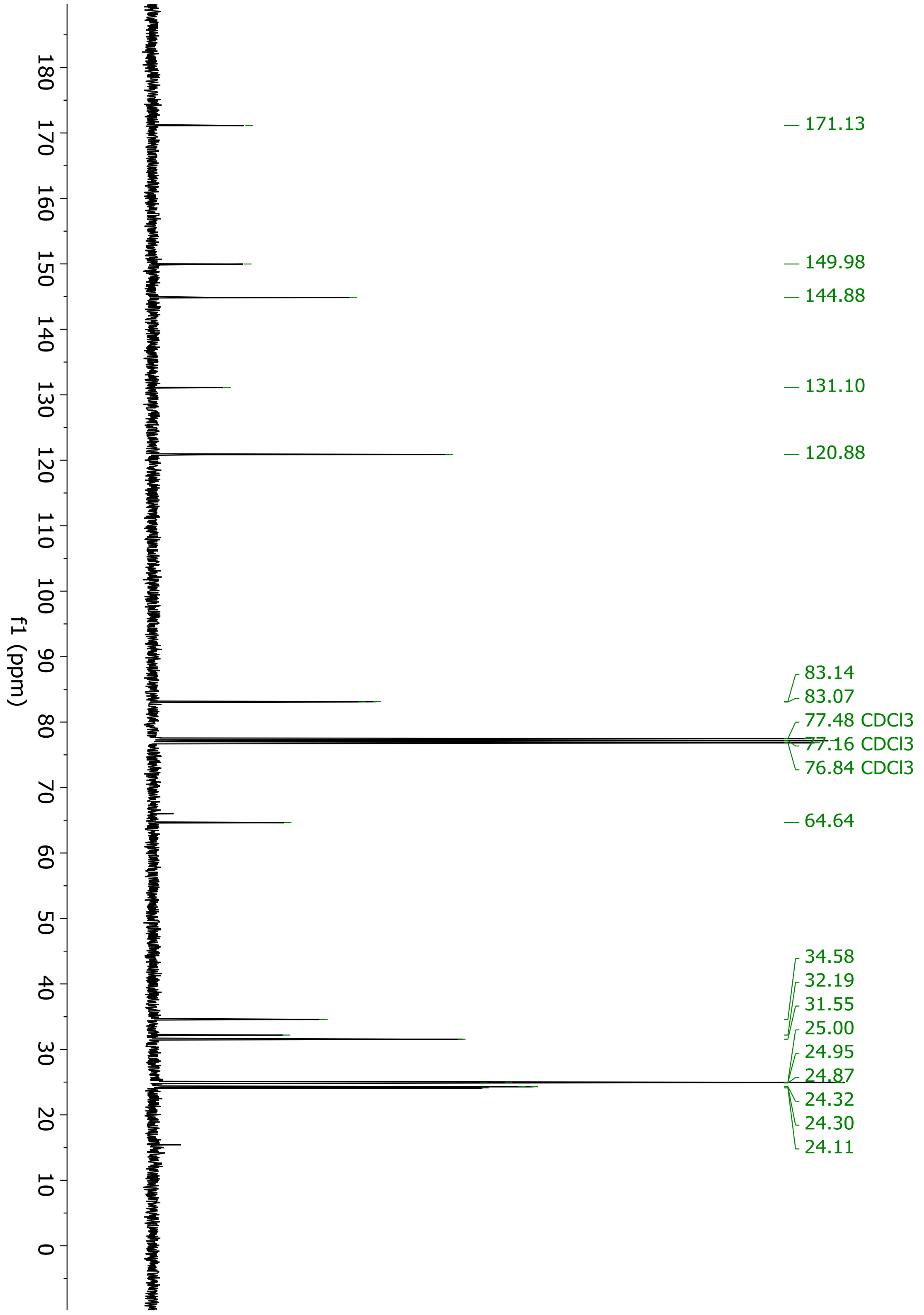

^{1}H NMR (400 MHz, $CDCl_3$) of compound **S7**. *See procedure.*

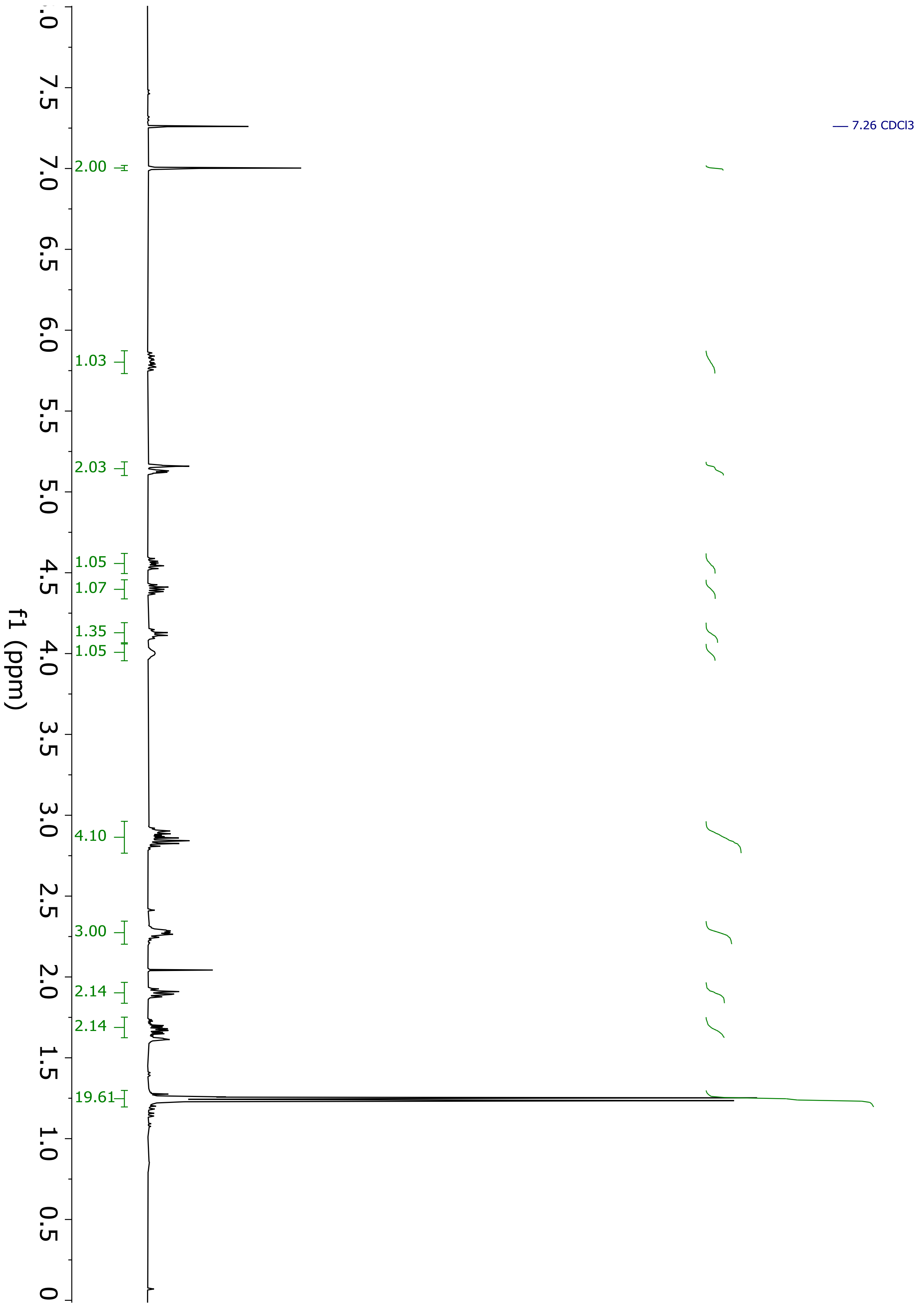

^{13}C NMR (101 MHz, $CDCl_3$) of compound **S7**. *See procedure*.

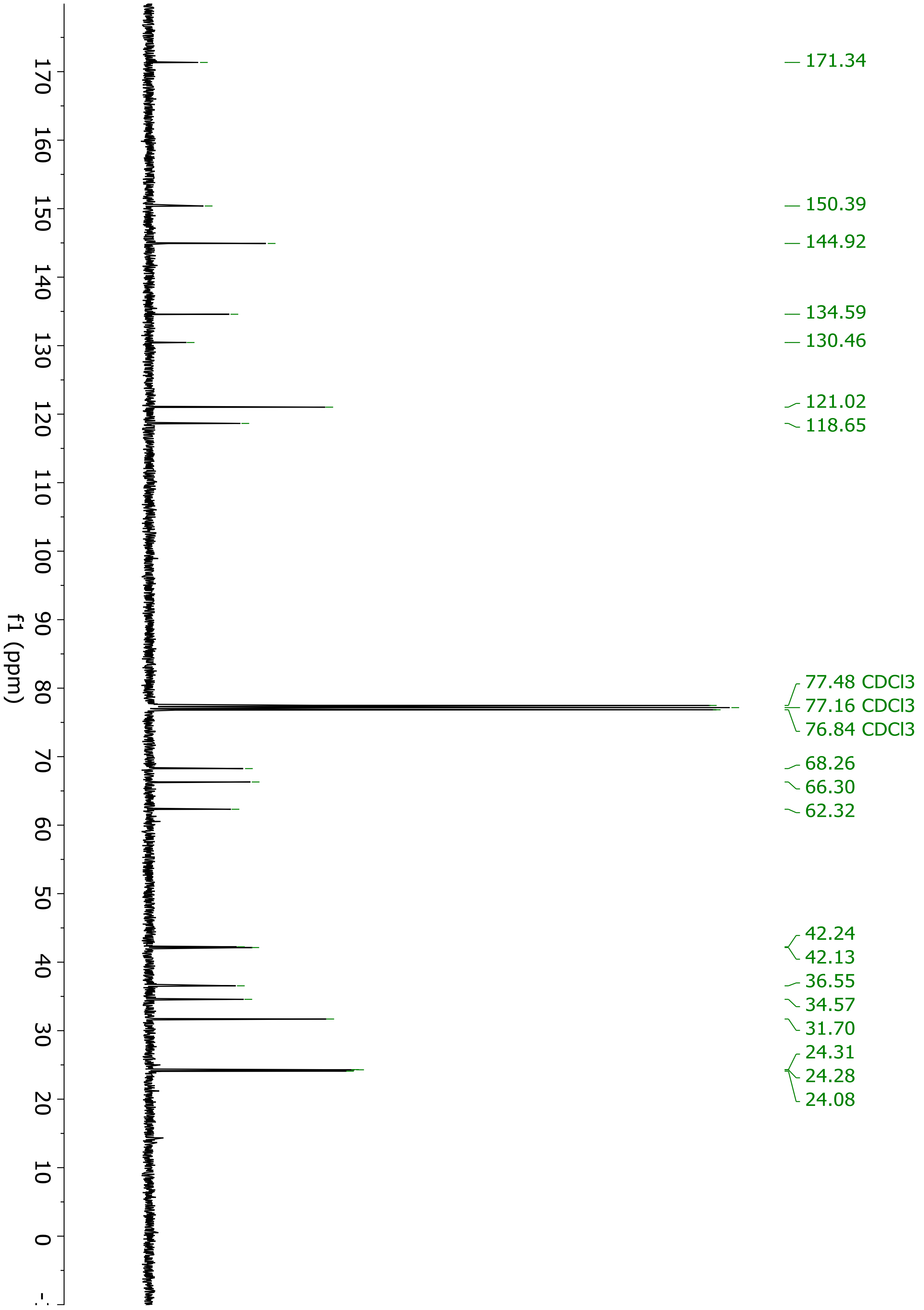

^{1}H NMR (400 MHz, $CDCl_3$) of compound **S8**. *See procedure.*

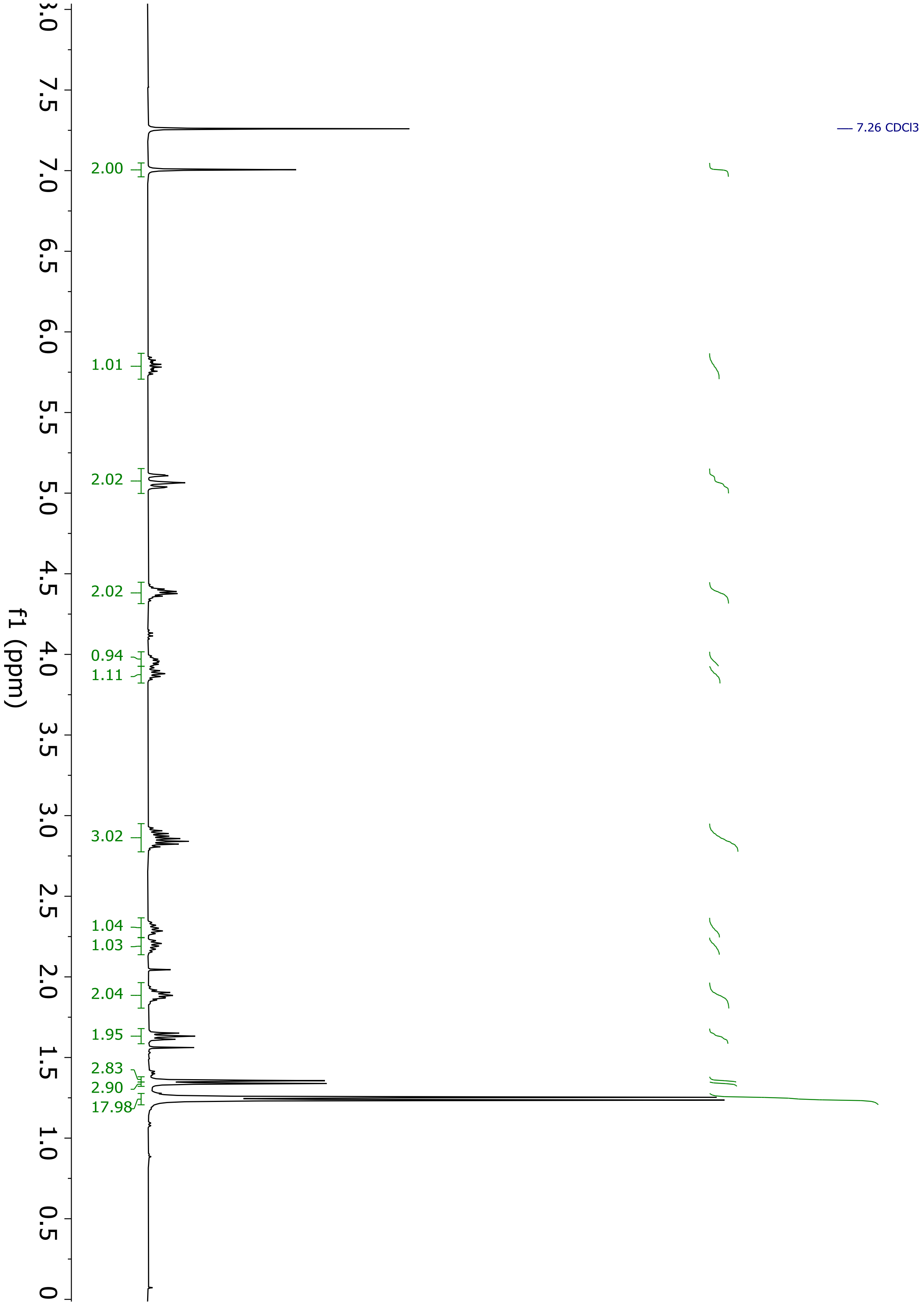

^{13}C NMR (101 MHz, $CDCl_3$) of compound **S8**. *See procedure*.

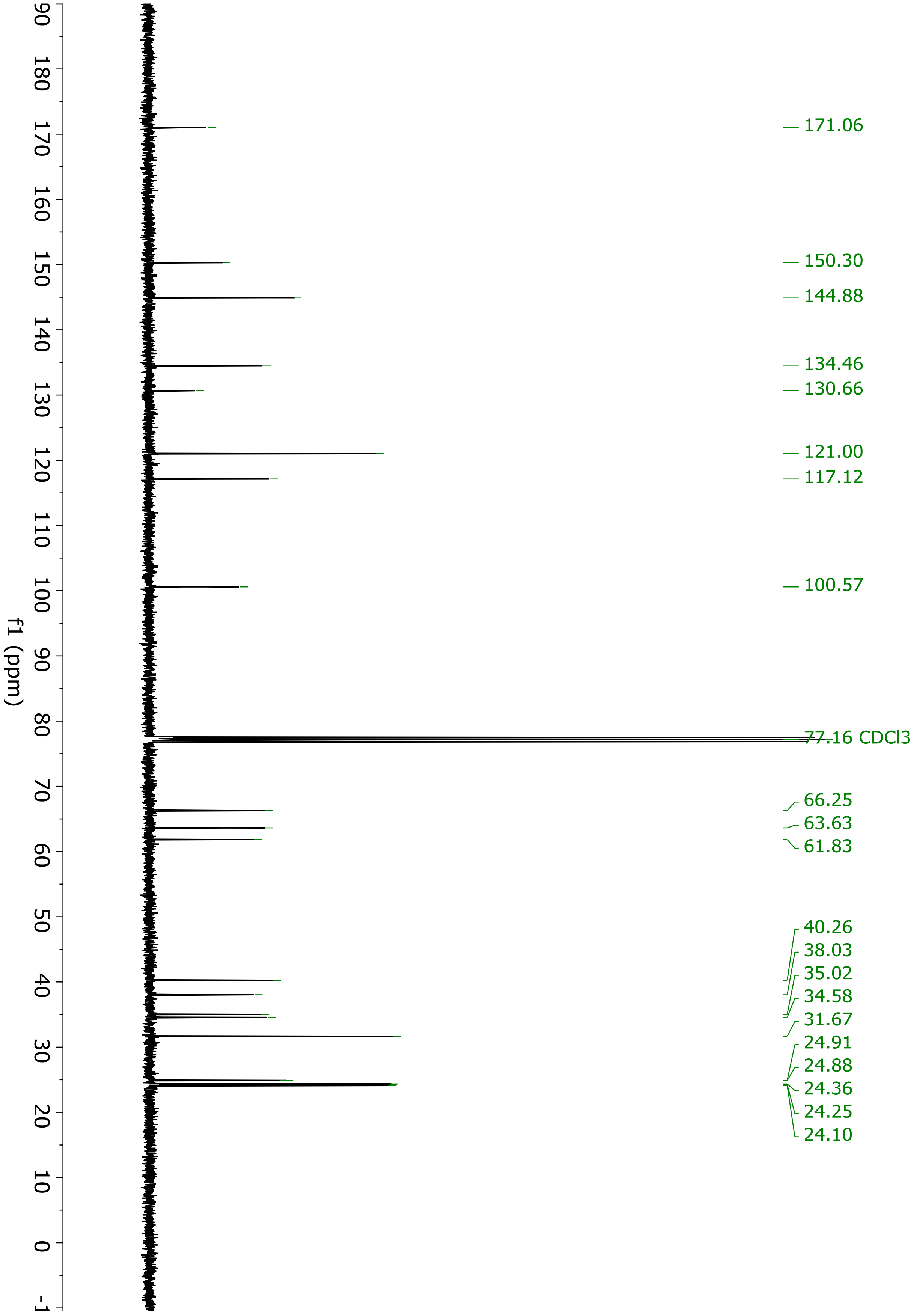

^{1}H NMR (400 MHz, $CDCl_3$) of **15**. *See procedure*.

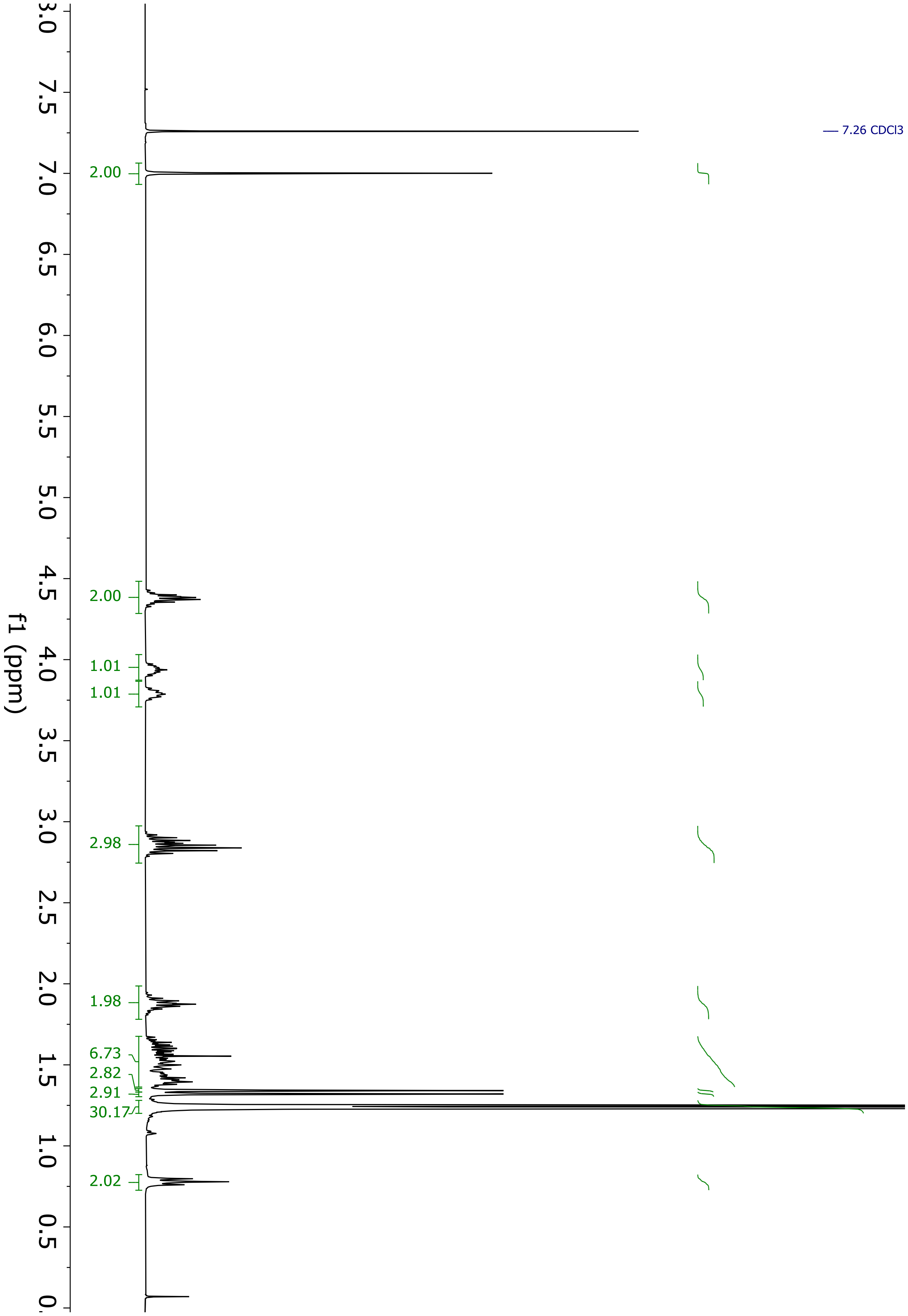

^{13}C NMR (101 MHz, $CDCl_3$) of **15**. *See procedure*.

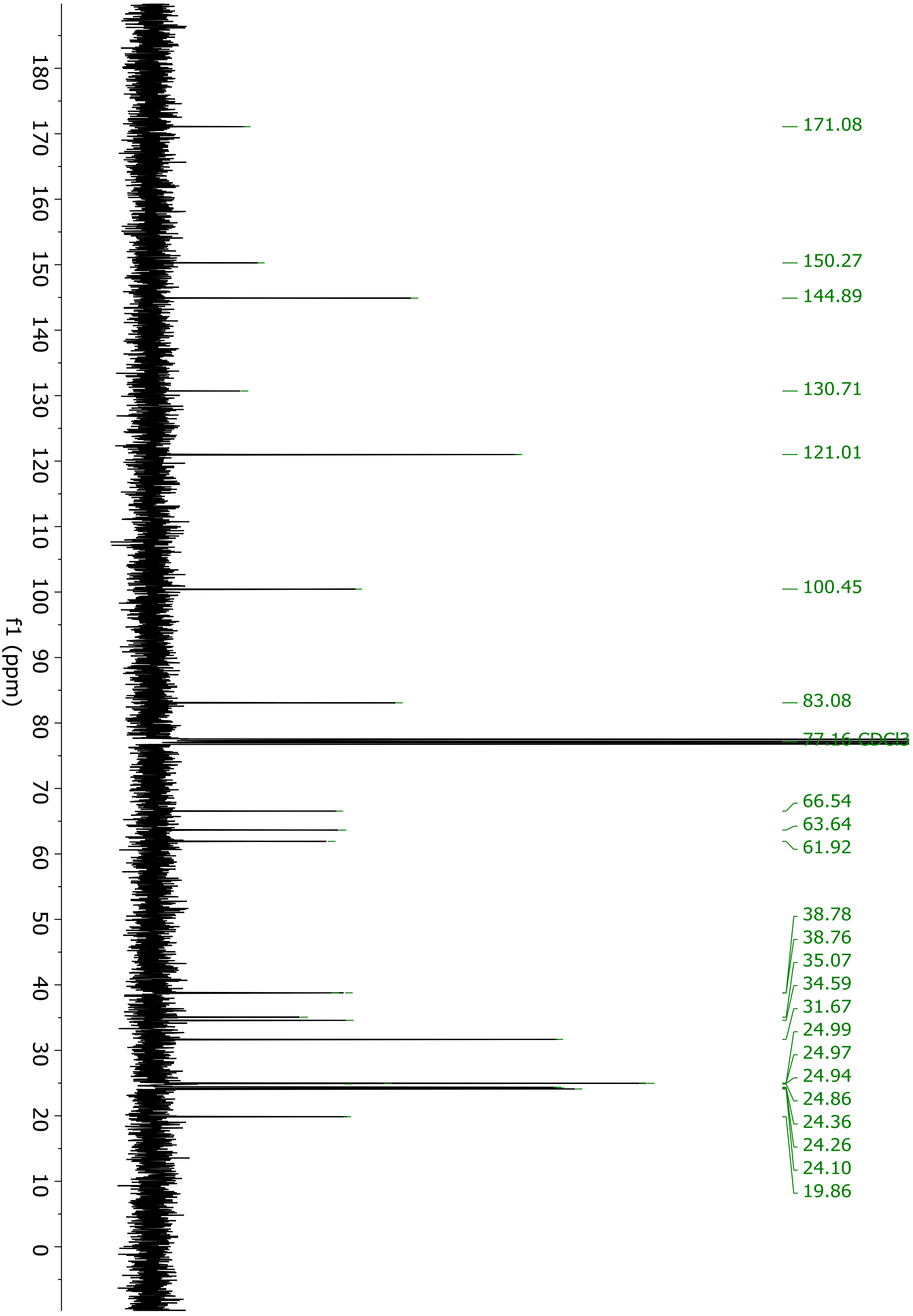

^{1}H NMR (400 MHz, $CDCl_3$) of compound **S9**. *See procedure*.

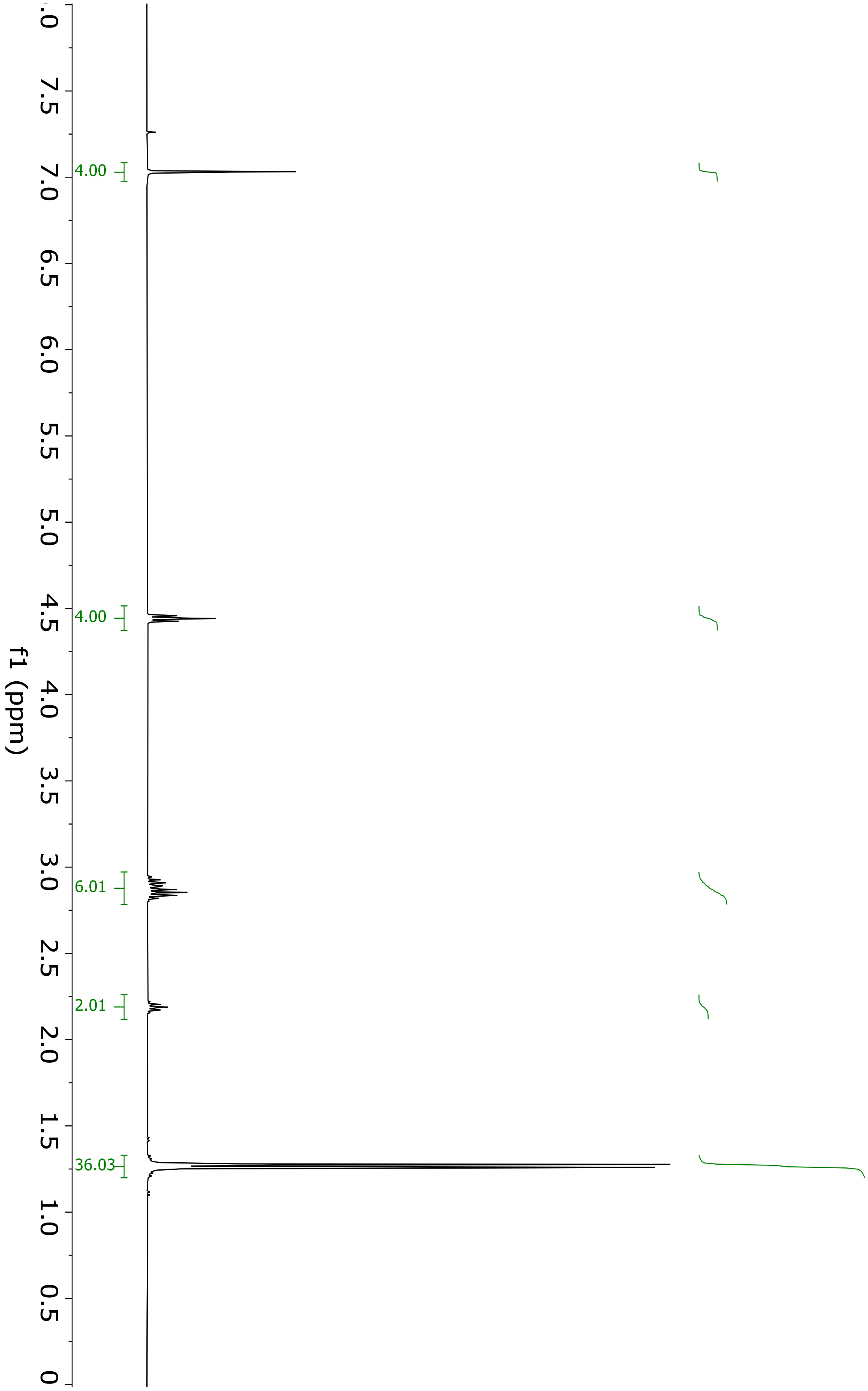

^{13}C NMR (101 MHz, $CDCl_3$) of compound **S9**. *See procedure*.

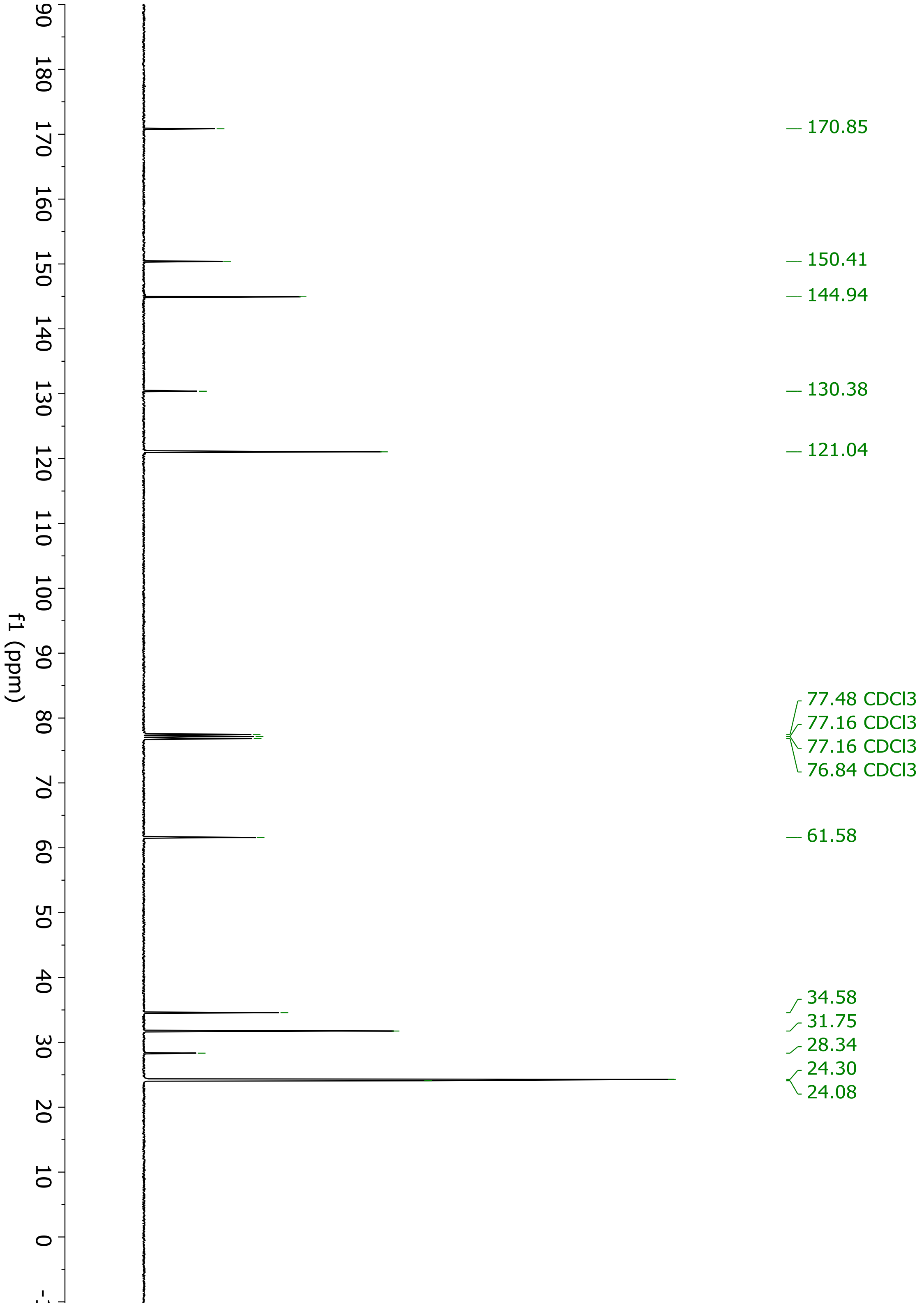

^{1}H NMR (400 MHz, $CDCl_3$) of compound **S10**. See procedure

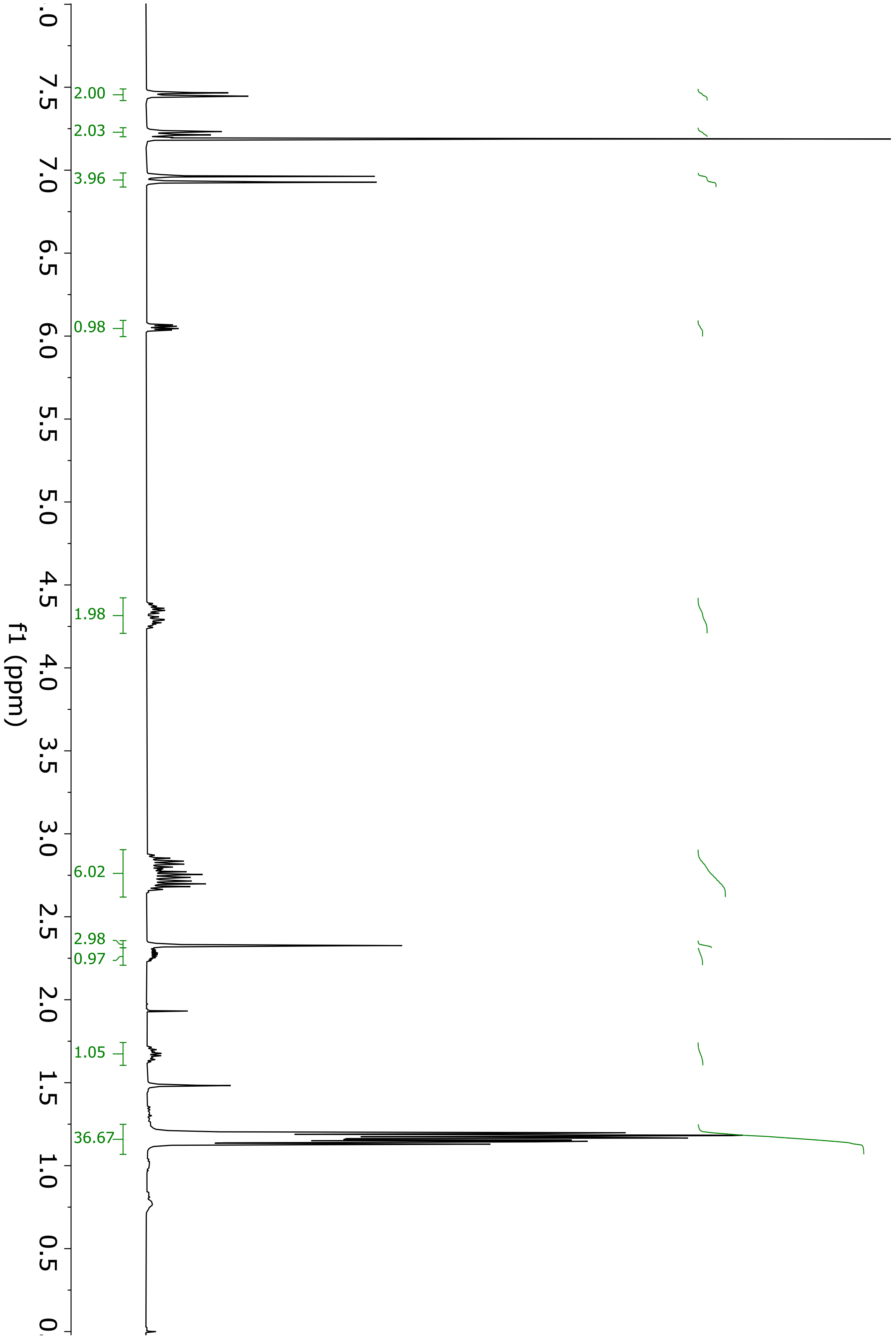

^{13}C NMR (101 MHz, $CDCl_3$) of compound **S10**. See procedure

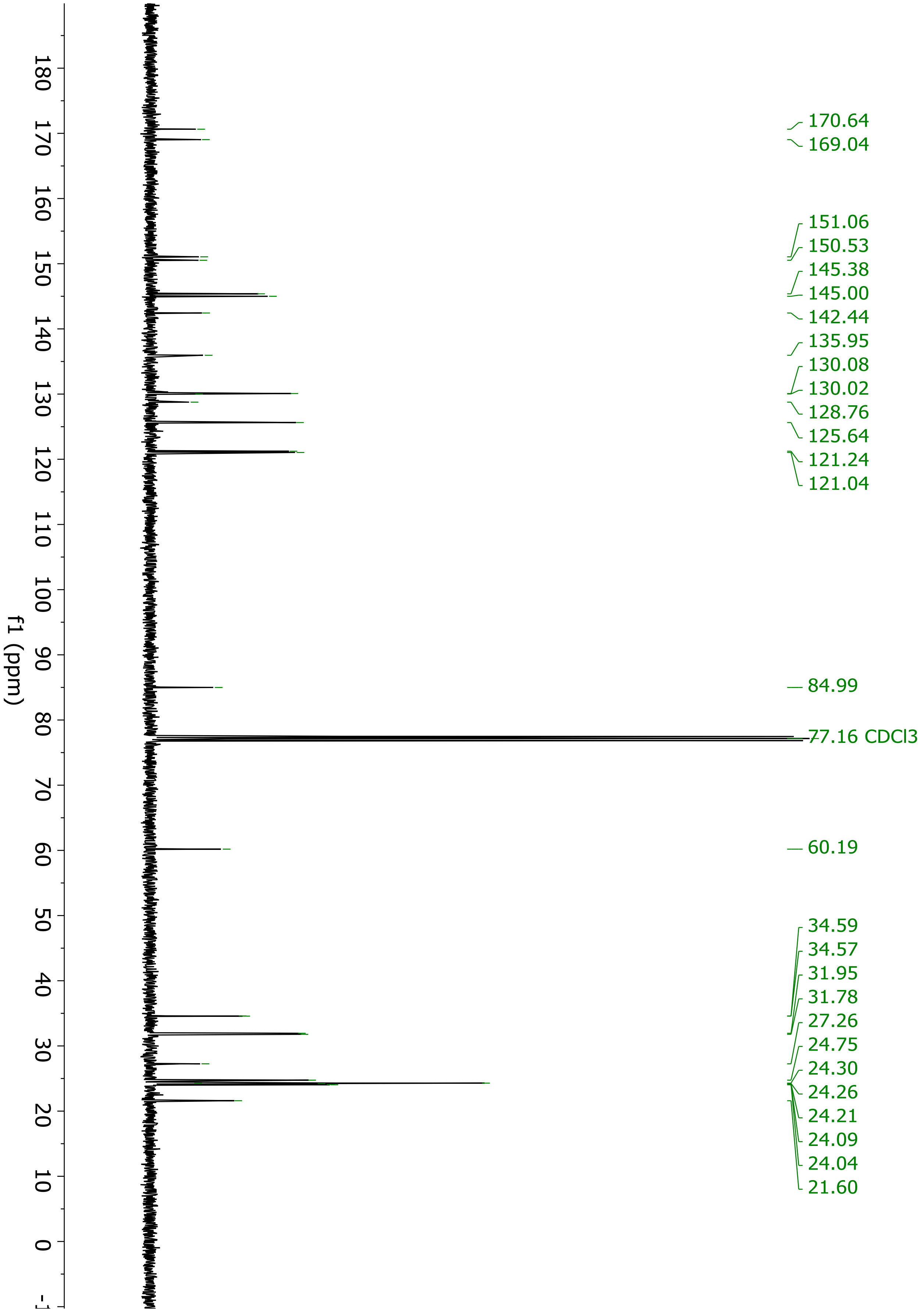

^{1}H NMR (400 MHz, $CDCl_3$) of compound **S11**. *See procedure*

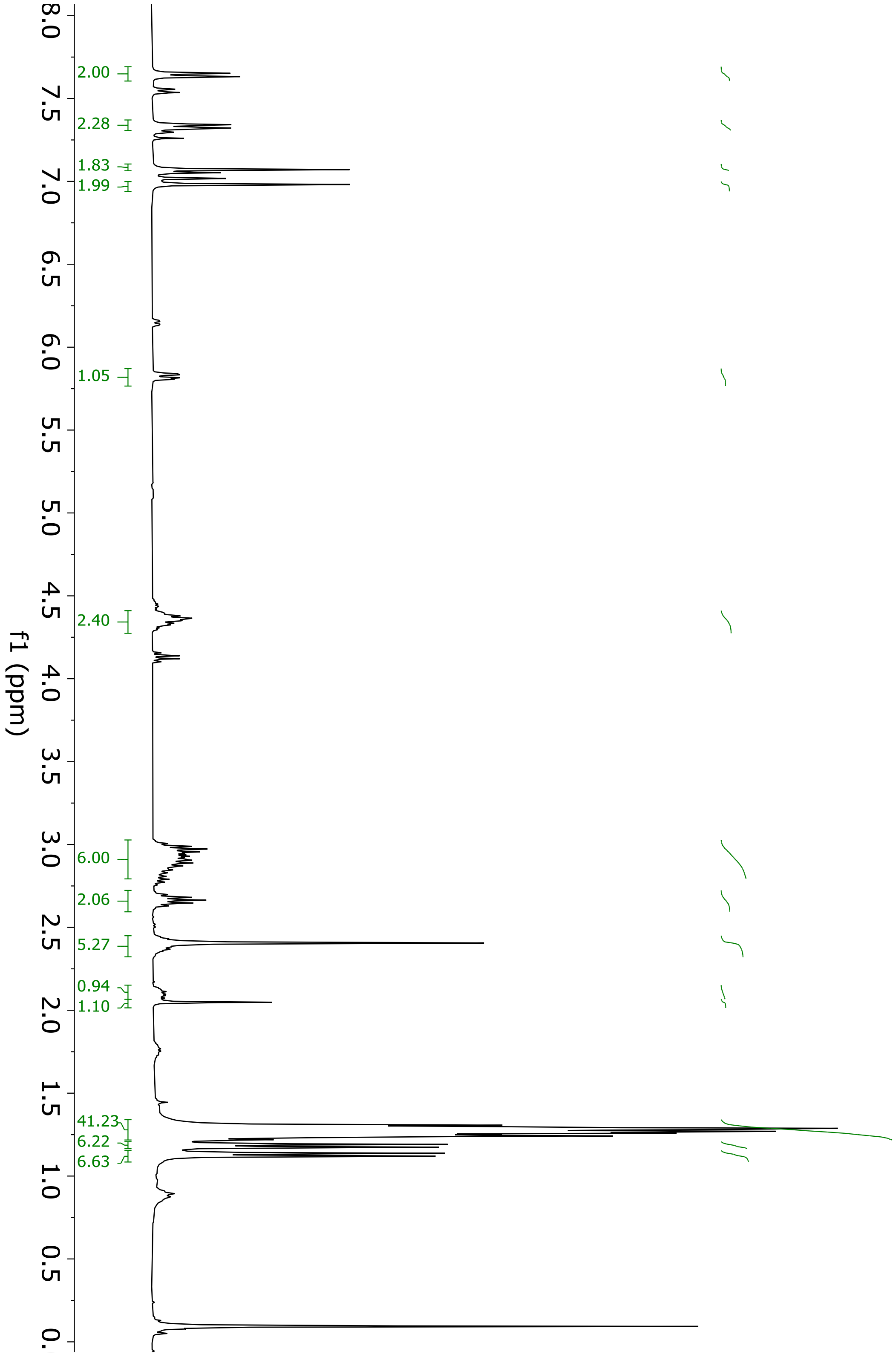

^{13}C NMR (101 MHz, $CDCl_3$) of compound **S11**. See procedure

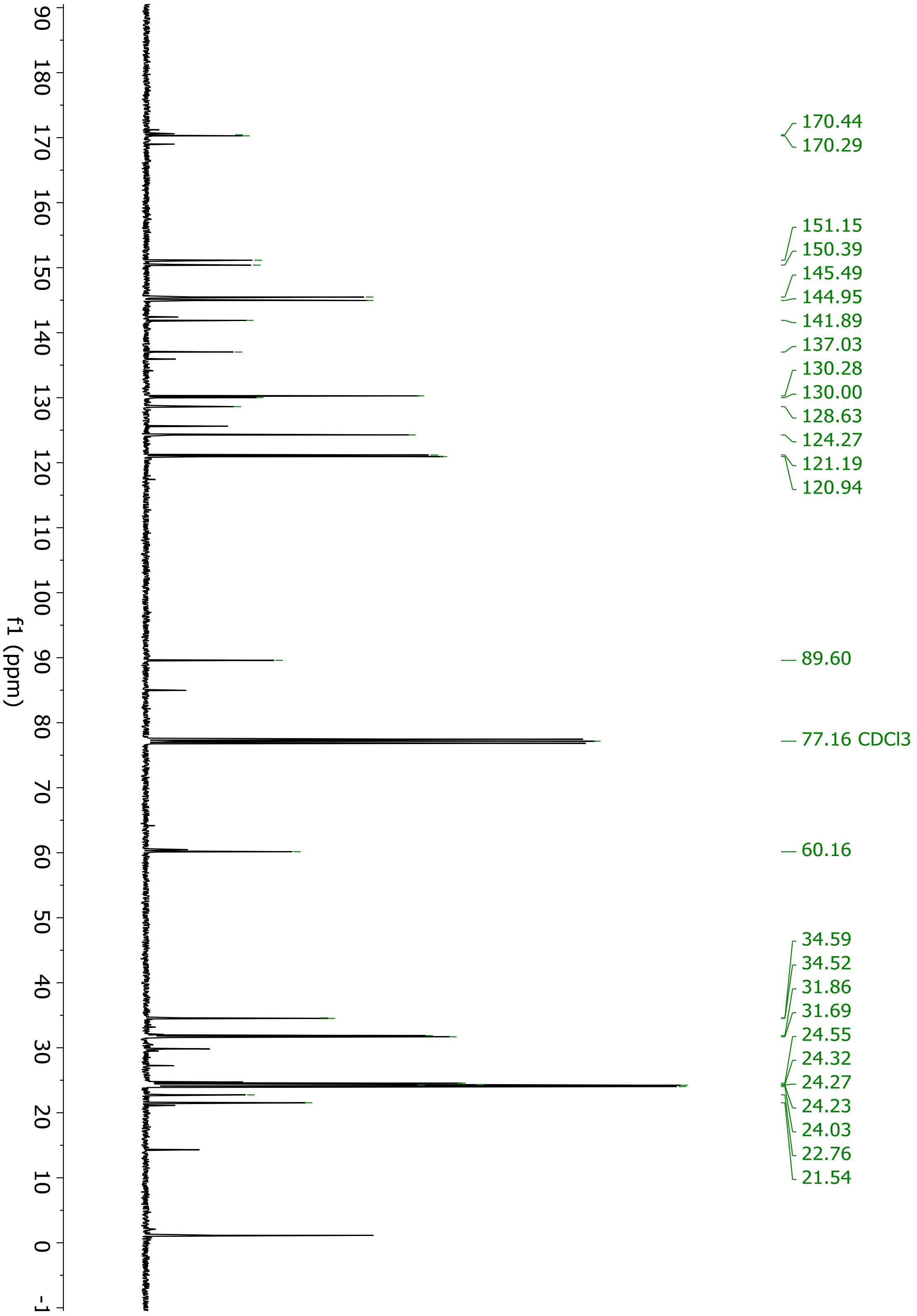

^{1}H NMR (400 MHz, $CDCl_3$) of compound **S12** (with bis TIB ester **S9** impurity). *See procedure*

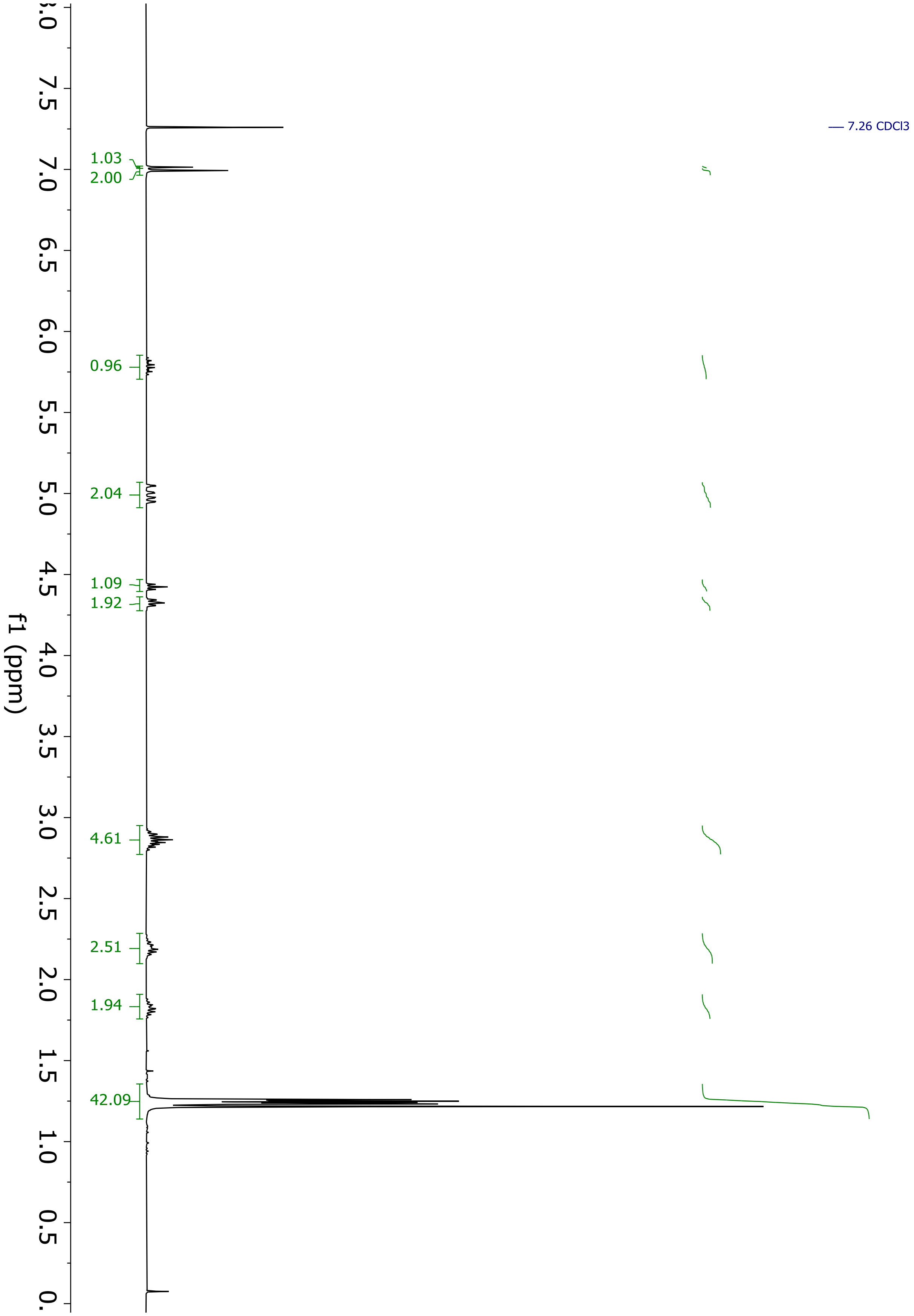

^{1}H NMR (400 MHz, $CDCl_3$) of compound **S12** (repurified for analysis)

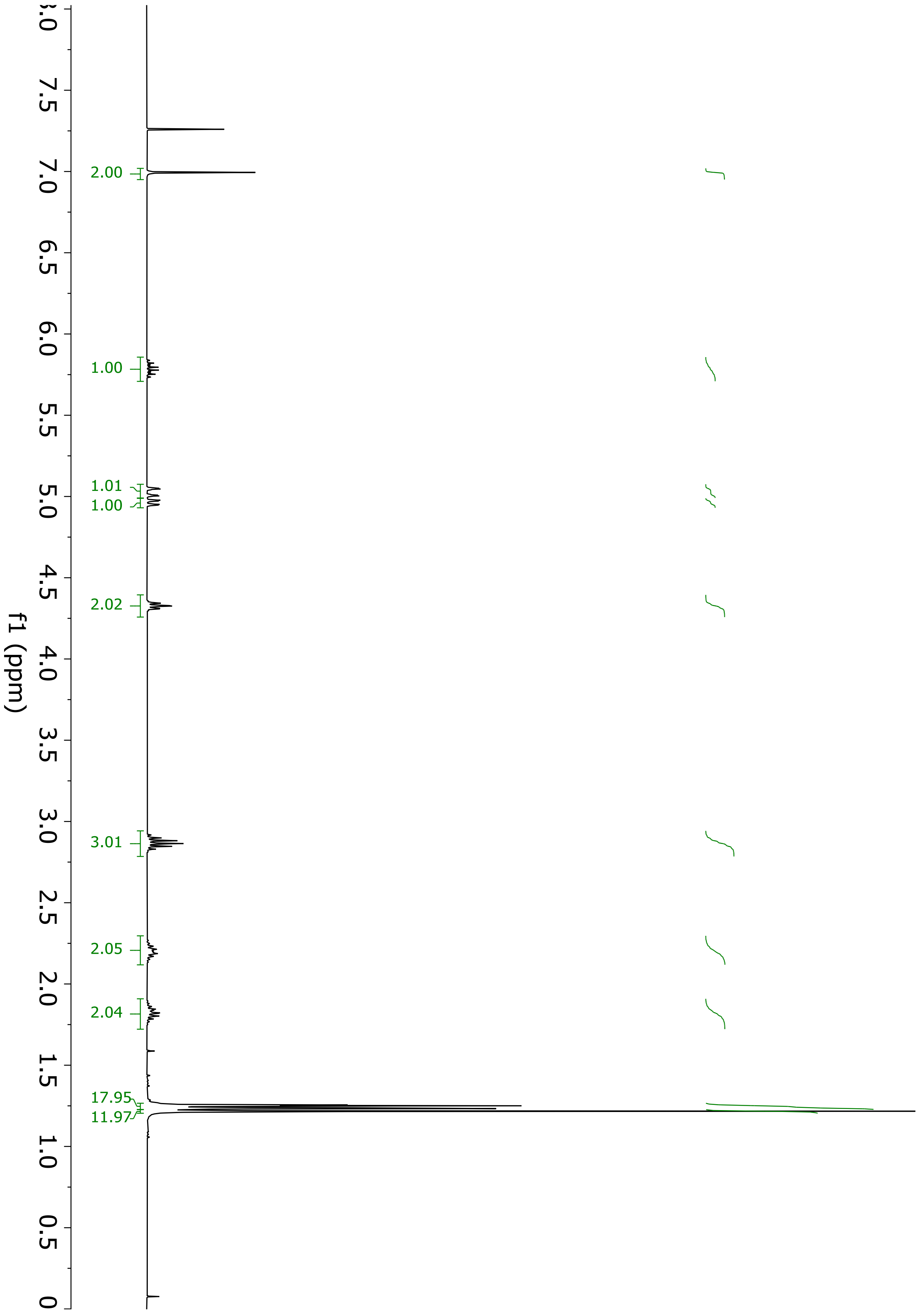

^{13}C NMR (101 MHz, $CDCl_3$) of compound **S12** (with bis TIB ester **S9** impurity). *See procedure*

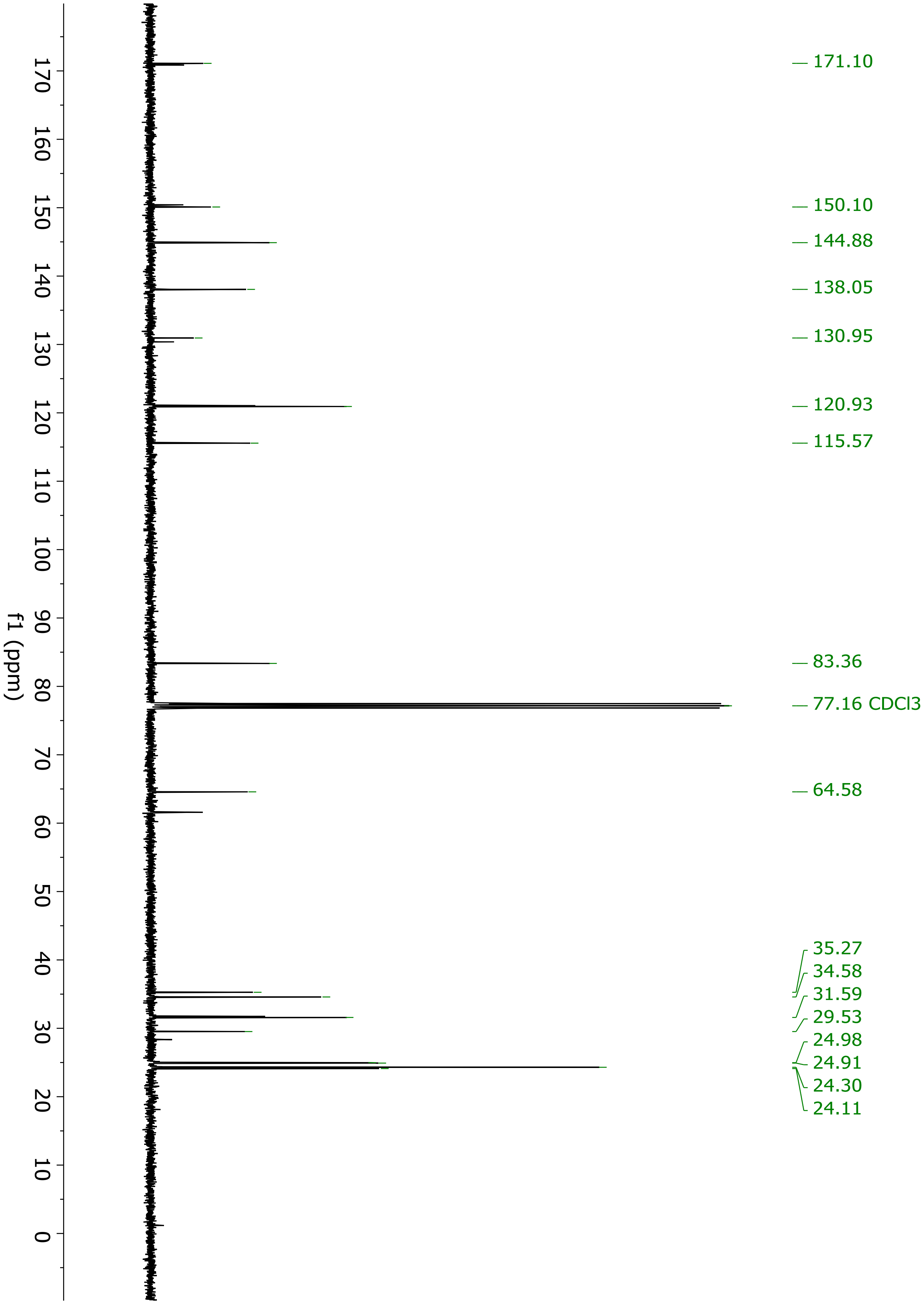

^{1}H NMR (400 MHz, $CDCl_3$) of compound **S13**. *See procedure*.

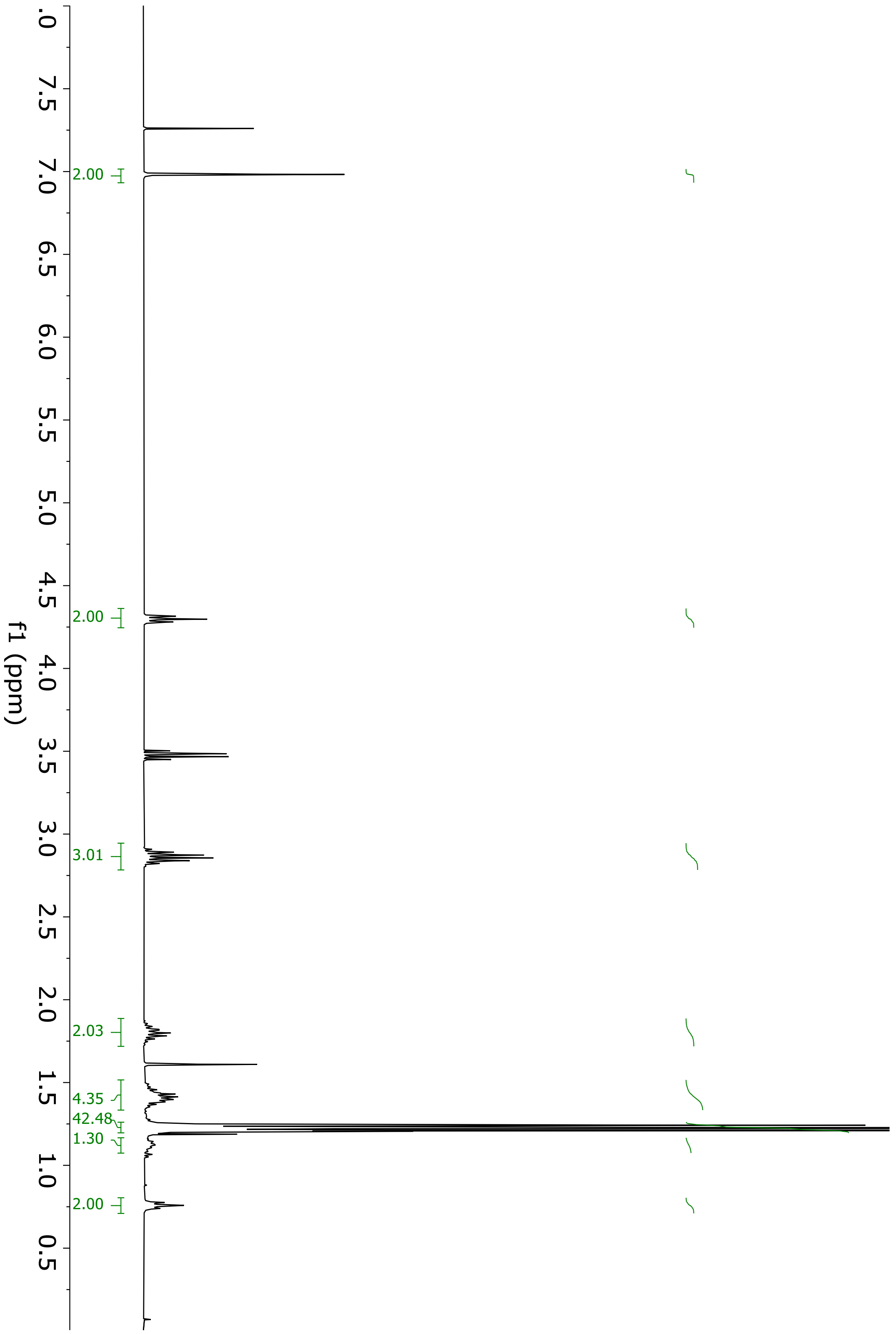

^{13}C NMR (101 MHz, $CDCl_3$) of compound **S13**. *See procedure*.

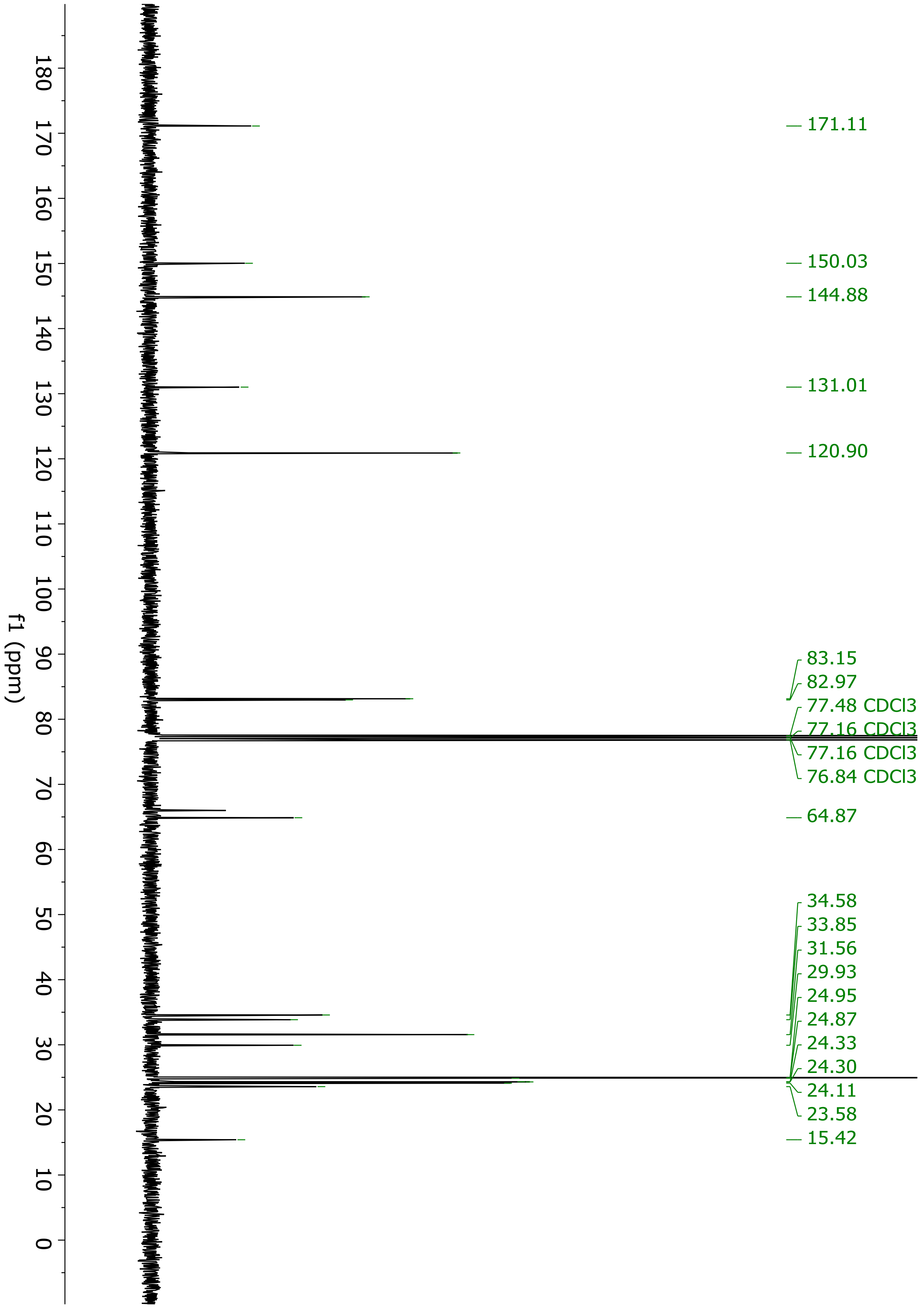

^{1}H NMR (400 MHz, $CDCl_3$) of compound **S14**. *See procedure*.

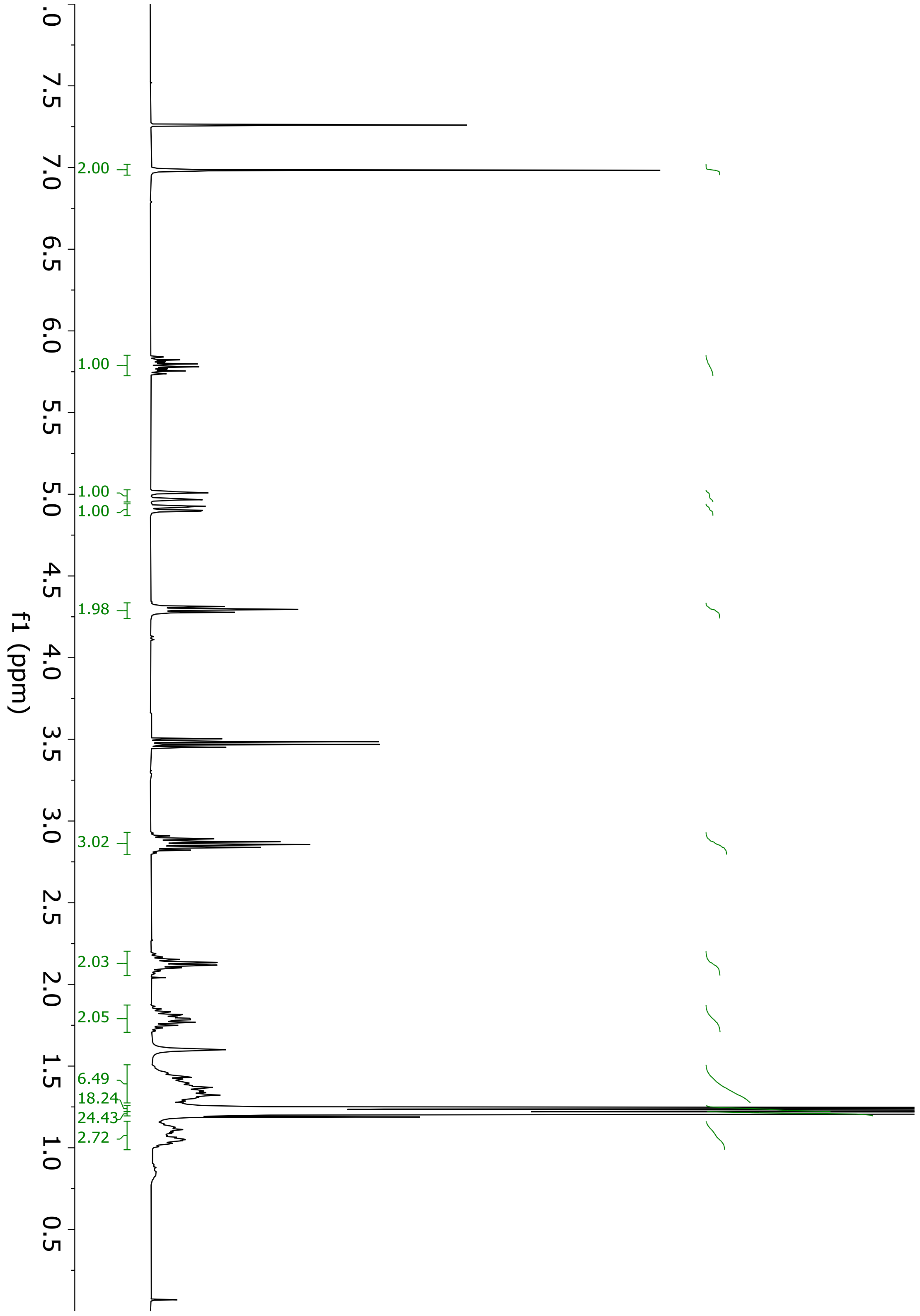

^{13}C NMR (101 MHz, $CDCl_3$) of compound **S14**. *See procedure*.

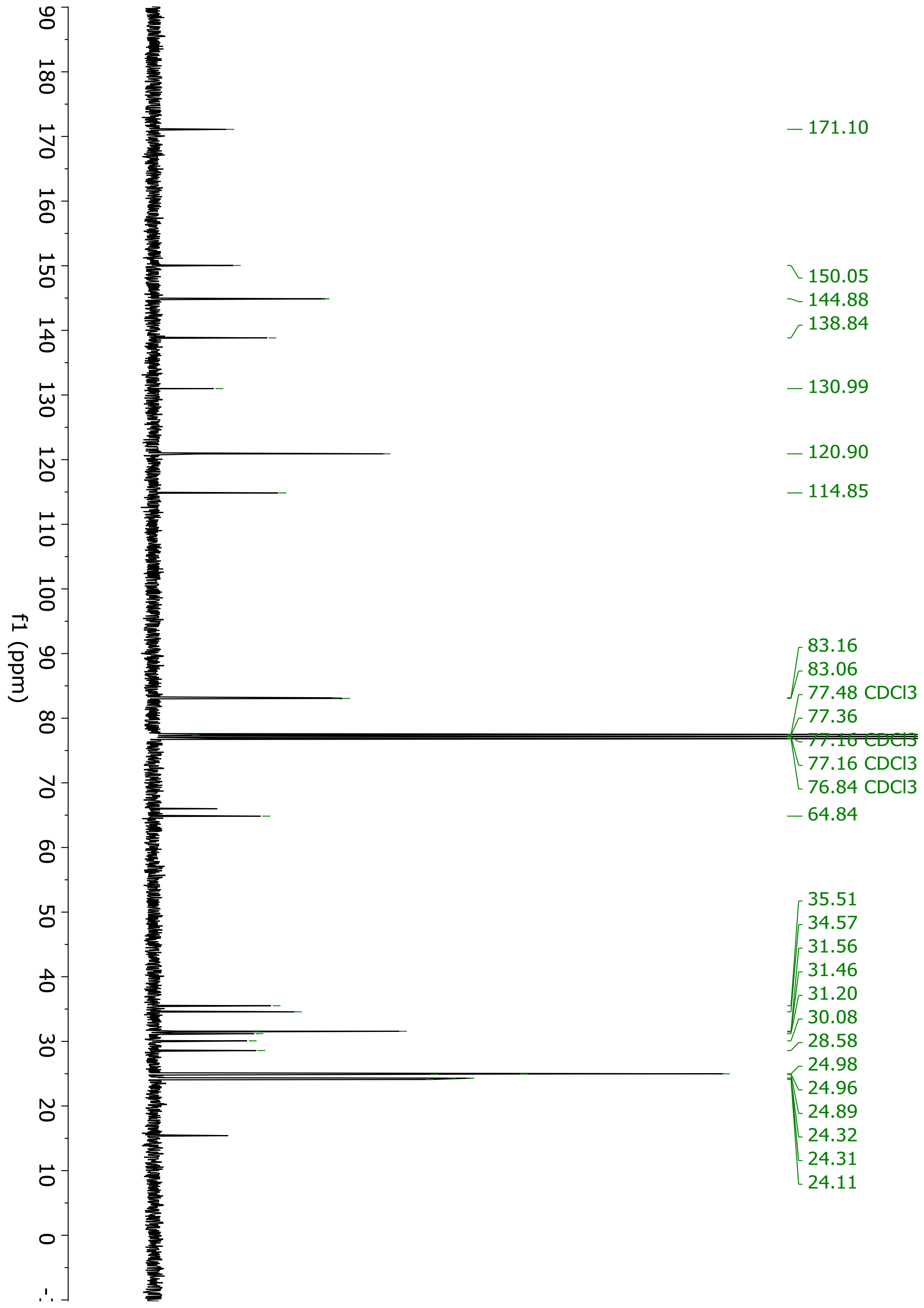

^{1}H NMR (400 MHz, $CDCl_3$) of compound **S15**. *See procedure*.

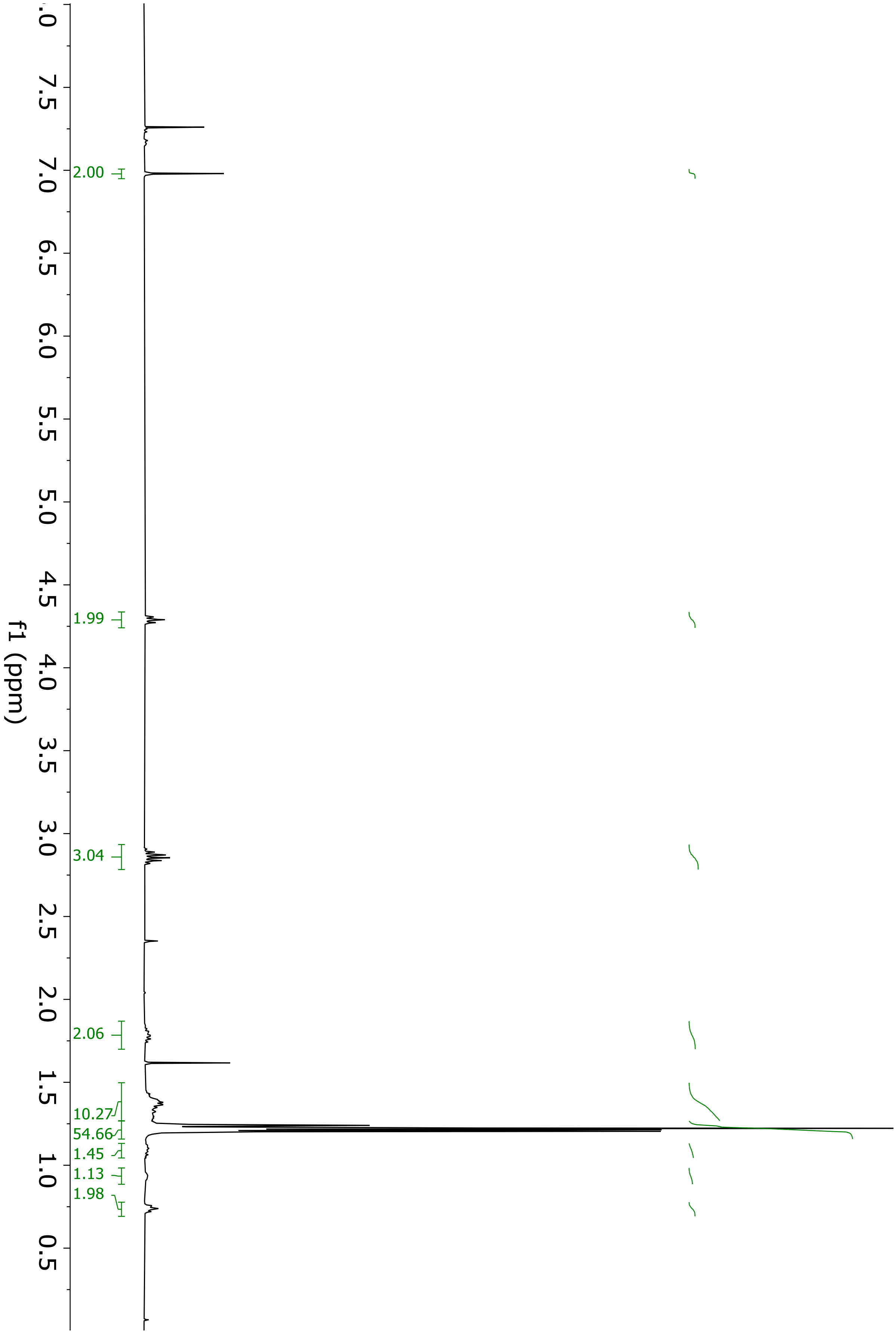

^{13}C NMR (101 MHz, $CDCl_3$) of compound **S15**. *See procedure*.

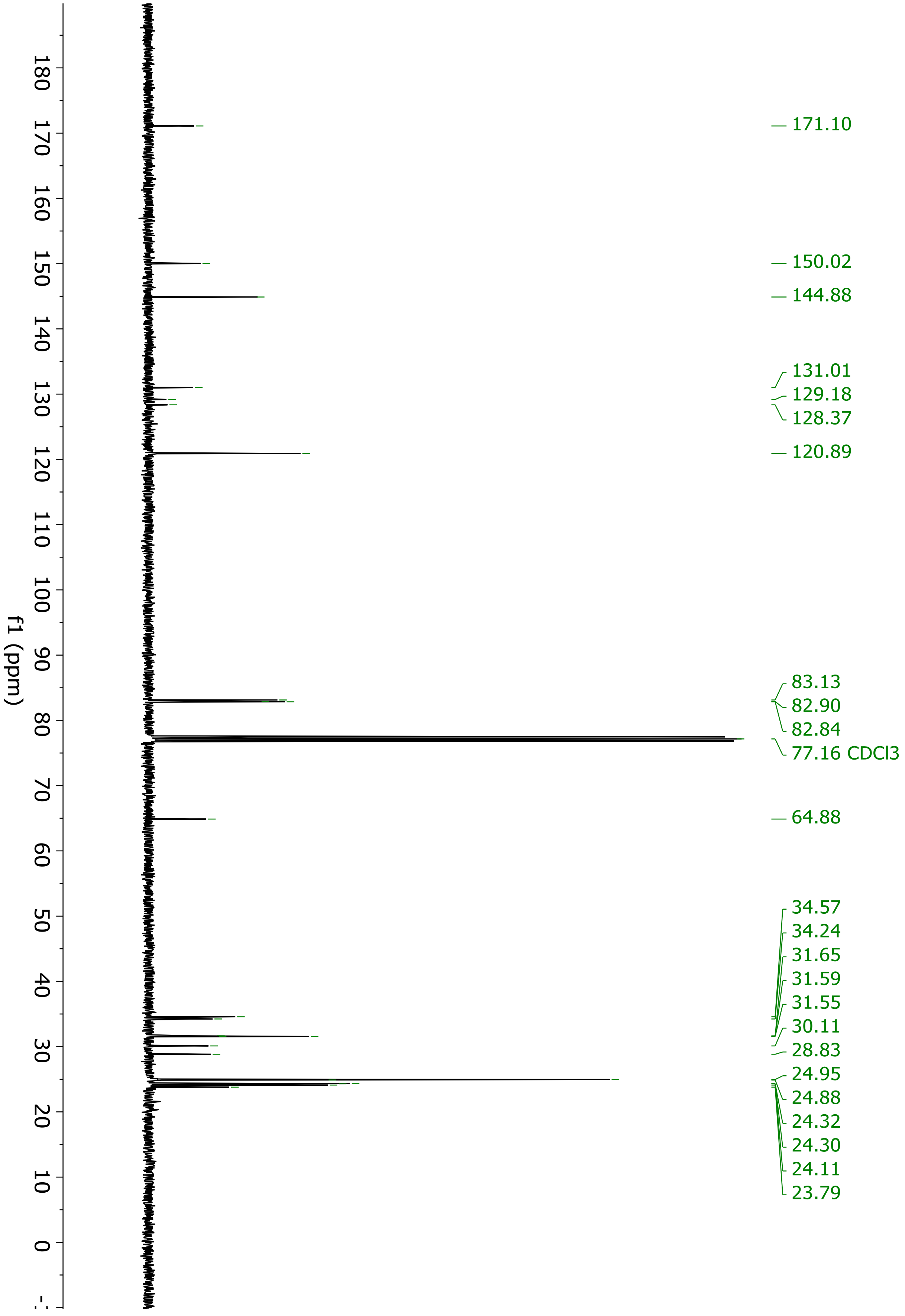

^{1}H NMR (400 MHz, $CDCl_3$) of compound **S16**. See procedure.

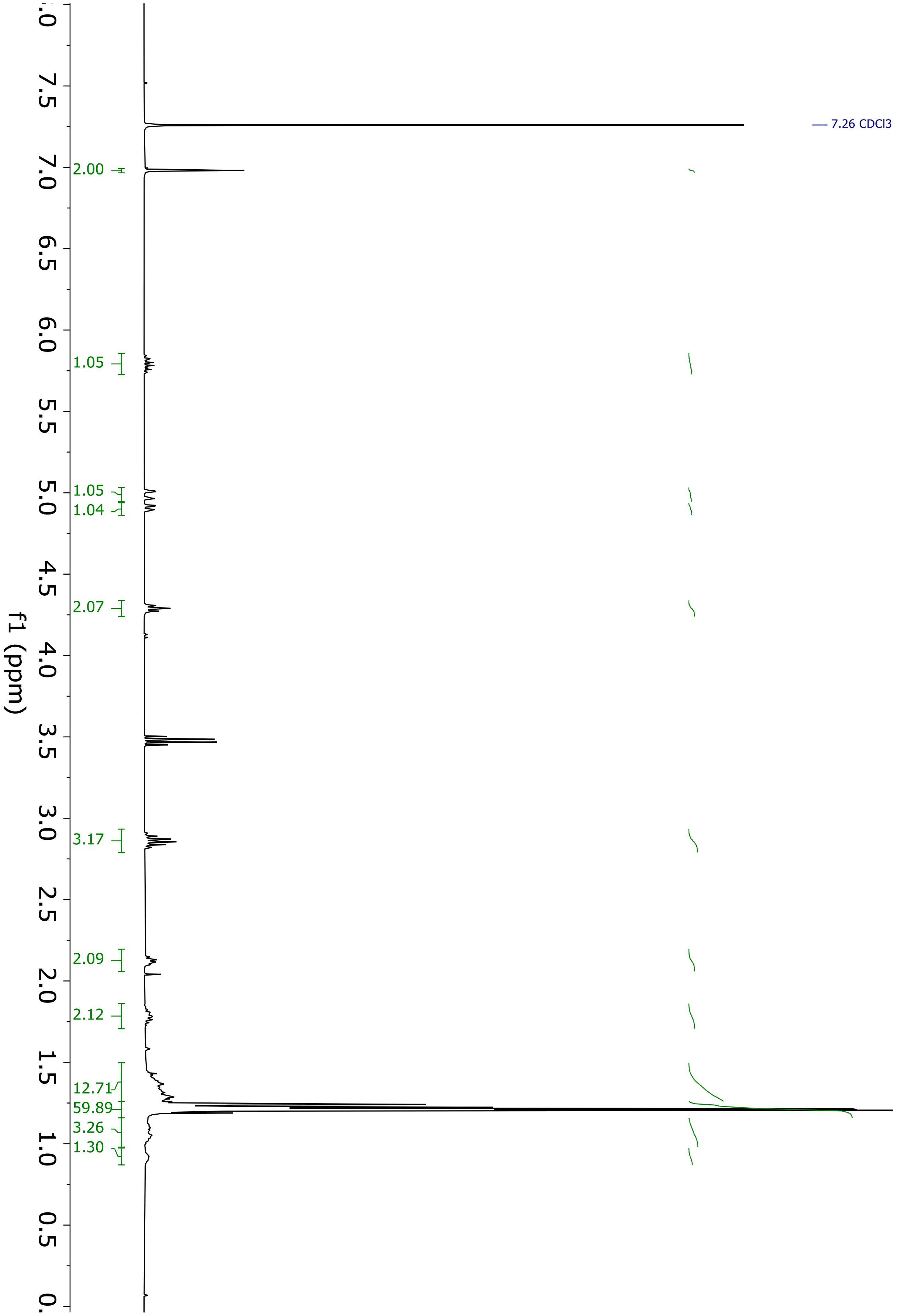

^{13}C NMR (101 MHz, $CDCl_3$) of compound **S16**. See procedure

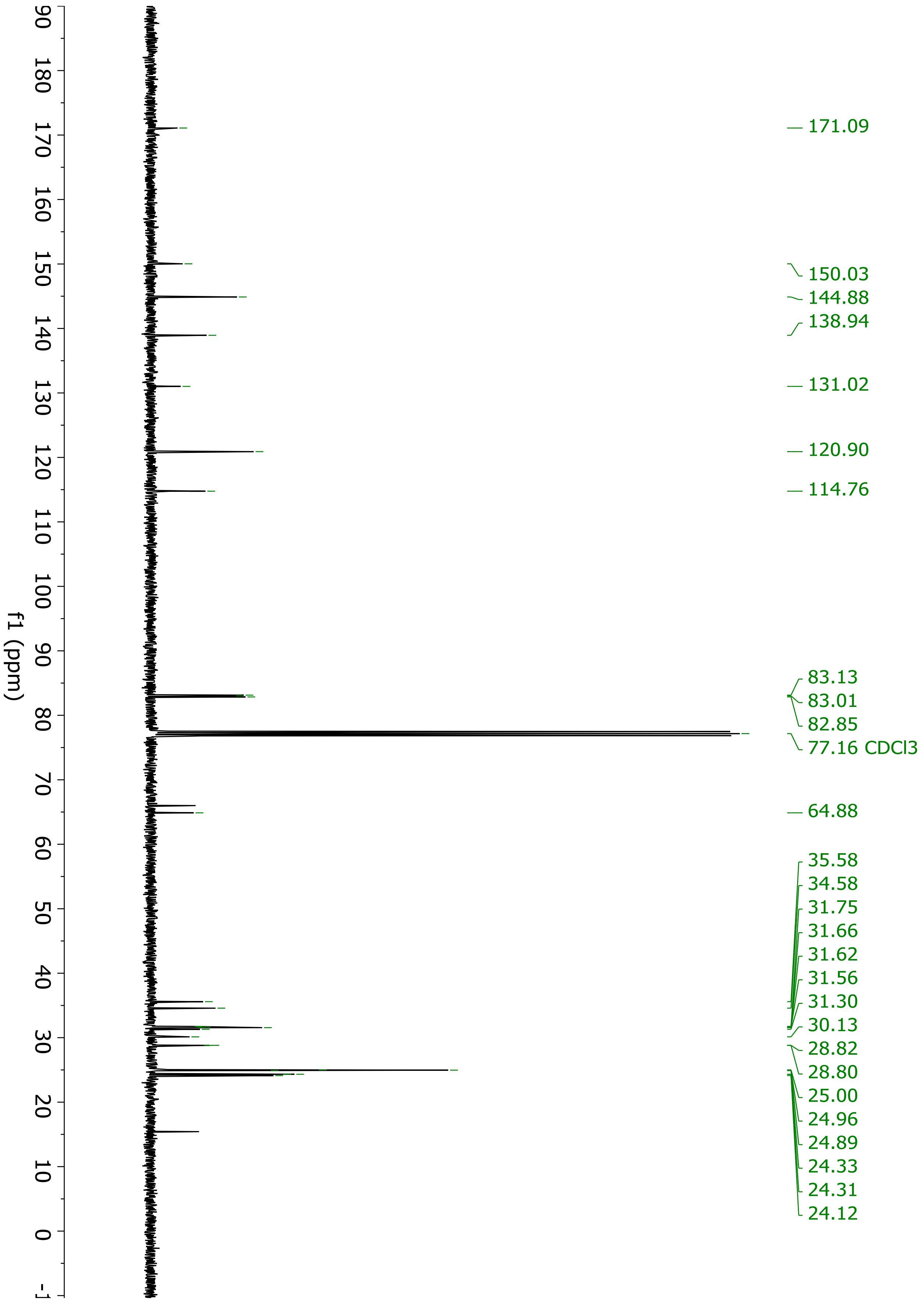

^{1}H NMR (400 MHz, $CDCl_3$) of compound **16.** *See procedure*.

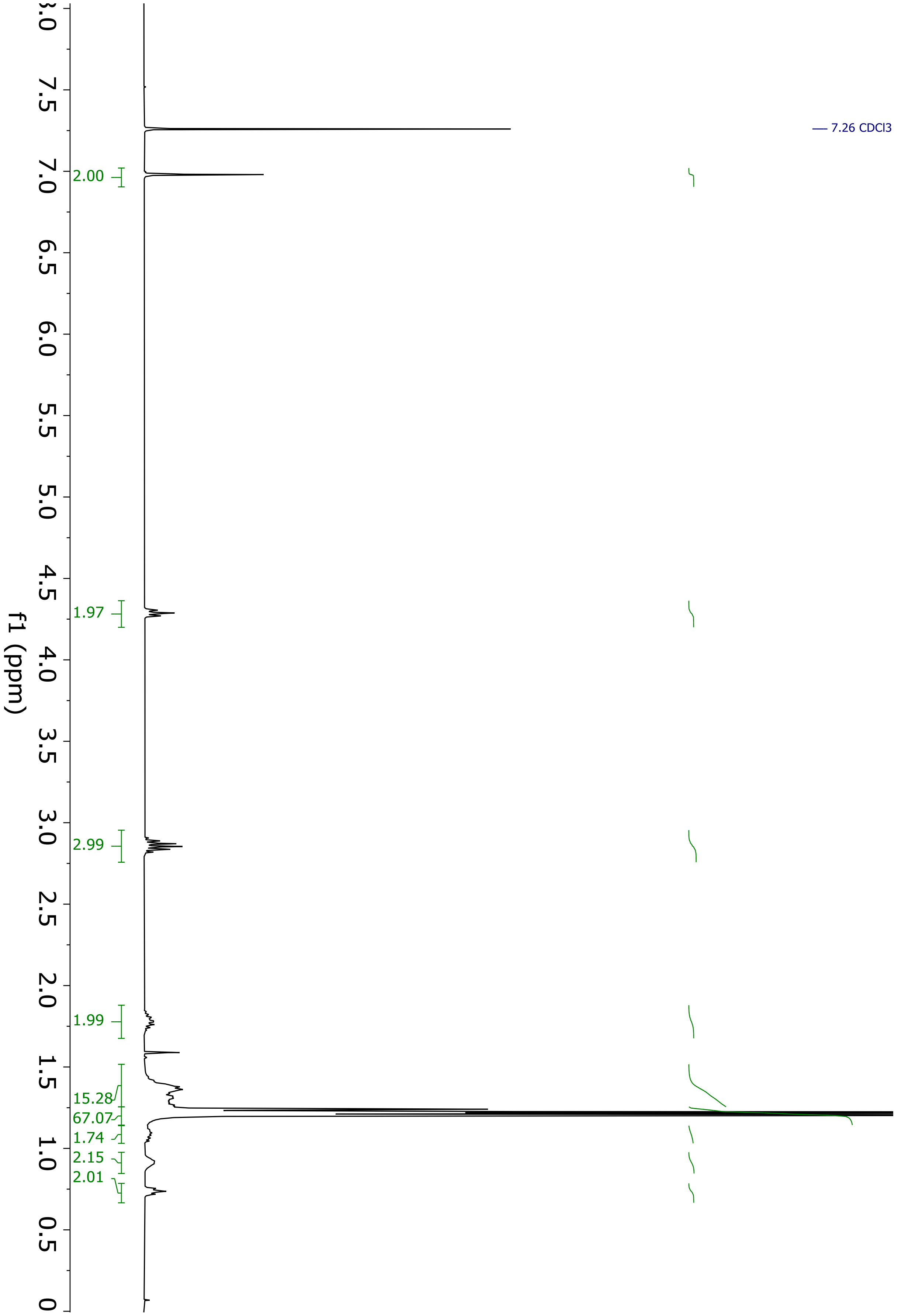

^{13}C NMR (101 MHz, $CDCl_3$) of compound **16**. *See procedure*.

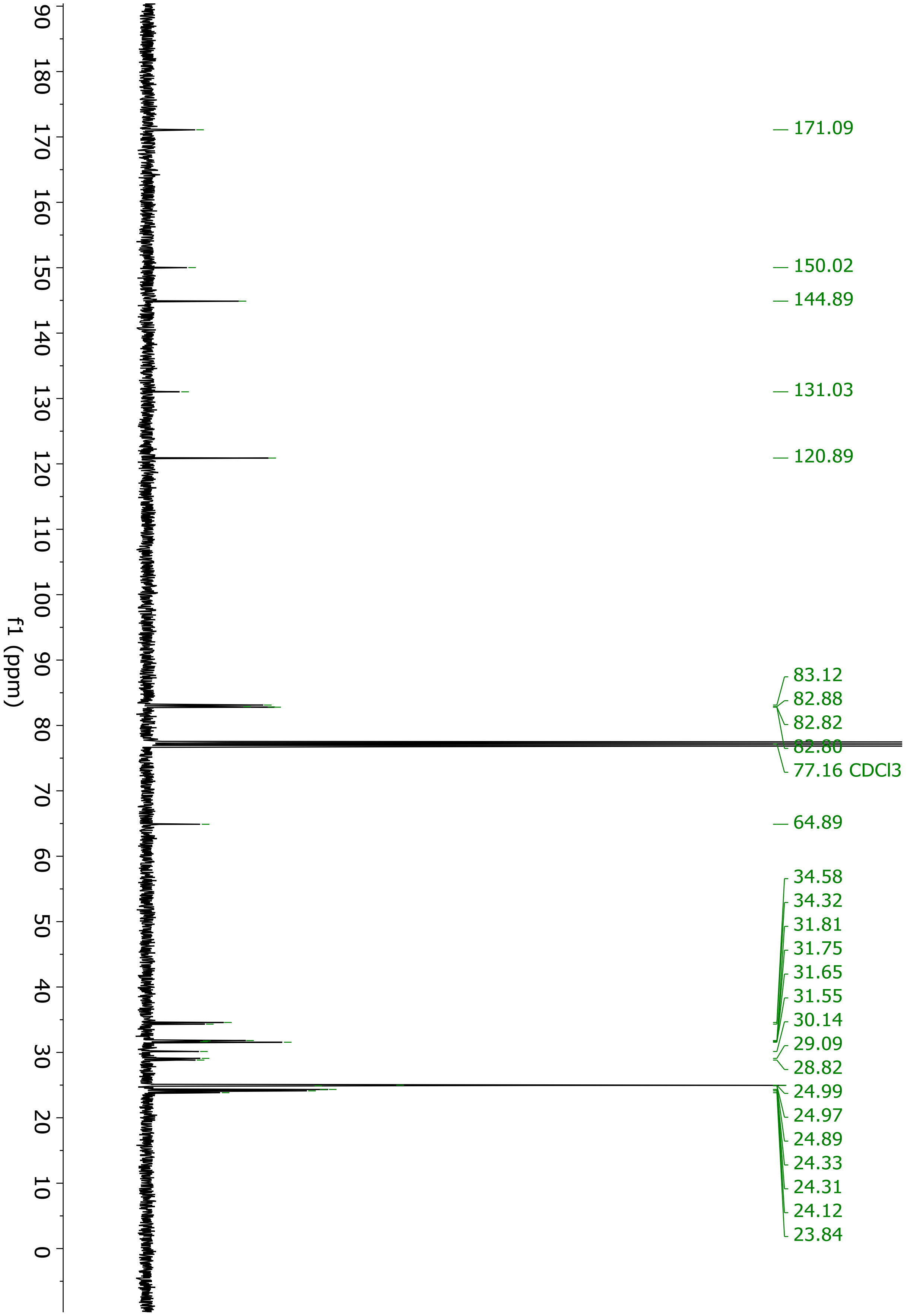

^{1}H NMR (400 MHz, $CDCl_3$) of compound **S17.** See procedure.

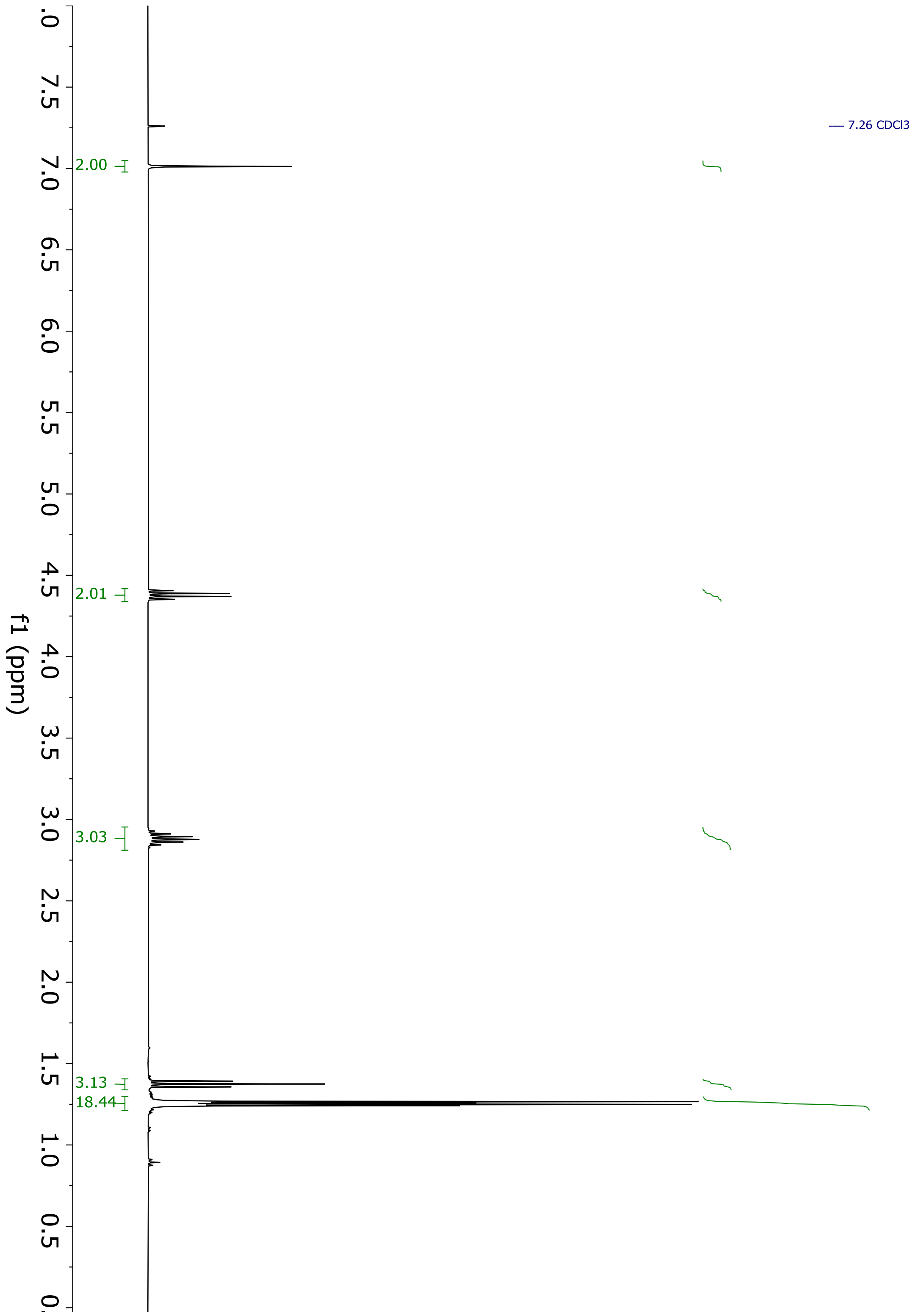

^{13}C NMR (101 MHz, $CDCl_3$) of compound **S17**. *See procedure*.

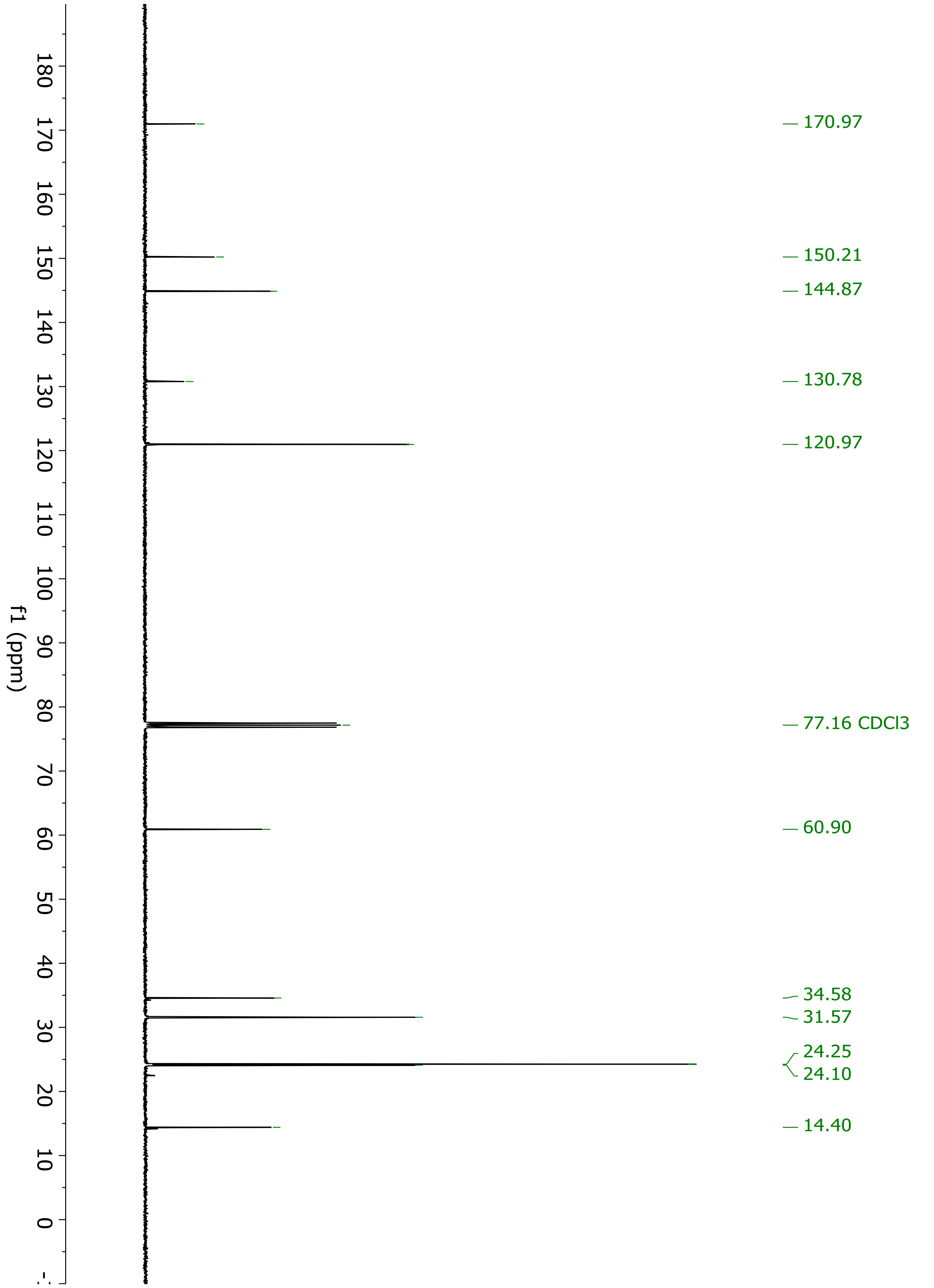

^{1}H NMR (400 MHz, $CDCl_3$) of compound **S18.** *See procedure*.

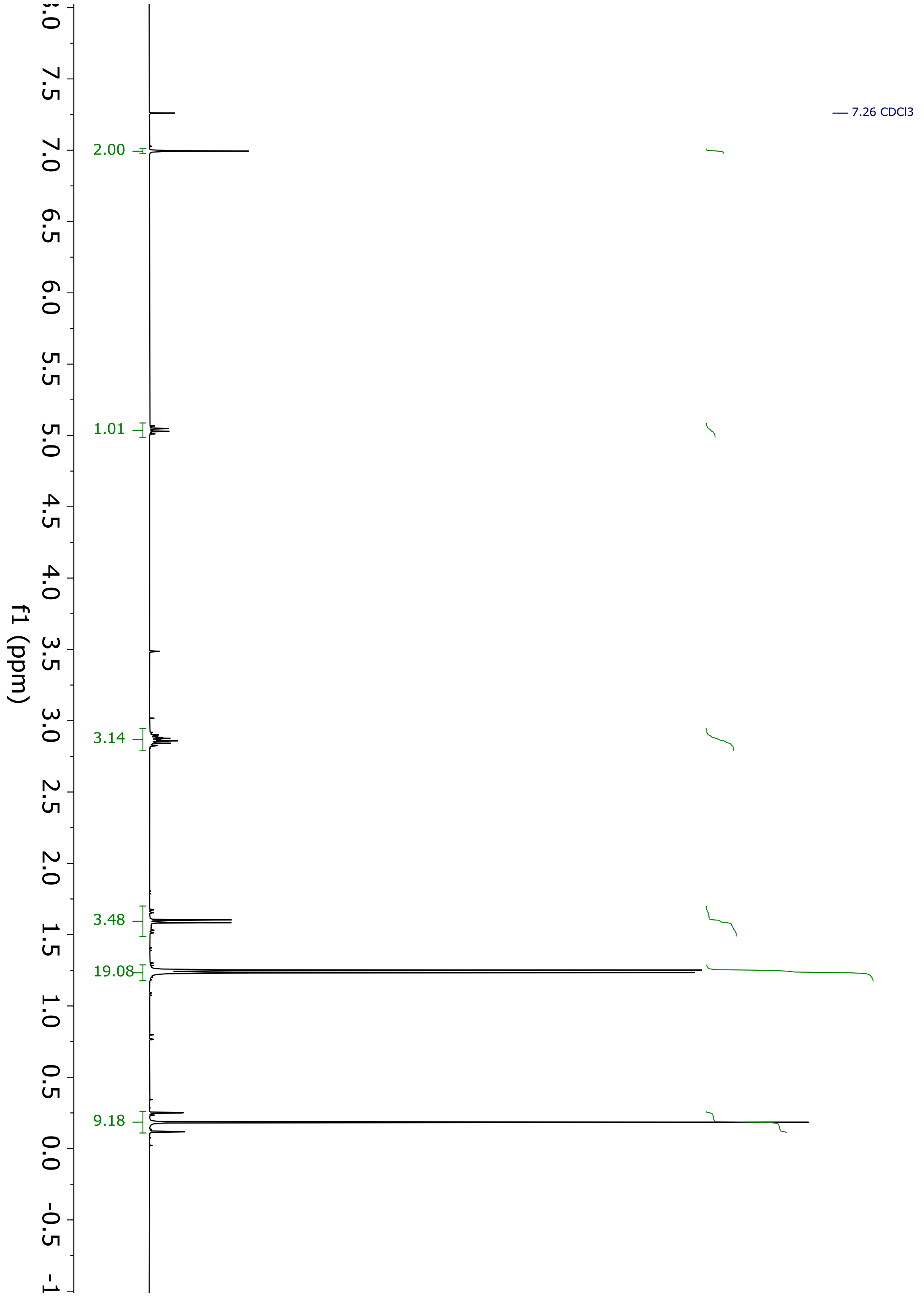

^{13}C NMR (101 MHz, $CDCl_3$) of compound **S18**. *See procedure*.

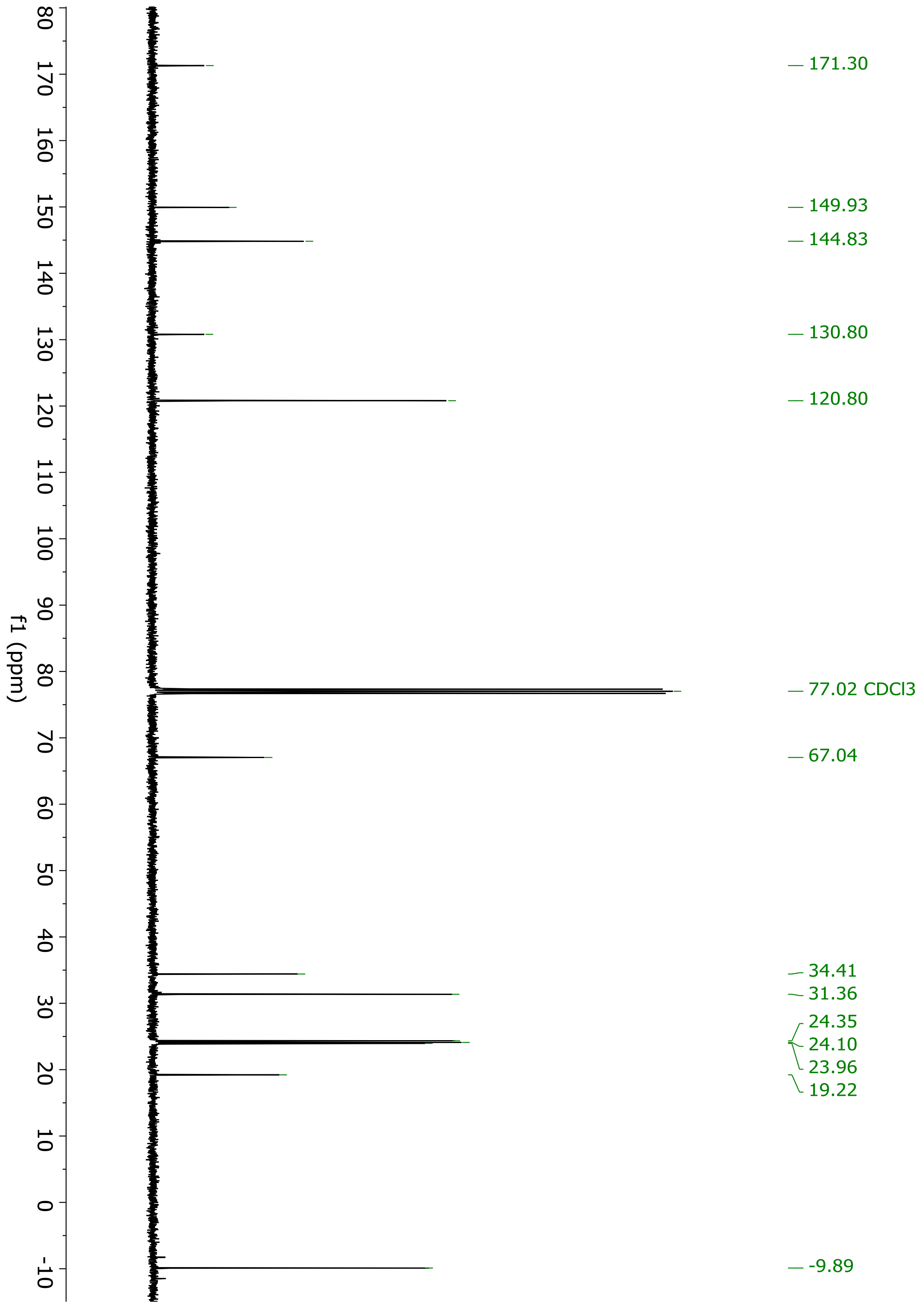

^{1}H NMR (400 MHz, $CDCl_3$) of compound **20.** *See procedure*.

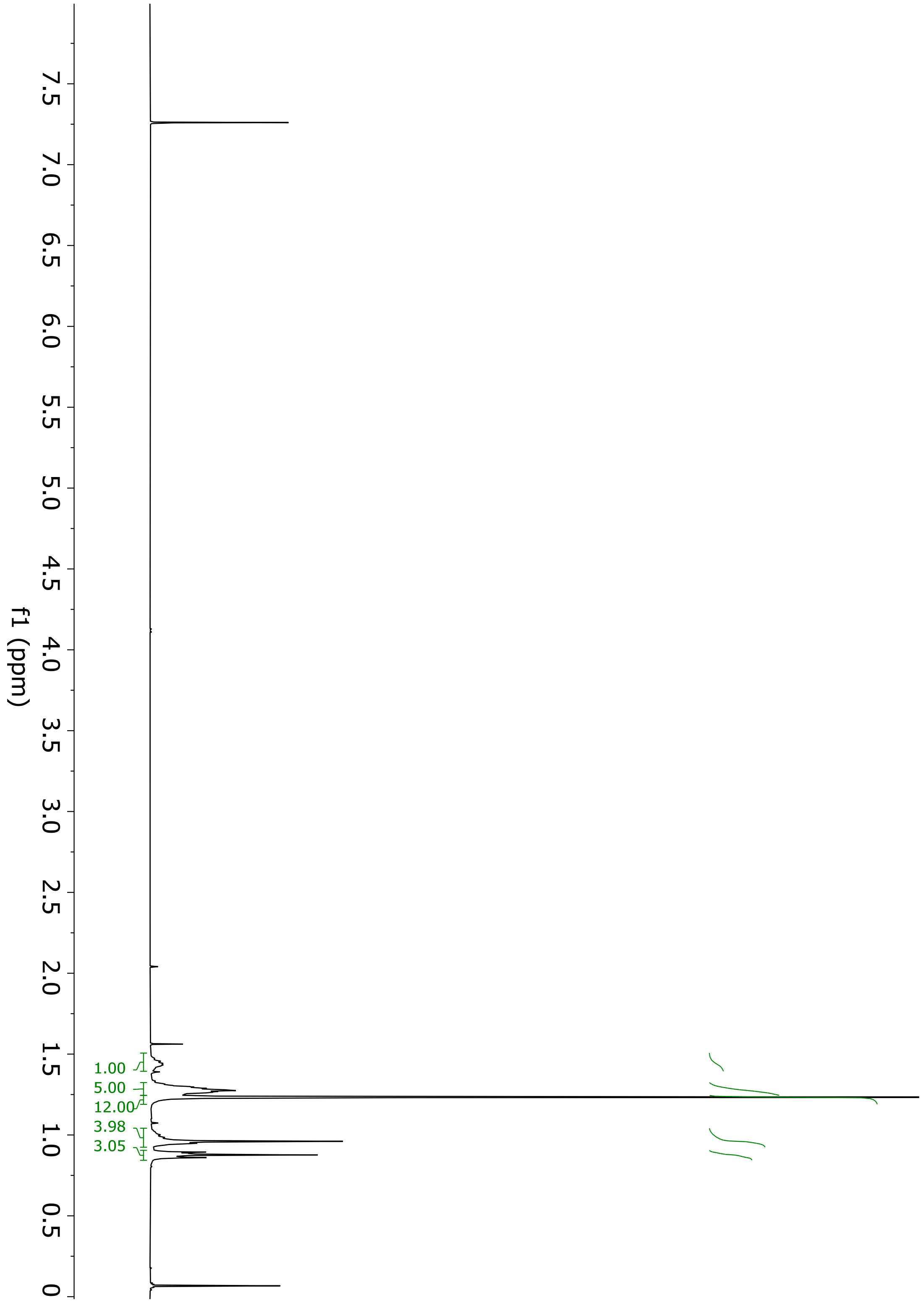

^{13}C NMR (101 MHz, $CDCl_3$) of compound **20**. *See procedure*

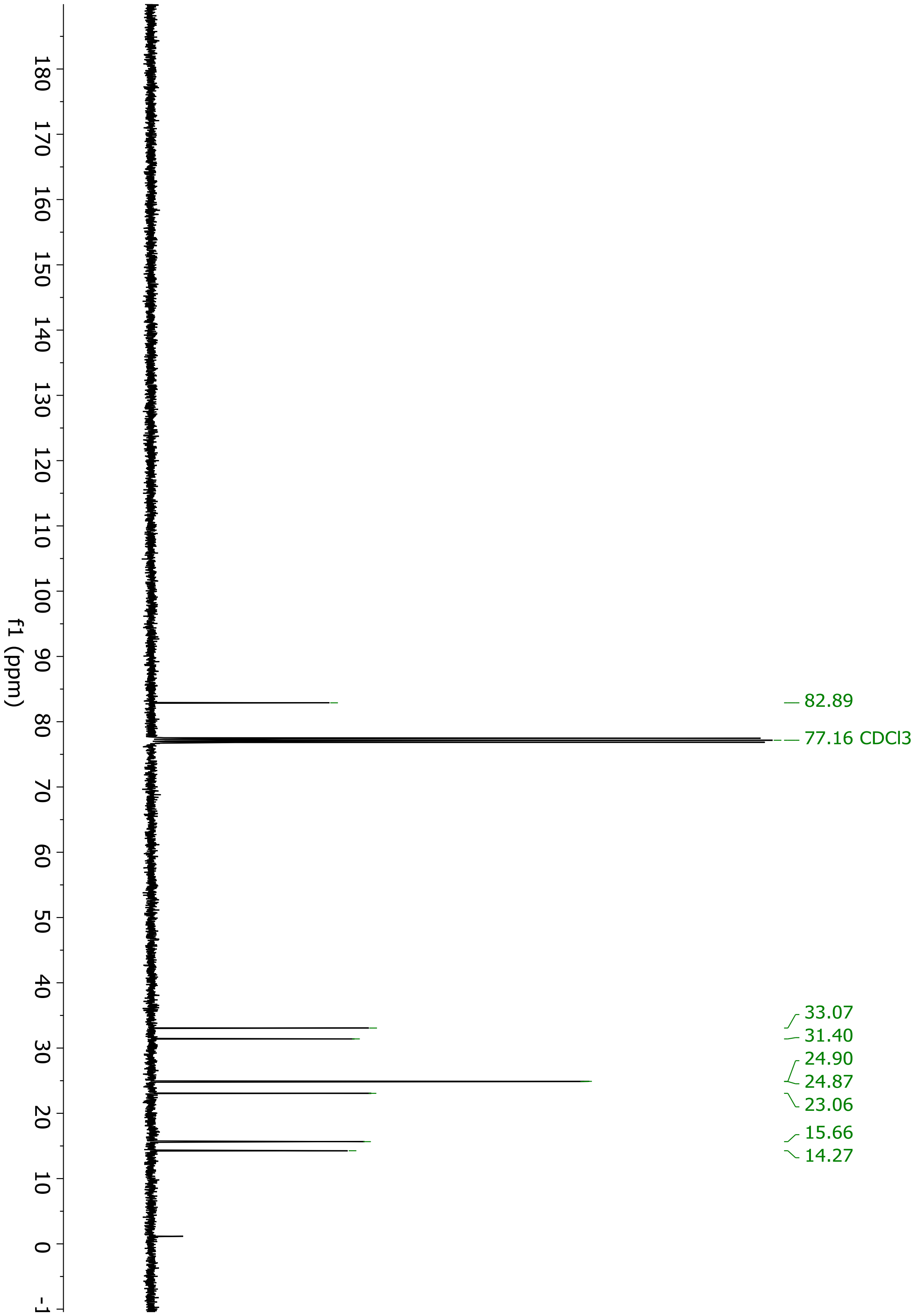

^{1}H NMR (600 MHz, $CDCl_3$) of compound **S22**. *See procedure*.

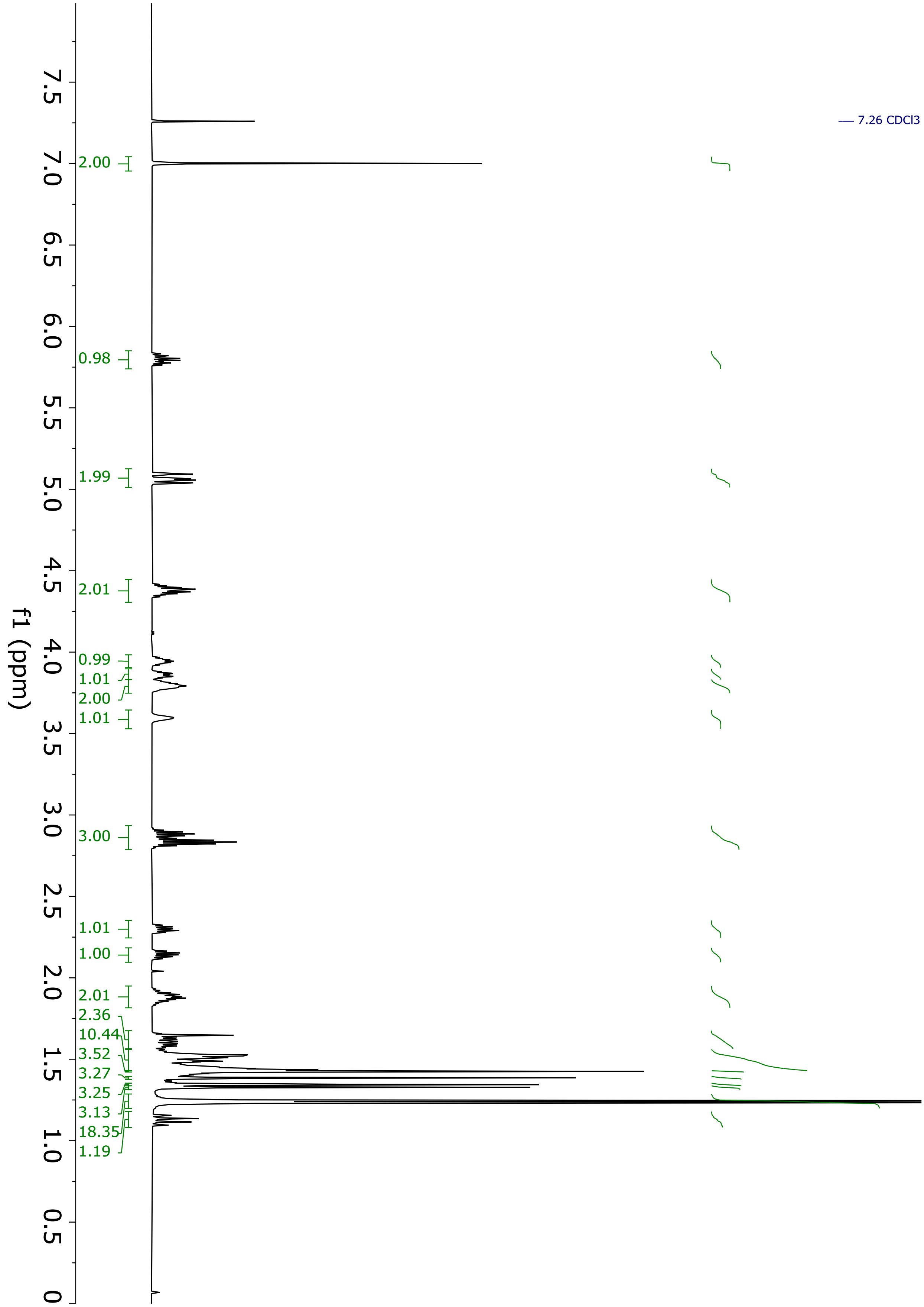

^{13}C NMR (151 MHz, $CDCl_3$) of compound **S22**. <u>See procedure</u>.

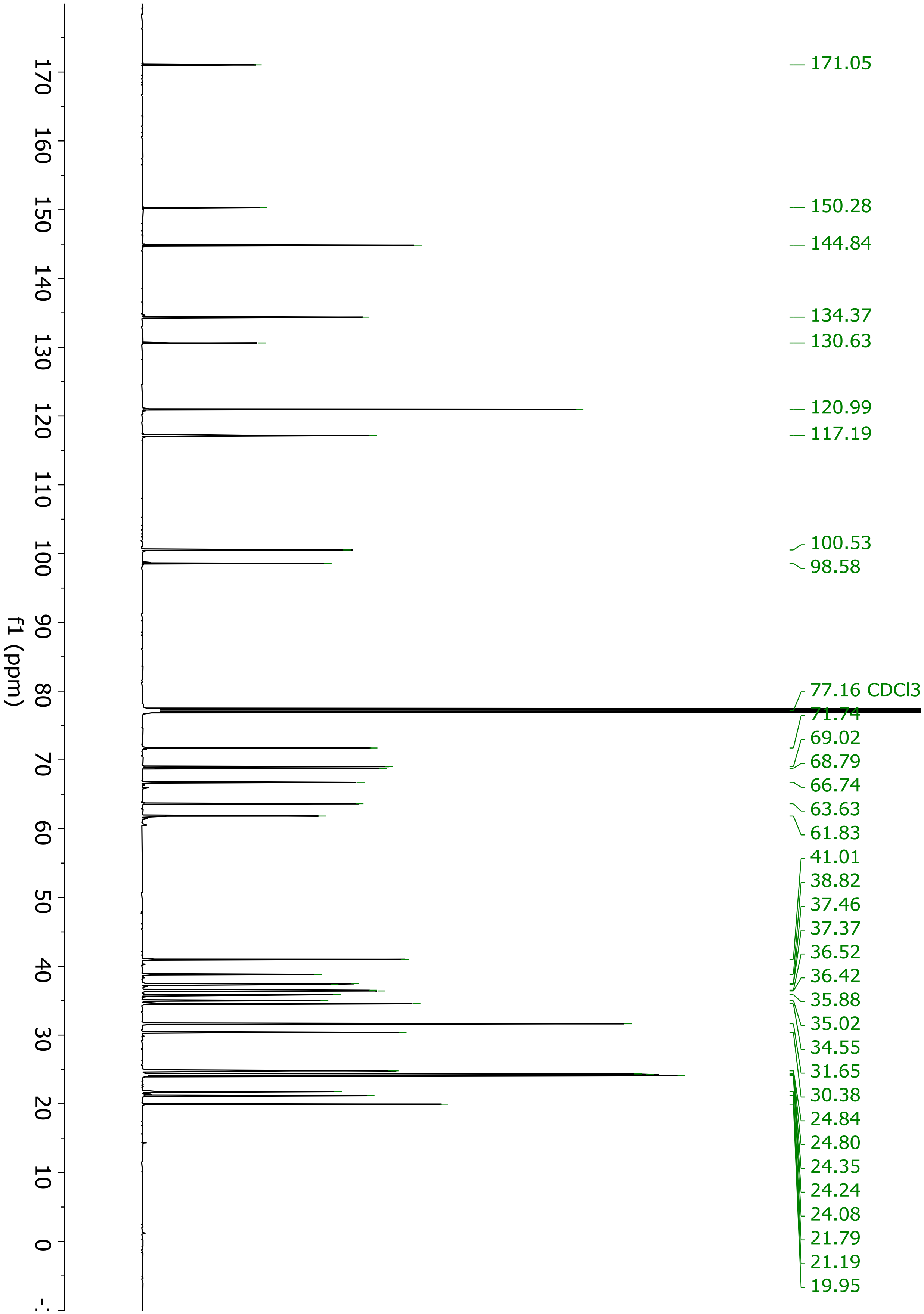

^{1}H NMR (500 MHz, $CDCl_3$) of compound **S23**. *See procedure*.

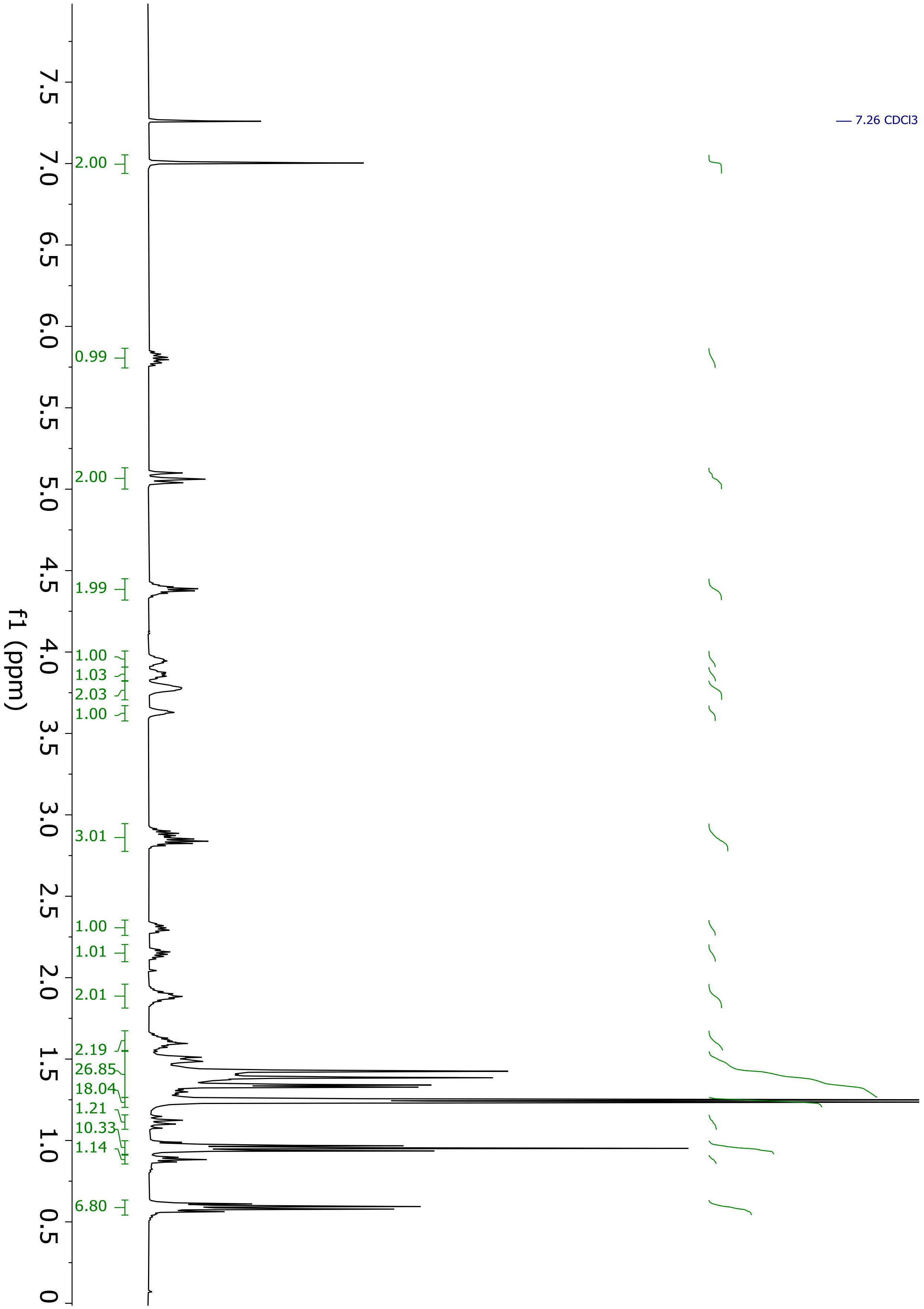

^{13}C NMR (126 MHz, $CDCl_3$) of compound **S23**. [See procedure.](#)

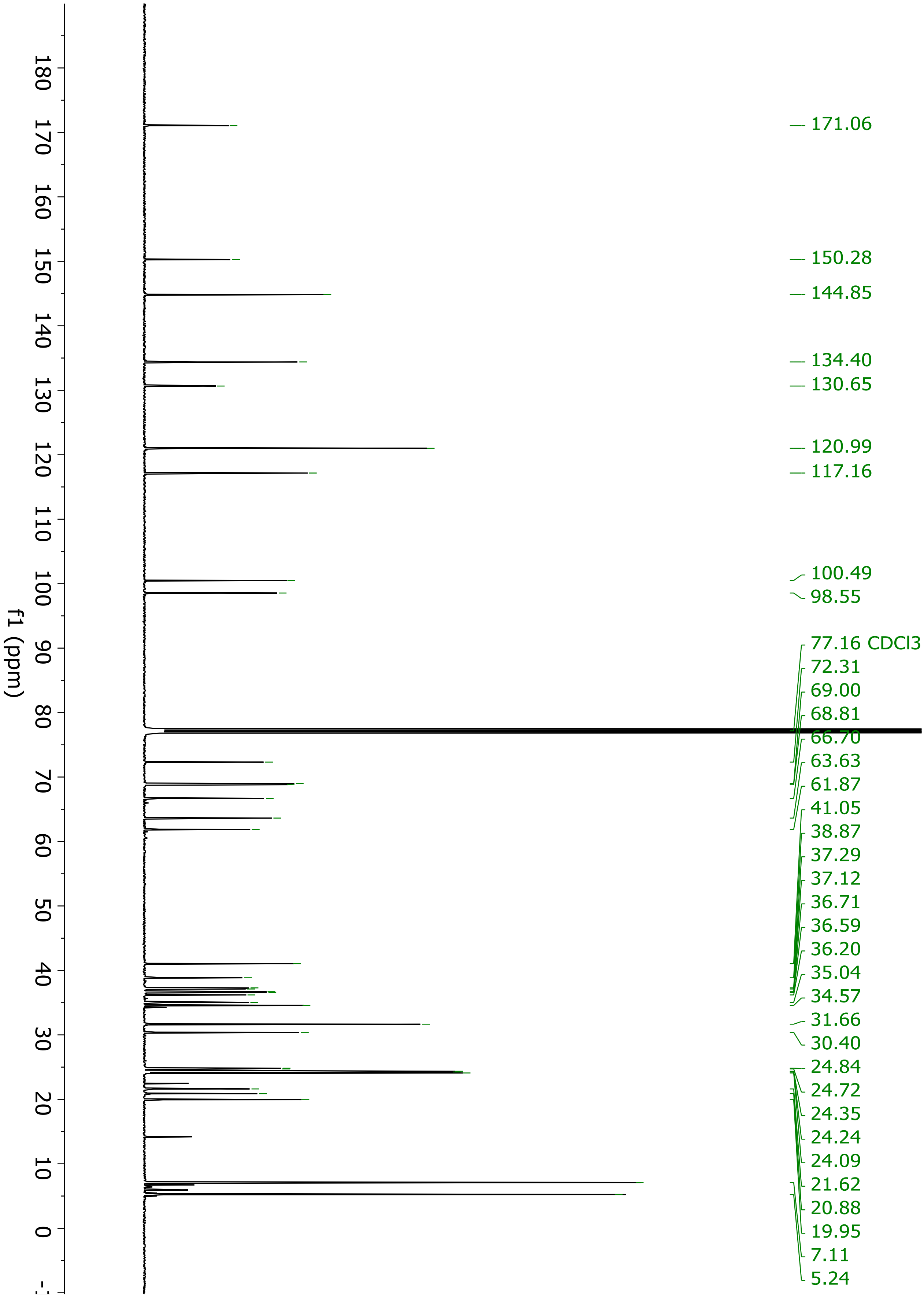

^{1}H NMR (600 MHz, $CDCl_3$) of compound **22**. <u>See procedure</u>.

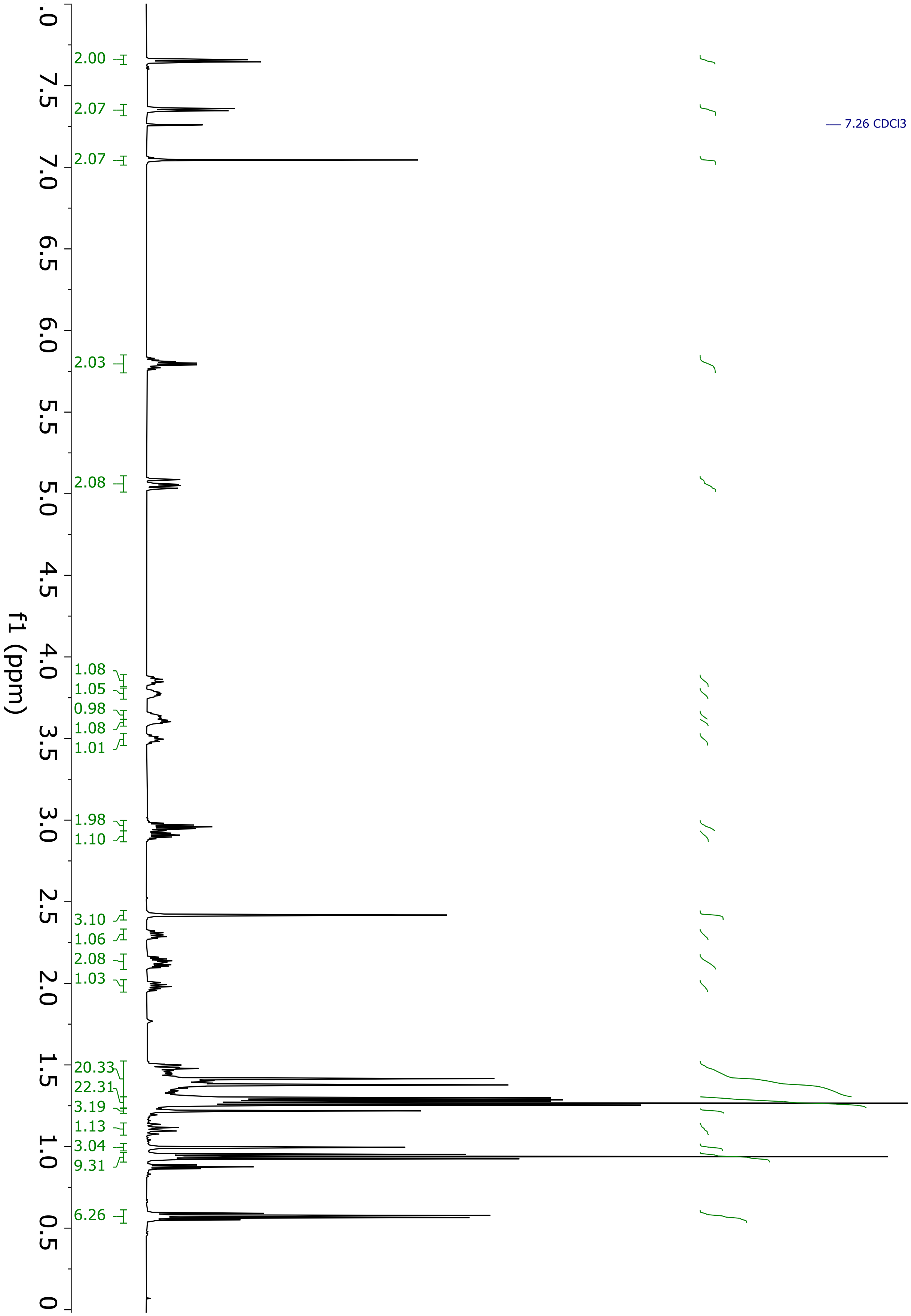

^{13}C NMR (151 MHz, $CDCl_3$) of compound **22**. *See procedure*.

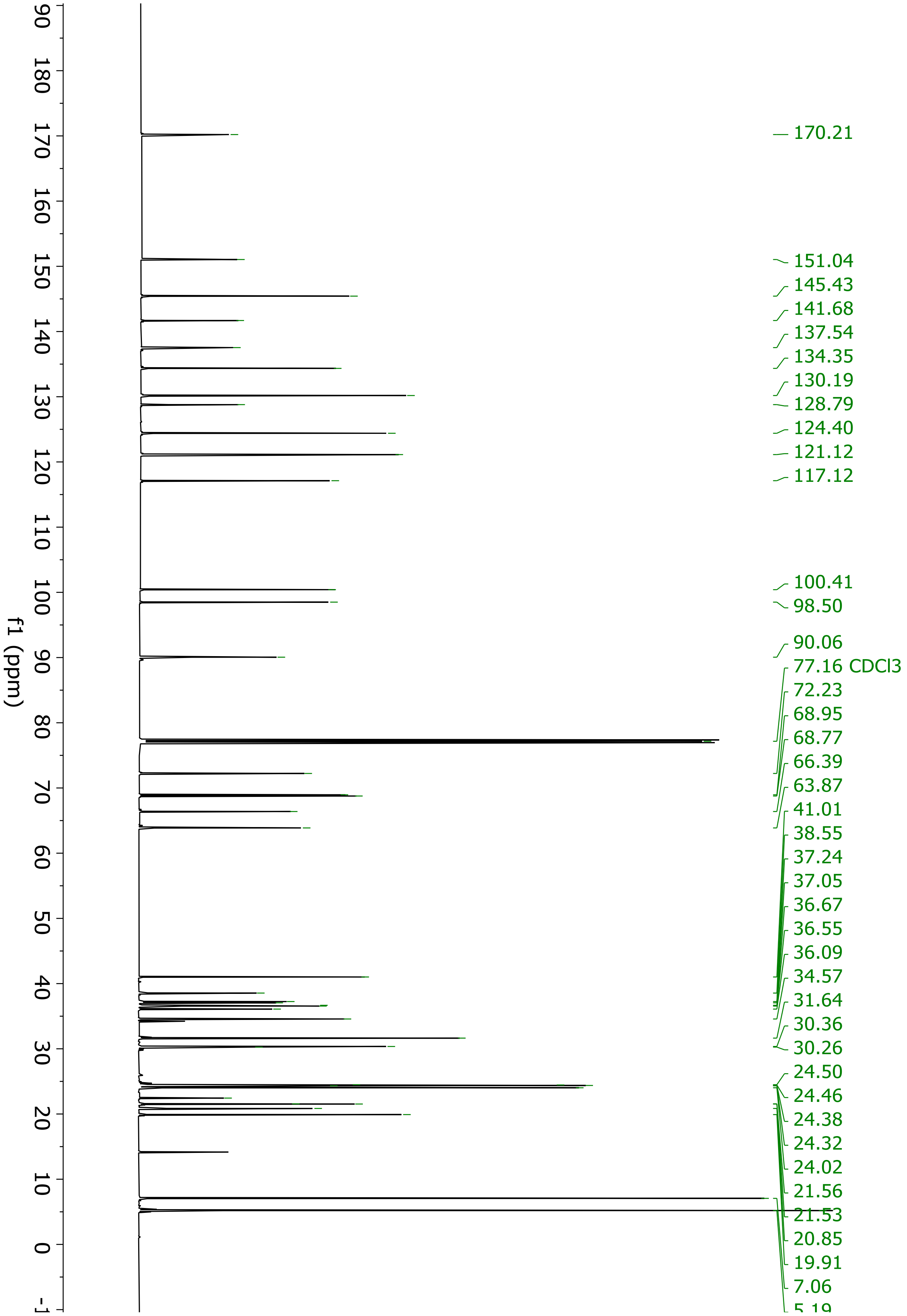

^{1}H NMR (600 MHz, $CDCl_3$) of compound **S24**. *See procedure*.

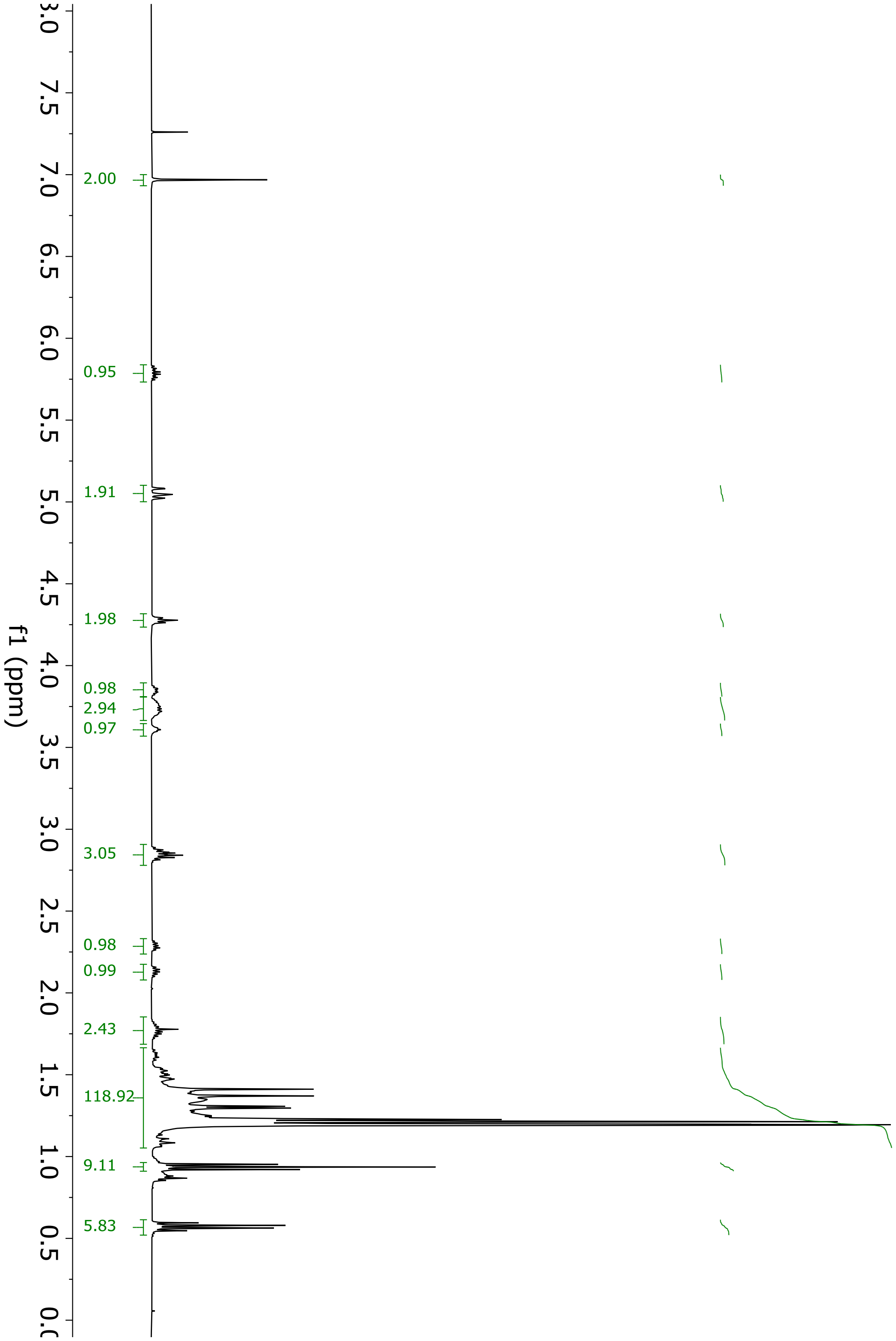

^{13}C NMR (151 MHz, $CDCl_3$) of compound **S24**. *See procedure.*

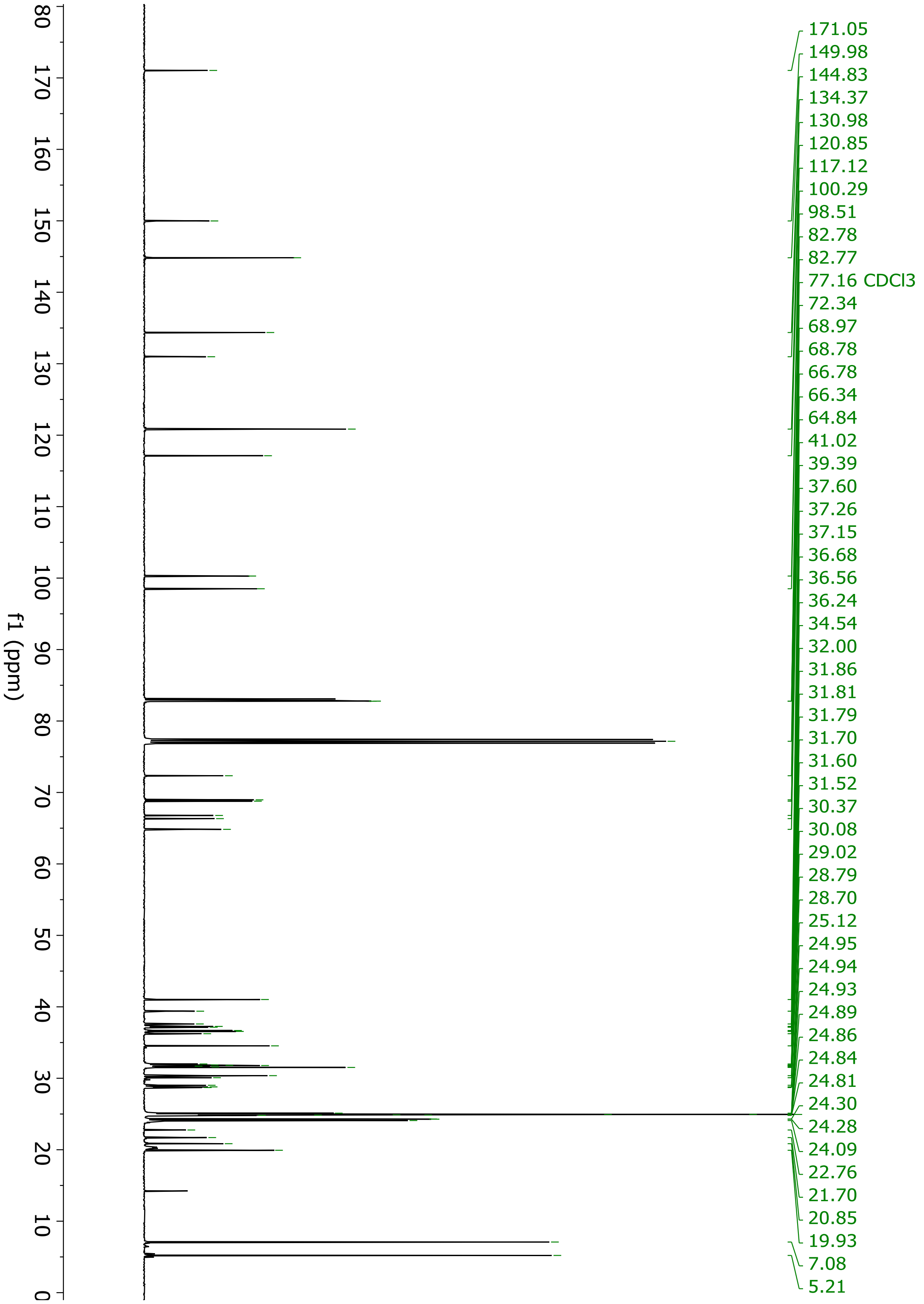

^{1}H NMR (600 MHz, $CDCl_3$) of compound **23**. *See procedure*.

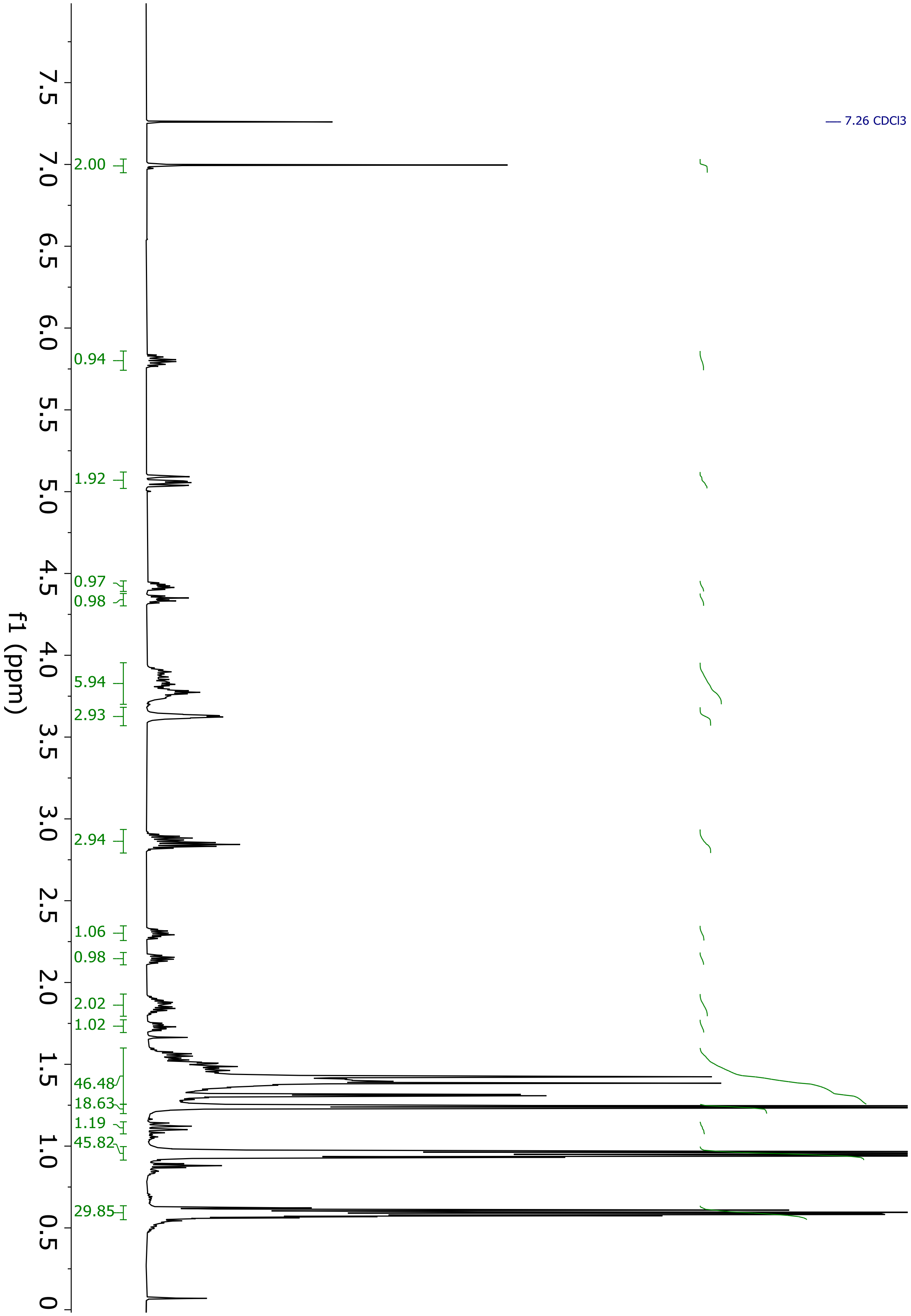

^{13}C NMR (151 MHz, $CDCl_3$) of compound **23**. *See procedure.*

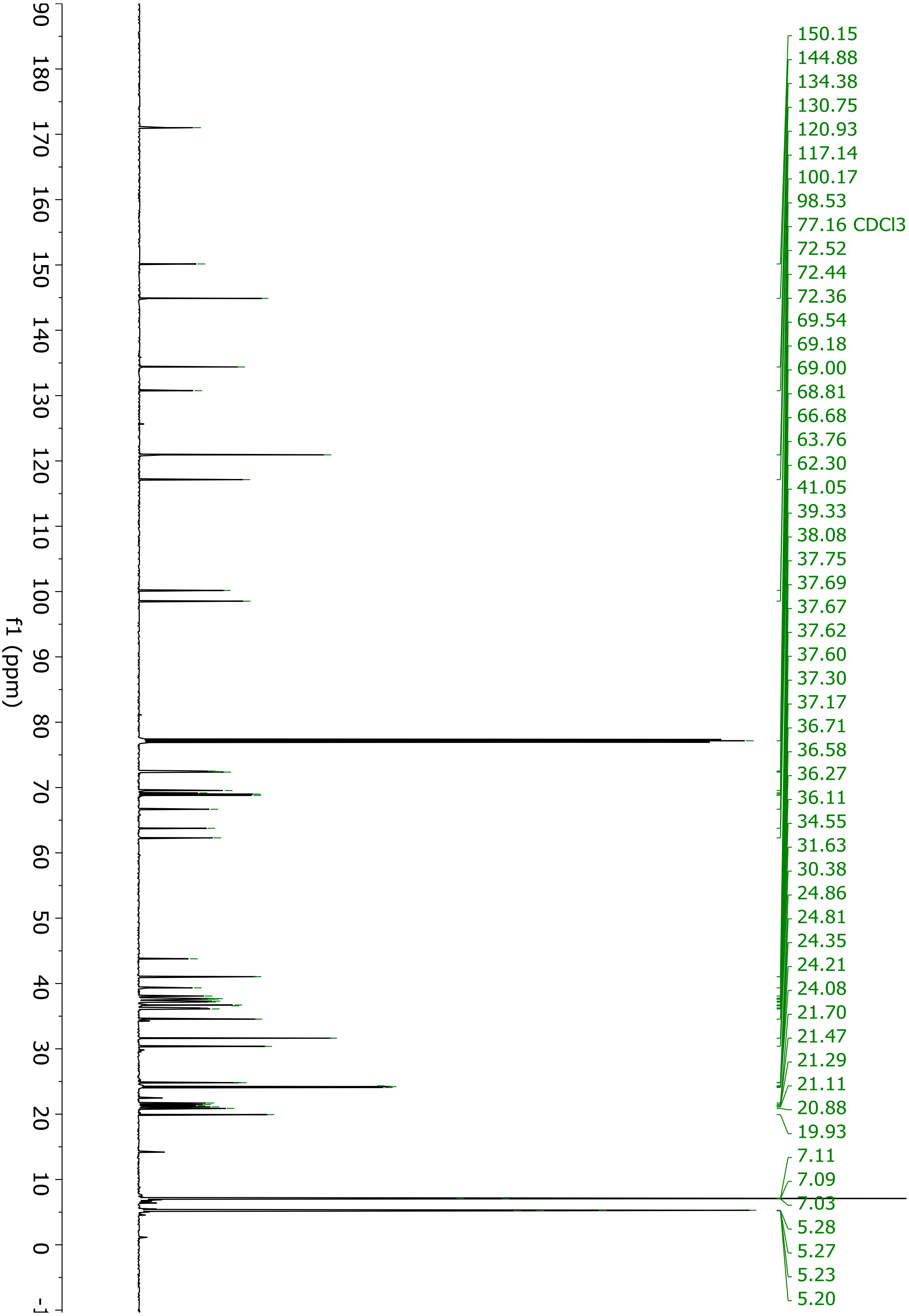

^{1}H NMR (600 MHz, $CDCl_3$) of compound **24**. *See procedure*.

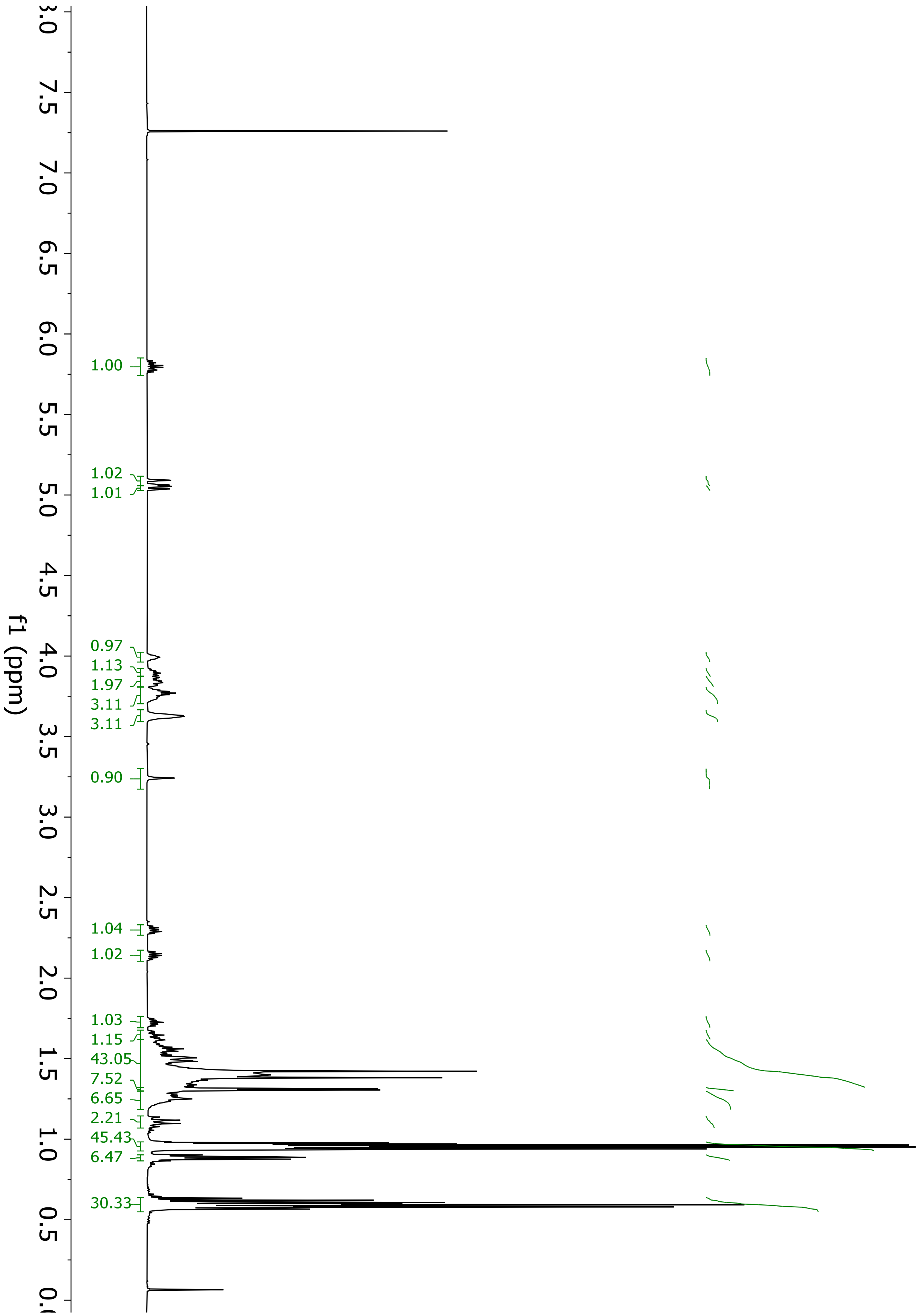

^{13}C NMR (151 MHz, $CDCl_3$) of compound **24**. See procedure.

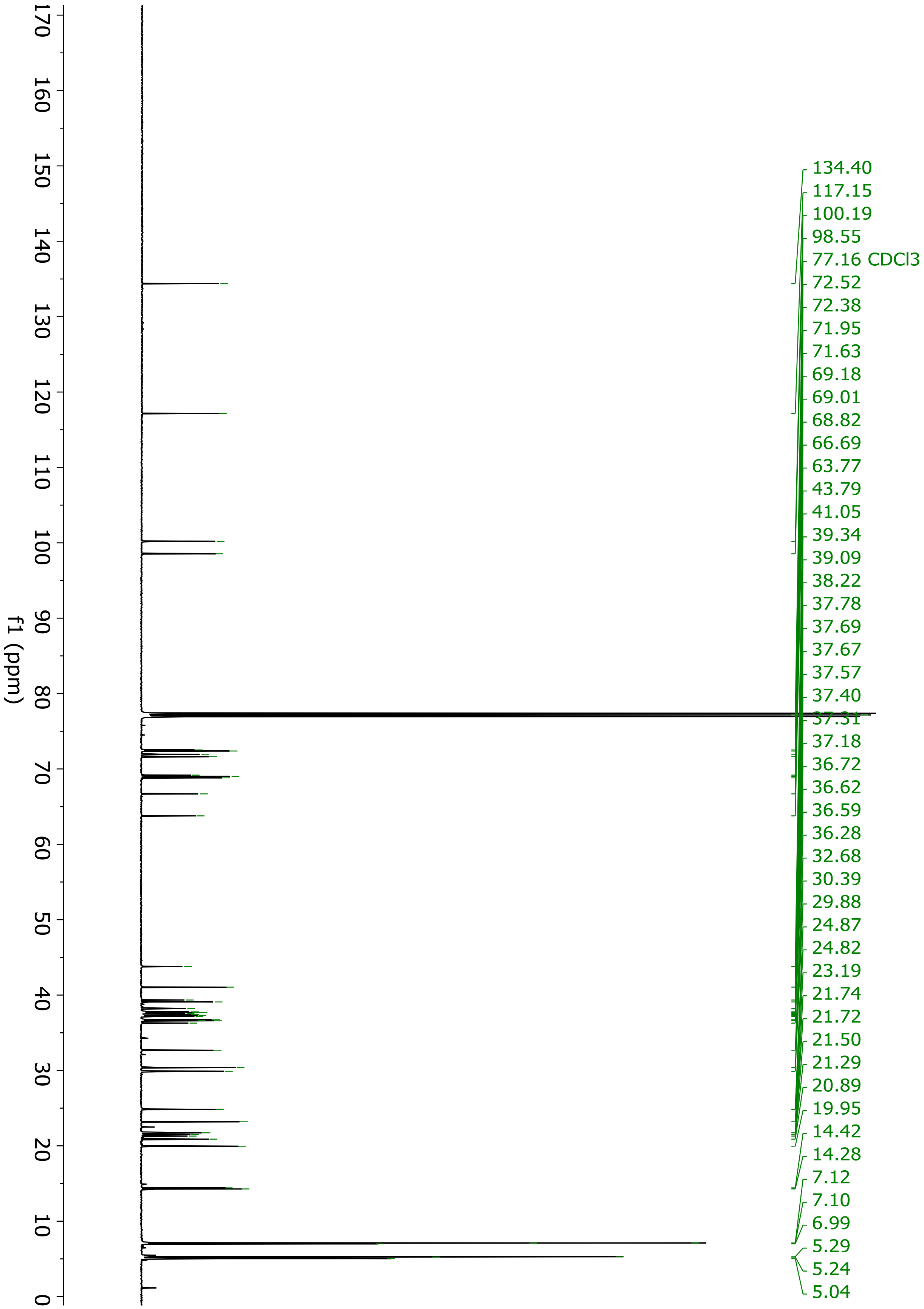

^{1}H NMR (600 MHz, $CDCl_3$) of compound **26**. See procedure.

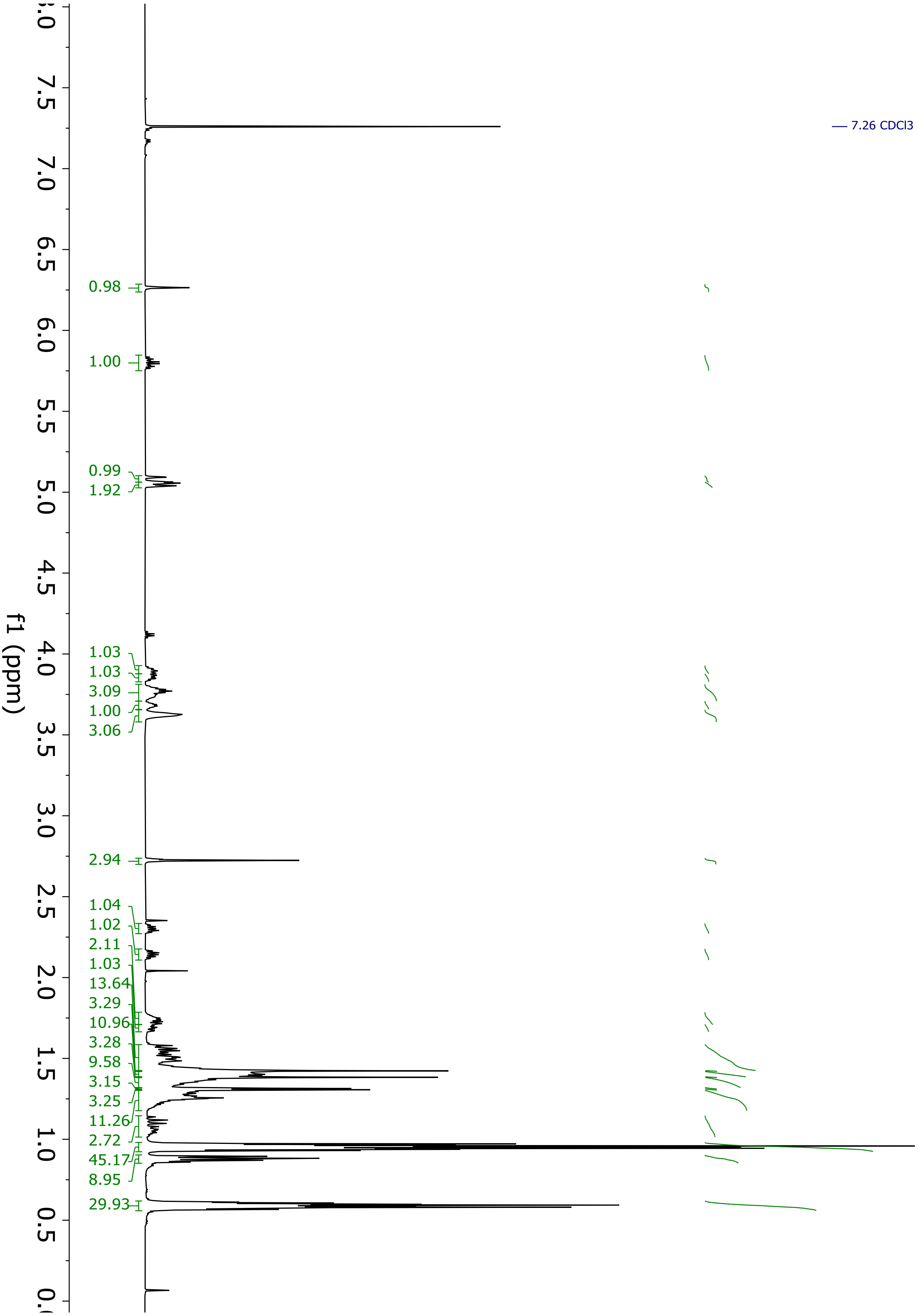

^{13}C NMR (151 MHz, $CDCl_3$) of compound **26**. *See procedure*.

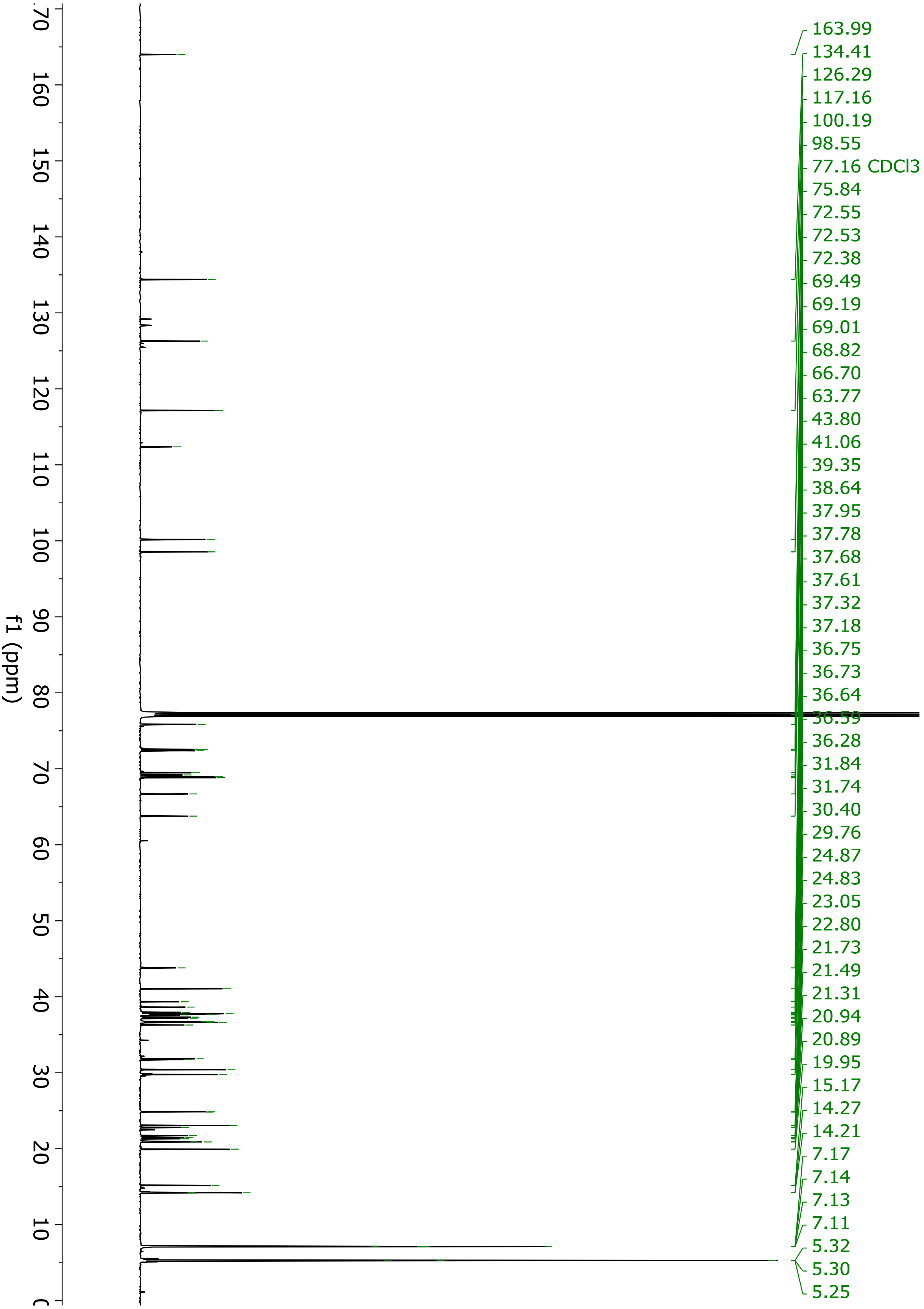

^{1}H NMR (600 MHz, $CDCl_3$) of compound **28**. See procedure.

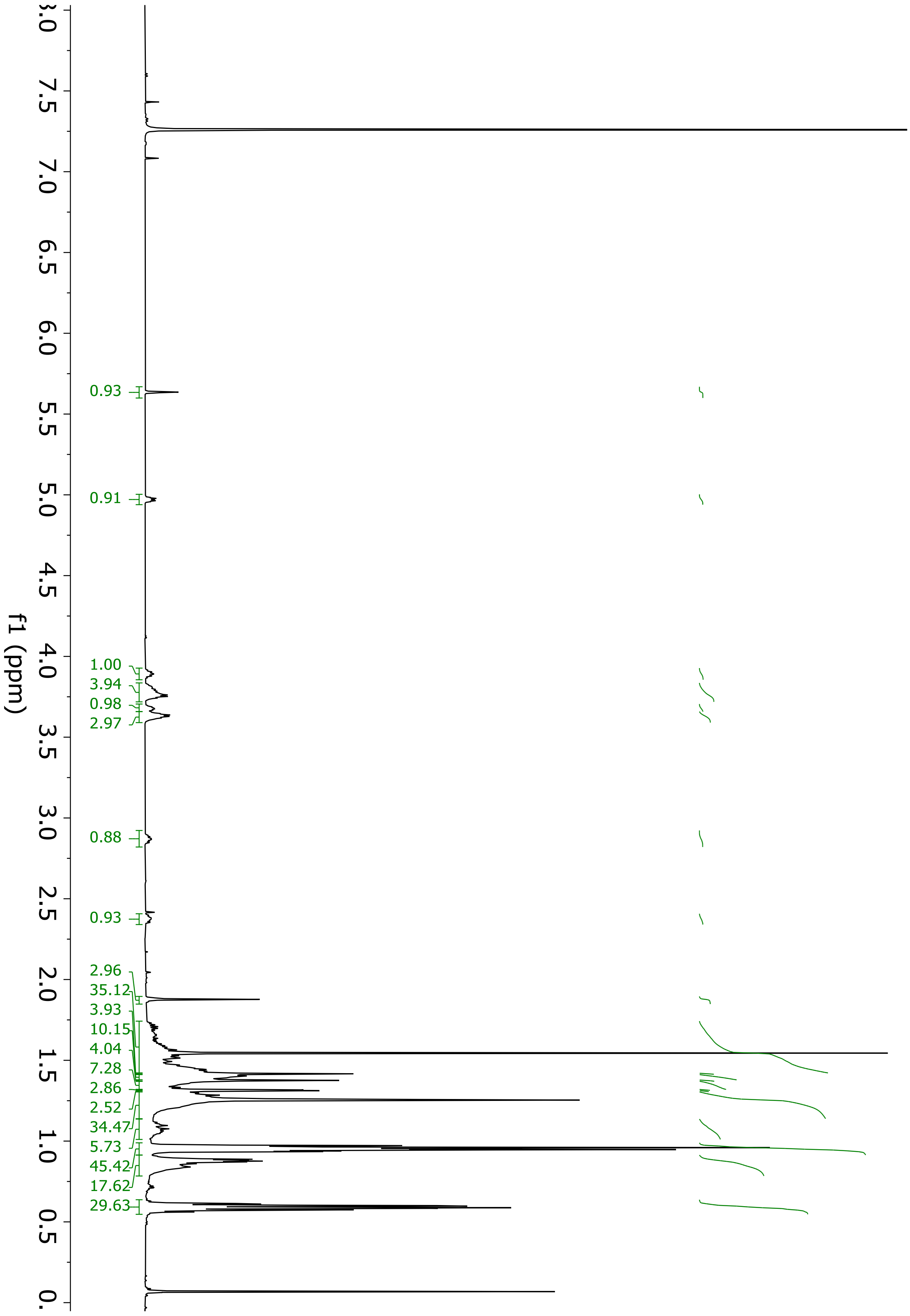

^{13}C NMR (151 MHz, $CDCl_3$) of compound **28**. *See procedure*.

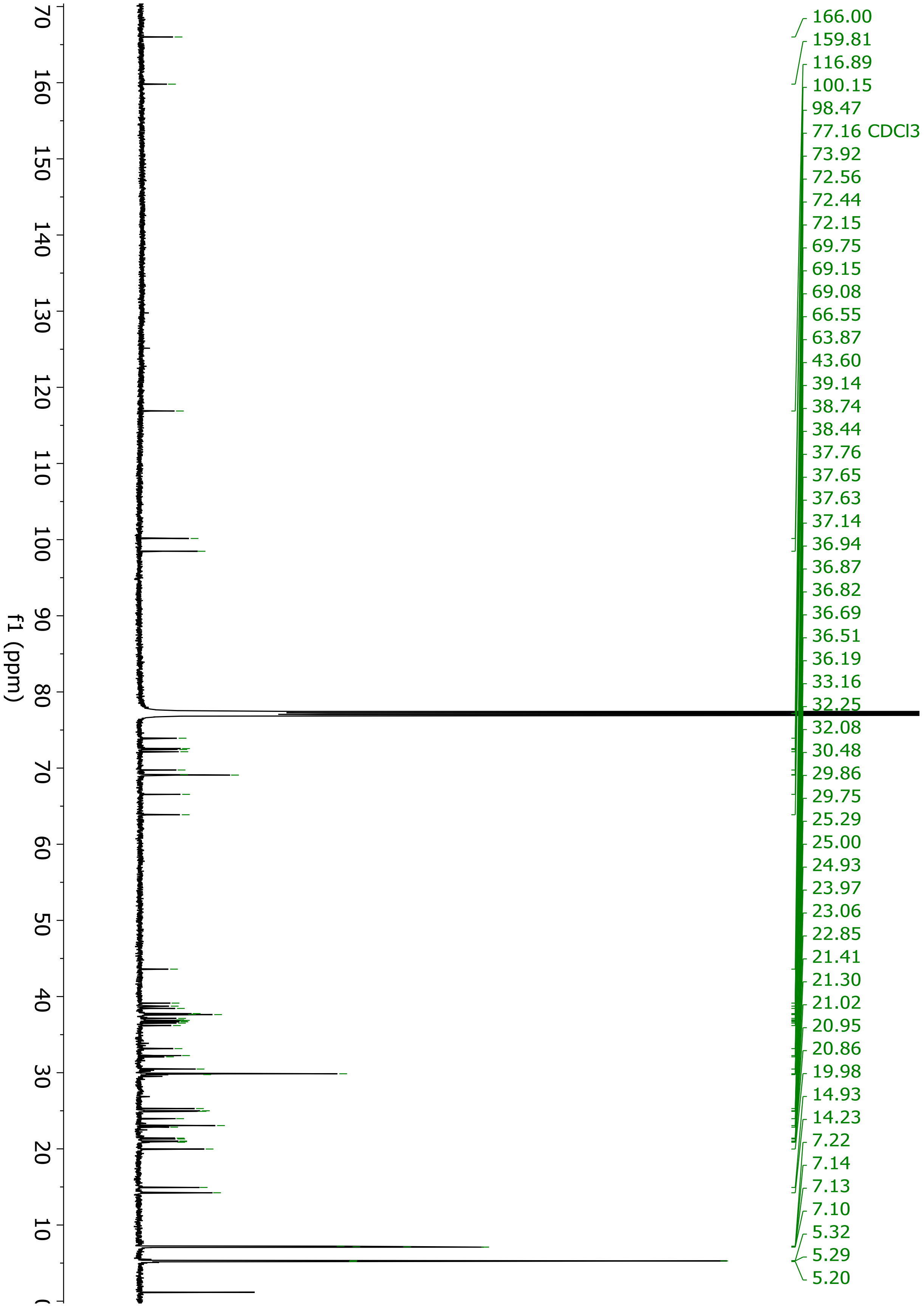

^{1}H NMR (600 MHz, pyridine-d_5) of synthetic *iso*-caylobolide B (**2**). [See procedure](#).

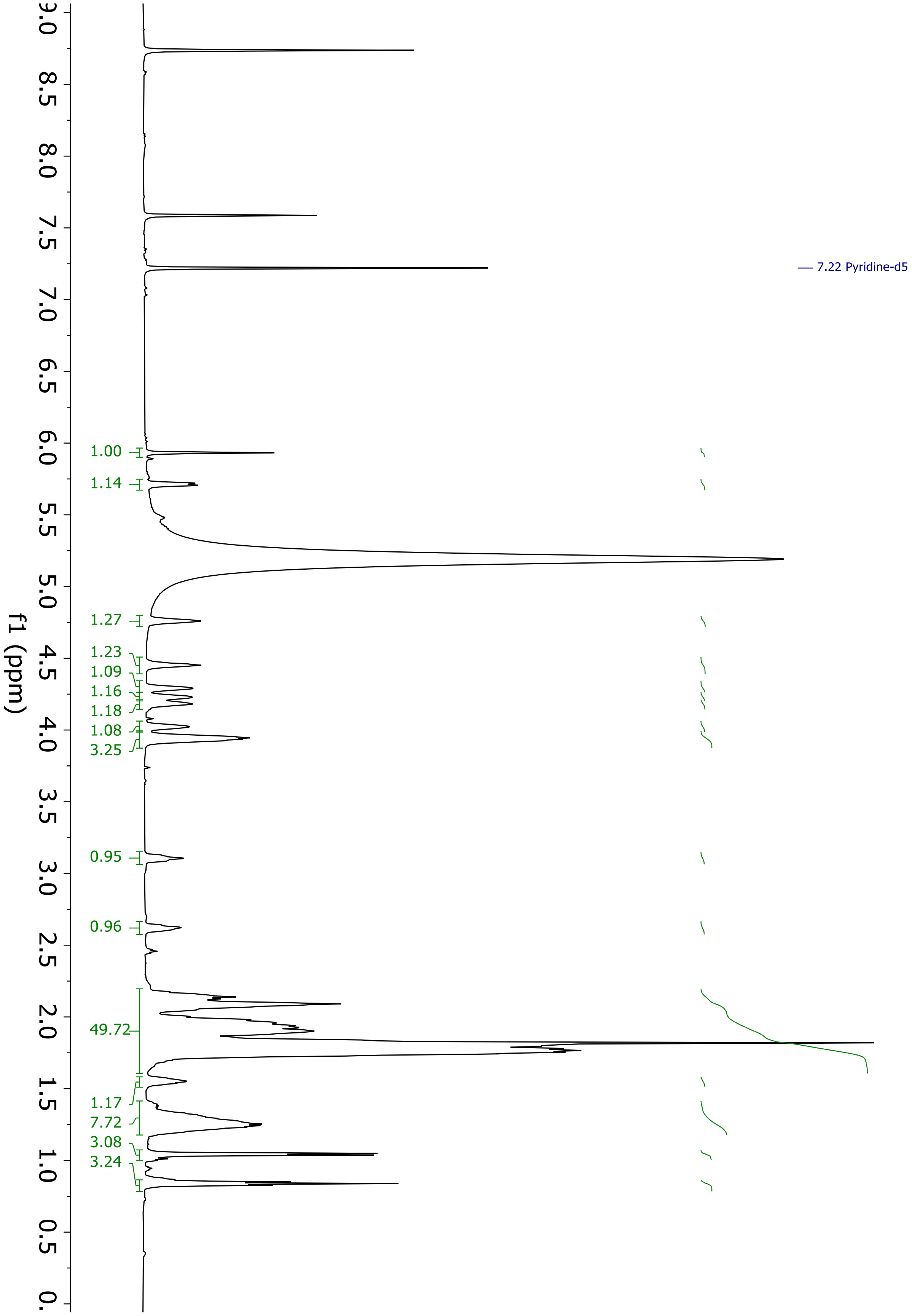

^{1}H NMR (600 MHz, pyridine-d_5) of synthetic *iso*-caylobolide B (**2**) with H_2O presaturation

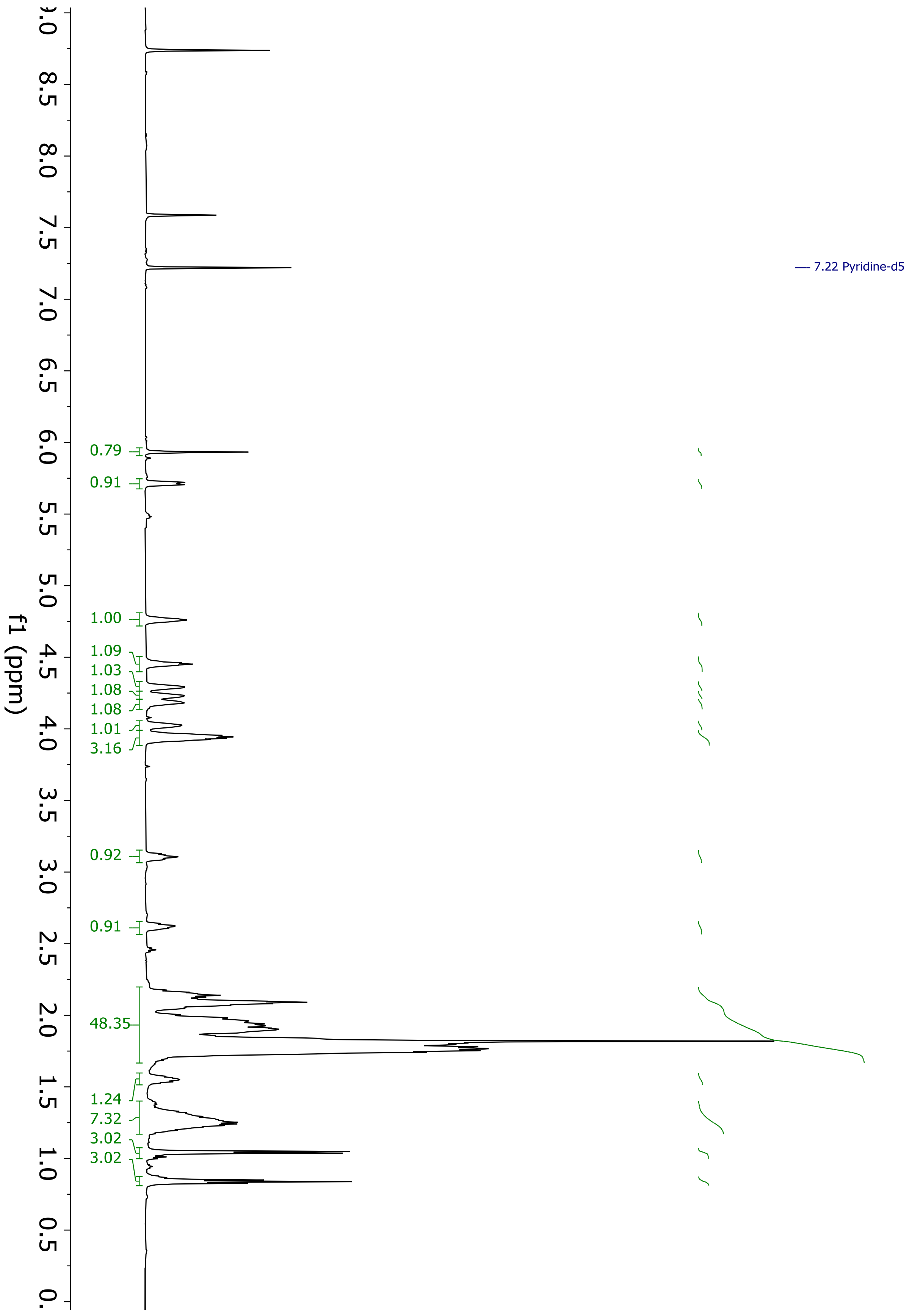

^{13}C NMR (151 MHz, pyridine-d_5) of synthetic *iso*-caylobolide B (**2**). See procedure.

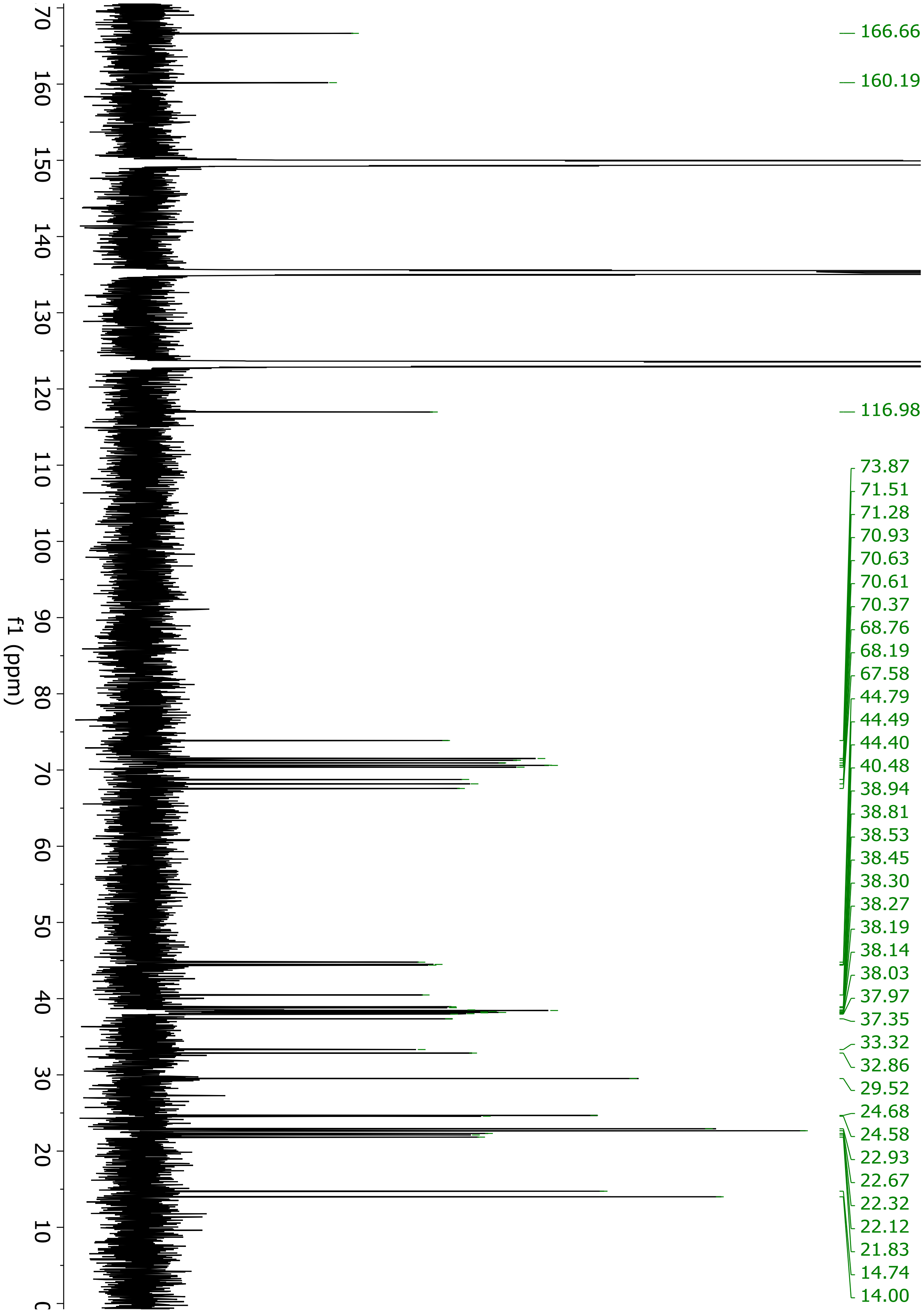

^{1}H NMR (500 MHz, $CDCl_3$) of compound **25**. See procedure.

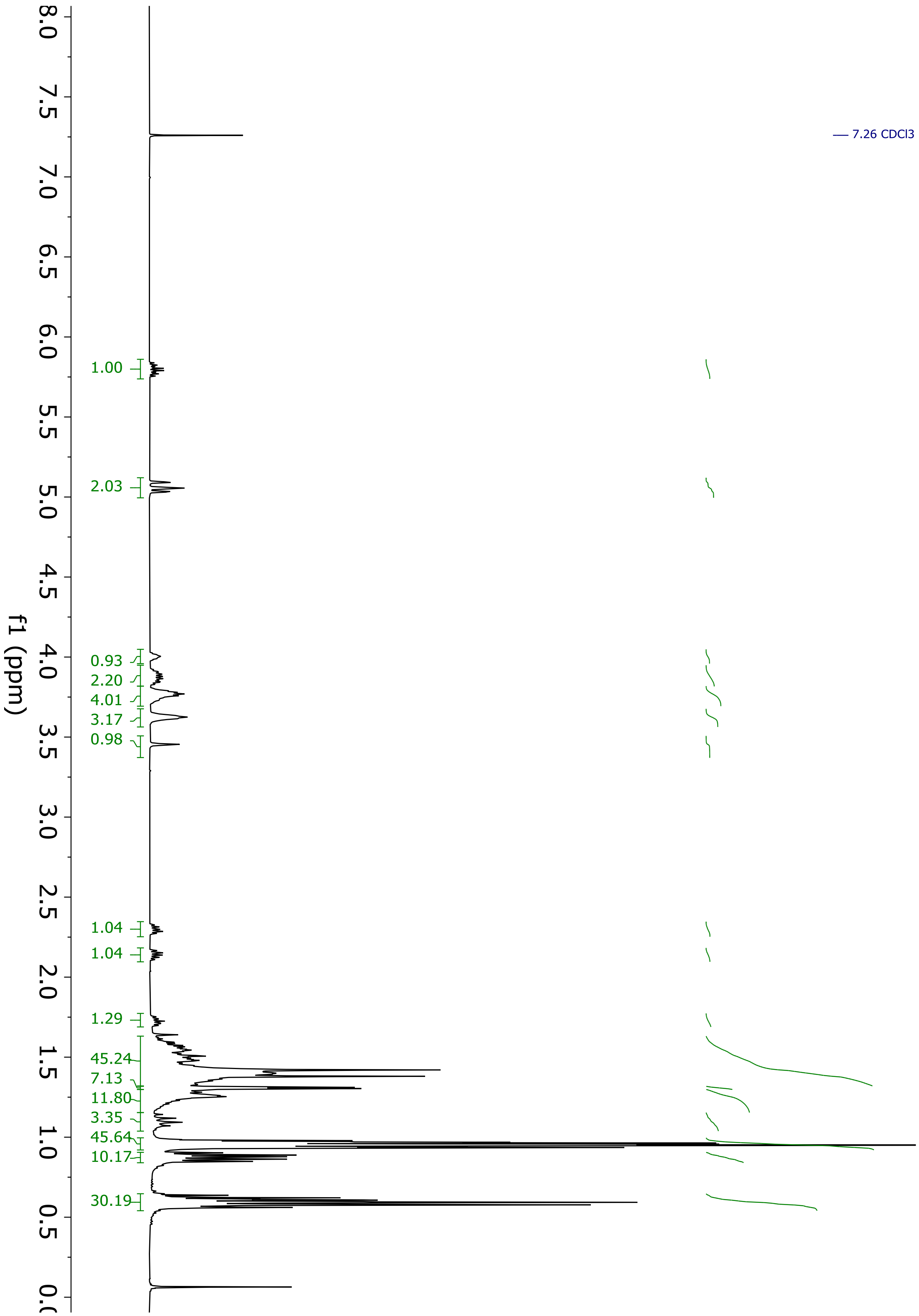

^{13}C NMR (126 MHz, $CDCl_3$) of compound **25**. *See procedure.*

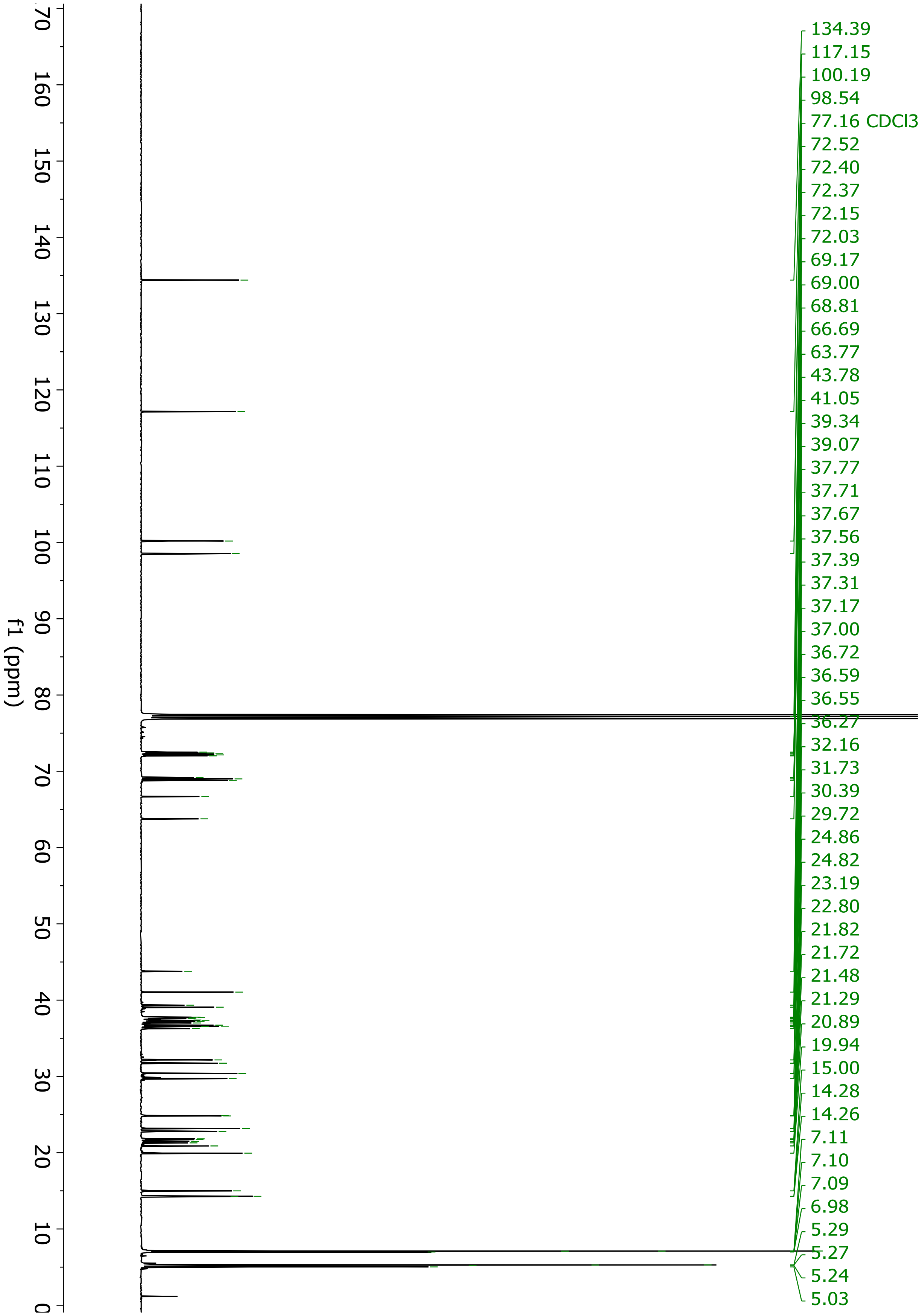

^{1}H NMR (500 MHz, $CDCl_3$) of compound **27**. See procedure.

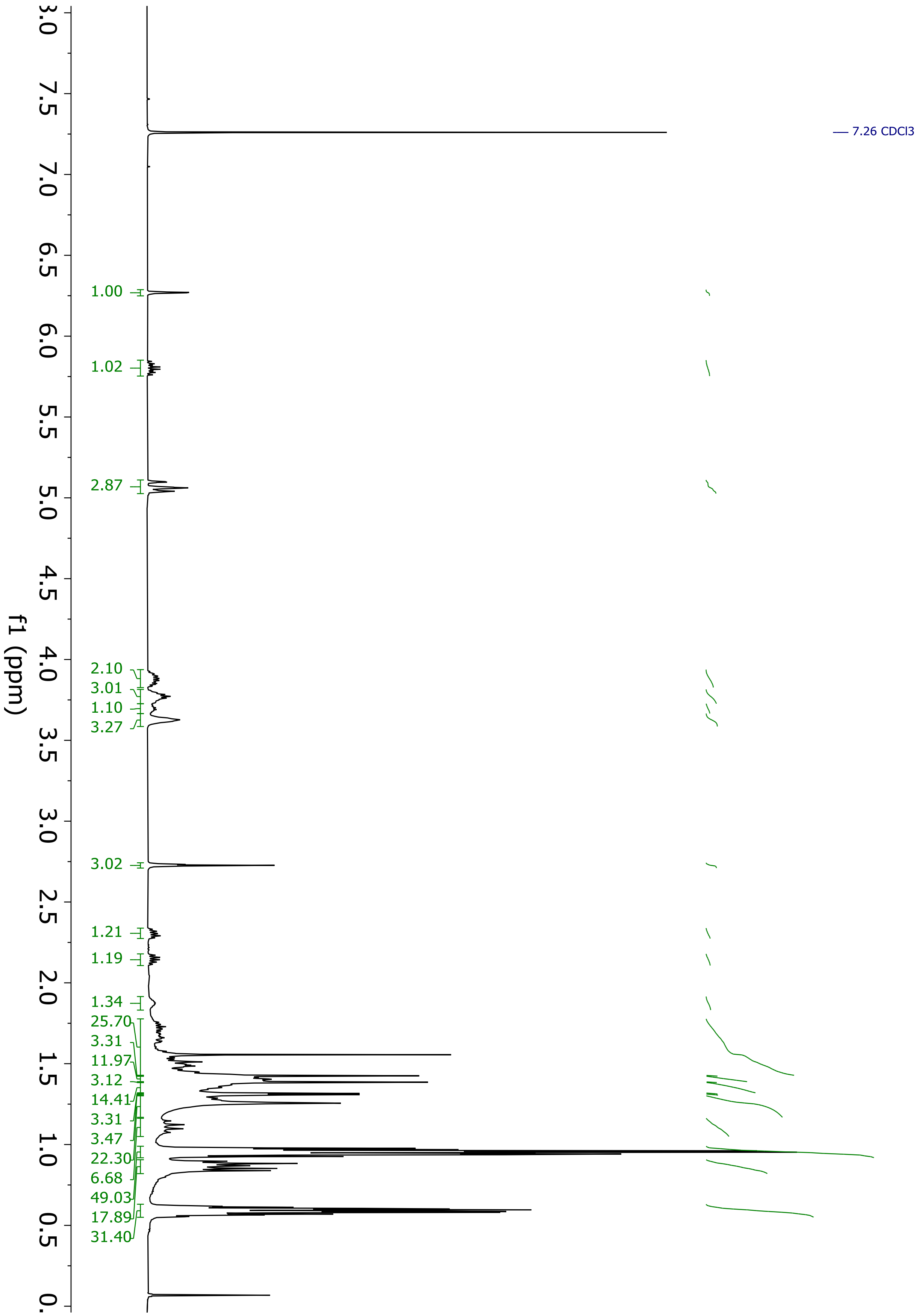

^{13}C NMR (126 MHz, $CDCl_3$) of compound **27**. *See procedure.*

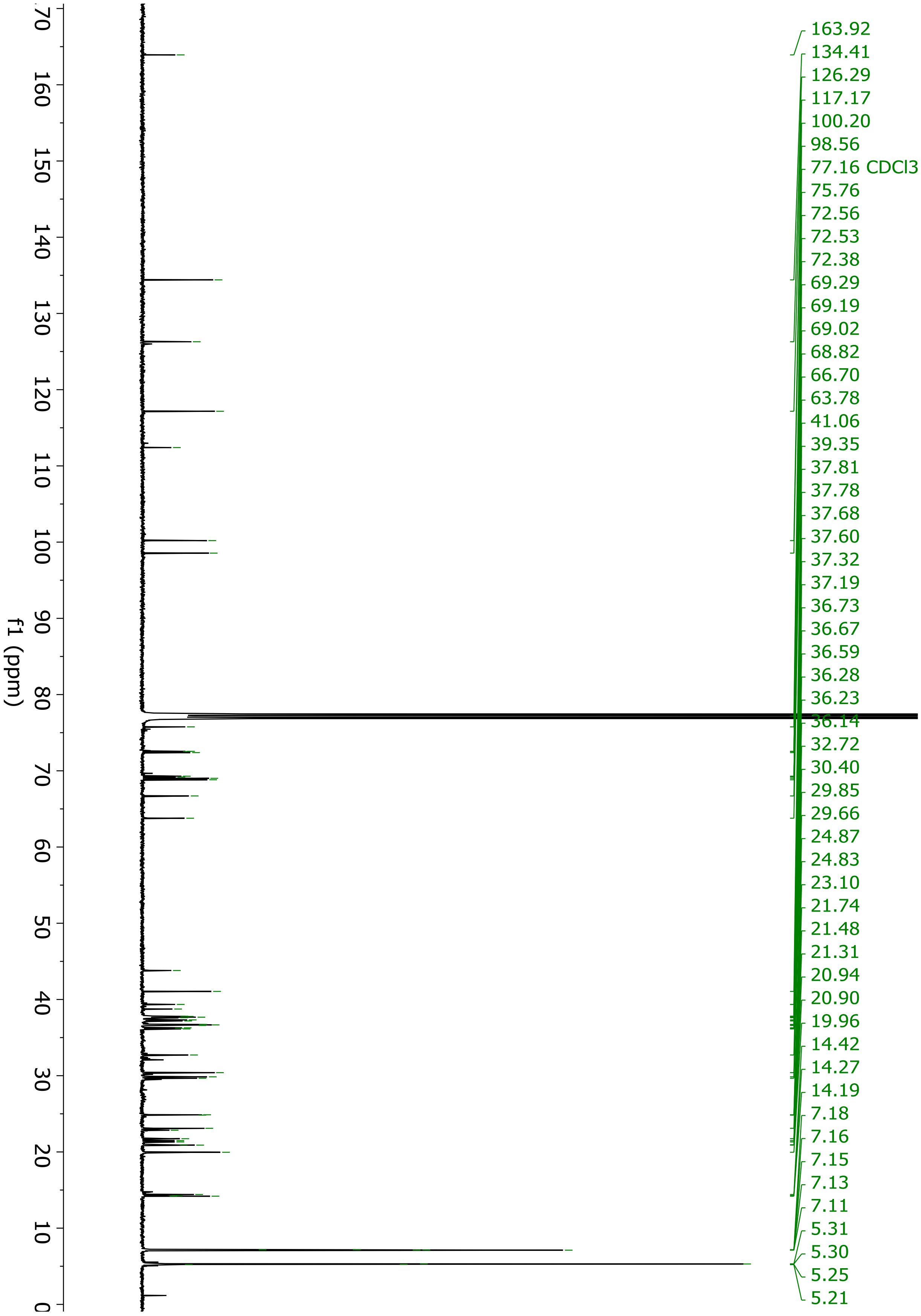

^{1}H NMR (500 MHz, $CDCl_3$) of compound **29**. *See procedure*.

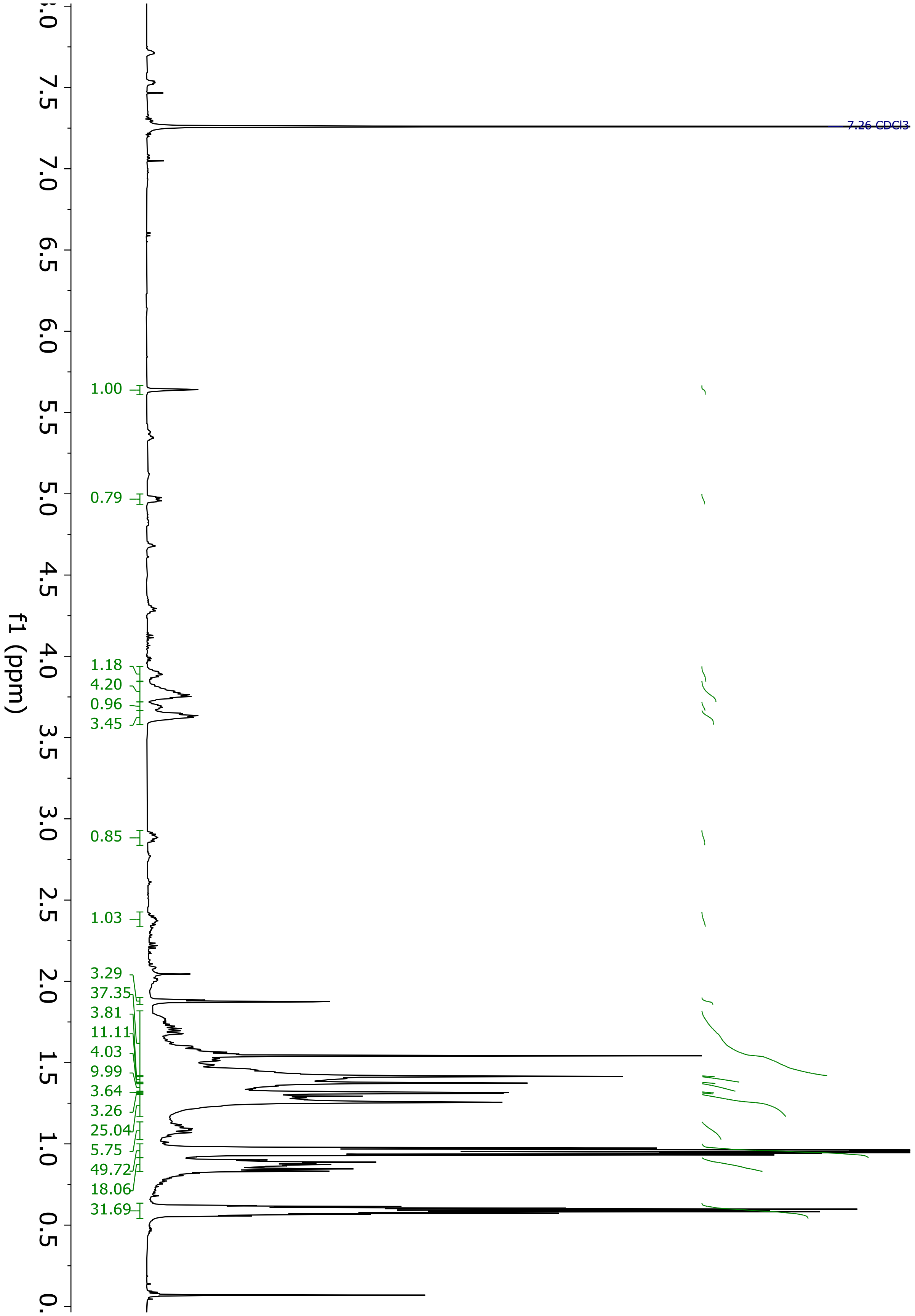

^{13}C NMR (126 MHz, $CDCl_3$) of compound **29**. *See procedure.*

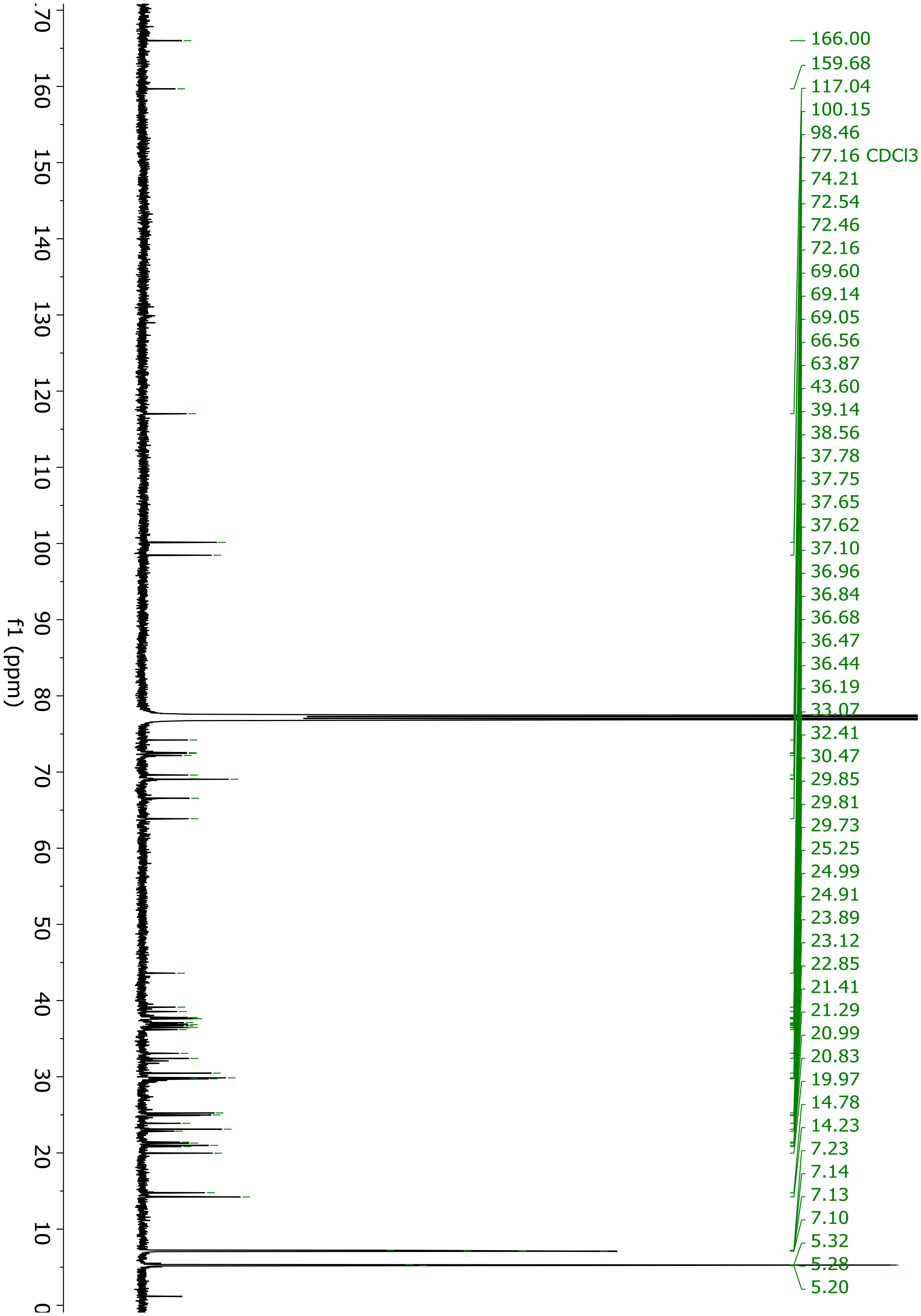

¹H NMR (700 MHz, pyridine-d_5) of synthetic caylobolide B (**1**). See procedure.

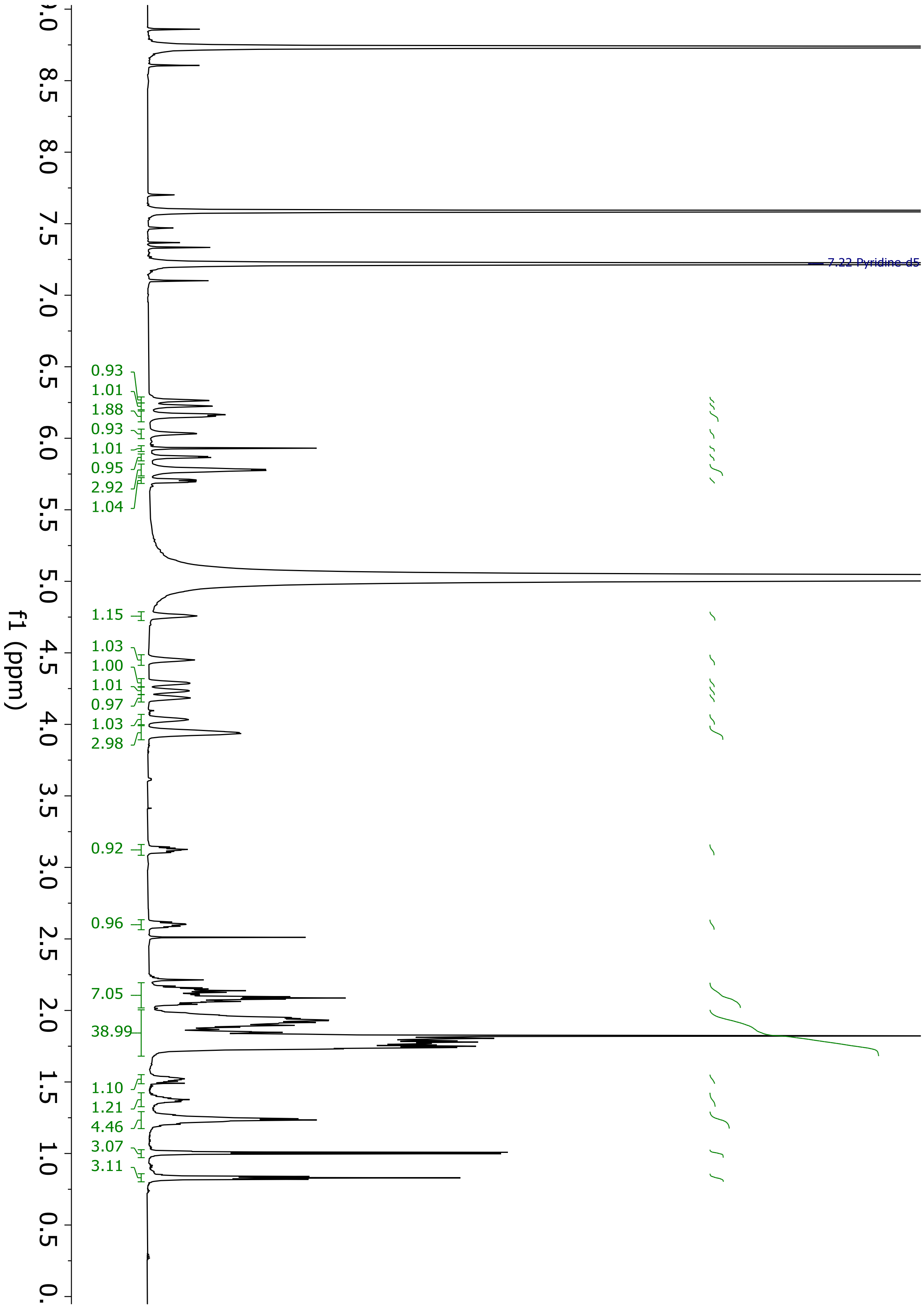

^{1}H NMR (700 MHz, pyridine-d_5) of synthetic caylobolide B (**1**) (with water presaturation)

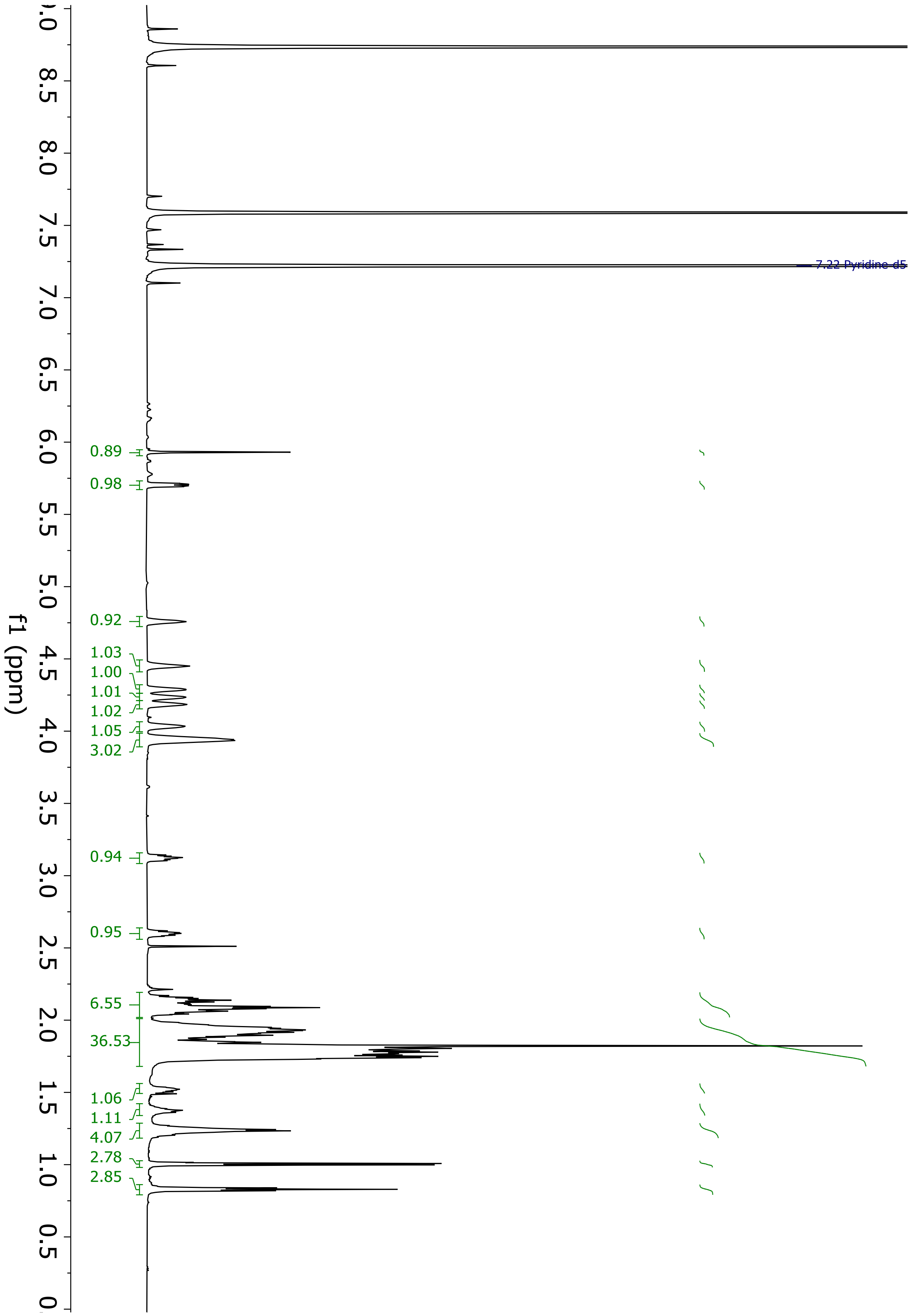

^{13}C NMR (151 MHz, pyridine-d_5) of synthetic caylobolide B (**1**). See procedure.

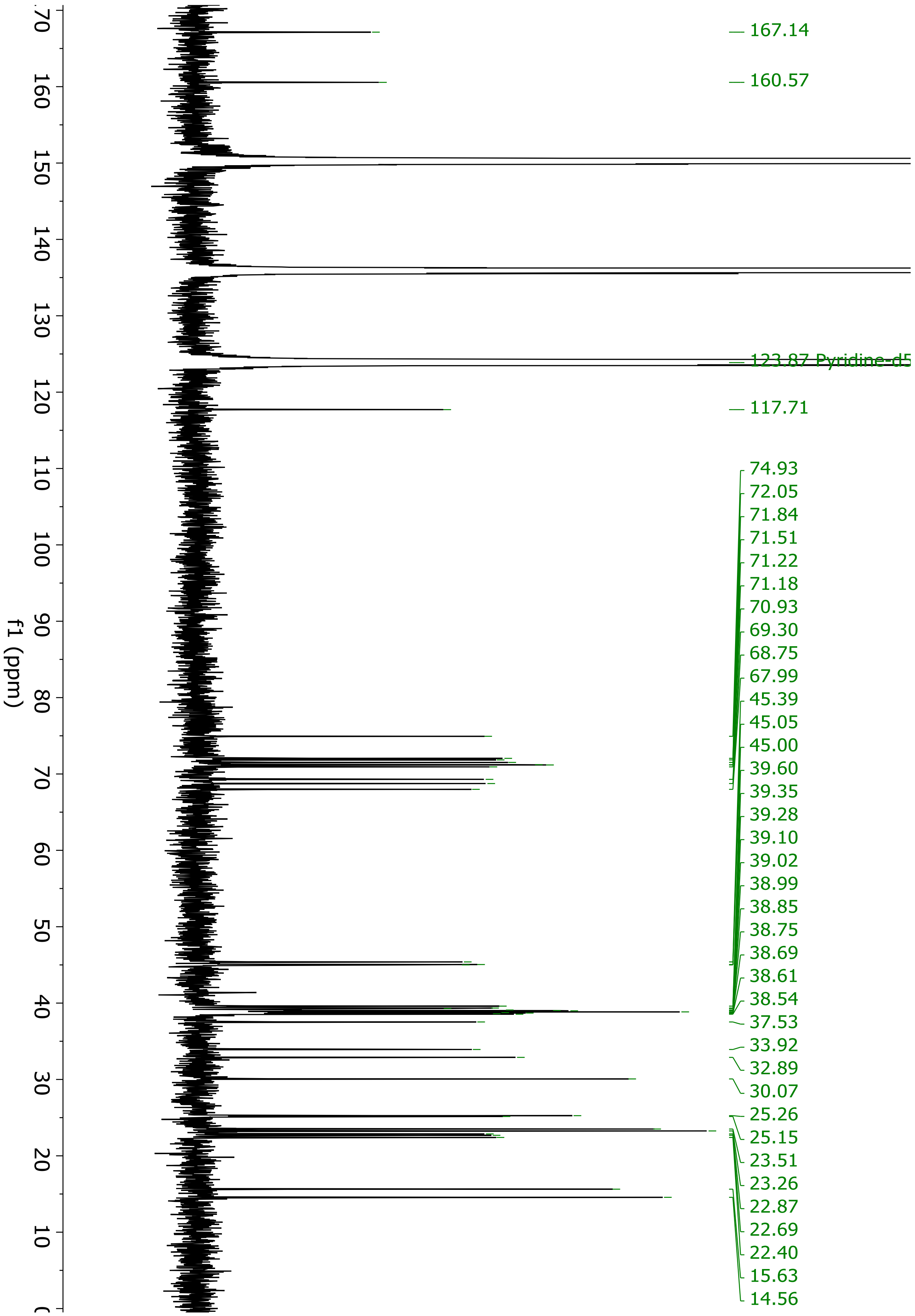

Supplement: Supplementary file 1 — Supporting Information [file ANIE-65-e23117-s001.pdf]
